# Exploring the Role of Nesprin1 in isoproterenol-induced heart failure: Insights into pathogenesis and therapeutic implications

**DOI:** 10.1371/journal.pone.0354482

**Published:** 2026-08-11

**Authors:** Qin Qin, Cenyuan Dong, Ziyi Zhou, Yangyuanzhi Liu, Mengpan Liu, Chunyu Cao, Lin Teng

**Affiliations:** 1 Department of Cardiology, The First College of Clinical Medical Science, China Three Gorges University & Yichang Central People’s Hospital, Yichang, China; 2 Hubei Key Laboratory of Ischemic Cardiovascular Disease, Yichang, China; 3 School of Basic Medicine, China Three Gorges University, Yichang, Hubei, People’s Republic of China; 4 Hubei Provincial Clinical Research Center for Ischemic Cardiovascular Disease, Yichang, China; Taylor’s University, MALAYSIA

## Abstract

**Objective:**

This study aimed to investigate the role of Nesprin-1 in the development and progression of isoproterenol (ISO)-induced heart failure (HF) and to elucidate the underlying molecular mechanisms.

**Methods:**

HF was induced in mice via daily intraperitoneal injections of ISO (5 mg/kg/day) for 24 consecutive days. Cardiac function was evaluated using transthoracic echocardiography to measure diastolic left ventricular diameters (LVDd), systolic left ventricular end diameter (LVDs), ejection fraction (EF), and fractional shortening (FS). The HF model was validated through gross cardiac observation, WGA staining, and Western blot analysis of B-type Natriuretic Peptide (BNP). Histological changes were assessed via H&E staining. Oxidative stress was evaluated by quantifying malondialdehyde (MDA) levels and superoxide dismutase (SOD) activity. The expression and localization of Nesprin-1, Connexin 43 (Cx43), DNA damage markers (53BP1, γH2AX), and epigenetic markers (H3K9me3, H3K27me3) were examined using immunofluorescence and Western blotting. The activation of the ERK signaling pathway was also analyzed.

**Results:**

Compared with the control group, ISO-induced HF mice exhibited significant increases in body/heart weight and cardiomyocyte cross-sectional area, alongside elevated BNP expression (*P* < 0.01). Echocardiography revealed increased LVDd and LVDs with markedly reduced EF and FS (*P* < 0.01). Histopathologically, the model group showed disorganized myocardial structures, vascular proliferation, and hemorrhage. Crucially, Nesprin-1 expression was significantly downregulated (*P* < 0.01), accompanied by abnormal nuclear membrane localization and morphological changes in myocardial cell nuclei. Significant nuclear distortions in the model group included increased area, perimeter, and length-to-diameter ratio alongside reduced roundness. Parallel to these changes, the model group showed increased malondialdehyde levels and decreased superoxide dismutase activity (*P* < 0.05). Notably, several markers showed highly significant differences (*P* < 0.01), including the downregulation of Connexin 43 and the upregulation of DNA damage markers γH2AX and 53BP1. Significant epigenetic remodeling, characterized by elevated H3K9me3 and H3K27me3, was also observed. Finally, a marked hyperactivation of the ERK signaling pathway was recorded, also reaching statistical significance at (*P* < 0.01).

**Conclusion:**

Our findings indicate that the downregulation of Nesprin-1 is closely associated with HF pathogenesis. In the ISO-induced HF model, diminished Nesprin-1 expression correlated with disrupted nuclear homeostasis, reduced Cx43 expression, severe oxidative stress, DNA damage accumulation, and epigenetic disturbances, which may be mediated through the abnormal activation of the ERK signaling pathway.

## Introduction

Heart failure (HF), or cardiac failure, signifies the heart’s diminished capacity to efficiently pump blood, resulting in venous congestion and inadequate arterial perfusion due to compromised systolic and/or diastolic functions [1–4]. This condition represents the terminal phase of heart disease and stands as a leading cause of cardiovascular-related mortality worldwide. HF affects approximately 1% to 2% of the global population, with a notably higher prevalence among the elderly [5–7]. Sympathetic nervous system (SNS) overactivity is a hallmark of HF, correlating with disease severity and prognosis. Under normal circumstances, the cardiac sympathetic nervous system enhances cardiac output, gene transcription, and metabolism by releasing neurotransmitters and engaging β-adrenergic receptors (β-AR) [8,9]. However, excessive SNS activation detrimentally affects the cardiovascular system, potentially precipitating or exacerbating HF [10,11]. This overdrive leads to heightened heart rate and contractility, inducing a sustained high-load state that depletes cardiac energy reserves, impeding cardiac function. Furthermore, it promotes adverse structural cardiac changes such as ventricular hypertrophy and atrial dilation, diminishing cardiac pump efficiency [12–14]. Increased arterial vasoconstriction further strains the heart by elevating vascular tension. Excessive sympathetic stimulation also incites inflammation and oxidative stress, damaging myocardial cells and hastening disease progression. Disruption of neuroendocrine system balance, including the renin-angiotensin-aldosterone system, can exacerbate fluid retention and increase blood volume, exacerbating cardiac workload [15–18]. Current HF management strategies primarily target the sympathetic nervous system with medications like β-blockers, angiotensin-converting enzyme inhibitors, angiotensin II receptor blockers, and aldosterone receptor antagonists. Despite these interventions, HF incidence, readmission rates, and mortality remain high, underscoring the need for novel therapeutic approaches [19–23].

Traditionally, HF research focused on intracellular factors, neglecting the nuclear envelope’s role. The nuclear envelope (NE) is pivotal, spatially segregating nuclear genetic material and regulating macromolecular transport [24]. The Linker of Nucleoskeleton and Cytoskeleton (LINC) complex, anchored to the NE, includes Nesprin proteins, which bridge the nuclear and cytoskeletal proteins, contributing to cellular structure and function. Nesprin mutations are implicated in various diseases, including Dilated Cardiomyopathy (DCM) and Emery-Dreifuss Muscular Dystrophy (EDMD), highlighting their importance in muscle physiology [25–27]. Nesprin family proteins, integral to the NE, include Nesprin-1 and Nesprin-2, which interact with actin, intermediate filaments, and the nuclear envelope, influencing cell signaling, morphology, and mechanical properties [28]. These proteins regulate gene expression, chromatin structure, and epigenetic modifications, impacting cell function and pathology. Mutations in Nesprin-1 and Nesprin-2 are associated with abnormal nuclear morphology, positioning, and chromatin organization, contributing to DCM and EDMD. Nesprin alterations also affect intercalated disc protein localization, leading to conduction defects in Nesprin-associated cardiomyopathies [27,29,30].

Given this background, we hypothesize that the downregulation of Nesprin-1 disrupts the structural linkage between the nucleoskeleton and cytoskeleton. This disruption likely impairs nuclear homeostasis, interferes with intracellular signal transduction, and causes abnormal transcriptional regulation, ultimately driving adverse myocardial remodeling, contractile dysfunction, and the progression of heart failure.

## Materials and methods

### Drugs and Chemicals

MedChemExpress provided isoproterenol (HY-B0468) and isoflurane, while Biosharp supplied 4% paraformaldehyde (BP003), hematoxylin-eosin staining (BP019), and other staining-related reagents. Sinopharm Chemical Reagent offered basic reagents such as paraffin (69019361) and xylene (10023418). Antibodies were mainly sourced from Boster, including Cx43 antibody (A00599), BNP antibody (M01186-3), 53BP1 antibody (BM5099), γH2AX antibody (BM4148), H3K9me3 antibody (BM5079), H3K27me3 antibody (BM4341), and ERK antibody (BM4156). Protein-related reagents were primarily obtained from Beyotime, including RIPA lysis buffer (P0013B) and BCA protein assay kit (P0010). Additionally, Amresco provided electrophoresis reagents such as TEMED (Amresco 0761) and acrylamide (Exp2016109), while Jackson supplied HRP-labeled secondary antibodies, specifically HRP-conjugated goat anti-rabbit (BA1054), HRP-conjugated rabbit anti-goat (BA1060), HRP-conjugated goat anti-mouse (BA1051), and HRP-conjugated goat anti-rat (BA1058). Other essential reagents included WGA stock solution (BBL-0351) from Sigma-Aldrich, DAPI stock solution (C1002) from Solarbio, and cytoplasmic/nuclear protein extraction kit (KGP150) from Nanjing KeyGen Biotech.

### Establishment of Isoproterenol-Induced Heart Failure Model in Mice

The male C57BL/6 mice weighing 20-22g were purchased from the Animal Experimental Center of China Three Gorges University. All mice were housed in specific pathogen-free (SPF) animal facilities at China Three Gorges University (Yichang, Hubei, China). Twenty-four C57BL/6 mice were randomly divided into two groups: model group and control group. In the model group, mice were administered intraperitoneal injections of isoproterenol (ISO) at a dosage of 5 mg/kg. In the control group, mice were administered intraperitoneal injections of an equivalent volume of medical-grade physiological saline. Two groups received intraperitoneal injections continuously for 24 days.

### Cardiac function assessment

Mice were anesthetized with isoflurane, and after achieving stable anesthesia, the hair in the chest area was removed. The mice were then placed in a supine position and fixed on an ultrasound examination table. Ultrasound coupling agent was applied to the chest area. Using small animal ultrasound, M-mode images of the long axis and short axis of the mouse heart were obtained. Parameters including ejection fraction (EF), fractional shortening (FS), left ventricular end-diastolic diameter (LVEDd), and left ventricular end-systolic diameter (LVESd) were measured and calculated to assess cardiac function in mice. Statistical analysis was performed to analyze the cardiac function of mice.

### Measurement of Body and Heart Weight

All animal procedures were performed in accordance with the National Institutes of Health guidelines and were approved by the Committee of Animal Experiments at the China Three Gorgers University (No. 2023660H). Throughout the 24-day isoproterenol administration period, all mice were strictly monitored daily. To minimize suffering, humane endpoints were pre-established; however, no animals reached these endpoints prior to the scheduled conclusion of the study. All intraperitoneal injections and handling were performed by trained personnel to minimize stress. At the end of the experiment (at least 24 hours after the final dose), mice were deeply anesthetized via inhalation of 3% isoflurane. The adequacy of anesthesia was confirmed by the absence of the pedal withdrawal reflex. Following confirmed deep anesthesia, mice were euthanized via cervical dislocation, which served as a secondary physical method to ensure a humane death. The hearts were immediately harvested, rinsed in ice-cold physiological saline, and trimmed of excess connective tissue and vessels. Total heart weight was recorded. Myocardial samples were either snap-frozen in dry ice and stored at −80°C for molecular analysis or fixed in 4% paraformaldehyde for histological evaluation. All efforts were made to minimize suffering in the animal experiments.

### Pathological Section Staining

**HE staining:** After dehydration, embedding, and sectioning of tissue fixed with 4% paraformaldehyde, the paraffin sections were dewaxed to water. Stain with hematoxylin for 4 minutes, followed by 2-minute rinsing with tap water to remove excess stain. The sections were then differentiated with 0.8% hydrochloric acid alcohol for 2 seconds, rinsed again with tap water, immersed in eosin solution for 20 seconds, adjusted with 95% ethanol for 5 seconds, and dehydrated with anhydrous ethanol for 2 minutes. Finally, the sections are sealed with a transparent environmental agent and observed under a microscope.

**WGA staining:** The paraffin sections were sequentially dewaxed with eco-friendly degreasing agents (1), (2), and (3) for 10 minutes each, followed by 5-minute immersion in anhydrous ethanol, 95% ethanol, and 75% ethanol respectively. The sections underwent three 3-minute rinses with distilled water. Antigen retrieval was performed by high-pressure treatment of the sections in boiling citric acid (pH6.0) solution for 2 minutes. After cooling, the sections were immersed in distilled water for three 5-minute washes. A circular mark was made on the section using a histological pen, then it was immersed in TBST buffer. A 1:200 diluted WGA working solution (50 uL/section) was added and incubated under light protection for 30 minutes, followed by three 5-minute TBST washes. After removing TBST, a 1:500 diluted DAPI working solution (50 uL/section) was applied to stain nuclei for 5 minutes. Following TBST rinsing, the sections were mounted with anti-fluorescence quenching medium and stored at 4°C under light protection. Finally, microscopic examination and image analysis were conducted.

### Detection of oxidative stress indicators using biochemical kits

**Sample Preparation:** Myocardial tissue was mixed with PBS buffer at ratios of 1:10 (for MDA detection) and 1:9 (for SOD detection), homogenized, and centrifuged at 10,000 g for 15 min at 4 °C. The supernatant was collected for subsequent analysis.

**Reagent Preparation:** MDA detection working solution (including probe working solution and standard) and SOD detection working solution (including enzyme reaction solution) were prepared according to the kit instructions.

**MDA Measurement:** The sample was mixed with the detection working solution, incubated at 95 °C for 40 min, cooled on ice for 5 min, and centrifuged at 10,000 g for 10 min. The supernatant was measured at 532 nm absorbance.

### Calculation formula:


$$\begin{array}{c} \text{MDA }(\mu \text{mol/gprot})=[({\text{A}}_{\text{sample}}-\text{A\{blank}\})/({\text{A}}_{\text{standard}}-\text{A\{blank}\})]\times \text{C\{standard}\}\times \text{dilution factor / protein concentration} \end{array}$$

**SOD Measurement:** 20 uL of sample was added, incubated at 37 °C for 20 min, and measured at 450 nm absorbance.

### Calculation formulas:


$$\begin{array}{c} \text{SOD inhibition rate }(\%)=[(\Delta {\text{A}}_{\text{control}}-\Delta {\text{A}}_{\text{sample}})/\Delta {\text{A}}_{\text{control}}]\times 100\% \end{array}$$

$$\begin{array}{c} \text{SOD activity (U/mg prot)}=\text{Inhibition rate}/(100\%-\text{Inhibition rate}\times 12/\text{protein concentration} \end{array}$$

*(Note: In the formulas, C_standard_ is the standard concentration, ΔA is the absorbance difference, and 12 is the conversion factor for reaction volume/sample volume.)*

### Immunofluorescence staining protocol for mouse cardiac tissue

Initially, tissues fixed with 4% paraformaldehyde were processed through dehydration, embedding, and sectioning, followed by dewaxing the paraffin sections to water. Antigen retrieval was then performed by incubating the sections in preheated 95°C sodium citrate buffer (pH 6.0) under high-pressure heating for 10 minutes. After natural cooling, the sections were washed three times with phosphate-buffered saline (PBS), 3 minutes each. Excess moisture was removed using absorbent paper, and the tissue areas were circled with an immunohistochemical pen before applying diluted normal goat serum for 30-minute blocking at room temperature. After blocking, excess liquid was removed (without washing), and diluted primary antibody was directly applied for overnight incubation at 4 °C in a humidified chamber. The following day, sections were thoroughly washed with PBS (≥3 times, 3 minutes each), dried, and treated with diluted fluorescent secondary antibody for 1-hour incubation at 25 °C in a humidified chamber, followed by four additional PBS washes (3 minutes each). For nuclear staining, DAPI was applied under light-protected conditions for 5 minutes, followed by four PBS washes (5 minutes each) to remove excess dye. Finally, sections were dried and mounted with anti-fade mounting medium for observation and image acquisition under a fluorescence microscope.

### Western blot experimental procedure

Protein extraction was first performed by mincing tissue samples in a 2mL homogenizer, followed by adding 400 uL lysis buffer containing PMSF for ice-cold homogenization with repeated processing at 5-minute intervals until complete tissue disruption was achieved. After 30 minutes of lysis, the lysate was centrifuged at 12,000 rpm for 5 minutes at 4 °C, and the supernatant was aliquoted and stored at −20 °C. Protein concentration was determined using the BCA method: samples were diluted 1:19 with PBS, and a standard curve (0.2–1 mg/mL BSA) was prepared and loaded onto a 96-well plate alongside samples (standards in duplicate, samples in triplicate). After adding BCA working solution, the plate was incubated at 37°C for 30 minutes protected from light, and absorbance at 568 nm was measured to calculate protein concentration. Protein samples were mixed with 5 × loading buffer and denatured by boiling for 10 minutes before storage.

For electrophoresis, separating and stacking gels were prepared, and 40 μg total protein was loaded per well. Initial electrophoresis was performed at 80V until samples entered the separating gel, then switched to 120V for approximately 1.5 hours until the bromophenol blue reached the gel bottom. Prior to transfer, PVDF membranes were cut and activated with methanol, and the transfer sandwich was assembled in the order of “negative plate-fiber pad-filter paper-gel-membrane-filter paper-fiber pad-positive plate” for constant current transfer. After transfer, membranes were blocked with 5% skim milk in TBST (2% BSA for phosphoproteins) at room temperature for 2 hours.

Following overnight primary antibody incubation at 4 °C, membranes were washed 6 times with TBST, then incubated with HRP-conjugated secondary antibody at 37 °C for 2 hours, followed by 5 additional TBST washes. For ECL detection, enhanced solution and peroxidase solution were mixed in equal proportions and applied to membranes. After several minutes of reaction, X-ray films were exposed and processed through development and fixation to obtain results. Throughout the procedure, care was taken to maintain samples at low temperatures, ensure bubble-free transfer, and keep membranes moist during antibody incubations.

### Statistical analysis

All quantitative data were expressed as mean ± standard deviation (X ± SD). The comparison between the two groups was evaluated using the t-test. *P* value <0.05 indicated statistically significant differences. The data were analyzed using GraphPad Prism 10.0 software.

## 3. Resuls

### 3.1. Establishment and Validation of the Isoproterenol-Induced Heart Failure Mouse Model

**Fig 1:**
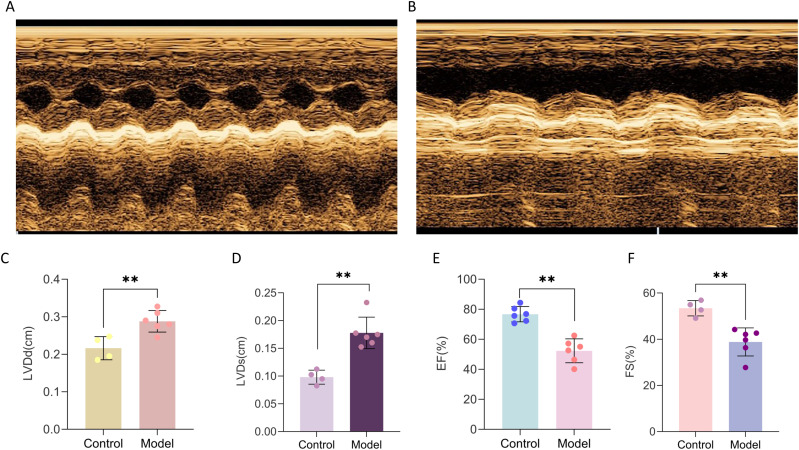
Isoproterenol-induced Decrease in Cardiac Function in Mice. A: Echocardiogram of the control group mice; B: Echocardiogram of the model group mice; C-F: Left ventricular diameter (LVDd), left ventricular diameter at systole (LVDs), ejection fraction (EF), and fractional shortening (FS) of the left ventricle in mice; n ≥ 4; Compared to the control group, **P < 0.01.

Echocardiographic evaluation revealed notable alterations in cardiac parameters across the two experimental groups (Fig 1A-1B). Mice in the model group displayed significant increases in left ventricular end-diastolic diameter (LVEDd) and left ventricular end-systolic diameter (LVESd) compared to the control groups (Fig 1C-1D) (*P* < 0.01). Conversely, ejection fraction (EF) and fractional shortening (FS) were markedly reduced in the model group (Fig 1E-1F) (*P* < 0.01). These findings underscore the effectiveness of the isoproterenol-induced myocardial hypertrophy model in inducing structural and functional changes in the heart.

**Fig 2:**
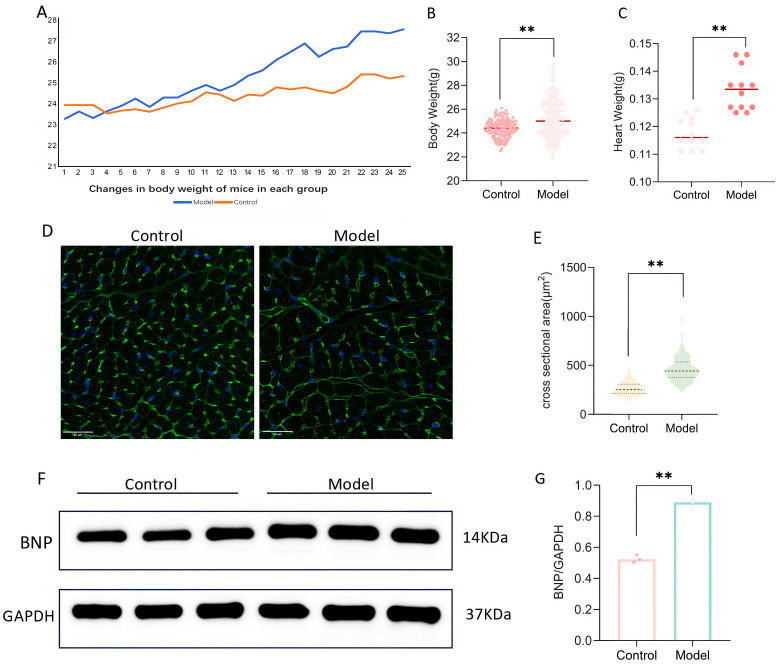
Isoproterenol-induced heart failure in mice. A: Graph of changes in mouse body weight; B-C: Body and cardiac weights of the two groups of mice; n = 12; D-E: Myocardial WGA staining of mice; n = 5; F-G: BNP protein expression levels of the two groups of mice; n = 3. Compared with the control group, **P < 0.01.

Assessment of cardiac weight provided additional insights into physiological changes among the experimental groups. Mice in the isoproterenol-induced heart failure model group exhibited a significant increase in body weight starting from day 13 of drug administration, compared to controls (Fig 2A). Moreover, body weight and cardiac weight significantly increased in the model group after 24 days of isoproterenol administration, indicative of myocardial hypertrophy (Fig 2B-2C) (*P* < 0.01). WGA staining demonstrated a significant increase in cardiomyocyte cross-sectional area in the model group compared to controls (Fig 2D-2E) (*P* < 0.01). By examining BNP in both groups using Western Blot, we found that BNP was significantly elevated in the model group compared to the control group (Fig 2F-2G) (*P* < 0.01). These findings highlight the capacity of isoproterenol to induce cardiac hypertrophy in mice.

**Fig 3:**
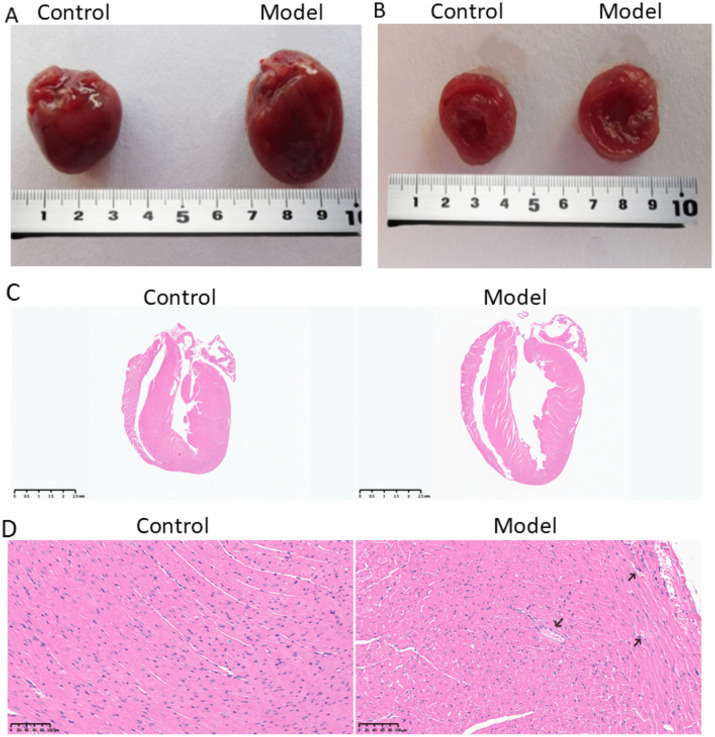
Gross view and pathological staining of mouse heart. A: Gross view of mouse heart; B: Coronary section of mouse heart; C-D: HE staining of mouse myocardial tissue; n = 5.

Morphological and pathological staining of the heart in mice revealed significant differences in myocardial structure between the two groups (Fig 3A). Compared to the control group, the model group mice exhibited a significantly larger heart volume (Fig 3A). A comparison of cross-sectional images from the hearts of the two groups showed that the ventricular cavity in the model group mice was notably enlarged (Fig 3B). HE staining results indicated that the model group mice exhibited disordered myocardial structure, vascular proliferation, and myocardial hemorrhage (Fig 3C-D). These findings collectively highlight the pathological changes in myocardial hypertrophy induced by isoproterenol in mice.

### 3.2. Downregulation of Nesprin-1 Expression in the Myocardium of Isoproterenol-Induced Heart Failure Mice

**Fig 4:**
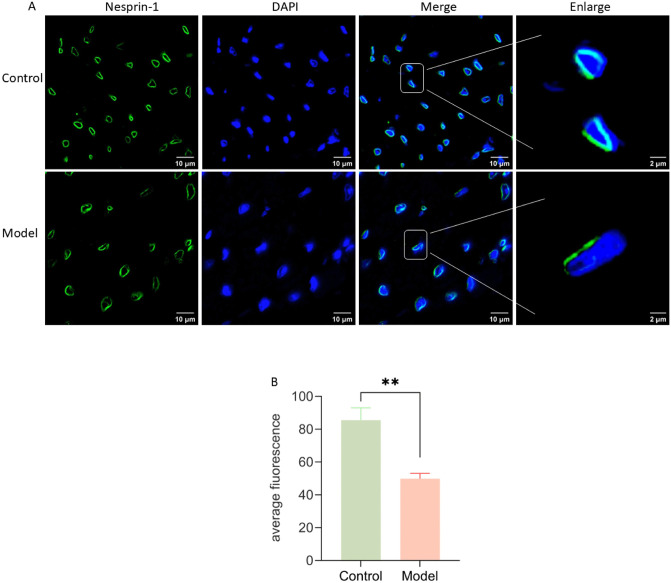
Expression changes of Nesprin-1 in myocardial tissue. A-B: Immunofluorescence staining of Nesprin in mouse myocardial tissue; n = 5; Compared with the control group, **P < 0.01.

The immunofluorescence analysis revealed a significant decrease in Nesprin-1 expression in the hearts of mice induced with isoproterenol-induced heart failure (Fig 4A) (*P* < 0.01). By fluorescent staining of Nesprin-1 with specific antibodies, we observed that Nesprin-1 was mainly located at the nuclear membrane of cardiomyocytes (Fig 4A). This localization is consistent with its function in maintaining nuclear membrane structure and nuclear-cell skeleton connection.

### 3.3. Disruption of Nuclear Homeostasis in Heart Failure Mice with Reduced Nesprin-1 Expression

**Fig 5:**
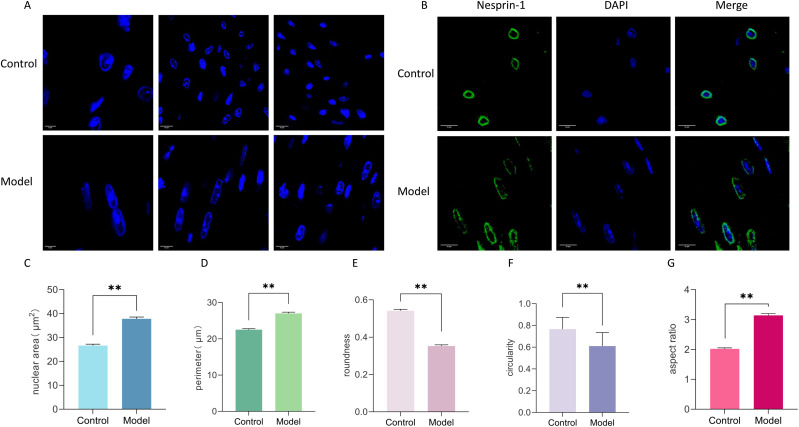
Nuclear Homeostasis Disruption in Heart Failure Mice. A: Changes in nuclear morphology; B: Nesprin-1 immunofluorescence staining; C-G: Nuclear area, nuclear perimeter, nuclear roundness, and aspect ratio of the two groups of mice; n = 5; Compared to the control group, **P < 0.01.

In our experimental study, we employed immunofluorescence to investigate the effects of Nesprin-1 expression changes on nuclear homeostasis in cardiomyocytes. By randomly selecting 40 fluorescently stained pathological sections from five model groups, we obtained 40 tissue micrographs. Ten nuclei were randomly selected from each image and analyzed using ImageJ software for morphological parameters including nuclear area, perimeter, and aspect ratio. Results demonstrated that compared with the control group, the nuclear morphology of cardiomyocytes in the model group exhibited significant abnormalities, characterized by marked increases in nuclear area, perimeter, and aspect ratio, along with a notable reduction in nuclear roundness (Fig 5A) (*P* < 0.01). Furthermore, immunofluorescence staining revealed partial or complete absence of Nesprin-1 on the nuclear membrane in some cardiomyocytes of the model group (Fig 5B) (*P* < 0.01). These results indicate that dysregulated expression of Nesprin-1 may contribute to cardiac homeostasis abnormalities in heart failure mice, suggesting a potential causal relationship between the two, though the exact mechanism requires further investigation.

### 3.4. Exacerbation of Oxidative Stress in the Nesprin-1 Downregulated Heart Failure Model

**Fig 6:**
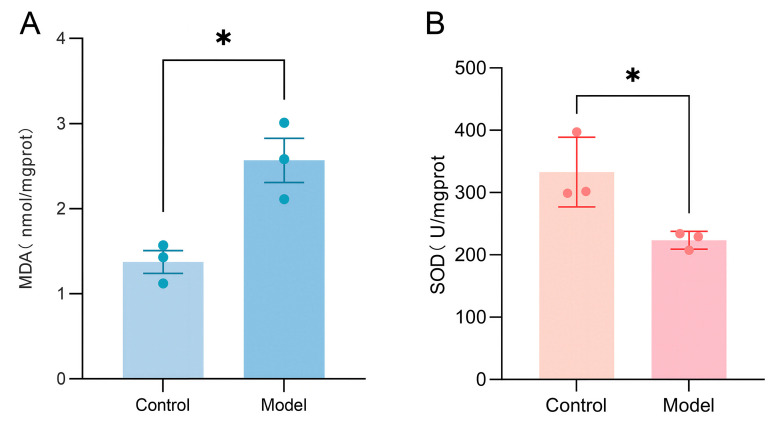
Oxidative stress aggravation in heart failure mice. A-B: Quantitative analysis of MDA and SOD in mouse myocardium; MDA: malondialdehyde; SOD: superoxide dismutase; n = 3; compared with the control group, *P < 0.05.

Oxidative stress, characterized by an imbalance between oxidizing agents and antioxidant defenses, predisposes tissues to oxidation. This imbalance fuels neutrophilic inflammation, upregulates protease secretion, and fosters the generation of various oxidative intermediates. Acknowledged as a detrimental factor contributing to aging and various diseases, oxidative stress primarily stems from the overproduction of free radicals within the body. In line with this, Nesprin-1 depletion in Drosophila melanogaster has been correlated with mitochondrial dysfunction, thereby inducing oxidative stress [31]. To investigate this phenomenon, we evaluated the expression levels of malondialdehyde (MDA) and superoxide dismutase (SOD) in cardiac tissue. Notably, compared to the control groups, the model group exhibited heightened MDA activity and diminished SOD content (Fig 6A-6B) (*P* < 0.05). These results indicate that oxidative stress levels were significantly increased and antioxidant capacity was inhibited in the Nesprin-1 dysregulated HF model.

### 3.5. The expression of Cx43 was downregulated in the myocardium of mice with dysregulation of Nesprin-1 expression in heart failure

**Fig 7:**
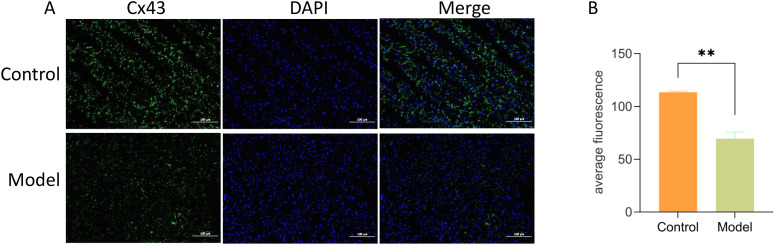
Expression of Cx43 in both groups of mice. A: Cx43 expression was detected by immunofluorescence; B Quantitative analysis of the Cx43; n = 5; compared with the control group, **P < 0.01.

Conduction dysfunction is a hallmark feature observed in the isoproterenol-induced heart failure mouse model. Cx43, primarily localized at the intercalated discs within mature cardiac myocytes, plays a pivotal role in facilitating electrical conduction among myocytes and enabling chemical signaling and energy substrate exchange, essential for coordinating cellular function [32]. Through immunofluorescence analysis, we investigated the expression levels of Cx43 in mice from each experimental group. Our results demonstrate a significant reduction in Cx43 expression specifically within the model group (Fig 7A-7B) (*P* < 0.01). In addition, combined with our previous studies, this result will provide a reliable experimental basis for our further study on how the reduction of Nesprin-1 expression affects the localization and function of Cx43 and jointly participates in the occurrence of conduction dysfunction in ISO-induced HF model.

### 3.6. Exacerbation of Myocardial DNA Damage in Heart Failure Mice

**Fig 8:**
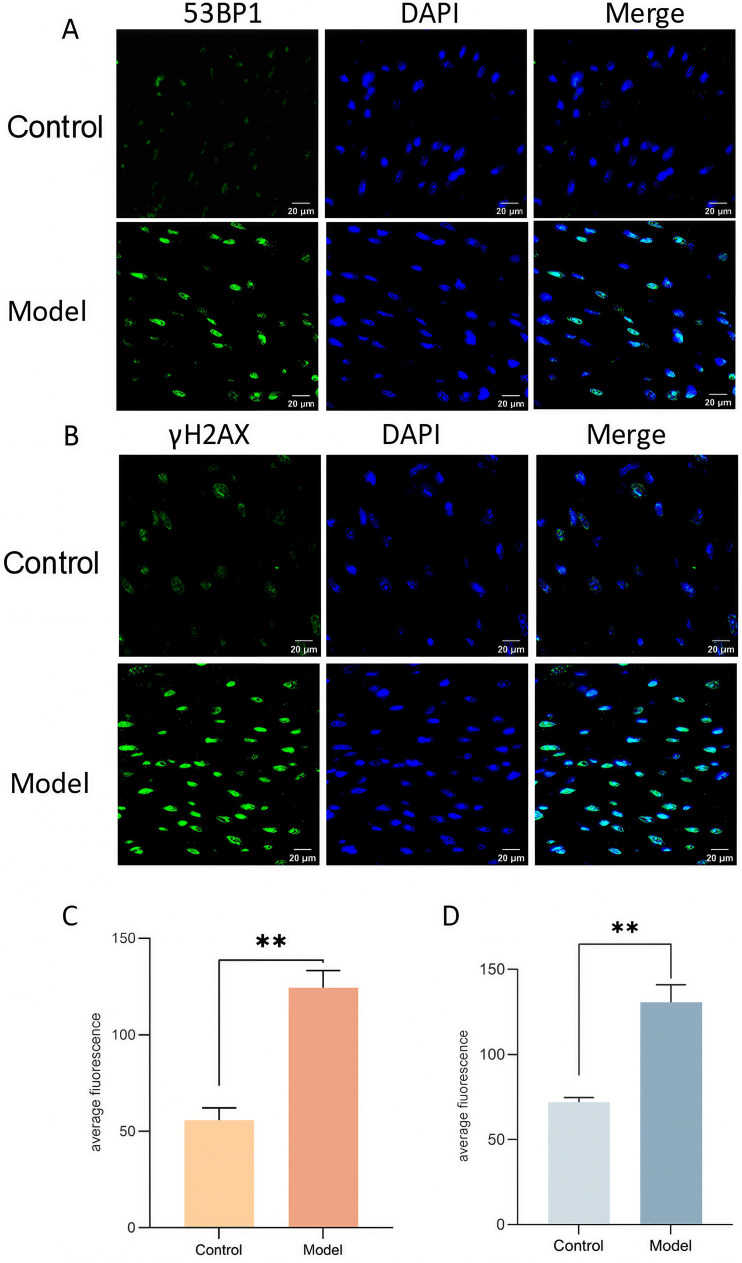
Increased DNA damage in myocardium of mice with heart failure. A-D: Detection of expression of 53 BP1 and γH2AX by immunofluorescence; 53 BP1: P53 binding protein 1; γH2AX: phosphorylated histone; n = 5; compared with the control group, **P < 0.01.

**Fig 9:**
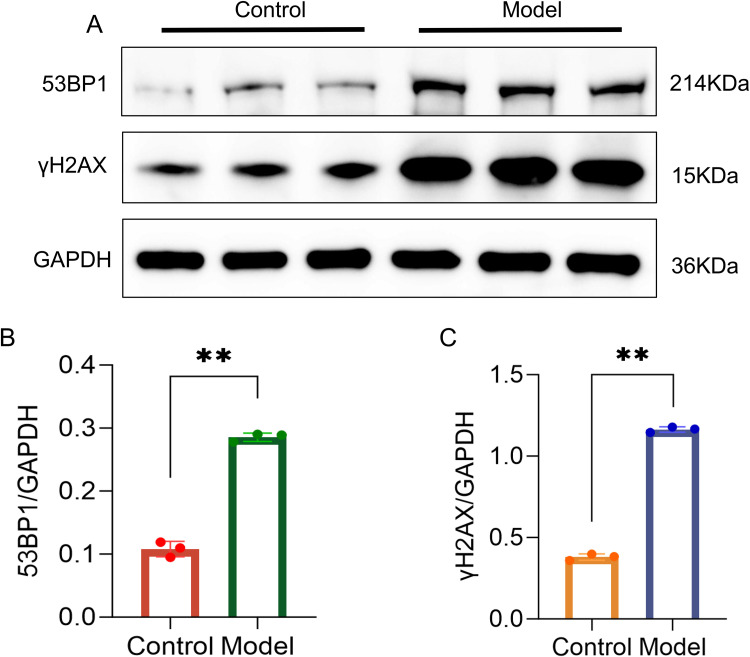
Increased DNA damage in myocardium of mice with heart failure. A-C: Protein quantitative analysis of 53 BP1 and γH2AX; 53 BP1: P53 binding protein 1; γH2AX: phosphorylated histone; n = 3; compared with the control group, **P < 0.01.

In the mouse model of heart failure induced by isoproterenol, the activation of cell DNA damage was observed when Nesprin-1 expression was reduced. Nesprin plays a crucial role in maintaining genomic integrity, and its reduction may lead to impaired DNA repair mechanisms [33]. To investigate this phenomenon, immunofluorescence and Western blot analyses were conducted to assess the expression levels of 53 BP1 and γH2AX in cardiac tissue. Our results reveal an increased expression of 53 BP1 and γH2AX in the model group compared to the control groups (Fig 8A-8D) (*P* < 0.01). In agreement with this, Western blot indicated elevated levels of DNA damage in Nesprin-deficient hearts (Fig 9A-9C) (*P* < 0.01). These results suggest that the decrease in Nesprin-1 expression may interact with the accumulation of DNA damage in the occurrence of HF.

### 3.7. Abnormal Histone Methylation in the Myocardial Tissue of Heart Failure Mice

**Fig 10:**
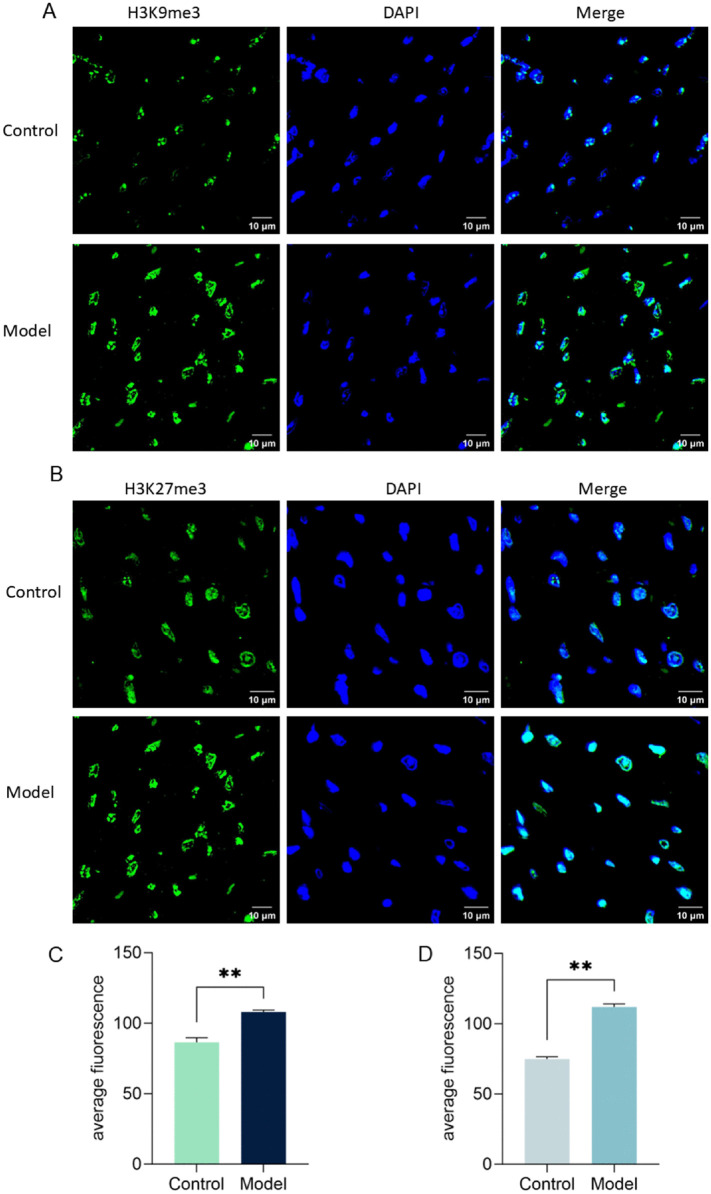
Changes in chromatin recombination in the myocardium of heart failure mice. A-D: Immunofluorescence detection of H3K9me3 and H3K27me3 expression; H3K9me3: trimethylation of histone H3 lysine 9; H3K27me3: trimethylation of histone H3 lysine 27; n = 5; compared to the control group, **P < 0.01.

**Fig 11:**
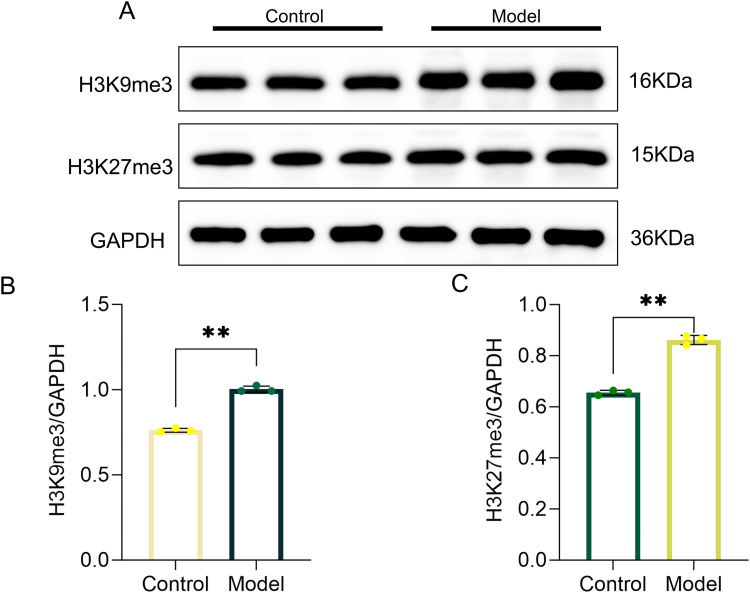
Changes in chromatin recombination in the myocardium of heart failure mice. A-C: Quantitative analysis of H3K9me3 and H3K27me3 proteins; H3K9me3: trimethylation of lysine 9 on histone H3; H3K27me3: trimethylation of lysine 27 on histone H3; n = 3; compared with the control group,**P < 0.01.

Histone methylation represents a critical modification that alters chromosome structure. Recent studies have highlighted the pivotal role of histone methylation modifications in myocardial hypertrophy, fibrosis, and cardiac function [34,35]. Through immunofluorescence and Western blot analysis, we assessed the expression levels of H3K9me3 and H3K27me3 in mice from each experimental group. As depicted in the figures, the model group exhibited significantly increase expression levels of H3K9me3 and H3K27me3 compared to the control groups (Fig 10A-10D) (*P* < 0.01). This result is consistent with the Western blot results (Fig 11A-11C) (*P* < 0.01). These findings suggest that the histone methylation pattern disorder in the isoproterenol induced heart failure mouse model may be associated with the decreased level of Nesprin-1.

### 3.8. Activation of the ERK Signaling Pathway in the Heart Failure Mouse Model

**Fig 12:**
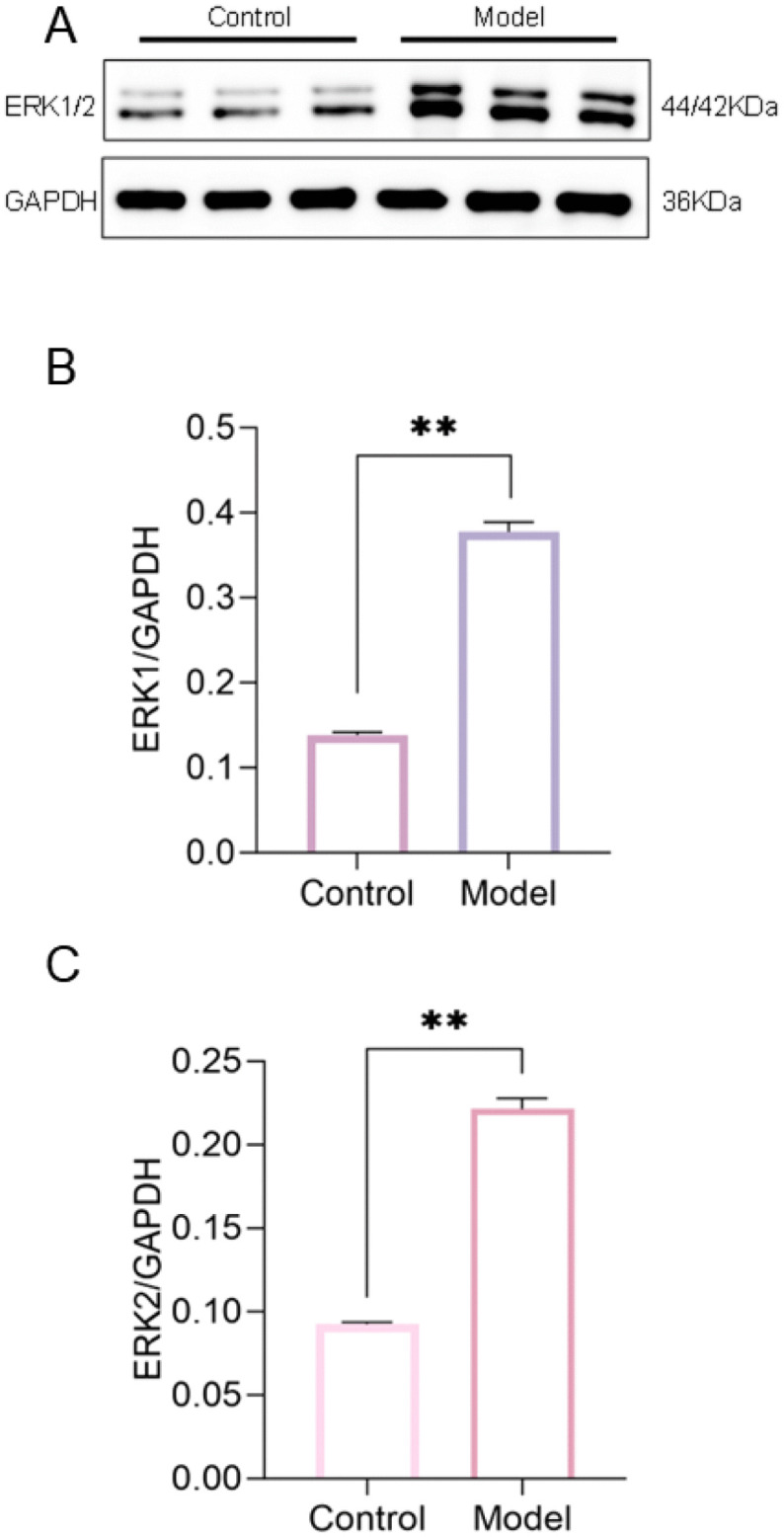
Activation of ERK1/2 expression in myocardium of mice with heart failure. A-C: Detection of ERK1/2 expression by protein immunoblotting; ERK: extracellular regulated protein kinase; n = 3; compared with the control group, **P < 0.01.

The ERK pathway is recognized as a pivotal regulator in the development of myocardial hypertrophy. Studies have elucidated the formation of Nesprin-2/ERK complexes at sites of DNA damage in aortic smooth muscle cells, indicating a potential role of Nesprin proteins in modulating ERK signaling [36]. To investigate this phenomenon in the context of heart failure, we employed Western blot analysis to evaluate the expression levels of ERK in cardiac tissue samples from mice in each experimental group. Strikingly, our results revealed a notable increase in ERK expression in the model group compared to the control group, indicative of the activation of the ERK pathway in the cardiac tissue of the isoproterenol-induced heart failure mouse model (Fig 12A-12C) (*P* < 0.01). Combined with the reduced expression of Nesprin-1 in iprindel induced heart failure mice, we speculate that the reduced expression of Nesprin-1 may be associated with the activation of ERK pathway.

## Discussion

Heart failure stands as an enduring challenge within global healthcare systems, presenting a formidable burden characterized by its pervasive prevalence, grim prognostic outlook, and profound detriment to patients’ quality of life. Despite notable strides in HF management witnessed over recent decades, including the advent of neurohormonal blockade, device-based interventions, and cardiac transplantation, the persistently high rates of mortality and morbidity underscore the insufficiency of current therapeutic strategies [37–39]. Consequently, there exists an imperative mandate for sustained investigative efforts directed towards unraveling the intricate molecular mechanisms governing HF pathogenesis.

In this study, we induced myocardial hypertrophy in mice by intraperitoneal injection of ISO. Our findings indicate that the administration of ISO successfully induced myocardial hypertrophy, characterized by significant changes in heart weight, body weight, and WGA pathological staining results compared to the control group mice, consistent with previous studies [40,41]. Long-term stimulation by pathological factors can lead to persistent changes in cardiac structure, resulting in HF [42]. HE staining showed that the myocardial structure of the mice with Nesprin-1 deficiency was disordered, and the blood vessels were proliferated and the myocardial was hemorrhagic. Additionally, cardiac function tests confirmed the onset of HF in the mice, with significant changes in LVEDd, LVESd, EF, and FS. When myocardial cell volume and pressure overload occur, ventricular muscle cells release BNP, which exerts sodium-losing, diuretic, vasodilatory, and antihypertensive effects [43]. BNP is a crucial biomarker for the clinical diagnosis of HF. Western blotting detected a significant increase in BNP in the myocardial tissue of mice induced by isoproterenol, providing a more reliable basis for confirming the onset of HF in mice.

Nesprin are giant scaffolding proteins located at the NE that play essential roles in maintaining nuclear structure and regulating various cellular processes, including nuclear positioning, cytoskeletal organization, and gene expression [27,44–48]. While previous research has primarily focused on the role of Nesprin in skeletal and muscular diseases, their involvement in cardiac pathology, particularly HF, remains poorly understood. In this study, we used the ISO-induced mouse heart failure model to initially assess the changes in Nesprin-1 expression and its potential links with nuclear structure, signaling pathways, and gene expression regulation. The ISO-induced HF mouse model established and validated in this study provides new insights into the pathophysiology of heart failure and the role of Nesprin-1 in cardiac function.

Our research has revealed that, compared to the control group, the expression of Nesprin-1 in the myocardium of ISO-induced HF mice is significantly reduced. This finding suggests that changes in Nesprin-1 expression may be linked to pathological remodeling processes associated with HF, such as myocardial hypertrophy and dysfunction. The downregulation of Nesprin-1 may be related to abnormalities in the morphology, nuclear localization, and structural integrity of myocardial cells, which have been preliminarily observed through histopathological examination. However, the specific molecular mechanisms still require further investigation. Additionally, the dysregulation of Nesprin-1 expression may affect nuclear stability by disrupting the integrity of the nuclear membrane, potentially leading to cellular dysfunction. These findings suggest that, as a key component of the nuclear scaffold and cytoskeletal connection complex, changes in Nesprin-1 expression may be associated with nuclear instability and abnormal cell signaling.

The exact mechanisms underlying Nesprin-1-mediated pathogenesis in HF remain incompletely understood, but our study has shed light on several potential avenues for further investigation. Our findings elucidate the multifaceted role of Nesprin-1 in HF pathogenesis, encompassing oxidative stress, electrical conduction abnormalities, and epigenetic dysregulation. First, Nesprin-1 may affect cardiac morphology through interactions with cytoskeletal elements and extracellular matrix proteins, thereby regulating the signaling pathways involved in fibroblast activation. However, the causal relationship between Nesprin-1 and these processes has not been directly demonstrated. For example, further research is needed to determine how Nesprin-1 affects fibroblast activation by influencing cardiac morphology through interactions with cytoskeletal components and extracellular matrix proteins.

Additionally, in the ISO-induced heart failure model, reduced Nesprin expression was observed to be associated with enhanced oxidative stress, manifested as alterations in oxidative stress markers (e.g., elevated malondialdehyde levels and decreased superoxide dismutase content). Concurrently, increased expression of DNA damage repair proteins (such as γH2AX and 53 BP1) in this model further suggests that Nesprin may play a critical role in maintaining genomic integrity and protecting against oxidative stress-induced cardiac injury. Furthermore, the observed reduction in connexin 43 expression in Nesprin-deficient hearts highlights Nesprin’s involvement in regulating cardiac conduction and electromechanical coupling. Diminished Cx43 expression may contribute to conduction abnormalities and arrhythmias commonly observed in HF, emphasizing Nesprin’s role in regulating both structural and functional aspects of cardiac tissue. Lastly, alterations in histone methylation patterns, specifically changes in H3K9me3 and H3K27me3 levels in Nesprin-deficient hearts, suggest dysregulated epigenetic regulation in HF pathogenesis. These findings underscore the intricate interplay between Nesprin and epigenetic regulatory mechanisms in maintaining cardiac homeostasis.

Activation of the ERK pathway has been implicated in various hypertrophic stimuli and is known to regulate gene expression, protein synthesis, and cell survival in cardiomyocytes [49–51]. Our study elucidates the involvement of the ERK signaling pathway may be involved in mediating Nesprin-1-induced cardiac hypertrophy and remodeling in response to isoproterenol stimulation. Activation of the ERK pathway has been implicated in various cellular processes, including cell proliferation, differentiation, and survival [52–54]. Here, we demonstrate a significant increase in ERK expression in the cardiac tissue of mice subjected to isoproterenol-induced heart failure, suggesting the activation of the ERK pathway in response to cardiac stress. The observed upregulation of ERK signaling in Nesprin-deficient hearts highlights a potential interaction between Nesprin and the ERK pathway in regulating cardiac function. Previous studies have indicated the formation of Nesprin-2/ERK complexes at sites of DNA damage, implicating Nesprin proteins in the modulation of ERK signaling in other cellular contexts [36]. Our findings extend this understanding to the cardiac setting, where Nesprin deficiency may contribute to ERK pathway activation, thereby exacerbating myocardial injury and dysfunction. The activation of the ERK pathway in response to cardiac stressors, such as isoproterenol administration, suggests a role of ERK signaling in mediating pathological remodeling and maladaptive responses in the heart. Future studies aimed at elucidating the precise mechanisms underlying the crosstalk between Nesprin and the ERK pathway may provide further insights into the molecular mechanisms of heart failure pathogenesis.

The identification of Nesprin-1 as a potential regulator of HF pathogenesis opens up new avenues for therapeutic intervention. Targeting Nesprin-1 and its associated signaling pathways may offer novel strategies for preventing or attenuating adverse cardiac remodeling and dysfunction in HF [55–58]. Small molecule inhibitors, gene therapy approaches, or gene editing techniques could be explored to modulate Nesprin-1 expression or activity selectively in the heart. Additionally, our study underscores the importance of further research into the molecular mechanisms governing nuclear envelope dynamics and their contribution to cardiovascular disease. In conclusion, our study provides compelling evidence implicating Nesprin-1 in the pathogenesis of HF induced by isoproterenol. By elucidating the role of Nesprin-1 in cardiac remodeling and electrical conduction, we have shed light on novel molecular mechanisms underlying HF pathophysiology. Further investigation into the functional significance of Nesprin-1 and its interactions with key signaling pathways may yield valuable insights into disease progression and facilitate the development of targeted therapies for HF. Ultimately, our findings contribute to a deeper understanding of the complex molecular networks driving HF and offer hope for improved outcomes for patients afflicted with this devastating condition. This comprehensive discussion highlights the significance of our findings and provides a roadmap for future research endeavors aimed at unraveling the intricate molecular mechanisms of HF pathogenesis and identifying innovative therapeutic strategies to combat this disease.

However, this study has several limitations. First, our findings regarding the association between decreased Nesprin-1 expression and various pathological features—such as myocardial structural disorder, oxidative stress, DNA damage, and epigenetic alterations—remain observational and correlational. Due to the current lack of specific Nesprin-1 inhibitors and the methodological challenges associated with generating Nesprin-1 knockout models, we could not establish a direct causal relationship or elucidate the precise downstream molecular mechanisms, including its exact interaction with the ERK signaling pathway. Second, our study relied exclusively on an isoproterenol (ISO)-induced HF mouse model. Future studies incorporating diverse HF models, such as transverse aortic constriction (TAC) or myocardial infarction (MI), are necessary to validate the generalizability of these findings. In future research, we aim to employ Nesprin-1-specific knockout models combined with multi-omics analyses to comprehensively decipher its exact functional role and mechanistic pathways in HF pathogenesis

## Supporting information

S1 Fig Original uncropped western blot images. (ZIP)

S1 Table Raw data of mouse body weight. (XLSX)
