# Supplementary material for: Exploring the Role of Nesprin1 in isoproterenol-induced heart failure: Insights into pathogenesis and therapeutic implications
Source: PLoS One. 2026 Aug 11;21(8):e0354482. doi: 10.1371/journal.pone.0354482 (PMC13460607; doi:10.1371/journal.pone.0354482)
Supplement: Supplementary file 1 [file pone.0354482.s003.pdf]

Abstract:

1. BNP should be spelled out as the term was stated the first time in the article
2. CX43 and Nesprin 1 expression were both determined using immunofluorescence analysis, hence can be stated in one sentence.
3. BNP, CX43 and Nesprin 1 should be briefly introduced in the abstract.
4. Please state the cardiac parameters examined.
5. Any gene manipulation done before the conclusion made by stating that "This study elucidates the critical role of Nesprin1 in isoproterenol-induced……" ?

Introduction

The background and rationale were sufficiently addressed

Materials and Methods:

General comments: Technical details are not sufficient

Drugs and Chemicals

Catalog no. of the chemicals/products used were not stated

Establishment of Isoproterenol-Induced Heart Failure Model in Mice

Sex and Age of the animals were not stated

Cardiac Function Assessment

Specific name of the statistical test was not stated

Measurement of Body and Heart Weight

Clarify the timing of which the mice were anesthetized

The Biochemical Kits Detects The Oxidative Stress Indicators

Please simplify the demonstration of protein concentration calculation

Immunofluorescence Staining Protocol for Mouse Cardiac Tissue

The model of the microscope and the software were not stated

Western Blot Experimental Procedure

1. The catalog no and the company which RIPA buffer, phosphate and protease inhibitors were purchased from should be stated
2. Details of gel documentation system and densitometry analysis software should be stated

Results:

1. Sample size determination (animal numbers) was not demonstrated/explained
2. Western blot in Figure 2 was not labelled.
3. Specific statistical test for each assay was not stated clearly
4. Importantly knockdown of Nesprin 1 was not demonstrated and hence the data doesn't support the stated results

Discussion:

The data provided doesn't support the discussion as knockdown model of Nesprin 1 was not used

Declarations:

Ethics approval and consent to participate

Animal ethics approval no. was not stated in the main text

Availability of Data and Material:

While original western blots were provided, other minimal data sets were not included in the supplement information sheet
