# Supplementary material for: Exploring the Role of Nesprin1 in isoproterenol-induced heart failure: Insights into pathogenesis and therapeutic implications
Source: PLoS One. 2026 Aug 11;21(8):e0354482. doi: 10.1371/journal.pone.0354482 (PMC13460607; doi:10.1371/journal.pone.0354482)
Supplement: Supplementary file 2 [file pone.0354482.s004.pdf]

PLOS ONE

Exploring the Role of Nesprin1 in Isoproterenol-Induced Heart Failure Insights into Pathogenesis and Therapeutic Implications

--Manuscript Draft--

**Manuscript Number:**	PONE-D-25-08527R1
**Article Type:**	Research Article
**Full Title:**	Exploring the Role of Nesprin1 in Isoproterenol-Induced Heart Failure Insights into Pathogenesis and Therapeutic Implications
**Short Title:**	Nesprin and Heart Failure
**Corresponding Author:**	Lin Teng<br>Yichang Central People's Hospital<br>yichang, CHINA
**Keywords:**	
**Abstract:**	Objective: This study investigates the role of Nesprin1 in isoproterenol-induced heart failure and its implications for cardiac pathogenesis mechanism.<br>Methods: A mouse model of isoproterenol-induced heart failure was established and validated through echocardiographic evaluation, gross view , histopathological examination, assessment of cardiac parameters and Western Blot for BNP expression. Nesprin1 expression in cardiac tissue was examined via immunofluorescence staining, while cardiac tissue sections underwent staining to assess myocardial structure, cellular morphology. Quantification of malondialdehyde (MDA) and superoxide dismutase (SOD) levels was conducted to evaluate oxidative stress. Cx43 expression was measured using immunofluorescence. DNA damage and histone methylation were determined by examining the expression of 53BP1, γH2AX, H3K9me3 and H3K27me3, respectively, by immunofluorescence and Western blot. Additionally, Western blot analysis was performed to investigate the potential role of ERK in Nesprin1-mediated heart failure pathogenesis.<br>Results: Mice with isoproterenol-induced heart failure displayed significant alterations in cardiac parameters, accompanied by a notable reduction in Nesprin1 expression detected via immunofluorescence analysis. Moreover, the cardiac tissue of the model group exhibited abnormal nuclear morphology. Elevated MDA activity and decreased SOD content indicated heightened oxidative stress in the cardiac tissue of mice with heart failure. Furthermore, reduced Nesprin expression correlated with decreased Cx43, suggesting potential conduction abnormalities. Additionally, increased expression of γH2AX and 53BP1, markers of DNA damage, suggested activation of cellular DNA damage pathways. Dysregulated histone methylation patterns, as indicated by increase levels of H3K9me3 and H3K27me3, hinted at altered epigenetic regulation. Finally, a significant increase in ERK expression implied activation of the ERK pathway, potentially contributing to myocardial injury.<br>Conclusions: This study elucidates the critical role of Nesprin1 in isoproterenol-induced heart failure pathogenesis. Reduced Nesprin1 expression contributes to cardiac dysfunction and remodeling in isoproterenol-induced heart failure through mechanisms involving oxidative stress, DNA damage response, conduction abnormalities and epigenetic regulation.
**Order of Authors:**	琴 覃
	Dong cengyuan
	Zi-yi Zhou
	Liu Yang yuan zhi
	Chunyu Cao
	Lin Teng
**Opposed Reviewers:**	
**Response to Reviewers:**	Dear Editor,<br>We sincerely thank you and the reviewers for valuable comments and the support

Powered by Editorial Manager® and ProduXion Manager® from Aries Systems Corporation

extended to our manuscript. We deeply appreciate your dedication and professionalism throughout this process. In accordance with the guidelines, we have provided detailed responses to the reviewers' comments point by point below(Changes are marked in red in the manuscript).

Reviewer #1
Minor comment:
Comment 1: Throughout the manuscript:The English is understandable, but not highly polished. Many sentences are wordy and awkwardly structured, and some terms are misused. A professional language review is needed to improve fluency and scientific clarity.Exemple L221. The authors wrote: "The ross and pathological examination". did they mean "The gross pathological examination"?Many other places.Exemple L364 “significantly reduction” should be corrected to “significant reduction.” A careful proofreading of the section for grammatical accuracy is recommended.
Response 1:Thanks for your constructive feedback on our manuscript. We appreciate your careful assessment of the grammar and language issues. Each sentence has been carefully checked and revised to improve readability.The red indicates the modified text.

Comment 2: In the result presentation, the authors mention "significant" differences. These sentences should explicitly state the precise p-values and statistical tests.
Response 2:Thank you very much for your valuable suggestions. We have made changes to the original text according to your suggestions.

Comment 3: Overall, and specifically in the discussion section:Some sentences are complex and could benefit from restructuring to enhance readability. For example, the transition between the introduction of Nesprin1 and its connection to HF pathogenesis could be smoother.
Response 3: We feel great thanks for your professional review work on our article.We have made corresponding modifications to the discussion section according to your suggestions. You can see the specific modifications in the original text.

Comment 4: Ensure that the argument follows a logical progression, from the established burden of HF to the novel contributions of this study, in the discussion section.
Response 4:We sincerely thank you for your careful reading.In the discussion section, we first outlined the significant risks of heart failure and the necessity of conducting research on it. We then confirmed the successful construction of the heart failure mouse model by analyzing the basic physiological parameters, pathological staining, and cardiac color Doppler ultrasound results in mice. Next, by analyzing various pathological manifestations in heart failure mice, we explored potential directions for the role of Nesprin-1 in the disease process. Finally, we summarized the innovations and limitations of this study.

Major comments:
Comment 1: The authors suggest that Nesprin1 downregulation is causative, but this is not experimentally proven, unless I did not read correctly (authors did not use genetic knockdown or RNAi), wherease, the findings are correlational (i.e., Nesprin1 is reduced in heart failure). Would suggest the authors to clarify this limitation.
Response 1:Thank you for your valuable and practical suggestions. As you understand, this study has confirmed a series of pathological phenomena in heart failure mice with reduced Nesprin1 levels, which may be associated with the downregulation of Nesprin-1. However, due to certain objective reasons, this conclusion has not been experimentally verified. This limitation is addressed in the revised discussion. We will use additional techniques in future studies to further investigate this area.

Comment 2: The Authors suggest that reduced Nesprin1 leads to ERK activation. Would you please elaborate with more mechanistic evidence? I understand the discussion highlights the significance of Nesprin1, however, it would be beneficial to include a comparison with other known mechanisms of HF progression. More explicit connections between previous research and the current findings could strengthen the justification for Nesprin1’s role.I would suggest the authors to add more reference to prior studies when applicable) regarding the discussion on Nesprin1’s involvement in

Powered by Editorial Manager® and ProduXion Manager® from Aries Systems Corporation

oxidative stress, nuclear homeostasis, and the ERK pathway.
Response 2: Sincerely thank you for your insights into the paper. I understand the rationality of your suggestion to add a comparison of Nesprin1 with other mechanisms of heart failure and a more detailed explanation of the mechanism. After careful discussion by the team, we believe that this study is the first exploratory work to reveal the expression changes of Nesprin1 in ISO-induced heart failure models and its association with nuclear homeostasis imbalance and oxidative stress. Maintaining the existing discussion framework will better highlight the original value of this new finding.Regarding the molecular mechanism of Nesprin1 down-regulation and ERK activation, this is indeed a question of great scientific value, but considering that this study mainly focuses on establishing phenotypic associations, detailed mechanism analysis will be the focus of our follow-up research using the gene knockout animal model we are building. Your valuable suggestions have pointed to important follow-up research directions for us, including systematic comparison of the relationship between Nesprin1 and other heart failure pathways, and in-depth analysis of the molecular mechanisms of its regulation of ERK pathways.
We earnestly hope that you will understand the limitations of the current research phase, and we commit to further exploring these key scientific questions in future work.

Comment 3: While increased MDA and decreased SOD suggest oxidative stress, the study does not establish whether oxidative stress is the cause or consequence of Nesprin1 downregulation.
Response 3:I sincerely appreciate this insightful comment. My data demonstrate that ISO-induced heart failure mice with Nesprin-1 downregulation exhibit increased MDA and decreased SOD activity, indicating enhanced oxidative stress. However, as the reviewer astutely noted, the current study cannot definitively determine whether oxidative stress drives or results from Nesprin-1 suppression.
I recognize that Nesprin-1's role in cardiac pathology remains underexplored, with limited existing literature. Nevertheless, given heart failure's clinical significance and prior evidence of Nesprin-1's functional importance in other disease contexts, along with its myocardial-specific isoform expression patterns, this study was designed to investigate three key aspects: Nesprin-1's expression profile in ISO-induced HF, its potential functional contributions to disease progression, and possible mechanistic pathways.
These findings establish an initial association between Nesprin-1 dysregulation and oxidative stress in HF, creating a foundation for future mechanistic studies. I am currently generating Nesprin-1 knockout mice to specifically examine the causal relationship between Nesprin-1 deficiency and oxidative stress, elucidate the involved molecular pathways, and characterize its precise regulatory role in HF pathogenesis. I look forward to sharing these more definitive results in future work.

Comment 4:Overall, the study is scientifically sound but needs improved language, better statistical transparency, and stronger mechanistic evidence to fully support its conclusions.
Response 4:Thank you for your valuable comments. I have made the corresponding changes.

Comment 5:Major revisions are recommended to improve the manuscript's clarity, presentation, and interpretation of findings.
Response 5:Thank you very much for your valuable suggestions. In the original text, I have selected a clearer picture and modified the language expression and result explanation of the whole text to improve the readability of the article.

Reviewer #2:
Comment 1:Abstract:
1. BNP should be spelled out as the term was stated the first time in the article
2. CX43 and Nesprin 1 expression were both determined using immunofluorescence analysis, hence can be stated in one sentence.
3. BNP, CX43 and Nesprin 1 should be briefly introduced in the abstract.
4. Please state the cardiac parameters examined.
5. Any gene manipulation done before the conclusion made by stating that "This study elucidates the critical role of Nesprin1 in isoproterenol-induced……" ?
Response 1:Thank you very much for your suggestions and questions. Firstly, the issues with the wording in the abstract have been corrected in the original text.

Powered by Editorial Manager® and ProduXion Manager® from Aries Systems Corporation

Secondly, regarding whether gene manipulation was performed, my response is that although gene mice were constructed, this experiment did not include this part. The primary goal of this study is to provide a research foundation for further studies on the specific mechanisms of Nesprin-1 in the development of heart failure. Therefore, no gene manipulation was conducted in this study.

Comment 2:Introduction
The background and rationale were sufficiently addressed
Response 2:I sincerely thank you for your full affirmation of the background and argumentation basis in the introduction of this study. I am very glad that the scientific basis and clinical relevance of this study have been clearly and effectively expounded.

Comment 3:Materials and Methods:
General comments: Technical details are not sufficient
Response 3:Thank you for your valuable suggestions. Under your guidance, I have modified the details of the materials and methods section in the article. See the red font for the specific content.

Comment 4:Drugs and Chemicals
Catalog no. of the chemicals/products used were not stated
Response 4:Thank you for pointing this out. I have added the catalogue number of the important chemicals/products in the relevant section.

Comment 5:Establishment of Isoproterenol-Induced Heart Failure Model in Mice
Sex and Age of the animals were not stated
Response 5:Thank you for your suggestion. In line 140 of the article, it has been stated that the animals used in this study are male C57BL/6 mice with a weight of 20-22g.

Comment 6:Cardiac Function Assessment
Specific name of the statistical test was not stated
Response 6:Thank you for your suggestion. The relevant testing methods have been explained in the last part of the methodology.

Comment 7:Measurement of Body and Heart Weight
Clarify the timing of which the mice were anesthetized
Response 7:Thank you for raising this concern. In this study, mouse body weight was measured using an electronic scale while the animals were conscious, thereby avoiding the effects of repeated anesthesia. All mice were euthanized 24 hours after the final administration, followed by cardiac tissue collection and weighing. No anesthetic agents were used during any body weight measurements throughout the study.

Comment 8:The Biochemical Kits Detects The Oxidative Stress Indicators
Please simplify the demonstration of protein concentration calculation
Response 8:Thank you! I have simplified the MDA/SOD calculation formula in the original text.

Comment 9:Immunofluorescence Staining Protocol for Mouse Cardiac Tissue
The model of the microscope and the software were not stated
Response 9:Thank you for your insightful comments on the manuscript.I have updated the microscope model and software used for immunofluorescence in this article.

Comment 10:Western Blot Experimental Procedure:1. The catalog no and the company which RIPA buffer, phosphate and protease inhibitors were purchased from should be stated. 2. Details of gel documentation system and densitometry analysis software should be stated
Response 10:Thank you for your very meaningful suggestion. According to your instructions, the relevant parts have been described in the section of Materials and Methods respectively.

Comment 11:Results:1. Sample size determination (animal numbers) was not demonstrated/explained2. Western blot in Figure 2 was not labelled.3. Specific statistical test for each assay was not stated clearly4. Importantly knockdown of Nesprin 1 was not demonstrated and hence the data doesn’t support the stated

	results.Discussion:The data provided doesn’t support the discussion as knockdown model of Nesprin 1 was not used<br>Response 11:I sincerely appreciate your valuable comments on our manuscript. We have carefully examined all the issues you raised and made corresponding revisions. Regarding the sample size, we have supplemented the figure legends in the Results section to clarify that the sample size meets statistical requirements. For the oversight in labeling Figure 2 Western Blot, we have re-examined the original data and improved the labeling of experimental groups, molecular weight markers, and internal reference protein bands.<br>In terms of statistical methods, we have added clarification that two-sample t-tests were used for normally distributed data (confirmed by Shapiro-Wilk test) and Mann-Whitney U tests for non-normally distributed data. We fully understand your concern regarding the validation of Nesprin 1 knockdown efficiency. This study primarily focuses on preliminary exploration of Nesprin-1 expression changes and their pathological significance in the ISO-induced heart failure model. We are currently developing Nesprin-1 knockout mouse models to enable more systematic mechanistic studies in the future.<br>The key findings of this study include: significant downregulation of Nesprin-1 expression in the ISO-induced heart failure model, accompanied by pathological phenomena such as nuclear homeostasis imbalance and aggravated oxidative stress, which may be related to ERK signaling pathway activation. We sincerely accept your suggestions, which are invaluable for improving the completeness of this research. While this study has certain limitations, it establishes an important foundation for subsequent mechanistic investigations. We are fully prepared to provide any additional materials as needed.<br><br>Comment 12:Declarations:Ethics approval and consent to participate;Animal ethics approval no. was not stated in the main text;Availability of Data and Material:While original western blots were provided, other minimal data sets were not included in the supplement information sheet<br>Response 12: I sincerely appreciate your valuable feedback regarding ethical approval and data accessibility in this paper. Your suggestions to enhance research transparency and reproducibility are of great importance to me. Concerning the animal ethics review, I have supplemented complete information in the "Materials and Methods" section: "All mice were housed in a Specific Pathogen-Free (SPF) facility at China's Three Gorges University (Yichang, Hubei Province). The experimental animal quality certification number is 42010200009428, and the experimental animal use permit number is 00305510. All procedures complied with international standards for laboratory animal care and use." Regarding data availability, although I have provided all Western blot raw images in the supplementary materials, I recognize the importance of additional data. The foundational datasets supporting key research findings have been systematically organized and are available to corresponding authors upon request. This clarification has been incorporated into the "Data Availability Statement" section of the paper. Thank you for your attention to these critical aspects of my work, and I hope this explanation addresses your concerns. If any other supplementary materials are required, I will provide them at any time.<br>We have carefully considered all the comments and suggestions and have made the necessary revisions to improve the quality and clarity of our manuscript. We hope that the changes we've made address the concerns raised by the reviewers to your satisfaction. Simultaneously, we are also eagerly looking forward to your reply.<br>Thank you once again for your time and consideration.<br>Sincerely,<br>Teng Lin<br>Department of Cardiology/Yichang centre people's hospital<br>The First College of China Three Gorges University<br>No.183, Yiling Road, Yichang, 443003, China<br>Email:tenglin@ctgu.edu.cn
**Additional Information:**	
**Question**	**Response**
**Financial Disclosure**<br><br>Enter a financial disclosure statement that	Yes

Powered by Editorial Manager® and ProduXion Manager® from Aries Systems Corporation

describes the sources of funding for the work included in this submission. Review the submission guidelines for detailed requirements. View published research articles from *PLOS ONE* for specific examples.<br><br>This statement is required for submission and **will appear in the published article** if the submission is accepted. Please make sure it is accurate.<br><br>**Funded studies**<br>Enter a statement with the following details:<br>• Initials of the authors who received each award<br>• Grant numbers awarded to each author<br>• The full name of each funder<br>• URL of each funder website<br>• Did the sponsors or funders play any role in the study design, data collection and analysis, decision to publish, or preparation of the manuscript?<br><br>Did you receive funding for this work?	
Please add funding details.<br>as follow-up to "**Financial Disclosure**<br><br>Enter a financial disclosure statement that describes the sources of funding for the work included in this submission. Review the submission guidelines for detailed requirements. View published research articles from *PLOS ONE* for specific examples.<br><br>This statement is required for submission and **will appear in the published article** if the submission is accepted. Please make sure it is accurate.	This research was funded by the Hubei Provincial Natural Science Foundation Project (Joint Fund Project, Project No.: 2024AFD143).

Powered by Editorial Manager® and ProduXion Manager® from Aries Systems Corporation

**Funded studies**<br>Enter a statement with the following details:<br>• Initials of the authors who received each award<br>• Grant numbers awarded to each author<br>• The full name of each funder<br>• URL of each funder website<br>• Did the sponsors or funders play any role in the study design, data collection and analysis, decision to publish, or preparation of the manuscript?<br><br>Did you receive funding for this work?"	
Please select the country of your main research funder (please select carefully as in some cases this is used in fee calculation).<br>as follow-up to "**Financial Disclosure**<br><br>Enter a financial disclosure statement that describes the sources of funding for the work included in this submission. Review the submission guidelines for detailed requirements. View published research articles from *PLOS ONE* for specific examples.<br><br>This statement is required for submission and **will appear in the published article** if the submission is accepted. Please make sure it is accurate.<br><br>**Funded studies**<br>Enter a statement with the following details:<br>• Initials of the authors who received each award<br>• Grant numbers awarded to each author<br>• The full name of each funder<br>• URL of each funder website<br>• Did the sponsors or funders play any role in the study design, data collection and analysis, decision to publish, or preparation of the manuscript?<br><br>Did you receive funding for this work?"	CHINA - CN
**Competing Interests**	The authors have declared that no competing interests exist.

Use the instructions below to enter a competing interest statement for this submission. On behalf of all authors, disclose any competing interests that could be perceived to bias this work—acknowledging all financial support and any other relevant financial or non-financial competing interests.<br><br>This statement is required for submission and **will appear in the published article** if the submission is accepted. Please make sure it is accurate and that any funding sources listed in your Funding Information later in the submission form are also declared in your Financial Disclosure statement.<br><br>View published research articles from *PLOS ONE* for specific examples.<br><br>**NO authors have competing interests**<br><br>Enter: *The authors have declared that no competing interests exist.*<br><br>**Authors with competing interests**<br><br>Enter competing interest details beginning with this statement:<br><br>*I have read the journal's policy and the authors of this manuscript have the following competing interests: [insert competing interests here]*<br><br>* typeset	
**Ethics Statement**<br><br>Enter an ethics statement for this submission. This statement is required if the study involved:<br><br>• Human participants<br>• Human specimens or tissue<br>• Vertebrate animals or cephalopods<br>• Vertebrate embryos or tissues	The animal review number is 2023660H. All experimental animals were euthanized after being anesthetized with anesthetic drugs.

• Field research<br><br>Write "N/A" if the submission does not require an ethics statement.<br><br>General guidance is provided below. Consult the submission guidelines for detailed instructions. **Make sure that all information entered here is included in the Methods section of the manuscript.**	

**Format for specific study types**<br><br>**Human Subject Research (involving human participants and/or tissue)**<br>• Give the name of the institutional review board or ethics committee that approved the study<br>• Include the approval number and/or a statement indicating approval of this research<br>• Indicate the form of consent obtained (written/oral) or the reason that consent was not obtained (e.g. the data were analyzed anonymously)<br><br>**Animal Research (involving vertebrate animals, embryos or tissues)**<br>• Provide the name of the Institutional Animal Care and Use Committee (IACUC) or other relevant ethics board that reviewed the study protocol, and indicate whether they approved this research or granted a formal waiver of ethical approval<br>• Include an approval number if one was obtained<br>• If the study involved *non-human primates*, add *additional details* about animal welfare and steps taken to ameliorate suffering<br>• If anesthesia, euthanasia, or any kind of animal sacrifice is part of the study, include briefly which substances and/or methods were applied<br><br>**Field Research**<br><br>Include the following details if this study involves the collection of plant, animal, or other materials from a natural setting:<br>• Field permit number<br>• Name of the institution or relevant body that granted permission	
**Data Availability**<br><br>Authors are required to make all data underlying the findings described fully available, without restriction, and from the time of publication. PLOS allows rare exceptions to address legal and ethical concerns. See the PLOS Data Policy and FAQ for detailed information.	Yes - all data are fully available without restriction

Powered by Editorial Manager® and ProduXion Manager® from Aries Systems Corporation

A Data Availability Statement describing where the data can be found is required at submission. Your answers to this question constitute the Data Availability Statement and **will be published in the article**, if accepted.<br><br>**Important:** Stating 'data available on request from the author' is not sufficient. If your data are only available upon request, select 'No' for the first question and explain your exceptional situation in the text box.<br><br>Do the authors confirm that all data underlying the findings described in their manuscript are fully available without restriction?	
**Describe where the data may be found in full sentences. If you are copying our sample text, replace any instances of XXX with the appropriate details.**<br><br>• If the data are **held or will be held in a public repository**, include URLs, accession numbers or DOIs. If this information will only be available after acceptance, indicate this by ticking the box below. For example: *All XXX files are available from the XXX database (accession number(s) XXX, XXX.).*<br>• If the data are all contained **within the manuscript and/or Supporting Information files**, enter the following: *All relevant data are within the manuscript and its Supporting Information files.*<br>• If neither of these applies but you are able to provide **details of access elsewhere**, with or without limitations, please do so. For example:<br><br>*Data cannot be shared publicly because of [XXX]. Data are available from the XXX Institutional Data Access / Ethics Committee (contact via XXX) for researchers who meet the criteria for access to confidential data.*<br><br>*The data underlying the results presented in the study are available from (include the name of the third party*	All relevant data are within the manuscript and its Supporting Information files.

and contact information or URL).<br>• This text is appropriate if the data are owned by a third party and authors do not have permission to share the data.<br><br>* typeset	
Additional data availability information:	Tick here if the URLs/accession numbers/DOIs will be available only after acceptance of the manuscript for publication so that we can ensure their inclusion before publication.

Powered by Editorial Manager® and ProduXion Manager® from Aries Systems Corporation

Dear Prof□
I would like to submit the enclosed manuscript entitled"Exploring the Role of Nesprin1 in Isoproterenol-Induced Heart Failure Insights into Pathogenesis and Therapeutic Implications", which I wish to be considered for publication in"
PLOS One". No conflict of interest exits in the submission of this manuscript, and manuscript is approved by all authors for publication. I would like to declare on behalf of my co-author that the work described was original research that has not been published previously, and not under consideration for publication elsewhere, in whole or in part. All the authors listed have approved the manuscript that is enclosed.

Nesprin is a nuclear envelope protein. Nesprin proteins interact with proteins containing SUN domains through their KASH domain, forming LINC complexes. LINC complexes play a significant role in connecting the cytoskeleton to the nuclear skeleton. The integrity of the LINC complex structure is crucial for maintaining nuclear homeostasis, signal transduction, and gene transcription. Studies have found that Nesprin mutations affect the integrity of LINC complexes, leading to nuclear envelope diseases such as dilated cardiomyopathy. Cardiovascular diseases pose a significant threat to human health. Previous research on cardiovascular diseases has mainly focused on nuclear and extranuclear factors, lacking studies on nuclear envelope factors. Therefore, we investigated the role of Nesprin1 in heart failure from aspects of oxidative stress, DNA damage, signal transduction, and epigenetic regulation. We hope to provide potential targets for drug treatment of heart failure.

I deeply appreciate your consideration of my manuscript, and I look forward to your reply.If you have any queries, please don't hesitate to contact me at the address below.

Thank you and best regards.

Yours sincerely,

Corresponding author:Tenglin
E-mail: tenglin@ctgu.edu.cn

Manuscript Click here to access/download;Manuscript;manuscript without maker.docx

Exploring the Role of Nesprin-1 in Isoproterenol-Induced Heart Failure Insights into Pathogenesis and Therapeutic Implications

Qin Qin[1,2#] , Dong Cengyuan[1,2#] , Zhou Zi-yi[1,2] ,Liu Yangyuanzhi[1,2], Cao Chunyu [2,4], TENG Lin[1,3*]

1. Department of Cardiology, Yichang Central People's Hospital/The First Clinical Medical College, Three Gorges University, Yichang 443003, Hubei, People's Republic of China

2. School of Basic Medicine, China Three Gorges University, Yichang 443000, Hubei, People's Republic of China

3. King's College London British Heart Foundation Centre of Research Excellence, School of Cardiovascular and Metabolic Medicine & Sciences, London SE5 9NU, U.K

4. College of Basic Medical Sciences, Hubei Key Laboratory of Tumor Microencironment and immunotherapy, China Three Gorges University, Yichang 443000, Hubei, People's Republic of China

# Qin Qin and Dong Cengyuan have equally to this work.

* Correspondence to: TENG Lin, MD, Department of Cardiology, Yichang Central People's, NO,183 Yiling Road, Yichang 443003, Hubei, People's Republic of China. E-mail address: tenglin@ctgu.edu.cn

Objective: In this study, we used an isoprenaline-induced heart failure model to investigate the role of Nesprin-1 in the development and progression of heart failure and to clarify the underlying molecular mechanisms.

Methods: A mouse model of heart failure was induced by daily intraperitoneal injection of isoproterenol (5 mg/kg/day) for 24 consecutive days. The body weight of mice was monitored daily. The diastolic left ventricular diameter (LVDd), systolic left ventricular diameter (LVDs), ejection fraction (EF) and short axis shortening rate (FS) of mice were evaluated by echocardiography of small animal hearts. The heart failure model was validated by gross cardiac observation, WGA staining, and Western blot analysis of B-type Natriuretic Peptide (BNP) expression. Hematoxylin-Eosin staining of heart tissue was performed to assess myocardial structure, cell morphology and pathological changes. Quantitative determination of malondialdehyde (MDA) and superoxide dismutase (SOD) expression was performed to evaluate oxidative stress levels. The expression of Nesprin-1 and connexin 43 (Cx43) was detected by immunofluorescence staining. Immunofluorescence staining and Western blot were used to detect the effects of Nesprin-1 expression changes on p53-binding protein 1 (53BP1), phosphorylated histone H2AX (γH2AX), H3 lysine 9 trimethylation (H3K9me3), H3 lysine 27 trimethylation (H3K27me3) expression.

In addition, we used Western blot to analyze the expression of the extracellular regulatory protein kinase (ERK) pathway.

Results: Compared with the control group, mice in the isoproterenol-induced heart failure model group exhibited significant increases in both body weight and heart weight (P<0.01), enlargement of cardiomyocyte cross-sectional area (P<0.01), markedly elevated BNP expression (P<0.01). The echocardiographic results showed that the LVDd and LVDs in diastolic phase were significantly increased, and EF and FS were significantly decreased in mice with heart failure(P<0.01). Histopathological analysis demonstrated disordered myocardial structure, vascular proliferation, and myocardial hemorrhage in model group mice. The expression of Nesprin-1 was significantly downregulated in the model group (P<0.01), accompanied by abnormal nuclear envelope localization that resulted in distorted nuclear morphology of cardiomyocytes, including increased nuclear area and perimeter, elevated nuclear aspect ratio (P<0.01) , and decreased nuclear circularity(P<0.01). Additionally, the model group showed significantly increased levels of the oxidative stress marker MDA coupled with decreased SOD activity (P<0.05), reduced Cx43 expression (P<0.01), upregulated expression of DNA damage markers (γH2AX and 53BP1)(P<0.01), significantly elevated levels of H3K9me3 and H3K27me3 (P<0.01), and markedly enhanced activation of the ERK signaling pathway (P< 0.01).

Conclusion: This study revealed that downregulation of Nesprin-1 may serve as a crucial driver in the progression of heart failure. In the isoproterenol-induced murine heart failure model, reduced Nesprin-1 expression leads to multiple pathological alterations, including nuclear homeostasis imbalance, decreased Cx43 expression, elevated oxidative stress, accumulated DNA damage, and disordered epigenetic regulation. The underlying mechanism may involve activation of the ERK signaling pathway.

Keywords: Heart failure, Nesprin-1, Isoproterenol, Cardiac remodeling, Dysfunction, Epigenetic regulation, DNA damage

Introduction

Heart failure (HF), or cardiac failure, signifies the heart's diminished capacity to efficiently pump blood, resulting in venous congestion and inadequate arterial perfusion due to compromised systolic and/or diastolic functions[1-4]. This condition represents the terminal phase of heart disease and stands as a leading cause of cardiovascular-related mortality worldwide. HF affects approximately 1% to 2% of the global population, with a notably higher prevalence among the elderly[5-7]. Sympathetic nervous system (SNS) overactivity is a hallmark of HF, correlating with disease severity and prognosis. Under normal circumstances, the cardiac sympathetic nervous system enhances cardiac output, gene transcription, and metabolism by releasing neurotransmitters and engaging β-adrenergic receptors (β-AR)[8, 9]. However, excessive SNS activation detrimentally affects the cardiovascular system, potentially precipitating or exacerbating HF[10, 11]. This overdrive leads to heightened heart rate and contractility, inducing a sustained high-load state that depletes cardiac energy reserves, impeding cardiac function. Furthermore, it promotes adverse structural cardiac changes such as ventricular hypertrophy and atrial dilation, diminishing cardiac pump efficiency[12-14]. Increased arterial vasoconstriction further strains the heart by elevating vascular tension. Excessive sympathetic stimulation also incites inflammation and oxidative stress, damaging myocardial cells and hastening disease progression. Disruption of neuroendocrine system balance, including the renin-angiotensin-aldosterone system, can exacerbate fluid retention and increase blood volume, exacerbating cardiac workload[15-18]. Current HF management strategies primarily target the sympathetic nervous system with medications like β-blockers, angiotensin-converting enzyme inhibitors, angiotensin II receptor blockers, and aldosterone receptor antagonists. Despite these interventions, HF incidence, readmission rates, and mortality remain high, underscoring the need for novel therapeutic approaches[19-23].

Traditionally, HF research focused on intracellular factors, neglecting the nuclear envelope's role. The nuclear envelope (NE) is pivotal, spatially segregating nuclear genetic material and regulating macromolecular transport[24]. The Linker of

Nucleoskeleton and Cytoskeleton (LINC) complex, anchored to the NE, includes Nesprin proteins, which bridge the nuclear and cytoskeletal proteins, contributing to cellular structure and function. Nesprin mutations are implicated in various diseases, including Dilated Cardiomyopathy (DCM) and Emery-Dreifuss Muscular Dystrophy (EDMD), highlighting their importance in muscle physiology[25-27]. Nesprin family proteins, integral to the NE, include Nesprin-1 and Nesprin-2, which interact with actin, intermediate filaments, and the nuclear envelope, influencing cell signaling, morphology, and mechanical properties[28]. These proteins regulate gene expression, chromatin structure, and epigenetic modifications, impacting cell function and pathology. Mutations in Nesprin-1 and Nesprin-2 are associated with abnormal nuclear morphology, positioning, and chromatin organization, contributing to DCM and EDMD. Nesprin alterations also affect intercalated disc protein localization, leading to conduction defects in Nesprin-associated cardiomyopathies[27, 29, 30].

Given this background, we hypothesize that the dysregulation of Nesprin-1 expression disrupts the structural connection between the nucleus and the cytoskeleton, thereby impairing nuclear homeostasis, interfering with signal transduction and causing abnormal transcription regulation, which ultimately leads to myocardial remodeling and contractile dysfunction, and then heart failure. Our study aims to elucidate Nesprin-1's role and molecular mechanisms in HF, providing insights for diagnosis and treatment. Understanding Nesprin-1's function may reveal therapeutic targets, guiding future HF therapies.

Materials and methods

Drugs and Chemicals

Please rephrase and indicate as - XX and YY are obtained from AA. If the chemicals/ consumables were purchased, please indicate that they are obtained from. Otherwise, it could be indicated as given/ or provided.

MedChemExpress provided isoproterenol (HY-B0468) and isoflurane, while Biosharp supplied 4% paraformaldehyde (BP003), hematoxylin-eosin staining (BP019), and other staining-related reagents. Sinopharm Chemical Reagent offered basic reagents such as paraffin (69019361) and xylene (10023418). Antibodies were mainly sourced from Boster, including Cx43 antibody (A00599), BNP antibody (M01186-3), 53BP1 antibody (BM5099), γH2AX antibody (BM4148), H3K9me3 antibody (BM5079), H3K27me3 antibody (BM4341), and ERK antibody (BM4156).

Protein-related reagents were primarily obtained from Beyotime, including RIPA lysis buffer (P0013B) and BCA protein assay kit (P0010). Additionally, Amresco provided electrophoresis reagents such as TEMED (Amresc00761) and acrylamide (Exp2016109), while Jackson supplied HRP-labeled secondary antibodies, specifically HRP-conjugated goat anti-rabbit (BA1054), HRP-conjugated rabbit anti-goat (BA1060), HRP-conjugated goat anti-mouse (BA1051), and HRP-conjugated goat anti-rat (BA1058). Other essential reagents included WGA stock solution (BBL-0351) from Sigma-Aldrich, DAPI stock solution (C1002) from Solarbio, and cytoplasmic/nuclear protein extraction kit (KGP150) from Nanjing KeyGen Biotech.

Establishment of Isoproterenol-Induced Heart Failure Model in Mice

The male C57BL/6 mice weighing 20-22g were purchased from the Animal Experimental Center of Three Gorges University. All mice were housed in specific pathogen-free (SPF) animal facilities at Three Gorges University (Yichang, Hubei, China). The experimental animal quality certification number is 42010200009428. The experimental animal use permit number is 00305510. Twenty-four C57BL/6 mice were randomly divided into two groups: model group and control group. In the model group, mice were administered intraperitoneal injections of isoproterenol (ISO) at a dosage of 5mg/kg. In the control group, mice were administered intraperitoneal injections of an equivalent volume of medical-grade physiological saline. Two groups received intraperitoneal injections continuously for 24 days.

Cardiac Function Assessment

Mice were anesthetized with isoflurane, and after achieving stable anesthesia, the hair in the chest area was removed. The mice were then placed in a supine position and fixed on an ultrasound examination table. Ultrasound coupling agent was applied to the chest area. Using small animal ultrasound, M-mode images of the long axis and short axis of the mouse heart were obtained. Parameters including ejection fraction (EF), fractional shortening (FS), left ventricular end-diastolic diameter (LVEDd), and left ventricular end-systolic diameter (LVESd) were measured and calculated to assess cardiac function in mice. Statistical analysis was performed to analyze the cardiac

function of mice.

Measurement of Body and Heart Weight

Body weight of the mice was measured before each dose.At least 24 hours after the last dose the mice were anesthetized, and their chests were swiftly opened using surgical scissors and forceps to extract the hearts. The hearts were rinsed in pre-cooled physiological saline, and then scissors were used to trim the blood vessels and connective tissues surrounding the heart. The hearts were then rinsed again with fresh physiological saline and dried with filter paper. Gross images of the hearts were captured, and the total heart weight was measured. Some samples were frozen in dry ice and stored in-80℃ refrigerator, while others were fixed in 4% polyformaldehyde fixative for easy observation of histological morphology.

Pathological Section Staining

HE staining: After dehydration, embedding, and sectioning of tissue fixed with 4% polyformaldehyde, the paraffin sections are dewaxed to water. Stain with hematoxylin for 4 minutes, followed by 2-minute rinsing with tap water to remove excess stain. The sections are then differentiated with 0.8% hydrochloric acid alcohol for 2 seconds, rinsed again with tap water, immersed in eosin solution for 20 seconds, adjusted with 95% ethanol for 5 seconds, and dehydrated with anhydrous ethanol for 2 minutes. Finally, the sections are sealed with a transparent environmental agent and observed under a microscope.

WGA staining: The paraffin sections were sequentially dewaxed with eco-friendly degreasing agents (1), (2), and (3) for 10 minutes each, followed by 5-minute immersion in anhydrous ethanol, 95% ethanol, and 75% ethanol respectively. The sections underwent three 3-minute rinses with distilled water. Antigen retrieval was performed by high-pressure treatment of the sections in boiling citric acid (pH6.0) solution for 2 minutes. After cooling, the sections were immersed in distilled water for three 5-minute washes. A circular mark was made on the section using a histological pen, then it was immersed in TBST buffer. A 1:200 diluted WGA working solution (50 uL/section) was added and incubated under light protection for 30 minutes, followed by three 5-minute TBST washes. After removing TBST, a 1:500 diluted

DAPI working solution (50 uL/section) was applied to stain nuclei for 5 minutes. Following TBST rinsing, the sections were mounted with anti-fluorescence quenching medium and stored at 4℃ under light protection. Finally, microscopic examination and image analysis were conducted.

The biochemical kits detects the oxidative stress indicators

Sample Preparation: Myocardial tissue was mixed with PBS buffer at ratios of 1:10 (for MDA detection) and 1:9 (for SOD detection), homogenized, and centrifuged at 10,000 g for 15 min at 4 ℃. The supernatant was collected for subsequent analysis.

Reagent Preparation: MDA detection working solution (including probe working solution and standard) and SOD detection working solution (including enzyme reaction solution) were prepared according to the kit instructions.

MDA Measurement: The sample was mixed with the detection working solution, incubated at 95 ℃ for 40 min, cooled on ice for 5 min, and centrifuged at 10,000 g for 10 min. The supernatant was measured at 532 nm absorbance.

Calculation formula:

MDA(umol/gprot)=[(A_{sample}-A_{blank}) /(A_{standard}-A_{blank})]×C_{standard}×dilution factor / protein concentration

SOD Measurement: 20 uL of sample was added, incubated at 37 ℃ for 20 min, and measured at 450 nm absorbance.

Calculation formulas:

SOD inhibition rate (%) = [(ΔA_{control} - ΔA_{sample}) / ΔA_{control}] × 100%

SOD activity (U/mg prot) = Inhibition rate / (100% - Inhibition rate) × 12 / protein concentration

(Note: In the formulas, C_{standard} is the standard concentration, ΔA is the absorbance difference, and 12 is the conversion factor for reaction volume/sample volume.)

Immunofluorescence Staining Protocol for Mouse Cardiac Tissue

Initially, tissues fixed with 4% paraformaldehyde were processed through dehydration,

embedding, and sectioning, followed by dewaxing the paraffin sections to water. Antigen retrieval was then performed by incubating the sections in preheated 95° C sodium citrate buffer (pH 6.0) under high-pressure heating for 10 minutes. After natural cooling, the sections were washed three times with phosphate-buffered saline (PBS), 3 minutes each. Excess moisture was removed using absorbent paper, and the tissue areas were circled with an immunohistochemical pen before applying diluted normal goat serum for 30-minute blocking at room temperature. After blocking, excess liquid was removed (without washing), and diluted primary antibody was directly applied for overnight incubation at 4 ℃ in a humidified chamber. The following day, sections were thoroughly washed with PBS (⩾3 times, 3 minutes each), dried, and treated with diluted fluorescent secondary antibody for 1-hour incubation at 25 ℃ in a humidified chamber, followed by four additional PBS washes (3 minutes each). For nuclear staining, DAPI was applied under light-protected conditions for 5 minutes, followed by four PBS washes (5 minutes each) to remove excess dye. Finally, sections were dried and mounted with anti-fade mounting medium for observation and image acquisition under a fluorescence microscope.

Western Blot Experimental Procedure

Protein extraction was first performed by mincing tissue samples in a 2mL homogenizer, followed by adding 400 uL lysis buffer containing PMSF for ice-cold homogenization with repeated processing at 5-minute intervals until complete tissue disruption was achieved. After 30 minutes of lysis, the lysate was centrifuged at 12,000 rpm for 5 minutes at 4 ℃, and the supernatant was aliquoted and stored at -20 ℃. Protein concentration was determined using the BCA method: samples were diluted 1:19 with PBS, and a standard curve (0.2-1mg/mL BSA) was prepared and loaded onto a 96-well plate alongside samples (standards in duplicate, samples in triplicate). After adding BCA working solution, the plate was incubated at 37° C for 30 minutes protected from light, and absorbance at 568 nm was measured to calculate protein concentration. Protein samples were mixed with 5×loading buffer and denatured by boiling for 10 minutes before storage.

For electrophoresis, separating and stacking gels were prepared, and 40ug total protein was loaded per well. Initial electrophoresis was performed at 80V until samples entered the separating gel, then switched to 120V for approximately 1.5 hours until the bromophenol blue reached the gel bottom. Prior to transfer, PVDF membranes were cut and activated with methanol, and the transfer sandwich was assembled in the order of "negative plate-fiber pad-filter paper-gel-membrane-filter paper-fiber pad-positive plate" for constant current transfer. After transfer, membranes were blocked with 5% skim milk in TBST (2% BSA for phosphoproteins) at room temperature for 2 hours.

Following overnight primary antibody incubation at 4 ℃, membranes were washed 6 times with TBST, then incubated with HRP-conjugated secondary antibody at 37 ℃ for 2 hours, followed by 5 additional TBST washes. For ECL detection, enhanced solution and peroxidase solution were mixed in equal proportions and applied to membranes. After several minutes of reaction, X-ray films were exposed and processed through development and fixation to obtain results. Throughout the procedure, care was taken to maintain samples at low temperatures, ensure bubble-free transfer, and keep membranes moist during antibody incubations.

Statistical analysis

All quantitative data were expressed as mean ± standard deviation (X±SD). The comparison between the two groups was evaluated using the t-test. *P* value <0.05 indicated statistically significant differences. The data were analyzed using GraphPad Prism 10.0 software.

3.Result

1.Establishment and Validation of Isoproterenol-Induced Heart Failure Mouse Model

Echocardiographic evaluation revealed notable alterations in cardiac parameters across the two experimental groups(Fig1A-B). Mice in the model group displayed significant increases in left ventricular end-diastolic diameter (LVEDd) and left ventricular end-systolic diameter (LVESd) compared to the control groups(Fig1C-D)(*P*<0.01). Conversely, ejection fraction (EF) and fractional

shortening (FS) were markedly reduced in the model group(Fig1E-F)(P<0.01). These findings underscore the effectiveness of the isoproterenol-induced myocardial hypertrophy model in inducing structural and functional changes in cardiac function.

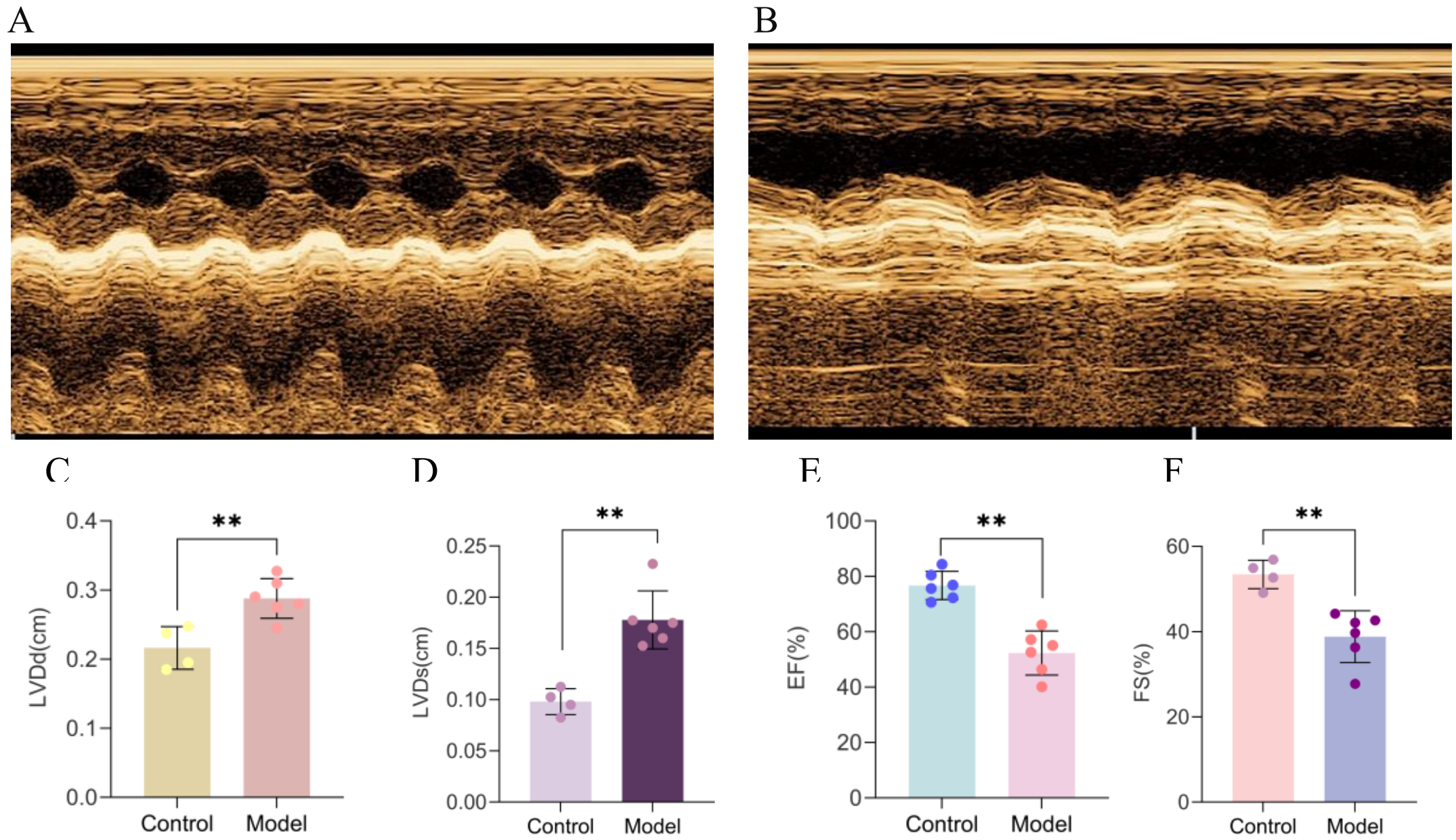

Figure 1: Isoproterenol-induced Decrease in Cardiac Function in Mice. A: Echocardiogram of the control group mice; B: Echocardiogram of the model group mice; C-F: Left ventricular diameter (LVDd), left ventricular diameter at systole (LVDs), ejection fraction (EF), and short-axis shortening rate (FS) of the left ventricle in mice; n≥4; Compared to the control group, **P<0.01

Assessment of cardiac weight provided additional insights into physiological changes among the experimental groups. Mice in the isoproterenol-induced heart failure model group exhibited a significant increase in body weight starting from day 13 of drug administration, compared to controls(Fig2A). Moreover, body weight and cardiac weight significantly increased in the model group after 24 days of isoproterenol administration, indicative of myocardial hypertrophy(Fig2B-C)(P<0.01). WGA staining demonstrated a significant increase in cardiomyocyte cross-sectional area in the model group compared to controls(Fig2D-E)(P<0.01).By examining BNP in both groups using Western Blot, we found that BNP was significantly elevated in the model group compared to the control group(Fig2F-G)(P<0.01). These findings highlight the capacity of isoproterenol to induce cardiac hypertrophy in mice.

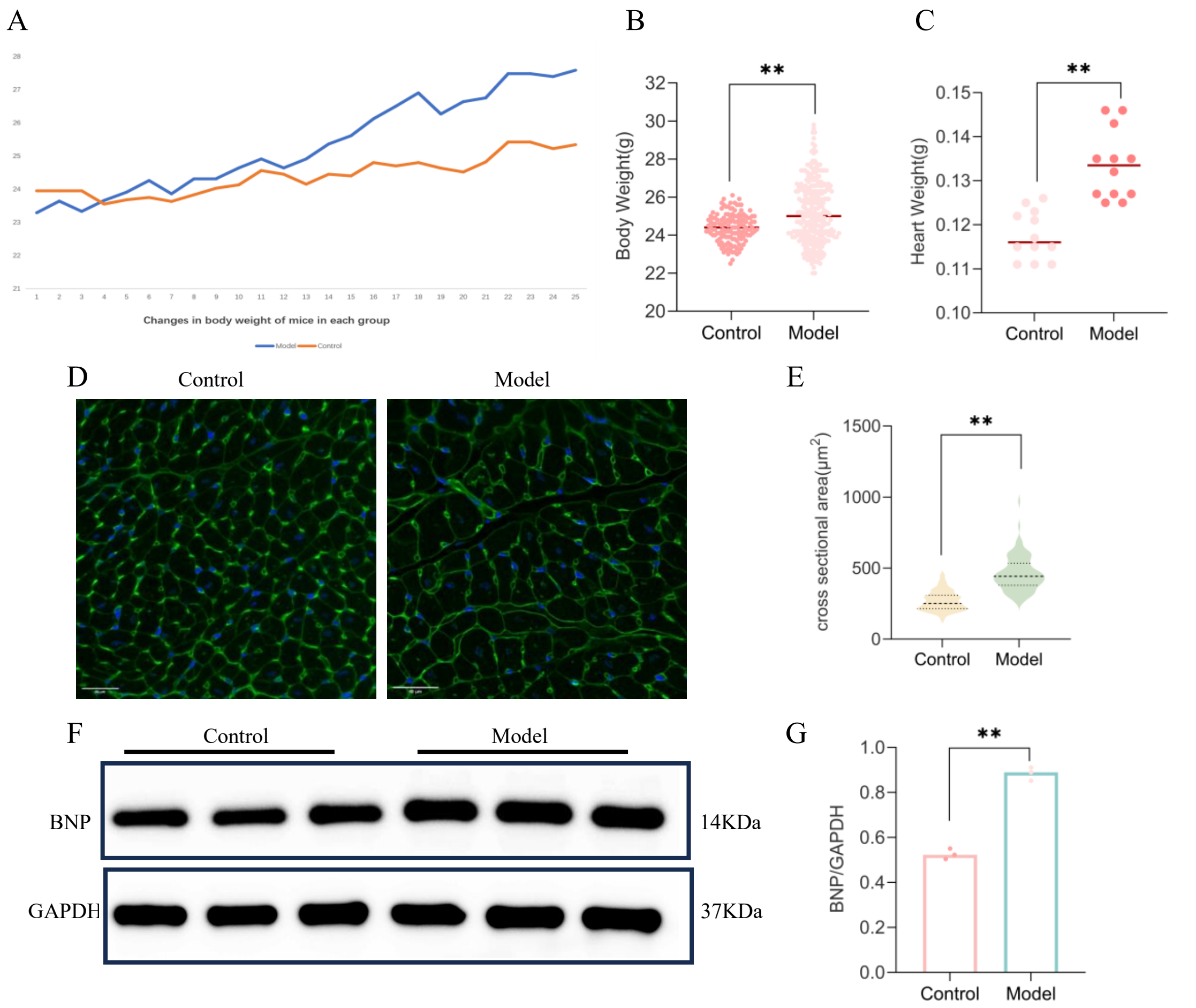

Figure2：Isoproterenol-induced heart failure in mice. A: Graph of changes in mouse body weight; B-C: Body and cardiac weights of the two groups of mice; n=12; D-E: Myocardial WGA staining of mice; n=5; F-G: BNP protein expression levels of the two groups of mice; n=3. Compared with the control group, **P<0.01

Morphological and pathological staining of the heart in mice revealed significant differences in myocardial structure between the two groups (Figure 3A). Compared to the control group, the model group mice exhibited a significantly larger heart volume (Figure 3A). A comparison of cross-sectional images from the hearts of the two groups showed that the ventricular cavity in the model group mice was notably enlarged (Figure 3B). HE staining results indicated that the model group mice exhibited disordered myocardial structure, vascular proliferation, and myocardial hemorrhage (Figure 3C-D). These findings collectively highlight the pathological changes in myocardial hypertrophy induced by isoproterenol in mice.

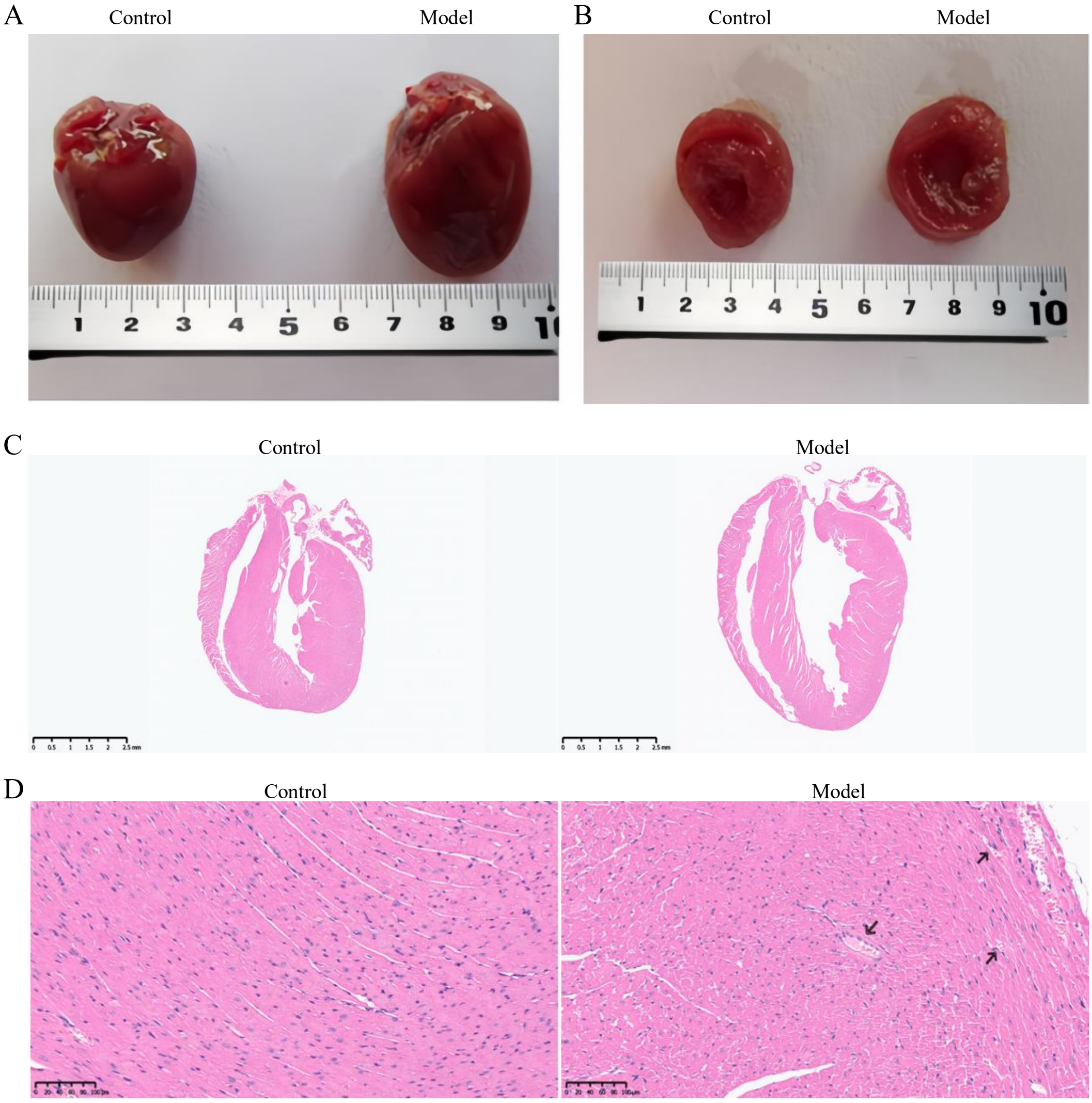

Figure 3: Gross view and pathological staining of mouse heart . A: Gross view of mouse heart; B: Coronary section of mouse heart; C-D: HE staining of mouse myocardial tissue; n=5

2.The expression of Nesprin-1 in the heart of mice with isoproterenol induced heart failure was significantly reduced

The immunofluorescence analysis revealed a significant decrease in Nesprin-1 expression in the hearts of mice induced with isoproterenol-induced heart failure(Fig4A)(P<0.01).By fluorescent staining of Nesprin-1 with specific antibodies, we observed that Nesprin-1 was mainly located at the nuclear membrane of cardiomyocytes (Fig4A). This localization is consistent with its function in maintaining nuclear membrane structure and nuclear-cell skeleton connection.

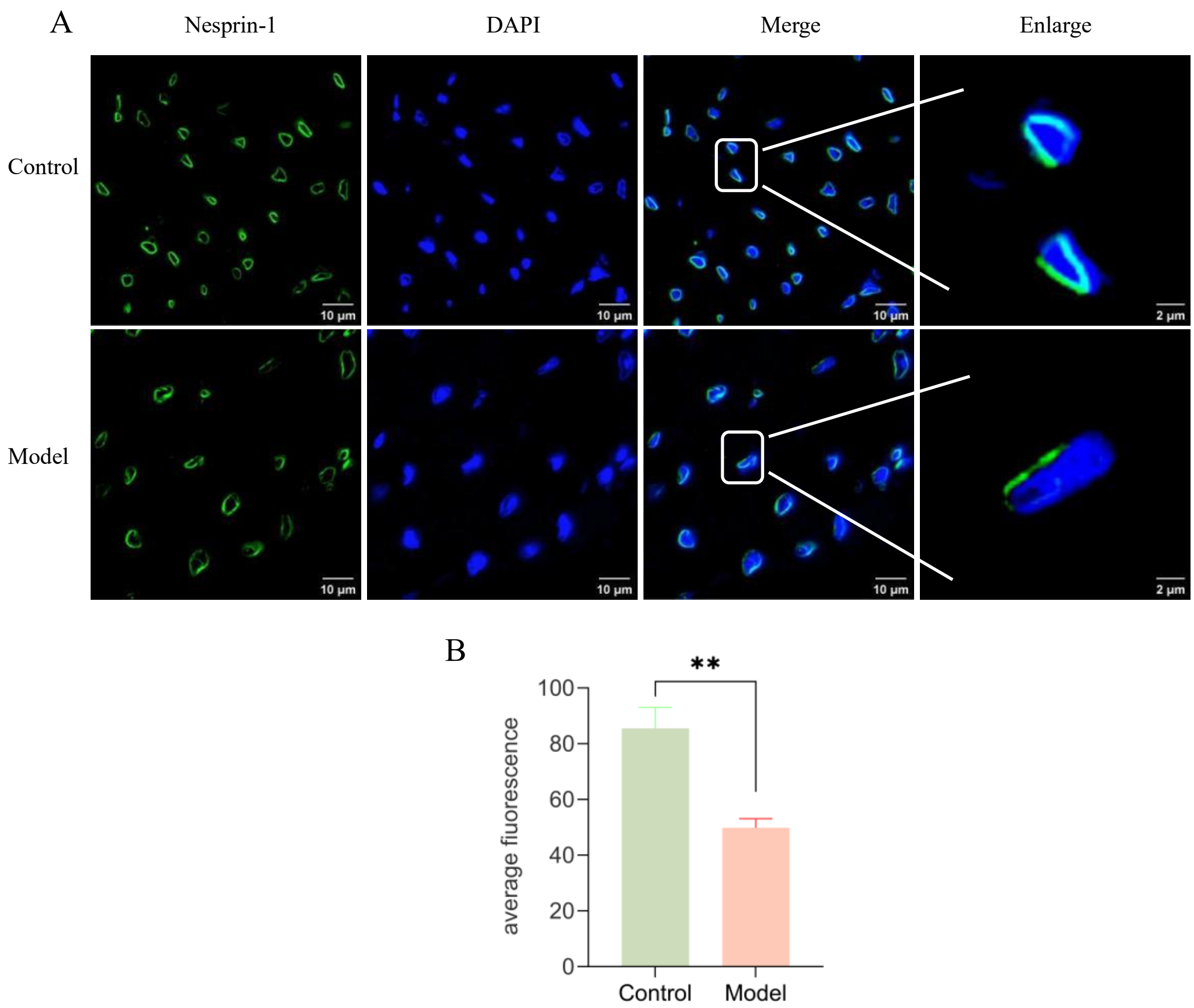

Figure 4: Expression changes of Nesprin-1 in myocardial tissue. A-B: Immunofluorescence staining of Nesprin in mouse myocardial tissue; n=5; Compared with the control group, $^{**}P<0.01$

3.Dysregulation of Nesprin-1 expression in cardiac tissue of isoproterenol induced heart failure mouse model affects nuclear homeostasis in heart failure

In our experimental study, we employed immunofluorescence to investigate the effects of Nesprin-1 expression changes on nuclear homeostasis in cardiomyocytes. By randomly selecting 40 fluorescently stained pathological sections from five model groups, we obtained 40 tissue micrographs. Ten nuclei were randomly selected from each image and analyzed using ImageJ software for morphological parameters including nuclear area, perimeter, and aspect ratio. Results demonstrated that compared with the control group, the nuclear morphology of cardiomyocytes in the model group exhibited significant abnormalities, characterized by marked increases in nuclear area, perimeter, and aspect ratio, along with a notable reduction in nuclear roundness (Figure 5A)($P<0.01$). Furthermore, immunofluorescence staining revealed partial or complete absence of Nesprin-1 on the nuclear membrane in some cardiomyocytes of the model group (Figure 5B)($P<0.01$). These findings indicate that

Nesprin-1 expression dysregulation affects nuclear homeostasis in ISO-induced HF mouse models, though the causal relationship between these two factors requires further investigation.

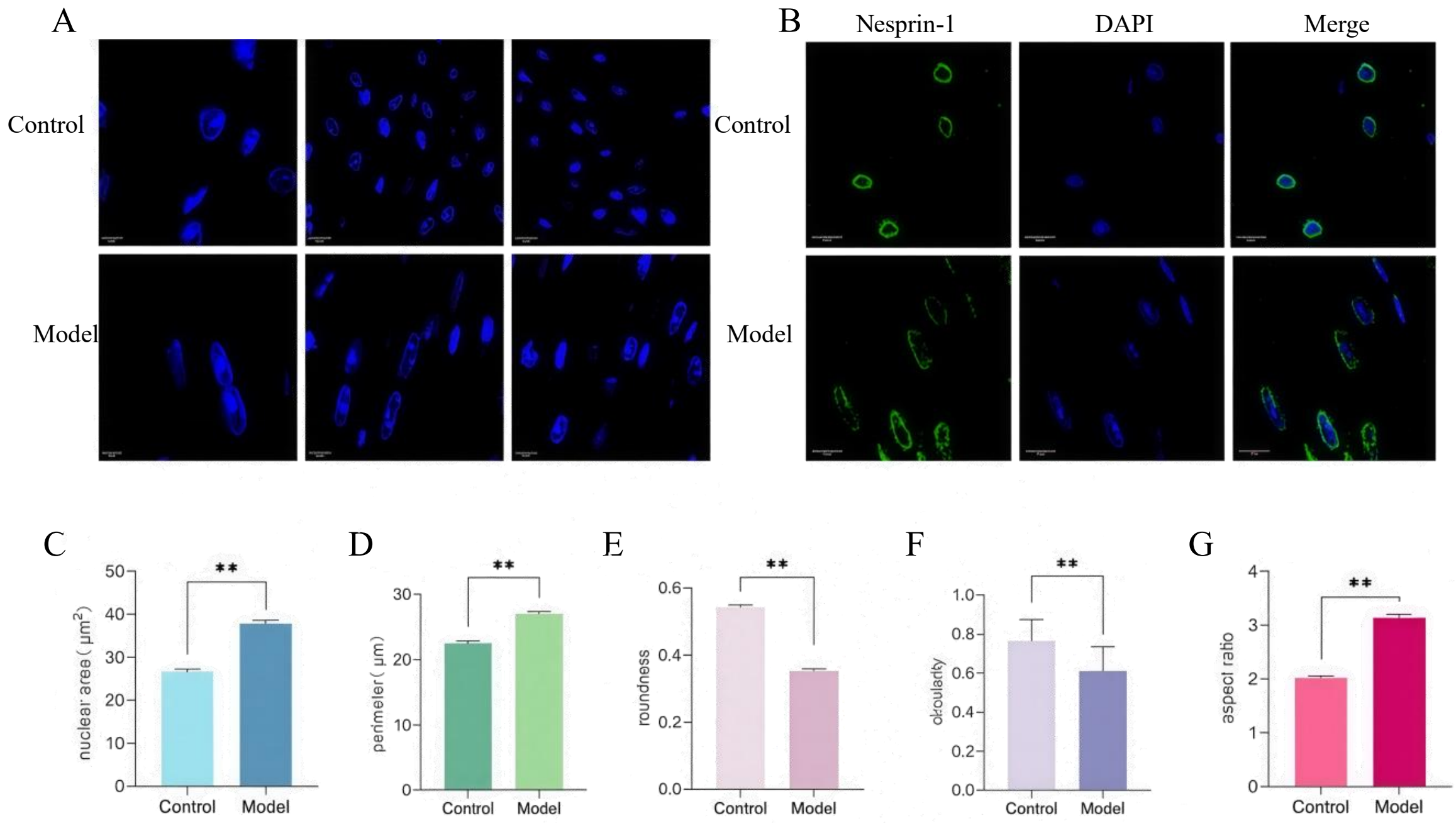

Figure 5: Nuclear Homeostasis Disruption in Heart Failure Mice. Note: A: Changes in nuclear morphology; B: Nesprin-1 immunofluorescence staining; C-G: Nuclear area, nuclear perimeter, nuclear roundness , and aspect ratio of the two groups of mice; n=5; Compared to the control group, ***P*<0.01

4.In a mouse model of heart failure with reduced Nesprin-1 expression, oxidative stress responses were increased

Oxidative stress, characterized by an imbalance between oxidizing agents and antioxidant defenses, predisposes tissues to oxidation. This imbalance fuels neutrophilic inflammation, upregulates protease secretion, and fosters the generation of various oxidative intermediates. Acknowledged as a detrimental factor contributing to aging and various diseases, oxidative stress primarily stems from the overproduction of free radicals within the body. In line with this, Nesprin-1 depletion in Drosophila melanogaster has been correlated with mitochondrial dysfunction, thereby inducing oxidative stress[31]. To investigate this phenomenon, we evaluated the expression levels of malondialdehyde (MDA) and superoxide dismutase (SOD) in cardiac tissue. Notably, compared to the control groups, the model group exhibited heightened MDA activity and diminished SOD content(Fig6A-B)(*P*<0.05). These results indicate that oxidative stress levels were significantly increased and

antioxidant capacity was inhibited in the Nesprin-1 dysregulated HF model.

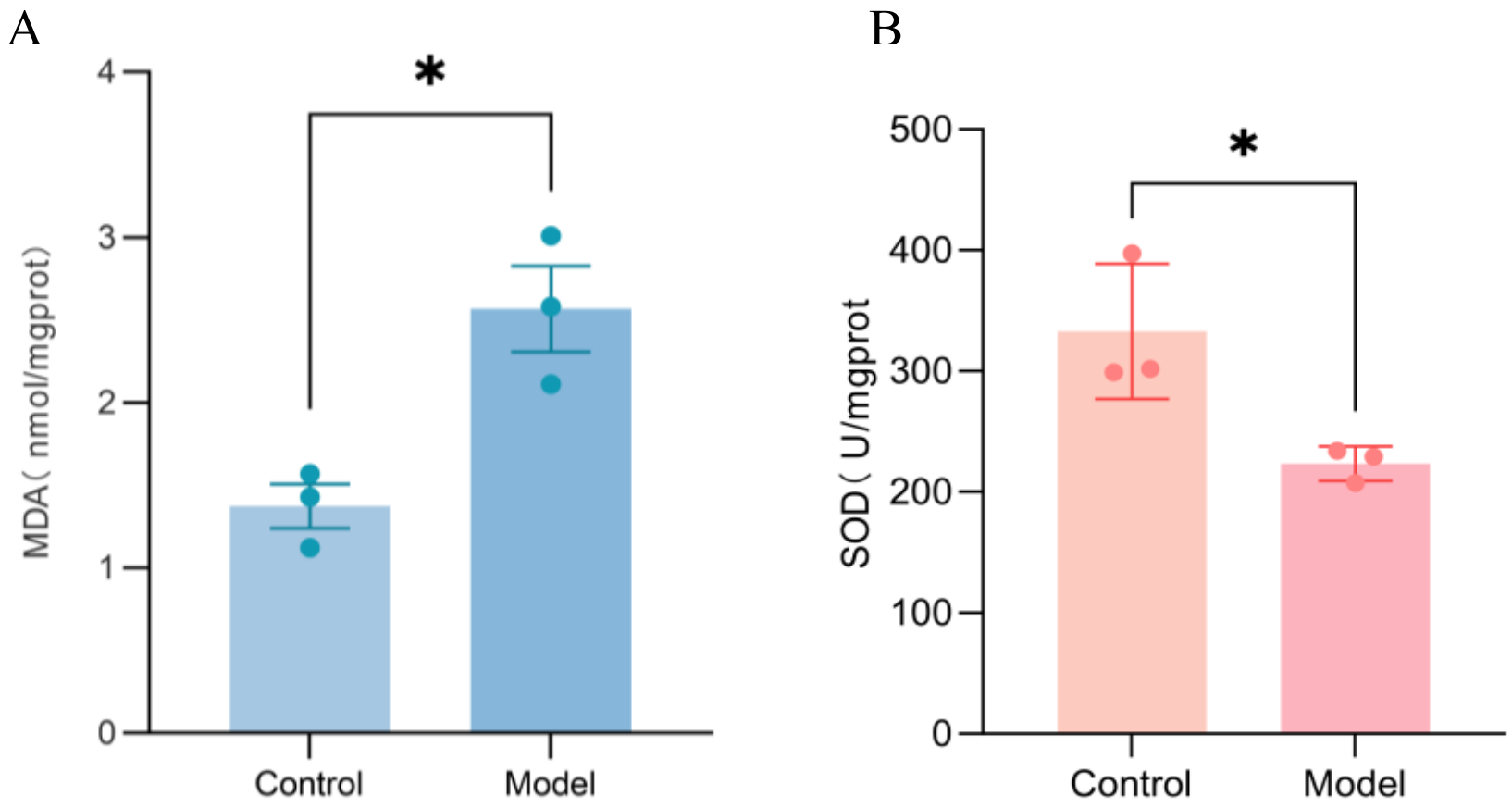

Figure 6: Oxidative stress aggravation in heart failure mice. A-B: Quantitative analysis of MDA and SOD in mouse myocardium; MDA: malondialdehyde; SOD: superoxide dismutase; n=3; compared with the control group, *P<0.05

5.The expression of Cx43 was downregulated in the myocardium of mice with dysregulation of Nesprin-1 expression in heart failure

Conduction dysfunction is a hallmark feature observed in the isoproterenol-induced heart failure mouse model. Cx43, primarily localized at the intercalated discs within mature cardiac myocytes, plays a pivotal role in facilitating electrical conduction among myocytes and enabling chemical signaling and energy substrate exchange, essential for coordinating cellular function[32]. Through immunofluorescence analysis, we investigated the expression levels of Cx43 in mice from each experimental group.Our results demonstrate a significant reduction in Cx43 expression specifically within the model group(Fig7A-B)(P<0.01). In addition, combined with our previous studies, this result will provide a reliable experimental basis for our further study on how the reduction of Nesprin-1 expression affects the localization and function of Cx43 and jointly participates in the occurrence of conduction dysfunction in ISO-induced HF model.

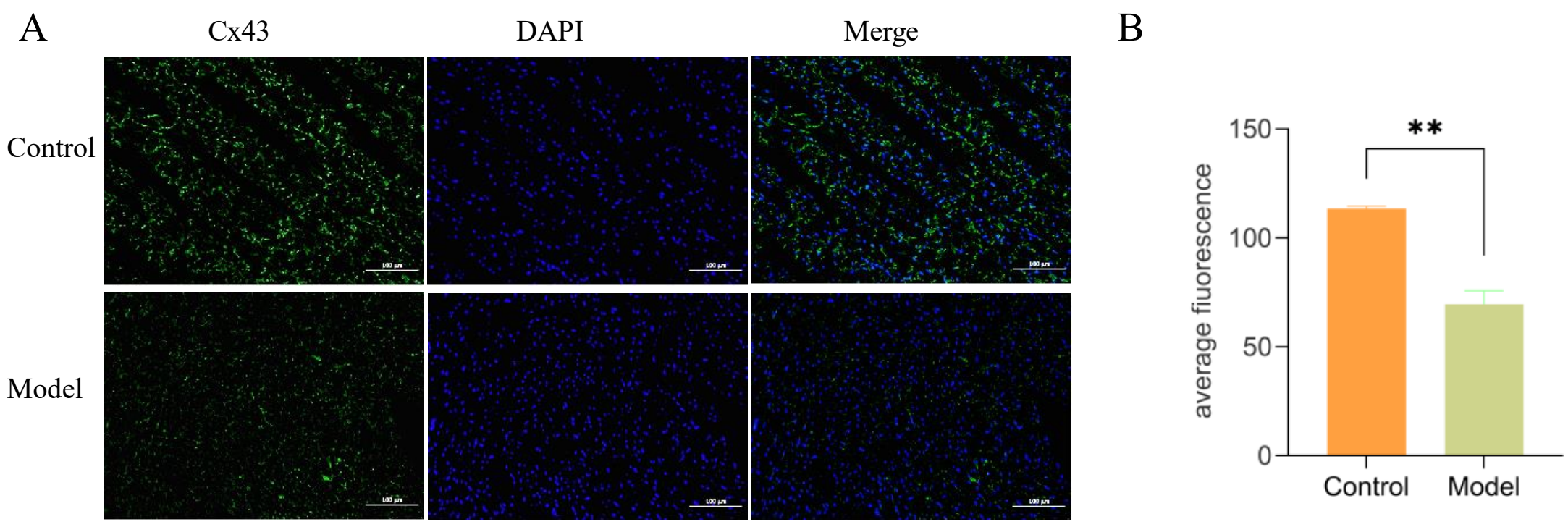

Figure 7: Expression of Cx43 in both groups of mice. A: Cx43 expression was detected by immunofluorescence; B Quantitative analysis of the Cx43; n=5; compared with the control group, ***P*<0.01

6.The myocardial DNA damage was exacerbated in mice exhibiting Nesprin-1 expression dysregulation during heart failure

Decreased Nesprin expression is associated with activated cellular DNA damage in the isoproterenol-induced heart failure mouse model. Nesprin plays a crucial role in maintaining genomic integrity, and its reduction may lead to impaired DNA repair mechanisms[33]. To investigate this phenomenon, immunofluorescence and Western blot analyses were conducted to assess the expression levels of 53BP1 and γH2AX in cardiac tissue. Our results reveal an increased expression of 53BP1 and γH2AX in the model group compared to the control groups(Fig8A-D)(*P*<0.01). In agreement with this, Western blot indicated elevated levels of DNA damage in Nesprin-deficient hearts(Fig9A-C)(*P*<0.01).These results suggest that the decrease in Nesprin-1 expression may interact with the accumulation of DNA damage in the occurrence of HF.

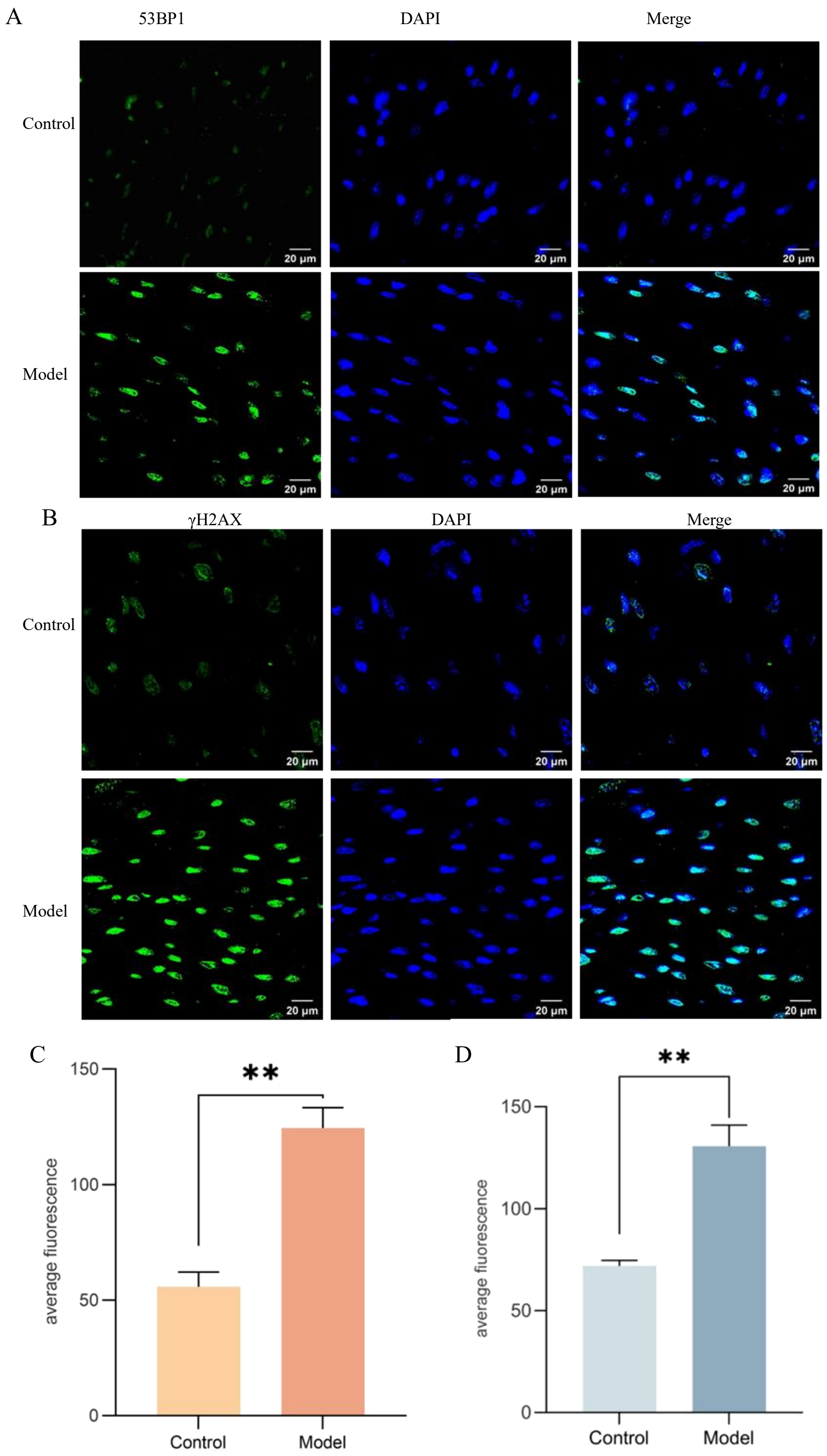

Figure 8: Increased DNA damage in myocardium of mice with heart failure. A-D: Detection of expression of 53BP1 and γH2AX by immunofluorescence; 53BP1: P53 binding protein 1; γH2AX: phosphorylated histone; n=5; compared with the control group,***P*<0.01

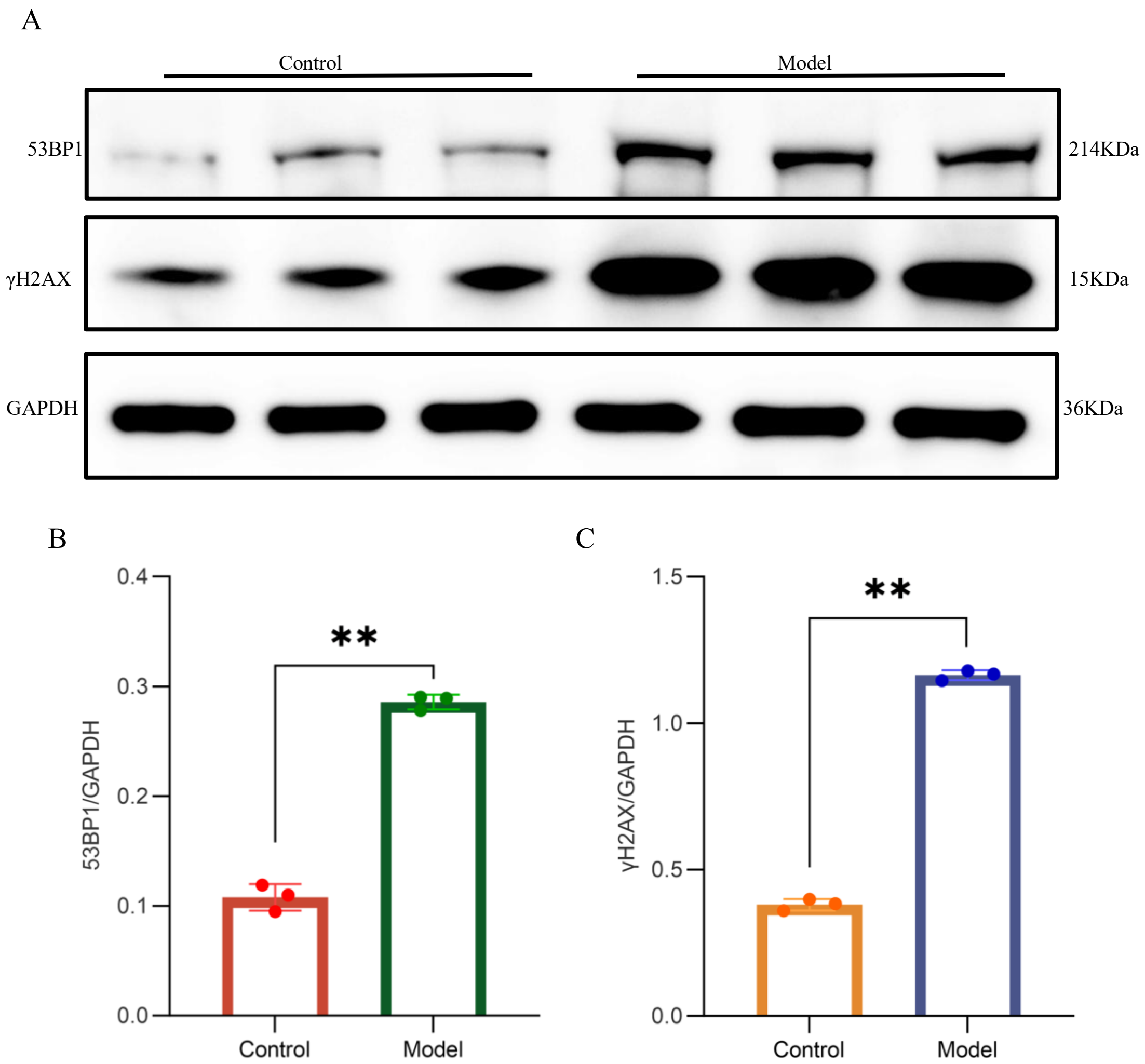

Figure 9: Increased DNA damage in myocardium of mice with heart failure; A-C: Protein quantitative analysis of 53BP1 and γH2AX; 53BP1: P53 binding protein 1; γH2AX: phosphorylated histone; n=3; compared with the control group,***P*<0.01

7.Dysregulation of Nesprin-1 interferes with histone methylation in heart failure

Histone methylation represents a critical modification that alters chromosome structure. Recent studies have highlighted the pivotal role of histone methylation modifications in myocardial hypertrophy, fibrosis, and cardiac function[34, 35]. Through immunofluorescence and Western blot analysis, we assessed the expression levels of H3K9me3 and H3K27me3 in mice from each experimental group. As depicted in the figures, the model group exhibited significantly increase expression levels of H3K9me3 and H3K27me3 compared to the control groups(Fig10A-D)(*P*<0.01). This result is consistent with the Western blot results(Fig11A-C)(*P*<0.01). These findings suggest that the histone methylation pattern disorder in the isoproterenol induced heart failure mouse model may be associated with the decreased level of Nesprin-1.

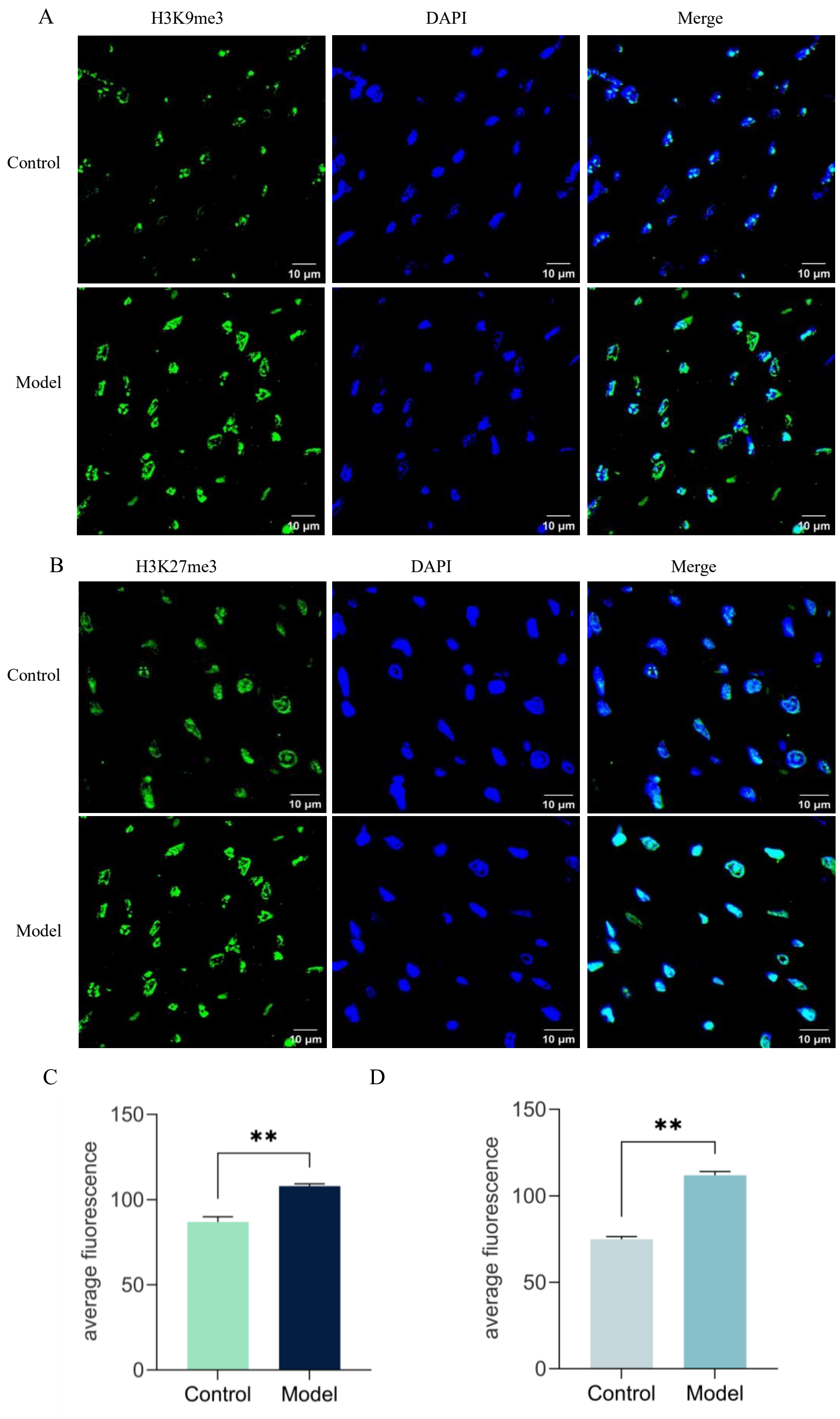

Figure 10: Changes in chromatin recombination in the myocardium of heart failure mice.A-D: Immunofluorescence detection of H3K9me3 and H3K27me3 expression; H3K9me3: trimethylation of histone H3 lysine 9; H3K27me3: trimethylation of histone H3 lysine 27; n=5; compared to the control group,***P*<0.01

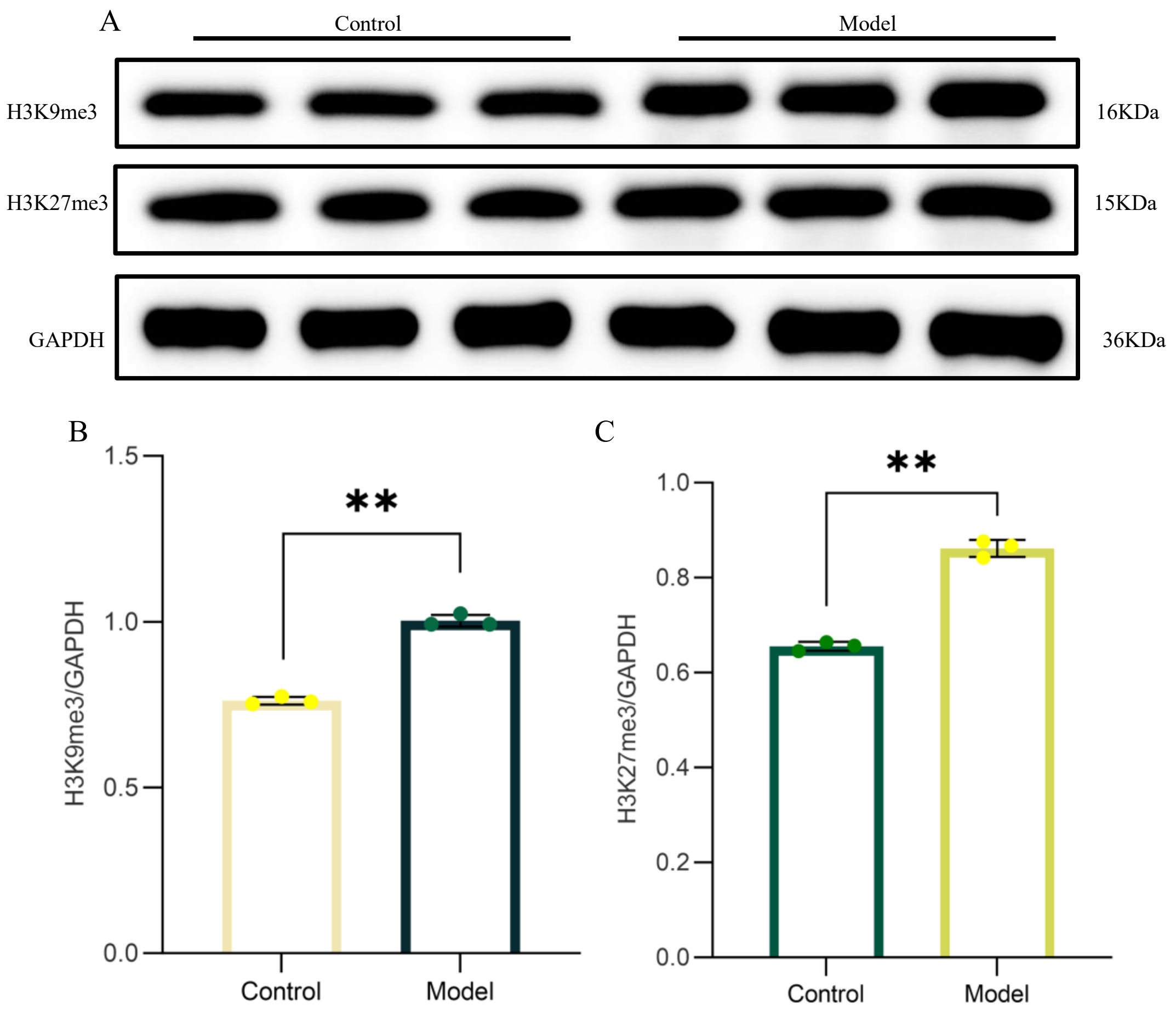

Figure 11: Changes in chromatin recombination in the myocardium of heart failure mice. A-C: Quantitative analysis of H3K9me3 and H3K27me3 proteins; H3K9me3: trimethylation of lysine 9 on histone H3; H3K27me3: trimethylation of lysine 27 on histone H3; n=3; compared with the control group,***P*<0.01

8.ERK pathway is activated in nuclear homeostasis abnormalities mediated by Nesprin-1 expression disorder and heart failure

The ERK pathway is recognized as a pivotal regulator in the development of myocardial hypertrophy. Studies have elucidated the formation of Nesprin-2/ERK complexes at sites of DNA damage in aortic smooth muscle cells, indicating a potential role of Nesprin proteins in modulating ERK signaling[36]. To investigate this phenomenon in the context of heart failure, we employed Western blot analysis to evaluate the expression levels of ERK in cardiac tissue samples from mice in each experimental group. Strikingly, our results revealed a notable increase in ERK expression in the model group compared to the control group, indicative of the activation of the ERK pathway in the cardiac tissue of the isoproterenol-induced heart failure mouse model(Fig12A-C)(P<0.01). Combined with the reduced expression of

Nesprin-1 in iprindel induced heart failure mice, we speculate that the reduced expression of Nesprin-1 may be associated with the activation of ERK pathway.

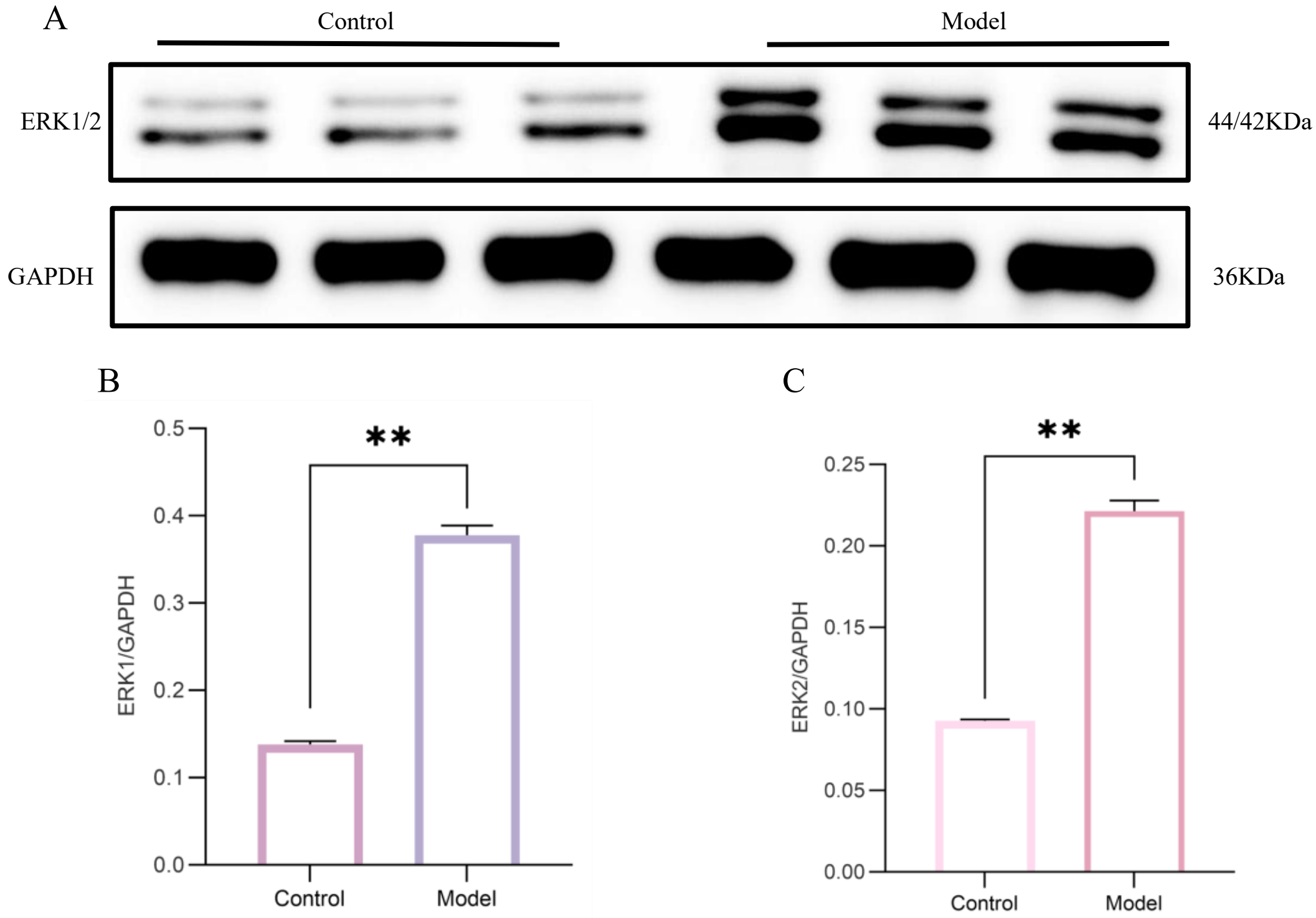

Figure 12: Downregulation of ERK1/2 expression in myocardium of mice with heart failure; A-C: Detection of ERK1/2 expression by protein immunoblotting; ERK: extracellular regulated protein kinase; n=3; compared with the control group,$^{**}P<0.01$

Discussion

Heart failure stands as an enduring challenge within global healthcare systems, presenting a formidable burden characterized by its pervasive prevalence, grim prognostic outlook, and profound detriment to patients' quality of life. Despite notable strides in HF management witnessed over recent decades, including the advent of neurohormonal blockade, device-based interventions, and cardiac transplantation, the persistently high rates of mortality and morbidity underscore the insufficiency of current therapeutic strategies[37-39]. Consequently, there exists an imperative mandate for sustained investigative efforts directed towards unraveling the intricate molecular mechanisms governing HF pathogenesis.

In this study, We induced myocardial hypertrophy in mice by intraperitoneal injection of ISO. Our findings indicate that the administration of ISO successfully induced myocardial hypertrophy, characterized by significant changes in heart weight, body weight, and WGA pathological staining results compared to the control group

mice, consistent with previous studies [40, 41]. Long-term stimulation by pathological factors can lead to persistent changes in cardiac structure, resulting in HF [42]. HE staining revealed that Nesprin-1 deficiency exacerbated myocardial structural disorder, vascular proliferation, and myocardial hemorrhage. Additionally, cardiac function tests confirmed the onset of HF in the mice, with significant changes in LVEDd, LVESd, EF, and FS. When myocardial cell volume and pressure overload occur, ventricular muscle cells release BNP, which exerts sodium-losing, diuretic, vasodilatory, and antihypertensive effects [43]. BNP is a crucial biomarker for the clinical diagnosis of HF . Western blotting detected a significant increase in BNP in the myocardial tissue of mice induced by isoproterenol, providing a more reliable basis for confirming the onset of HF in mice.

Nesprin are giant scaffolding proteins located at the NE that play essential roles in maintaining nuclear structure and regulating various cellular processes, including nuclear positioning, cytoskeletal organization, and gene expression[27, 44-48]. While previous research has primarily focused on the role of Nesprin in skeletal and muscular diseases, their involvement in cardiac pathology, particularly HF, remains poorly understood.In this study, we used the ISO-induced mouse heart failure model to initially assess the changes in Nesprin-1 expression and its potential links with nuclear structure, signaling pathways, and gene expression regulation. The ISO-induced HF mouse model established and validated in this study provides new insights into the pathophysiology of heart failure and the role of Nesprin-1 in cardiac function.

Our research has revealed that, compared to the control group, the expression of Nesprin-1 in the myocardium of ISO-induced HF mice is significantly reduced. This finding suggests that changes in Nesprin-1 expression may be linked to pathological remodeling processes associated with HF, such as myocardial hypertrophy and dysfunction. The downregulation of Nesprin-1 may be related to abnormalities in the morphology, nuclear localization, and structural integrity of myocardial cells, which have been preliminarily observed through histopathological examination. However, the specific molecular mechanisms still require further investigation. Additionally, the

dysregulation of Nesprin-1 expression may affect nuclear stability by disrupting the integrity of the nuclear membrane, potentially leading to cellular dysfunction. These findings suggest that, as a key component of the nuclear scaffold and cytoskeletal connection complex, changes in Nesprin-1 expression may be associated with nuclear instability and abnormal cell signaling.

The exact mechanisms underlying Nesprin-1-mediated pathogenesis in HF remain incompletely understood, but our study has shed light on several potential avenues for further investigation. our findings elucidate the multifaceted role of Nesprin-1 in HF pathogenesis, encompassing oxidative stress, electrical conduction abnormalities, and epigenetic dysregulation. First, Nesprin-1 may affect cardiac morphology through interactions with cytoskeletal elements and extracellular matrix proteins, thereby regulating the signaling pathways involved in fibroblast activation. However, the causal relationship between Nesprin-1 and these processes has not been directly demonstrated. For example, how Nesprin-1 affects fibroblast activation by influencing cardiac morphology through interactions with cytoskeletal components and extracellular matrix proteins.

Additionally, Nesprin appears to modulate oxidative stress and DNA damage responses in the heart. Reduced Nesprin expression is associated with heightened oxidative stress, as evidenced by alterations in oxidative stress markers such as malondialdehyde levels and decreased superoxide dismutase content. Increased expression of DNA damage repair proteins, such as γH2AX and 53BP1, further suggests Nesprin's role in preserving genomic integrity and protecting against oxidative stress-induced cardiac injury. Furthermore, the observed reduction in connexin 43 expression in Nesprin-deficient hearts highlights Nesprin's involvement in regulating cardiac conduction and electromechanical coupling. Diminished Cx43 expression may contribute to conduction abnormalities and arrhythmias commonly observed in HF, emphasizing Nesprin's role in regulating both structural and functional aspects of cardiac tissue. Lastly, alterations in histone methylation patterns, specifically changes in H3K9me3 and H3K27me3 levels in Nesprin-deficient hearts, suggest dysregulated epigenetic regulation in HF pathogenesis. These findings

underscore the intricate interplay between Nesprin and epigenetic regulatory mechanisms in maintaining cardiac homeostasis.

Activation of the ERK pathway has been implicated in various hypertrophic stimuli and is known to regulate gene expression, protein synthesis, and cell survival in cardiomyocytes[49-51]. In addition to the identified alterations in Nesprin expression and its implications in cardiac remodeling, our study elucidates the involvement of the ERK signaling pathwaymay be involved in mediating Nesprin-1-induced cardiac hypertrophy and remodeling in response to isoproterenol stimulation. Activation of the ERK pathway has been implicated in various cellular processes, including cell proliferation, differentiation, and survival[52-54]. Here, we demonstrate a significant increase in ERK expression in the cardiac tissue of mice subjected to isoproterenol-induced heart failure, suggesting the activation of the ERK pathway in response to cardiac stress. The observed upregulation of ERK signaling in Nesprin-deficient hearts highlights a potential interaction between Nesprin and the ERK pathway in regulating cardiac function. Previous studies have indicated the formation of Nesprin-2/ERK complexes at sites of DNA damage, implicating Nesprin proteins in the modulation of ERK signaling in other cellular contexts[36]. Our findings extend this understanding to the cardiac setting, where Nesprin deficiency may contribute to ERK pathway activation, thereby exacerbating myocardial injury and dysfunction. The activation of the ERK pathway in response to cardiac stressors, such as isoproterenol administration, suggests a role of ERK signaling in mediating pathological remodeling and maladaptive responses in the heart. Future studies aimed at elucidating the precise mechanisms underlying the crosstalk between Nesprin and the ERK pathway may provide further insights into the molecular mechanisms of heart failure pathogenesis.

The identification of Nesprin-1 as a potential regulator of HF pathogenesis opens up new avenues for therapeutic intervention. Targeting Nesprin-1 and its associated signaling pathways may offer novel strategies for preventing or attenuating adverse cardiac remodeling and dysfunction in HF[55-58]. Small molecule inhibitors, gene therapy approaches, or gene editing techniques could be explored to modulate

Nesprin-1 expression or activity selectively in the heart. Additionally, our study underscores the importance of further research into the molecular mechanisms governing nuclear envelope dynamics and their contribution to cardiovascular disease. In conclusion, our study provides compelling evidence implicating Nesprin-1 in the pathogenesis of HF induced by isoproterenol. By elucidating the role of Nesprin-1 in cardiac remodeling, electrical conduction, we have shed light on novel molecular mechanisms underlying HF pathophysiology. Further investigation into the functional significance of Nesprin-1 and its interactions with key signaling pathways may yield valuable insights into disease progression and facilitate the development of targeted therapies for HF. Ultimately, our findings contribute to a deeper understanding of the complex molecular networks driving HF and offer hope for improved outcomes for patients afflicted with this devastating condition. This comprehensive discussion highlights the significance of our findings and provides a roadmap for future research endeavors aimed at unraveling the intricate molecular mechanisms of HF pathogenesis and identifying innovative therapeutic strategies to combat this disease.

However, this study still has some limitations. Firstly, the lack of specific inhibitors for Nesprin-1, along with the high mortality and lengthy construction period of Nesprin gene knockout mice, prevented us from directly establishing a causal relationship between Nesprin-1, oxidative stress, reduced Cx43 expression, increased DNA damage, and chromatin recombination disorders. Secondly, while we observed that decreased Nesprin-1 expression is associated with various pathological features, such as myocardial structural disorder, changes in oxidative stress markers, and increased expression of DNA damage repair proteins, these findings are only correlational. The specific molecular mechanisms require further functional experiments to confirm. Additionally, this study primarily used ISO-induced HF mouse models. Future studies should validate the role of Nesprin-1 in other HF models, such as those induced by stress overload or myocardial infarction, to enhance the generalizability of the research results. Lastly, regarding the interaction between Nesprin-1 and the ERK signaling pathway and epigenetic regulation (such as histone methylation), this study only provides preliminary clues. The specific mechanisms

need to be elucidated through more in-depth molecular and cellular experiments. In future research, we plan to construct a Nesprin-1-specific knockout model, combined with multi-omics analysis and functional validation, to further uncover its exact role in HF.

Declarations

Ethics approval and consent to participate

Not applicable.

Consent for publication

The manuscript was obtained from all authors for consent for submission and publication

Competing interests

The authors declare no competing interests

Funding

This research was funded by the Hubei Provincial Natural Science Foundation Project (Joint Fund Project, Project No.: 2024AFD143).The funders had no role in study design, data collection and analysis, decision to publish, or preparation of the manuscript.

Authors' contributions

Qin Qin and Dong Cengyuan completed the experiment and wrote the manuscript; Zhou Ziyi participated in some of the experiments and manuscript writing; Liu Yangyuanzhi was involved in the article revision; Professor Cao Chunyu provided guidance on the experimental design; Doctor Teng Lin guided the entire experimental process and manuscript writing.

Availability of Data and Material

The data that support the findings of this study are available from the corresponding author upon reasonable request.

Acknowledgements

I would like to thank CJH for their technical assistance in this experiment. And I am very grateful for the funding provided by the Provincial Natural Science Foundation for this experiment.

Reference

[1] Tanai E, Frantz S. Pathophysiology of Heart Failure [J]. Compr Physiol, 2015, 6(1): 187-214.

[2] McDonagh T A, Metra M, Adamo M, et al. 2021 ESC Guidelines for the diagnosis and treatment of acute and chronic heart failure: Developed by the Task Force for the diagnosis and treatment of acute and chronic heart failure of the European Society of Cardiology (ESC) With the special contribution of the Heart Failure Association (HFA) of the ESC [J]. Rev Esp Cardiol (Engl Ed), 2022, 75(6): 523.

[3] Bozkurt B, Coats A J S, Tsutsui H, et al. Universal Definition and Classification of Heart Failure [J]. Journal of Cardiac Failure, 2021, 27(4): 387-413.

[4] Kemp C D, Conte J V. The pathophysiology of heart failure [J]. Cardiovasc Pathol, 2012, 21(5): 365-71.

[5] Ghionzoli N, Gentile F, Del Franco A M, et al. Current and emerging drug targets in heart failure treatment [J]. Heart Failure Reviews, 2021, 27(4): 1119-36.

[6] Savarese G, Becher P M, Lund L H, et al. Global burden of heart failure: a comprehensive and updated review of epidemiology [J]. Cardiovasc Res, 2023, 118(17): 3272-87.

[7] Shahim B, Kapelios C J, Savarese G, et al. Global Public Health Burden of Heart Failure: An Updated Review [J]. Card Fail Rev, 2023, 9: e11.

[8] Borovac J A, D'Amario D, Bozic J, et al. Sympathetic nervous system activation and heart failure: Current state of evidence and the pathophysiology in the light of novel biomarkers [J]. World J Cardiol, 2020, 12(8): 373-408.

[9] Bencivenga L, Palaia M E, Sepe I, et al. Why Do We Not Assess Sympathetic Nervous System Activity in Heart Failure Management: Might GRK2 Serve as a New Biomarker? [J]. Cells, 2021, 10(2).

[10] Wang J, Dai M, Cao Q, et al. Carotid baroreceptor stimulation suppresses ventricular fibrillation in canines with chronic heart failure [J]. Basic Research in Cardiology, 2019, 114(6).

[11] Huang Y, Liu Y-W, Pan H-Z, et al. Transthoracic Pulmonary Artery Denervation for Pulmonary Arterial Hypertension [J]. Arteriosclerosis, Thrombosis, and Vascular Biology, 2019, 39(4): 704-18.

[12] Triposkiadis F, Briasoulis A, Kitai T, et al. The sympathetic nervous system in heart failure revisited [J]. Heart Fail Rev, 2024, 29(2): 355-65.

[13] Grassi G, Mancia G, Esler M. Central and peripheral sympathetic activation in heart failure [J]. Cardiovasc Res, 2022, 118(8): 1857-71.

[14] Gronda E, Dusi V, D'Elia E, et al. Sympathetic activation in heart failure() [J]. Eur Heart J Suppl, 2022, 24(Suppl E): E4-E11.

[15] Munoz-Durango N, Fuentes C A, Castillo A E, et al. Role of the Renin-Angiotensin-Aldosterone System beyond Blood Pressure Regulation: Molecular and Cellular Mechanisms Involved in End-Organ Damage during Arterial Hypertension [J]. Int J Mol Sci, 2016, 17(7).

[16] Pugliese N R, Masi S, Taddei S. The renin-angiotensin-aldosterone system: a crossroad from arterial hypertension to heart failure [J]. Heart Fail Rev, 2020, 25(1): 31-42.

[17] Ferreira N S, Tostes R C, Paradis P, et al. Aldosterone, Inflammation, Immune System, and Hypertension [J]. Am J Hypertens, 2021, 34(1): 15-27.

[18] Weir M R, Dzau V J. The renin-angiotensin-aldosterone system: a specific target for hypertension management [J]. Am J Hypertens, 1999, 12(12 Pt 3): 205S-13S.

[19] Chen Y, He Q, Mo D-C, et al. The angiotensin receptor and neprilysin inhibitor, LCZ696, in heart failure: A meta-analysis of randomized controlled trials [J]. Medicine, 2022, 101(41).

[20] Heidenreich PA B B, Aguilar D,et al. Correction to: 2022 AHA/ACC/HFSA Guideline for the Management of Heart Failure: A Report of the American College of Cardiology/American Heart Association Joint Committee on Clinical Practice Guidelines [J]. Circulation, 2023, 147(14): e263-e421.

[21] Lachowska K, Gruchala M, Narkiewicz K, et al. Sympathetic Activation in Chronic Heart Failure: Potential Benefits of Interventional Therapies [J]. Curr Hypertens Rep, 2016, 18(7): 51.

[22] von Lueder T G, Kotecha D, Atar D, et al. Neurohormonal Blockade in Heart Failure [J]. Card Fail Rev, 2017, 3(1): 19-24.

[23] Ghionzoli N, Gentile F, Del Franco A M, et al. Current and emerging drug targets in heart failure treatment [J]. Heart Fail Rev, 2022, 27(4): 1119-36.

[24] Crisp M, Liu Q, Roux K, et al. Coupling of the nucleus and cytoplasm: Role of the LINC complex [J]. The Journal of Cell Biology, 2006, 172(1): 41-53.

[25] Meinke P, Nguyen T D, Wehnert M S. The LINC complex and human disease [J]. Biochem Soc Trans, 2011, 39(6): 1693-7.

[26] De Silva S, Catibog N, Shanahan C, et al. Investigating novel roles for nesprin-1 and the LINC complex in Dilated Cardiomyopathy [J]. Cardiovascular Research, 2022, 118(Supplement_1).

[27] Zhou C, Li C, Zhou B, et al. Novel nesprin-1 mutations associated with dilated cardiomyopathy cause nuclear envelope disruption and defects in myogenesis [J]. Hum Mol Genet, 2017, 26(12): 2258-76.

[28] Shanahan CM W P, Metcalfe JC. Isolation of gene markers of differentiated and proliferating vascular smooth muscle cells [J]. Circ Res, 1993, 73(1): 193-204.

[29] Chen Z, Ren Z, Mei W, et al. A novel SYNE1 gene mutation in a Chinese family of Emery-Dreifuss muscular dystrophy-like [J]. BMC Medical Genetics, 2017, 18(1).

[30] Stroud M J, Banerjee I, Veevers J, et al. Linker of Nucleoskeleton and Cytoskeleton Complex Proteins in Cardiac Structure, Function, and Disease [J]. Circulation Research, 2014, 114(3): 538-48.

[31] Lindenboim L, Grozki D, Amsalem-Zafran A R, et al. Apoptotic stress induces Bax-dependent, caspase-independent redistribution of LINC complex nesprins [J]. Cell Death Discov, 2020, 6(1): 90.

[32] Boengler K, Rohrbach S, Weissmann N, et al. Importance of Cx43 for Right Ventricular Function [J]. Int J Mol Sci, 2021, 22(3).

[33] Sur-Erdem I, Hussain M S, Asif M, et al. Nesprin-1 impact on tumorigenic cell phenotypes [J]. Mol Biol Rep, 2020, 47(2): 921-34.

[34] Yao L, He F, Zhao Q, et al. Spatial Multiplexed Protein Profiling of Cardiac Ischemia-Reperfusion Injury [J]. Circ Res, 2023, 133(1): 86-103.

[35] Li J, Sha Z, Zhu X, et al. Targeting miR-30d reverses pathological cardiac hypertrophy [J]. EBioMedicine, 2022, 81: 104108.

[36] Warren D T, Tajsic T, Porter L J, et al. Nesprin-2-dependent ERK1/2 compartmentalisation regulates the DNA damage response in vascular smooth muscle cell ageing [J]. Cell Death Differ, 2015, 22(9): 1540-50.

[37] Collaborators G B D R F. Global burden and strength of evidence for 88 risk factors in 204 countries and 811 subnational locations, 1990-2021: a systematic analysis for the Global Burden of Disease Study 2021 [J]. Lancet, 2024, 403(10440): 2162-203.

[38] Savarese G, Lund L H. Global Public Health Burden of Heart Failure [J]. Card Fail Rev, 2017, 3(1): 7-11.

[39] Sapna F, Raveena F, Chandio M, et al. Advancements in Heart Failure Management: A Comprehensive Narrative Review of Emerging Therapies [J]. Cureus, 2023, 15(10): e46486.

[40] Aimo A, Gaggin H K, Barison A, et al. Imaging, Biomarker, and Clinical Predictors of Cardiac Remodeling in Heart Failure With Reduced Ejection Fraction [J]. JACC Heart Fail, 2019, 7(9): 782-94.

[41] Goetze J P, Bruneau B G, Ramos H R, et al. Cardiac natriuretic peptides [J]. Nat Rev Cardiol, 2020, 17(11): 698-717.

[42] Salah K, Stienen S, Pinto Y M, et al. Prognosis and NT-proBNP in heart failure patients with preserved versus reduced ejection fraction [J]. Heart, 2019, 105(15): 1182-9.

[43] Apel E D, Lewis R M, Grady R M, et al. Syne-1, a dystrophin- and Klarsicht-related protein associated with synaptic nuclei at the neuromuscular junction [J]. J Biol Chem, 2000, 275(41): 31986-95.

[44] De Silva S, Fan Z, Kang B, et al. Nesprin-1: novel regulator of striated muscle nuclear positioning and mechanotransduction [J]. Biochem Soc Trans, 2023, 51(3): 1331-45.

[45] Leong E L, Khaing N T, Cadot B, et al. Nesprin-1 LINC complexes recruit microtubule cytoskeleton proteins and drive pathology in Lmna-mutant striated muscle [J]. Hum Mol Genet, 2023, 32(2): 177-91.

[46] Zhang Q, Ragnauth C D, Skepper J N, et al. Nesprin-2 is a multi-isomeric protein that binds lamin and emerin at the nuclear envelope and forms a subcellular network in skeletal muscle [J]. J Cell Sci, 2005, 118(Pt 4): 673-87.

[47] Rajgor D, Shanahan C M. Nesprins: from the nuclear envelope and beyond [J]. Expert Rev Mol Med, 2013, 15: e5.

[48] Zi-Yi Z, Qin Q, Fei Z, et al. Nesprin proteins: bridging nuclear envelope dynamics to muscular dysfunction [J]. Cell Commun Signal, 2024, 22(1): 208.

[49] Gallo S, Vitacolonna A, Bonzano A, et al. ERK: A Key Player in the Pathophysiology of Cardiac Hypertrophy [J]. Int J Mol Sci, 2019, 20(9).

[50] Mutlak M, Kehat I. Extracellular signal-regulated kinases 1/2 as regulators of cardiac hypertrophy [J]. Front Pharmacol, 2015, 6: 149.

[51] Ramos-Kuri M, Meka S H, Salamanca-Buentello F, et al. Molecules linked to Ras signaling as therapeutic targets in cardiac pathologies [J]. Biol Res, 2021, 54(1): 23.

[52] Sugiura R, Satoh R, Takasaki T. ERK: A Double-Edged Sword in Cancer. ERK-Dependent Apoptosis as a Potential Therapeutic Strategy for Cancer [J]. Cells, 2021, 10(10).

[53] Deschênes-Simard X, Malleshaiah M, Ferbeyre G. Extracellular Signal-Regulated Kinases: One Pathway, Multiple Fates [J]. Cancers (Basel), 2023, 16(1).

[54] Wu J, Chen Y, Li R, et al. Synergistic anticancer effect by targeting CDK2 and EGFR-ERK signaling [J]. J Cell Biol, 2024, 223(1).

[55] Li Y L, Cheng X N, Lu T, et al. Syne2b/Nesprin-2 Is Required for Actin Organization and Epithelial Integrity During Epiboly Movement in Zebrafish [J]. Front Cell Dev Biol, 2021, 9: 671887.

[56] Davidson P M, Battistella A, Dejardin T, et al. Nesprin-2 accumulates at the front of the nucleus during confined cell migration [J]. EMBO Rep, 2020, 21(7): e49910.
[57] Chen S N, Sbaizero O, Taylor M R G, et al. Lamin A/C Cardiomyopathy: Implications for Treatment [J]. Curr Cardiol Rep, 2019, 21(12): 160.
[58] Nicolas H A, Hua K, Quigley H, et al. A CRISPR/Cas9 zebrafish lamin A/C mutant model of muscular laminopathy [J]. Dev Dyn, 2022, 251(4): 645-61.

Manuscript

Click here to access/download;Manuscript;renamed_1ff35.docx

Exploring the Role of Nesprin-1 in Isoproterenol-Induced Heart Failure Insights into Pathogenesis and Therapeutic Implications

Qin Qin[1,2#] , Dong Cengyuan[1,2#] , Zhou Zi-yi[1,2] ,Liu Yangyuanzhi[1,2], Cao Chunyu [2,4], TENG Lin[1,3*]

1. Department of Cardiology, Yichang Central People's Hospital/The First Clinical Medical College, Three Gorges University, Yichang 443003, Hubei, People's Republic of China

2. School of Basic Medicine, China Three Gorges University, Yichang 443000, Hubei, People's Republic of China

3. King’s College London British Heart Foundation Centre of Research Excellence, School of Cardiovascular and Metabolic Medicine & Sciences, London SE5 9NU, U.K

4. College of Basic Medical Sciences, Hubei Key Laboratory of Tumor Microencironment and immunotherapy, China Three Gorges University, Yichang 443000, Hubei, People's Republic of China

# Qin Qin and Dong Cengyuan have equally to this work.

* Correspondence to: TENG Lin, MD, Department of Cardiology, Yichang Central People's, NO,183 Yiling Road, Yichang 443003, Hubei, People's Republic of China. E-mail address: tenglin@ctgu.edu.cn

Objective: In this study, we used an isoprenaline-induced heart failure model to investigate the role of Nesprin-1 in the development and progression of heart failure and to clarify the underlying molecular mechanisms.

Methods: A mouse model of heart failure was induced by daily intraperitoneal injection of isoproterenol (5 mg/kg/day) for 24 consecutive days. The body weight of mice was monitored daily. The diastolic left ventricular diameter (LVDd), systolic left ventricular diameter (LVDs), ejection fraction (EF) and short axis shortening rate (FS) of mice were evaluated by echocardiography of small animal hearts. The heart failure model was validated by gross cardiac observation, WGA staining, and Western blot analysis of B-type Natriuretic Peptide (BNP) expression. Hematoxylin-Eosin staining of heart tissue was performed to assess myocardial structure, cell morphology and pathological changes. Quantitative determination of malondialdehyde (MDA) and superoxide dismutase (SOD) expression was performed to evaluate oxidative stress levels. The expression of Nesprin-1 and connexin 43 (Cx43) was detected by immunofluorescence staining. Immunofluorescence staining and Western blot were used to detect the effects of Nesprin-1 expression changes on p53-binding protein 1 (53BP1), phosphorylated histone H2AX (γH2AX), H3 lysine 9 trimethylation (H3K9me3), H3 lysine 27 trimethylation (H3K27me3) expression.

In addition, we used Western blot to analyze the expression of the extracellular regulatory protein kinase (ERK) pathway.

Results: Compared with the control group, mice in the isoproterenol-induced heart failure model group exhibited significant increases in both body weight and heart weight ($P<0.01$), enlargement of cardiomyocyte cross-sectional area ($P<0.01$), markedly elevated BNP expression ($P<0.01$). The echocardiographic results showed that the LVDd and LVDs in diastolic phase were significantly increased, and EF and FS were significantly decreased in mice with heart failure($P<0.01$). Histopathological analysis demonstrated disordered myocardial structure, vascular proliferation, and myocardial hemorrhage in model group mice. The expression of Nesprin-1 was significantly downregulated in the model group ($P<0.01$), accompanied by abnormal nuclear envelope localization that resulted in distorted nuclear morphology of cardiomyocytes, including increased nuclear area and perimeter, elevated nuclear aspect ratio ($P<0.01$) , and decreased nuclear circularity($P<0.01$). Additionally, the model group showed significantly increased levels of the oxidative stress marker MDA coupled with decreased SOD activity ($P<0.05$), reduced Cx43 expression ($P<0.01$), upregulated expression of DNA damage markers (γH2AX and 53BP1)($P<0.01$), significantly elevated levels of H3K9me3 and H3K27me3 ($P<0.01$), and markedly enhanced activation of the ERK signaling pathway ($P< 0.01$).

Conclusion: This study revealed that downregulation of Nesprin-1 may serve as a crucial driver in the progression of heart failure. In the isoproterenol-induced murine heart failure model, reduced Nesprin-1 expression leads to multiple pathological alterations, including nuclear homeostasis imbalance, decreased Cx43 expression, elevated oxidative stress, accumulated DNA damage, and disordered epigenetic regulation. The underlying mechanism may involve activation of the ERK signaling pathway.

Keywords: Heart failure, Nesprin-1, Isoproterenol, Cardiac remodeling, Dysfunction, Epigenetic regulation, DNA damage

Introduction

Heart failure (HF), or cardiac failure, signifies the heart's diminished capacity to efficiently pump blood, resulting in venous congestion and inadequate arterial perfusion due to compromised systolic and/or diastolic functions[1-4]. This condition represents the terminal phase of heart disease and stands as a leading cause of cardiovascular-related mortality worldwide. HF affects approximately 1% to 2% of the global population, with a notably higher prevalence among the elderly[5-7]. Sympathetic nervous system (SNS) overactivity is a hallmark of HF, correlating with disease severity and prognosis. Under normal circumstances, the cardiac sympathetic nervous system enhances cardiac output, gene transcription, and metabolism by releasing neurotransmitters and engaging β-adrenergic receptors (β-AR)[8, 9]. However, excessive SNS activation detrimentally affects the cardiovascular system, potentially precipitating or exacerbating HF[10, 11]. This overdrive leads to heightened heart rate and contractility, inducing a sustained high-load state that depletes cardiac energy reserves, impeding cardiac function. Furthermore, it promotes adverse structural cardiac changes such as ventricular hypertrophy and atrial dilation, diminishing cardiac pump efficiency[12-14]. Increased arterial vasoconstriction further strains the heart by elevating vascular tension. Excessive sympathetic stimulation also incites inflammation and oxidative stress, damaging myocardial cells and hastening disease progression. Disruption of neuroendocrine system balance, including the renin-angiotensin-aldosterone system, can exacerbate fluid retention and increase blood volume, exacerbating cardiac workload[15-18]. Current HF management strategies primarily target the sympathetic nervous system with medications like β-blockers, angiotensin-converting enzyme inhibitors, angiotensin II receptor blockers, and aldosterone receptor antagonists. Despite these interventions, HF incidence, readmission rates, and mortality remain high, underscoring the need for novel therapeutic approaches[19-23].

Traditionally, HF research focused on intracellular factors, neglecting the nuclear envelope's role. The nuclear envelope (NE) is pivotal, spatially segregating nuclear genetic material and regulating macromolecular transport[24]. The Linker of

Nucleoskeleton and Cytoskeleton (LINC) complex, anchored to the NE, includes Nesprin proteins, which bridge the nuclear and cytoskeletal proteins, contributing to cellular structure and function. Nesprin mutations are implicated in various diseases, including Dilated Cardiomyopathy (DCM) and Emery-Dreifuss Muscular Dystrophy (EDMD), highlighting their importance in muscle physiology[25-27]. Nesprin family proteins, integral to the NE, include Nesprin-1 and Nesprin-2, which interact with actin, intermediate filaments, and the nuclear envelope, influencing cell signaling, morphology, and mechanical properties[28]. These proteins regulate gene expression, chromatin structure, and epigenetic modifications, impacting cell function and pathology. Mutations in Nesprin-1 and Nesprin-2 are associated with abnormal nuclear morphology, positioning, and chromatin organization, contributing to DCM and EDMD. Nesprin alterations also affect intercalated disc protein localization, leading to conduction defects in Nesprin-associated cardiomyopathies[27, 29, 30].

Given this background, we hypothesize that the dysregulation of Nesprin-1 expression disrupts the structural connection between the nucleus and the cytoskeleton, thereby impairing nuclear homeostasis, interfering with signal transduction and causing abnormal transcription regulation, which ultimately leads to myocardial remodeling and contractile dysfunction, and then heart failure. Our study aims to elucidate Nesprin-1's role and molecular mechanisms in HF, providing insights for diagnosis and treatment. Understanding Nesprin-1's function may reveal therapeutic targets, guiding future HF therapies.

Materials and methods

Drugs and Chemicals

MedChemExpress provided isoproterenol (HY-B0468) and isoflurane, while Biosharp supplied 4% paraformaldehyde (BP003), hematoxylin-eosin staining (BP019), and other staining-related reagents. Sinopharm Chemical Reagent offered basic reagents such as paraffin (69019361) and xylene (10023418). Antibodies were mainly sourced from Boster, including Cx43 antibody (A00599), BNP antibody (M01186-3), 53BP1 antibody (BM5099), γH2AX antibody (BM4148), H3K9me3 antibody (BM5079), H3K27me3 antibody (BM4341), and ERK antibody (BM4156).

Protein-related reagents were primarily obtained from Beyotime, including RIPA lysis buffer (P0013B) and BCA protein assay kit (P0010). Additionally, Amresco provided electrophoresis reagents such as TEMED (Amresc00761) and acrylamide (Exp2016109), while Jackson supplied HRP-labeled secondary antibodies, specifically HRP-conjugated goat anti-rabbit (BA1054), HRP-conjugated rabbit anti-goat (BA1060), HRP-conjugated goat anti-mouse (BA1051), and HRP-conjugated goat anti-rat (BA1058). Other essential reagents included WGA stock solution (BBL-0351) from Sigma-Aldrich, DAPI stock solution (C1002) from Solarbio, and cytoplasmic/nuclear protein extraction kit (KGP150) from Nanjing KeyGen Biotech.

Establishment of Isoproterenol-Induced Heart Failure Model in Mice

The male C57BL/6 mice weighing 20-22g were purchased from the Animal Experimental Center of Three Gorges University. All mice were housed in specific pathogen-free (SPF) animal facilities at Three Gorges University (Yichang, Hubei, China). The experimental animal quality certification number is 42010200009428. The experimental animal use permit number is 00305510. Twenty-four C57BL/6 mice were randomly divided into two groups: model group and control group. In the model group, mice were administered intraperitoneal injections of isoproterenol (ISO) at a dosage of 5mg/kg. In the control group, mice were administered intraperitoneal injections of an equivalent volume of medical-grade physiological saline. Two groups received intraperitoneal injections continuously for 24 days.

Cardiac Function Assessment

Mice were anesthetized with isoflurane, and after achieving stable anesthesia, the hair in the chest area was removed. The mice were then placed in a supine position and fixed on an ultrasound examination table. Ultrasound coupling agent was applied to the chest area. Using small animal ultrasound, M-mode images of the long axis and short axis of the mouse heart were obtained. Parameters including ejection fraction (EF), fractional shortening (FS), left ventricular end-diastolic diameter (LVEDd), and left ventricular end-systolic diameter (LVESd) were measured and calculated to assess cardiac function in mice. Statistical analysis was performed to analyze the cardiac

function of mice.

Measurement of Body and Heart Weight

Body weight of the mice was measured before each dose.At least 24 hours after the last dose the mice were anesthetized, and their chests were swiftly opened using surgical scissors and forceps to extract the hearts. The hearts were rinsed in pre-cooled physiological saline, and then scissors were used to trim the blood vessels and connective tissues surrounding the heart. The hearts were then rinsed again with fresh physiological saline and dried with filter paper. Gross images of the hearts were captured, and the total heart weight was measured. Some samples were frozen in dry ice and stored in-80℃ refrigerator, while others were fixed in 4% polyformaldehyde fixative for easy observation of histological morphology.

Pathological Section Staining

HE staining: After dehydration, embedding, and sectioning of tissue fixed with 4% polyformaldehyde, the paraffin sections are dewaxed to water. Stain with hematoxylin for 4 minutes, followed by 2-minute rinsing with tap water to remove excess stain. The sections are then differentiated with 0.8% hydrochloric acid alcohol for 2 seconds, rinsed again with tap water, immersed in eosin solution for 20 seconds, adjusted with 95% ethanol for 5 seconds, and dehydrated with anhydrous ethanol for 2 minutes. Finally, the sections are sealed with a transparent environmental agent and observed under a microscope.

WGA staining: The paraffin sections were sequentially dewaxed with eco-friendly degreasing agents (1), (2), and (3) for 10 minutes each, followed by 5-minute immersion in anhydrous ethanol, 95% ethanol, and 75% ethanol respectively. The sections underwent three 3-minute rinses with distilled water. Antigen retrieval was performed by high-pressure treatment of the sections in boiling citric acid (pH6.0) solution for 2 minutes. After cooling, the sections were immersed in distilled water for three 5-minute washes. A circular mark was made on the section using a histological pen, then it was immersed in TBST buffer. A 1:200 diluted WGA working solution (50 uL/section) was added and incubated under light protection for 30 minutes, followed by three 5-minute TBST washes. After removing TBST, a 1:500 diluted

DAPI working solution (50 uL/section) was applied to stain nuclei for 5 minutes. Following TBST rinsing, the sections were mounted with anti-fluorescence quenching medium and stored at 4℃ under light protection. Finally, microscopic examination and image analysis were conducted.

The biochemical kits detects the oxidative stress indicators

Sample Preparation: Myocardial tissue was mixed with PBS buffer at ratios of 1:10 (for MDA detection) and 1:9 (for SOD detection), homogenized, and centrifuged at 10,000 g for 15 min at 4 ℃. The supernatant was collected for subsequent analysis.

Reagent Preparation: MDA detection working solution (including probe working solution and standard) and SOD detection working solution (including enzyme reaction solution) were prepared according to the kit instructions.

MDA Measurement: The sample was mixed with the detection working solution, incubated at 95 ℃ for 40 min, cooled on ice for 5 min, and centrifuged at 10,000 g for 10 min. The supernatant was measured at 532 nm absorbance.

Calculation formula:

MDA(umol/gprot)=[(A_{sample}-A_{blank}) /(A_{standard}-A_{blank})]×C_{standard}×dilution factor / protein concentration

SOD Measurement: 20 uL of sample was added, incubated at 37 ℃ for 20 min, and measured at 450 nm absorbance.

Calculation formulas:

SOD inhibition rate (%) = [(ΔA_{control} - ΔA_{sample}) / ΔA_{control}] × 100%

SOD activity (U/mg prot) = Inhibition rate / (100% - Inhibition rate) × 12 / protein concentration

(Note: In the formulas, C_{standard} is the standard concentration, ΔA is the absorbance difference, and 12 is the conversion factor for reaction volume/sample volume.)

Immunofluorescence Staining Protocol for Mouse Cardiac Tissue

Initially, tissues fixed with 4% paraformaldehyde were processed through dehydration,

embedding, and sectioning, followed by dewaxing the paraffin sections to water. Antigen retrieval was then performed by incubating the sections in preheated 95° C sodium citrate buffer (pH 6.0) under high-pressure heating for 10 minutes. After natural cooling, the sections were washed three times with phosphate-buffered saline (PBS), 3 minutes each. Excess moisture was removed using absorbent paper, and the tissue areas were circled with an immunohistochemical pen before applying diluted normal goat serum for 30-minute blocking at room temperature. After blocking, excess liquid was removed (without washing), and diluted primary antibody was directly applied for overnight incubation at 4 ℃ in a humidified chamber. The following day, sections were thoroughly washed with PBS (⩾3 times, 3 minutes each), dried, and treated with diluted fluorescent secondary antibody for 1-hour incubation at 25 ℃ in a humidified chamber, followed by four additional PBS washes (3 minutes each). For nuclear staining, DAPI was applied under light-protected conditions for 5 minutes, followed by four PBS washes (5 minutes each) to remove excess dye. Finally, sections were dried and mounted with anti-fade mounting medium for observation and image acquisition under a fluorescence microscope.

Western Blot Experimental Procedure

Protein extraction was first performed by mincing tissue samples in a 2mL homogenizer, followed by adding 400 uL lysis buffer containing PMSF for ice-cold homogenization with repeated processing at 5-minute intervals until complete tissue disruption was achieved. After 30 minutes of lysis, the lysate was centrifuged at 12,000 rpm for 5 minutes at 4 ℃, and the supernatant was aliquoted and stored at -20 ℃. Protein concentration was determined using the BCA method: samples were diluted 1:19 with PBS, and a standard curve (0.2-1mg/mL BSA) was prepared and loaded onto a 96-well plate alongside samples (standards in duplicate, samples in triplicate). After adding BCA working solution, the plate was incubated at 37° C for 30 minutes protected from light, and absorbance at 568 nm was measured to calculate protein concentration. Protein samples were mixed with 5×loading buffer and denatured by boiling for 10 minutes before storage.

For electrophoresis, separating and stacking gels were prepared, and 40ug total protein was loaded per well. Initial electrophoresis was performed at 80V until samples entered the separating gel, then switched to 120V for approximately 1.5 hours until the bromophenol blue reached the gel bottom. Prior to transfer, PVDF membranes were cut and activated with methanol, and the transfer sandwich was assembled in the order of "negative plate-fiber pad-filter paper-gel-membrane-filter paper-fiber pad-positive plate" for constant current transfer. After transfer, membranes were blocked with 5% skim milk in TBST (2% BSA for phosphoproteins) at room temperature for 2 hours.

Following overnight primary antibody incubation at 4 ℃, membranes were washed 6 times with TBST, then incubated with HRP-conjugated secondary antibody at 37 ℃ for 2 hours, followed by 5 additional TBST washes. For ECL detection, enhanced solution and peroxidase solution were mixed in equal proportions and applied to membranes. After several minutes of reaction, X-ray films were exposed and processed through development and fixation to obtain results. Throughout the procedure, care was taken to maintain samples at low temperatures, ensure bubble-free transfer, and keep membranes moist during antibody incubations.

Statistical analysis

All quantitative data were expressed as mean ± standard deviation (X±SD). The comparison between the two groups was evaluated using the t-test. P value <0.05 indicated statistically significant differences. The data were analyzed using GraphPad Prism 10.0 software.

3.Result

1.Establishment and Validation of Isoproterenol-Induced Heart Failure Mouse Model

Echocardiographic evaluation revealed notable alterations in cardiac parameters across the two experimental groups(Fig1A-B). Mice in the model group displayed significant increases in left ventricular end-diastolic diameter (LVEDd) and left ventricular end-systolic diameter (LVESd) compared to the control groups(Fig1C-D)(P<0.01). Conversely, ejection fraction (EF) and fractional

shortening (FS) were markedly reduced in the model group(Fig1E-F)(P<0.01). These findings underscore the effectiveness of the isoproterenol-induced myocardial hypertrophy model in inducing structural and functional changes in cardiac function.

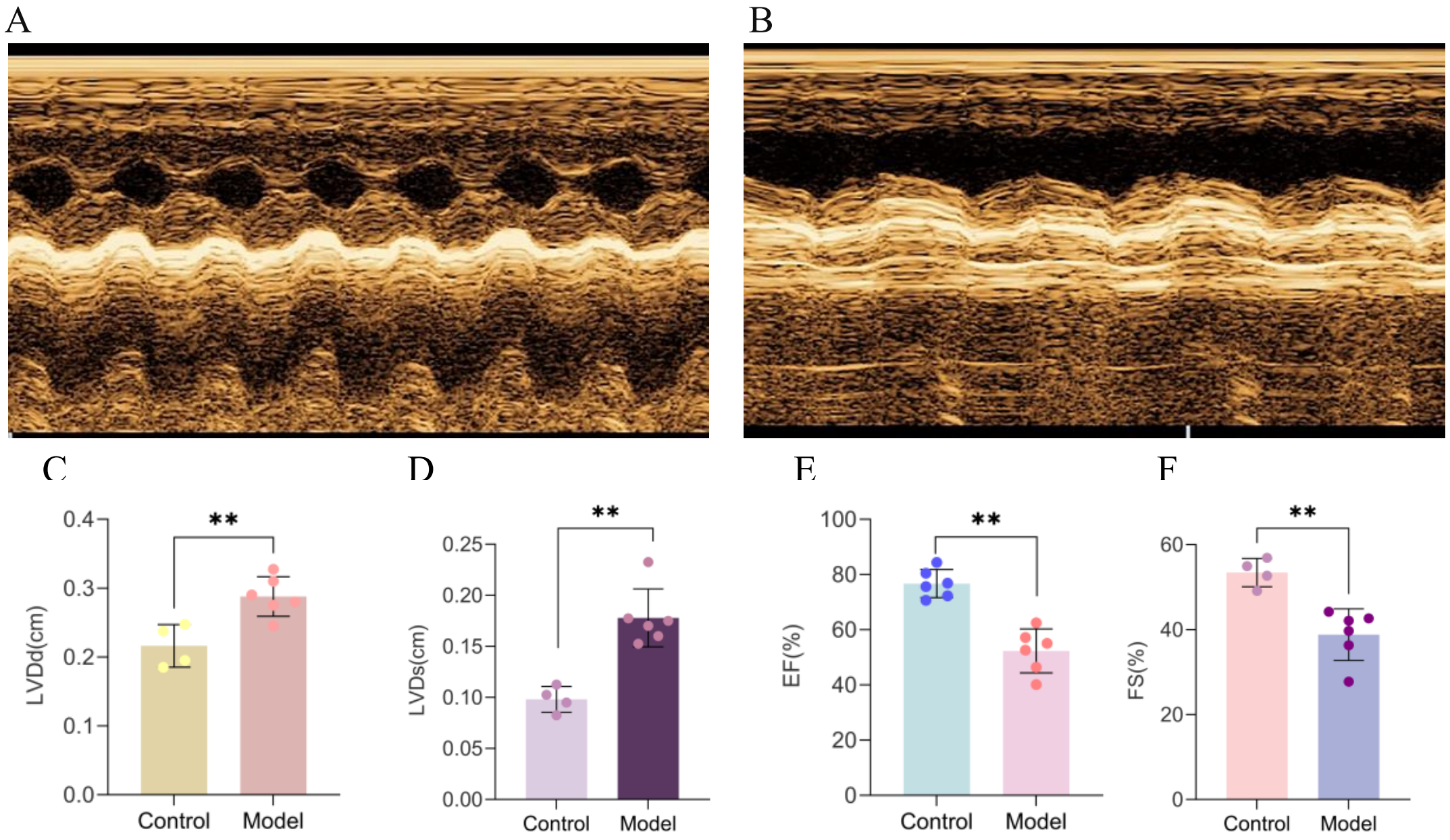

Figure 1: Isoproterenol-induced Decrease in Cardiac Function in Mice. A: Echocardiogram of the control group mice; B: Echocardiogram of the model group mice; C-F: Left ventricular diameter (LVDd), left ventricular diameter at systole (LVDs), ejection fraction (EF), and short-axis shortening rate (FS) of the left ventricle in mice; n≥4; Compared to the control group, **P<0.01

Assessment of cardiac weight provided additional insights into physiological changes among the experimental groups. Mice in the isoproterenol-induced heart failure model group exhibited a significant increase in body weight starting from day 13 of drug administration, compared to controls(Fig2A). Moreover, body weight and cardiac weight significantly increased in the model group after 24 days of isoproterenol administration, indicative of myocardial hypertrophy(Fig2B-C)(P<0.01). WGA staining demonstrated a significant increase in cardiomyocyte cross-sectional area in the model group compared to controls(Fig2D-E)(P<0.01).By examining BNP in both groups using Western Blot, we found that BNP was significantly elevated in the model group compared to the control group(Fig2F-G)(P<0.01). These findings highlight the capacity of isoproterenol to induce cardiac hypertrophy in mice.

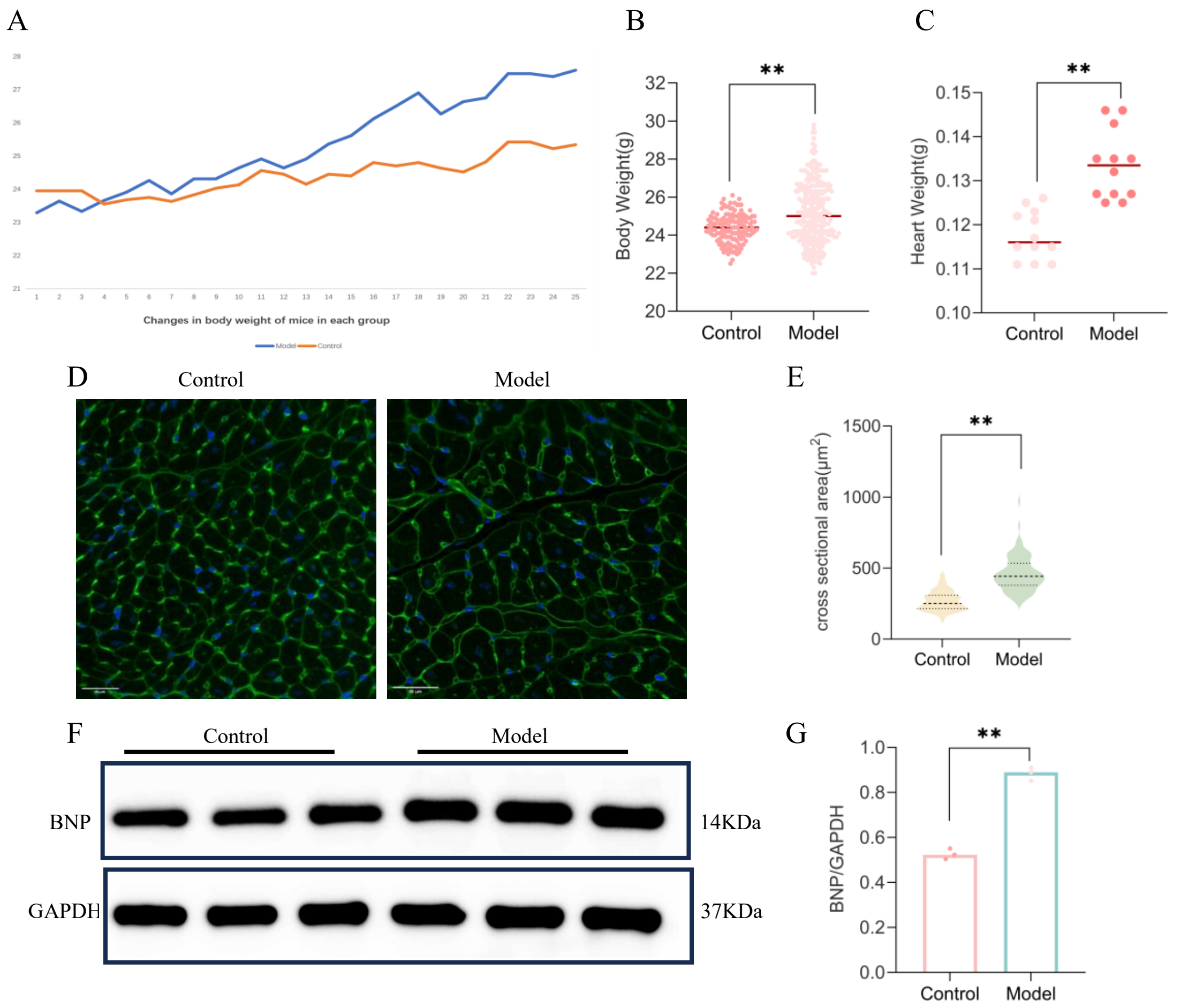

Figure2：Isoproterenol-induced heart failure in mice. A: Graph of changes in mouse body weight; B-C: Body and cardiac weights of the two groups of mice; n=12; D-E: Myocardial WGA staining of mice; n=5; F-G: BNP protein expression levels of the two groups of mice; n=3. Compared with the control group, ***P*<0.01

Morphological and pathological staining of the heart in mice revealed significant differences in myocardial structure between the two groups (Figure 3A). Compared to the control group, the model group mice exhibited a significantly larger heart volume (Figure 3A). A comparison of cross-sectional images from the hearts of the two groups showed that the ventricular cavity in the model group mice was notably enlarged (Figure 3B). HE staining results indicated that the model group mice exhibited disordered myocardial structure, vascular proliferation, and myocardial hemorrhage (Figure 3C-D). These findings collectively highlight the pathological changes in myocardial hypertrophy induced by isoproterenol in mice.

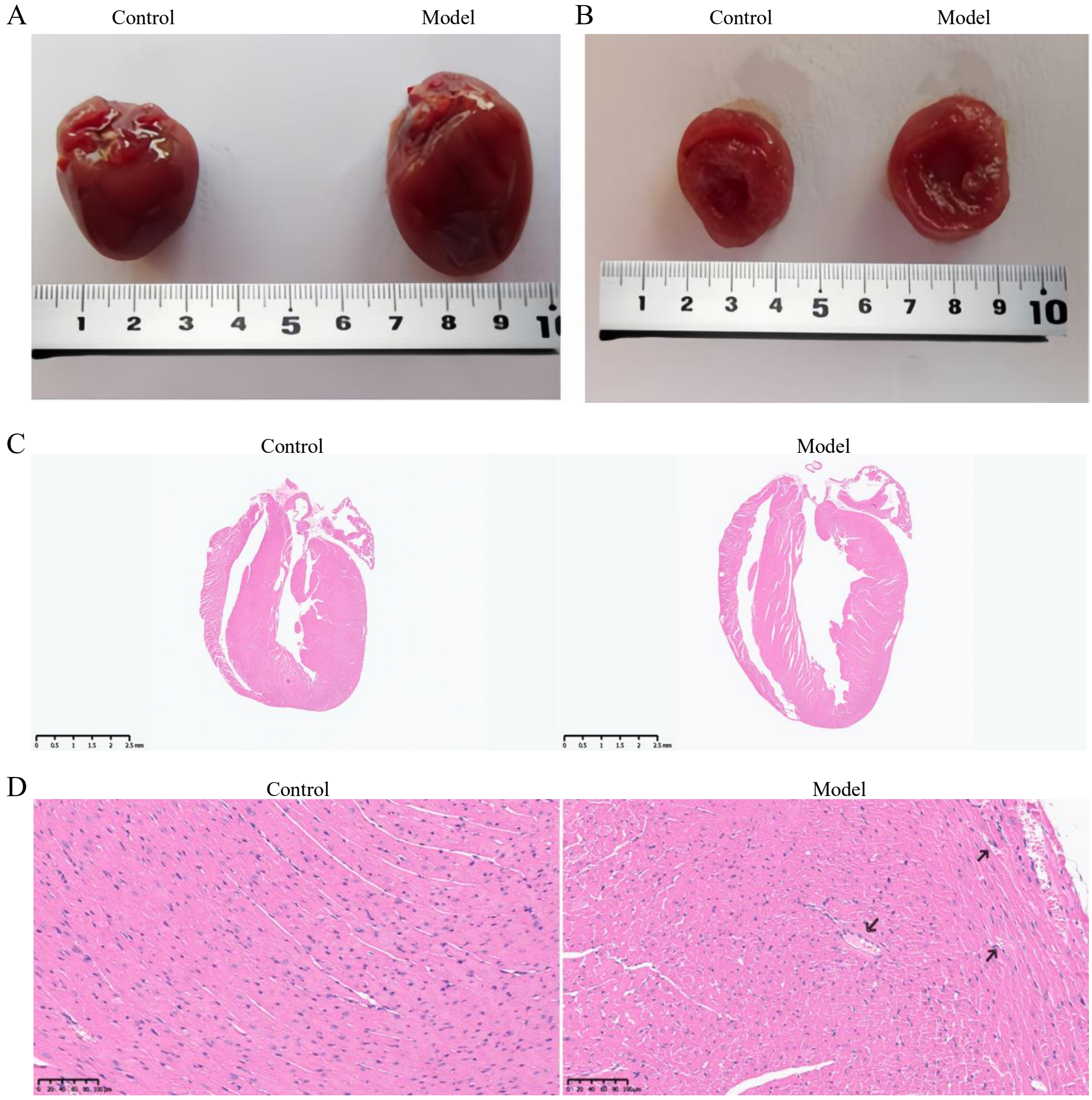

Figure 3: Gross view and pathological staining of mouse heart . A: Gross view of mouse heart; B: Coronary section of mouse heart; C-D: HE staining of mouse myocardial tissue; n=5

2.The expression of Nesprin-1 in the heart of mice with isoproterenol induced heart failure was significantly reduced

The immunofluorescence analysis revealed a significant decrease in Nesprin-1 expression in the hearts of mice induced with isoproterenol-induced heart failure(Fig4A)(P<0.01).By fluorescent staining of Nesprin-1 with specific antibodies, we observed that Nesprin-1 was mainly located at the nuclear membrane of cardiomyocytes (Fig4A). This localization is consistent with its function in maintaining nuclear membrane structure and nuclear-cell skeleton connection.

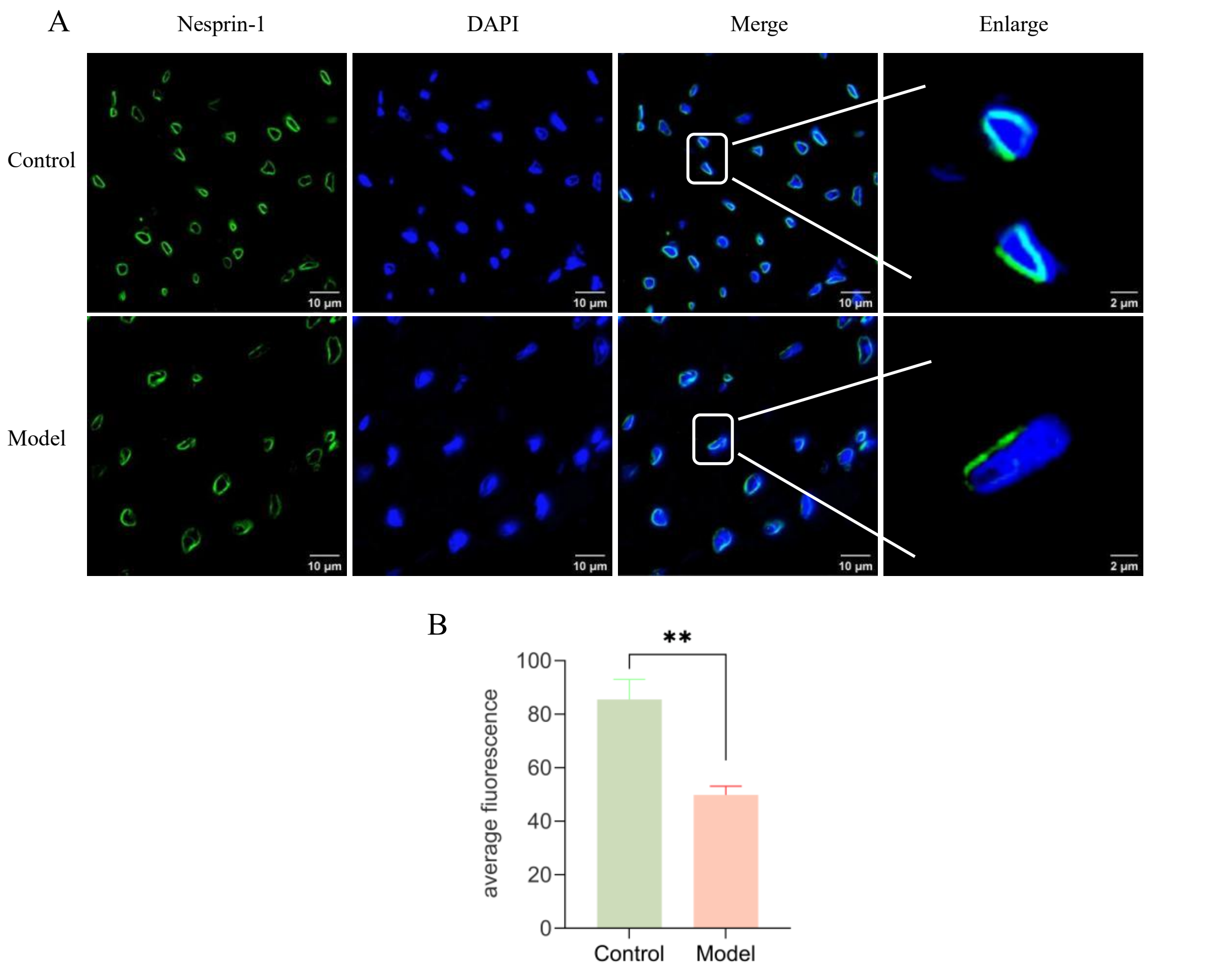

Figure 4: Expression changes of Nesprin-1 in myocardial tissue. A-B: Immunofluorescence staining of Nesprin in mouse myocardial tissue; n=5; Compared with the control group, $^{**}P<0.01$

3.Dysregulation of Nesprin-1 expression in cardiac tissue of isoproterenol induced heart failure mouse model affects nuclear homeostasis in heart failure

In our experimental study, we employed immunofluorescence to investigate the effects of Nesprin-1 expression changes on nuclear homeostasis in cardiomyocytes. By randomly selecting 40 fluorescently stained pathological sections from five model groups, we obtained 40 tissue micrographs. Ten nuclei were randomly selected from each image and analyzed using ImageJ software for morphological parameters including nuclear area, perimeter, and aspect ratio. Results demonstrated that compared with the control group, the nuclear morphology of cardiomyocytes in the model group exhibited significant abnormalities, characterized by marked increases in nuclear area, perimeter, and aspect ratio, along with a notable reduction in nuclear roundness (Figure 5A)($P<0.01$). Furthermore, immunofluorescence staining revealed partial or complete absence of Nesprin-1 on the nuclear membrane in some cardiomyocytes of the model group (Figure 5B)($P<0.01$). These findings indicate that

Nesprin-1 expression dysregulation affects nuclear homeostasis in ISO-induced HF mouse models, though the causal relationship between these two factors requires further investigation.

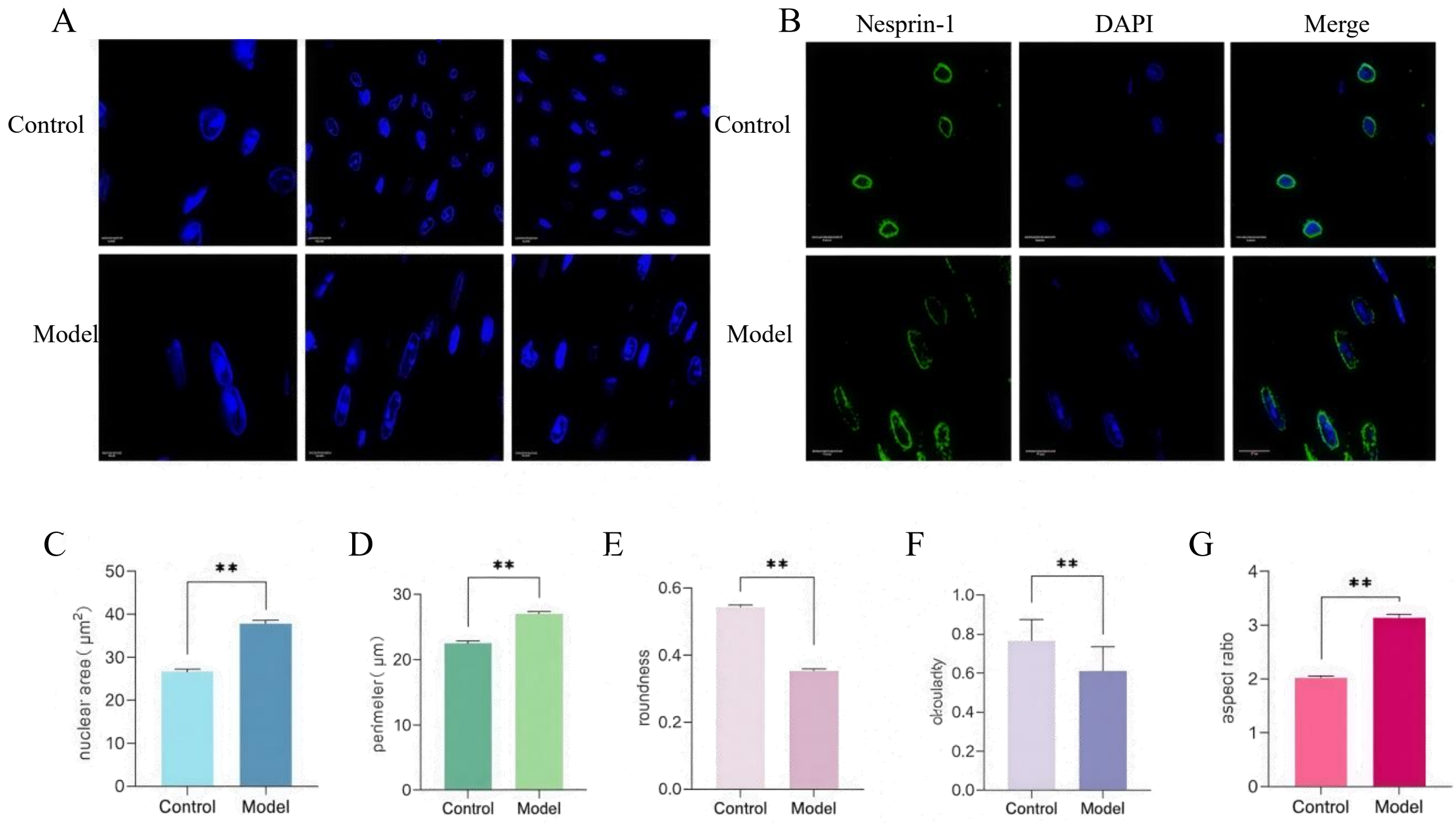

Figure 5: Nuclear Homeostasis Disruption in Heart Failure Mice. Note: A: Changes in nuclear morphology; B: Nesprin-1 immunofluorescence staining; C-G: Nuclear area, nuclear perimeter, nuclear roundness , and aspect ratio of the two groups of mice; n=5; Compared to the control group, $^{**}P<0.01$

4.In a mouse model of heart failure with reduced Nesprin-1 expression, oxidative stress responses were increased

Oxidative stress, characterized by an imbalance between oxidizing agents and antioxidant defenses, predisposes tissues to oxidation. This imbalance fuels neutrophilic inflammation, upregulates protease secretion, and fosters the generation of various oxidative intermediates. Acknowledged as a detrimental factor contributing to aging and various diseases, oxidative stress primarily stems from the overproduction of free radicals within the body. In line with this, Nesprin-1 depletion in Drosophila melanogaster has been correlated with mitochondrial dysfunction, thereby inducing oxidative stress[31]. To investigate this phenomenon, we evaluated the expression levels of malondialdehyde (MDA) and superoxide dismutase (SOD) in cardiac tissue. Notably, compared to the control groups, the model group exhibited heightened MDA activity and diminished SOD content(Fig6A-B)($P<0.05$). These results indicate that oxidative stress levels were significantly increased and

antioxidant capacity was inhibited in the Nesprin-1 dysregulated HF model.

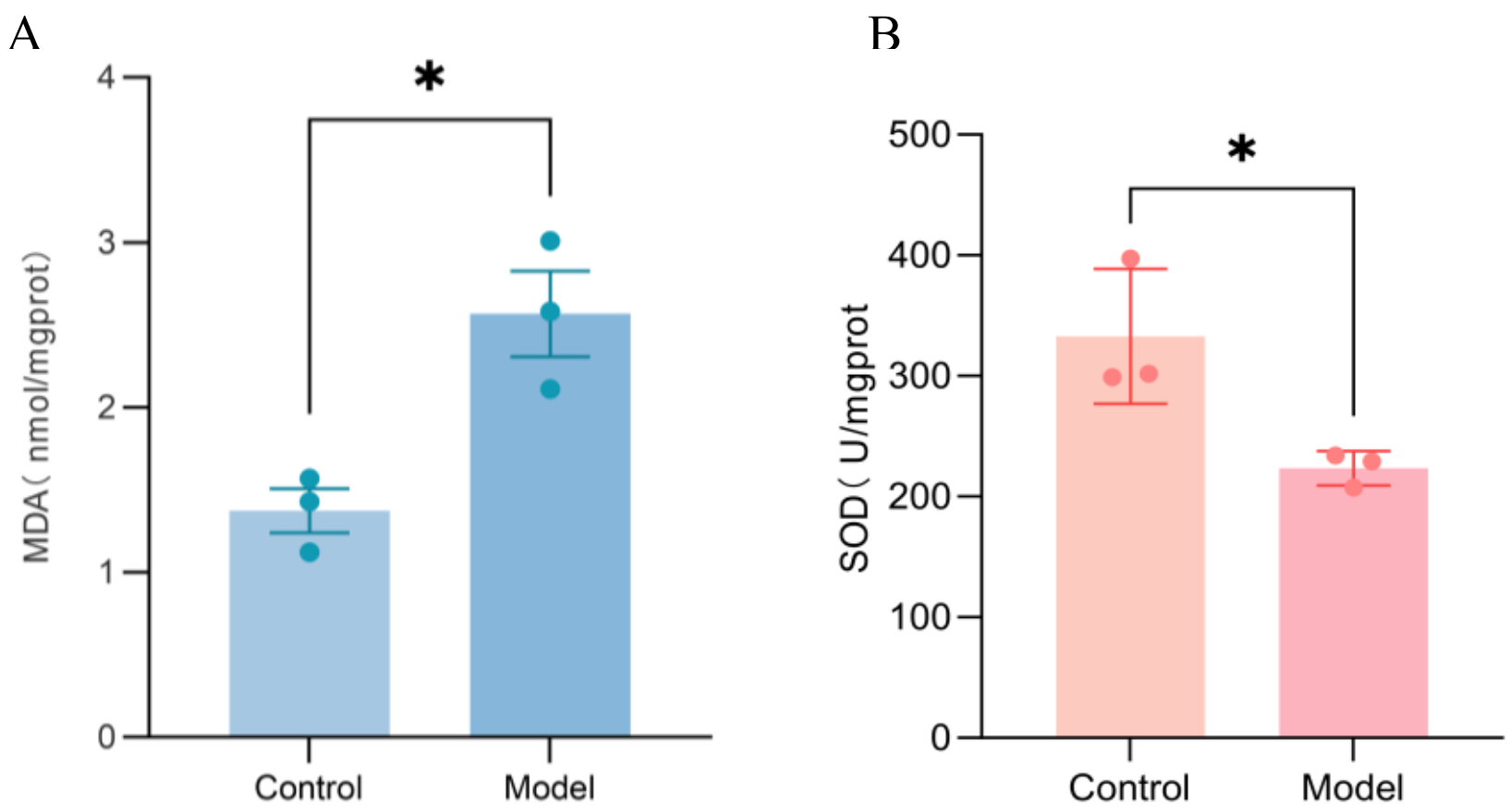

Figure 6: Oxidative stress aggravation in heart failure mice. A-B: Quantitative analysis of MDA and SOD in mouse myocardium; MDA: malondialdehyde; SOD: superoxide dismutase; n=3; compared with the control group, *$P<0.05$

5.The expression of Cx43 was downregulated in the myocardium of mice with dysregulation of Nesprin-1 expression in heart failure

Conduction dysfunction is a hallmark feature observed in the isoproterenol-induced heart failure mouse model. Cx43, primarily localized at the intercalated discs within mature cardiac myocytes, plays a pivotal role in facilitating electrical conduction among myocytes and enabling chemical signaling and energy substrate exchange, essential for coordinating cellular function[32]. Through immunofluorescence analysis, we investigated the expression levels of Cx43 in mice from each experimental group.Our results demonstrate a significant reduction in Cx43 expression specifically within the model group(Fig7A-B)($P<0.01$). In addition, combined with our previous studies, this result will provide a reliable experimental basis for our further study on how the reduction of Nesprin-1 expression affects the localization and function of Cx43 and jointly participates in the occurrence of conduction dysfunction in ISO-induced HF model.

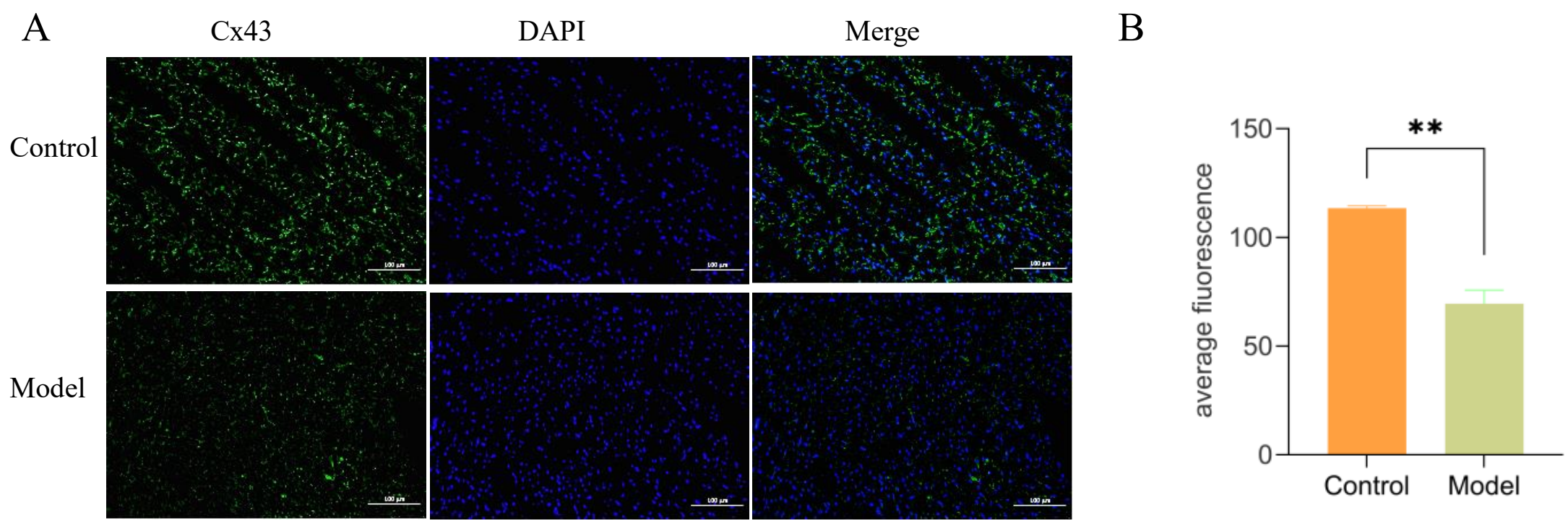

Figure 7: Expression of Cx43 in both groups of mice. A: Cx43 expression was detected by immunofluorescence; B Quantitative analysis of the Cx43; n=5; compared with the control group, ***P*<0.01

6.The myocardial DNA damage was exacerbated in mice exhibiting Nesprin-1 expression dysregulation during heart failure

Decreased Nesprin expression is associated with activated cellular DNA damage in the isoproterenol-induced heart failure mouse model. Nesprin plays a crucial role in maintaining genomic integrity, and its reduction may lead to impaired DNA repair mechanisms[33]. To investigate this phenomenon, immunofluorescence and Western blot analyses were conducted to assess the expression levels of 53BP1 and γH2AX in cardiac tissue. Our results reveal an increased expression of 53BP1 and γH2AX in the model group compared to the control groups(Fig8A-D)(*P*<0.01). In agreement with this, Western blot indicated elevated levels of DNA damage in Nesprin-deficient hearts(Fig9A-C)(*P*<0.01).These results suggest that the decrease in Nesprin-1 expression may interact with the accumulation of DNA damage in the occurrence of HF.

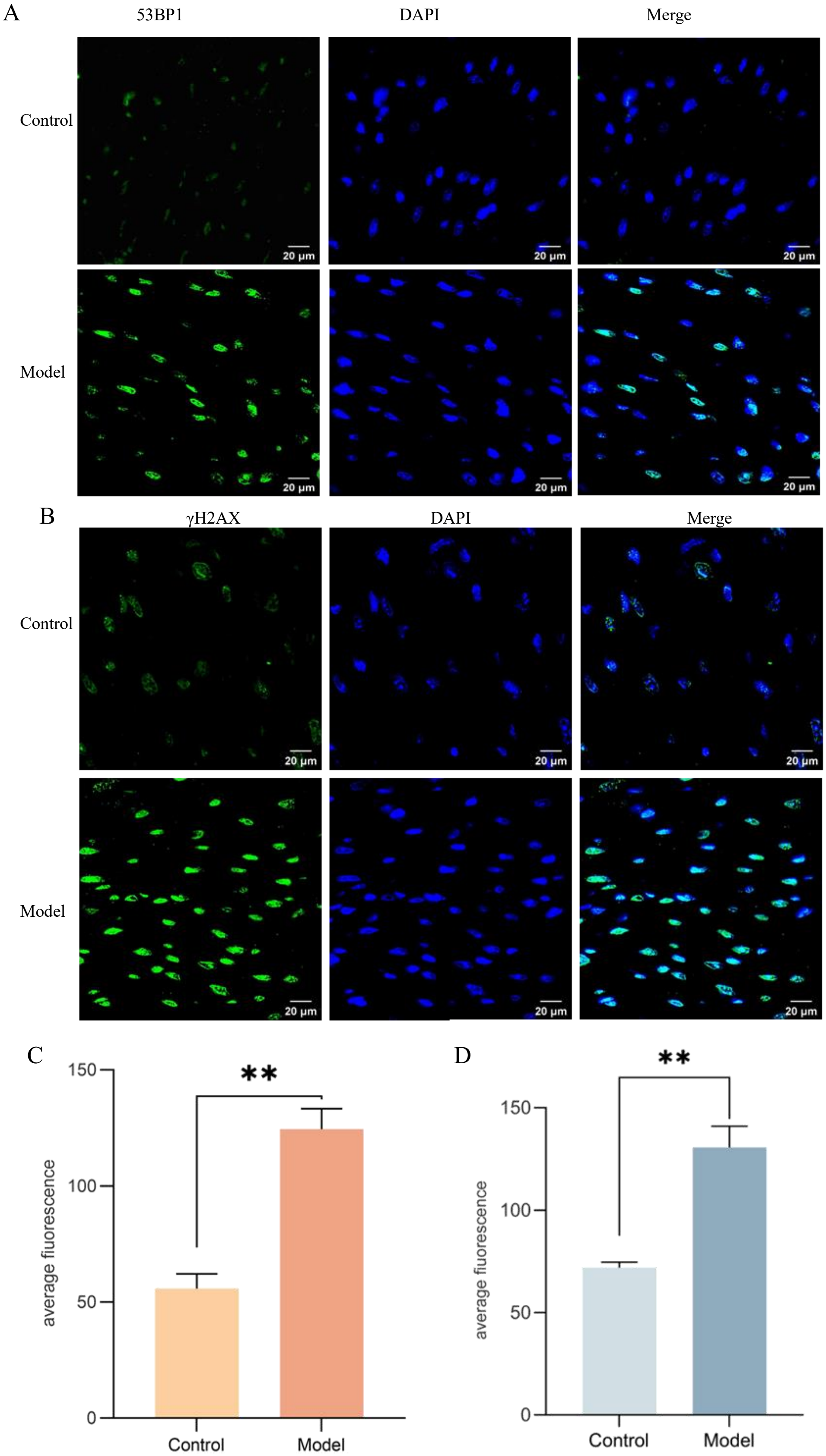

Figure 8: Increased DNA damage in myocardium of mice with heart failure. A-D: Detection of expression of 53BP1 and γH2AX by immunofluorescence; 53BP1: P53 binding protein 1; γH2AX: phosphorylated histone; n=5; compared with the control group,***P*<0.01

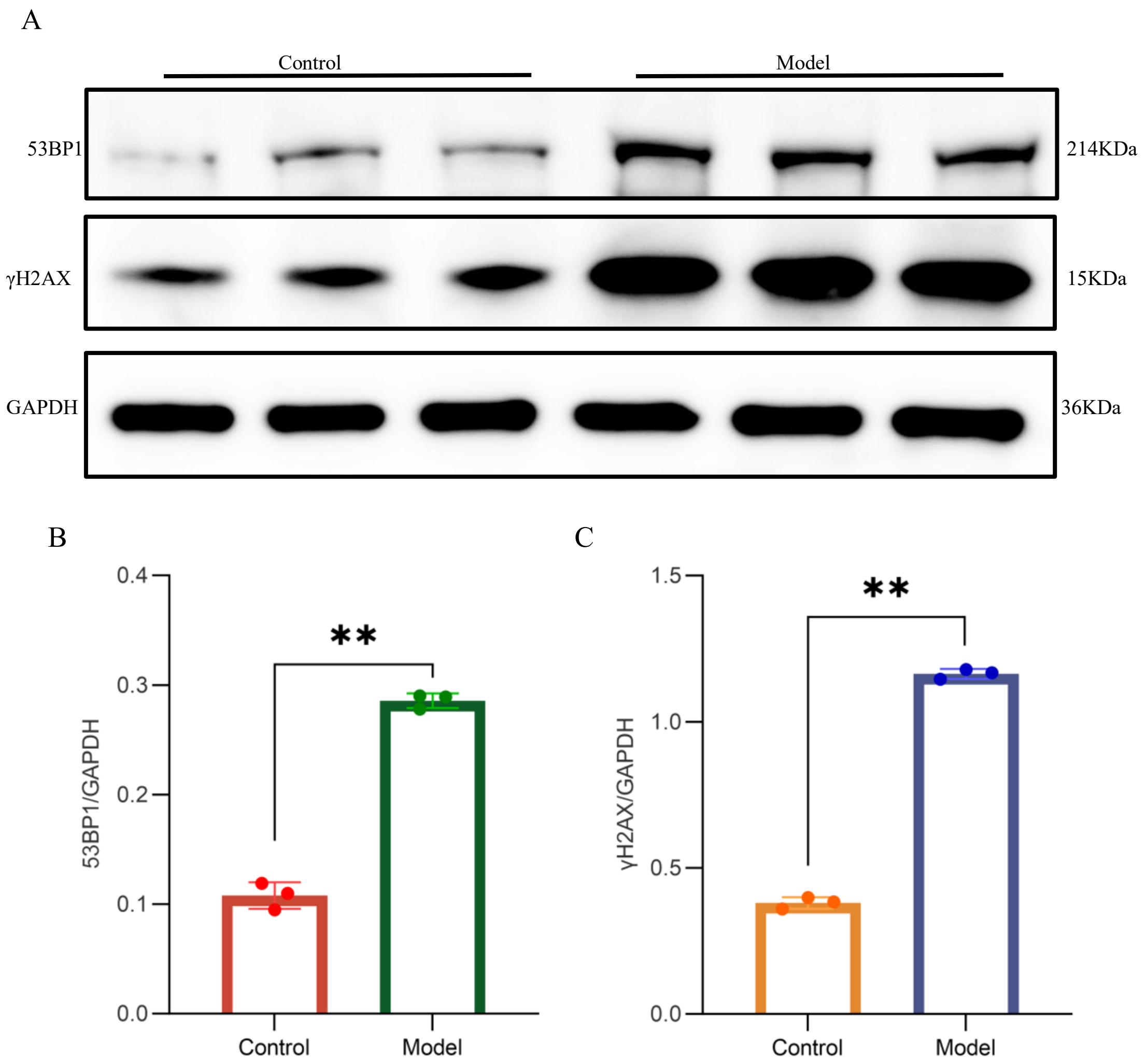

Figure 9: Increased DNA damage in myocardium of mice with heart failure; A-C: Protein quantitative analysis of 53BP1 and γH2AX; 53BP1: P53 binding protein 1; γH2AX: phosphorylated histone; n=3; compared with the control group,***P*<0.01

7.Dysregulation of Nesprin-1 interferes with histone methylation in heart failure

Histone methylation represents a critical modification that alters chromosome structure. Recent studies have highlighted the pivotal role of histone methylation modifications in myocardial hypertrophy, fibrosis, and cardiac function[34, 35]. Through immunofluorescence and Western blot analysis, we assessed the expression levels of H3K9me3 and H3K27me3 in mice from each experimental group. As depicted in the figures, the model group exhibited significantly increase expression levels of H3K9me3 and H3K27me3 compared to the control groups(Fig10A-D)(*P*<0.01). This result is consistent with the Western blot results(Fig11A-C)(*P*<0.01). These findings suggest that the histone methylation pattern disorder in the isoproterenol induced heart failure mouse model may be associated with the decreased level of Nesprin-1.

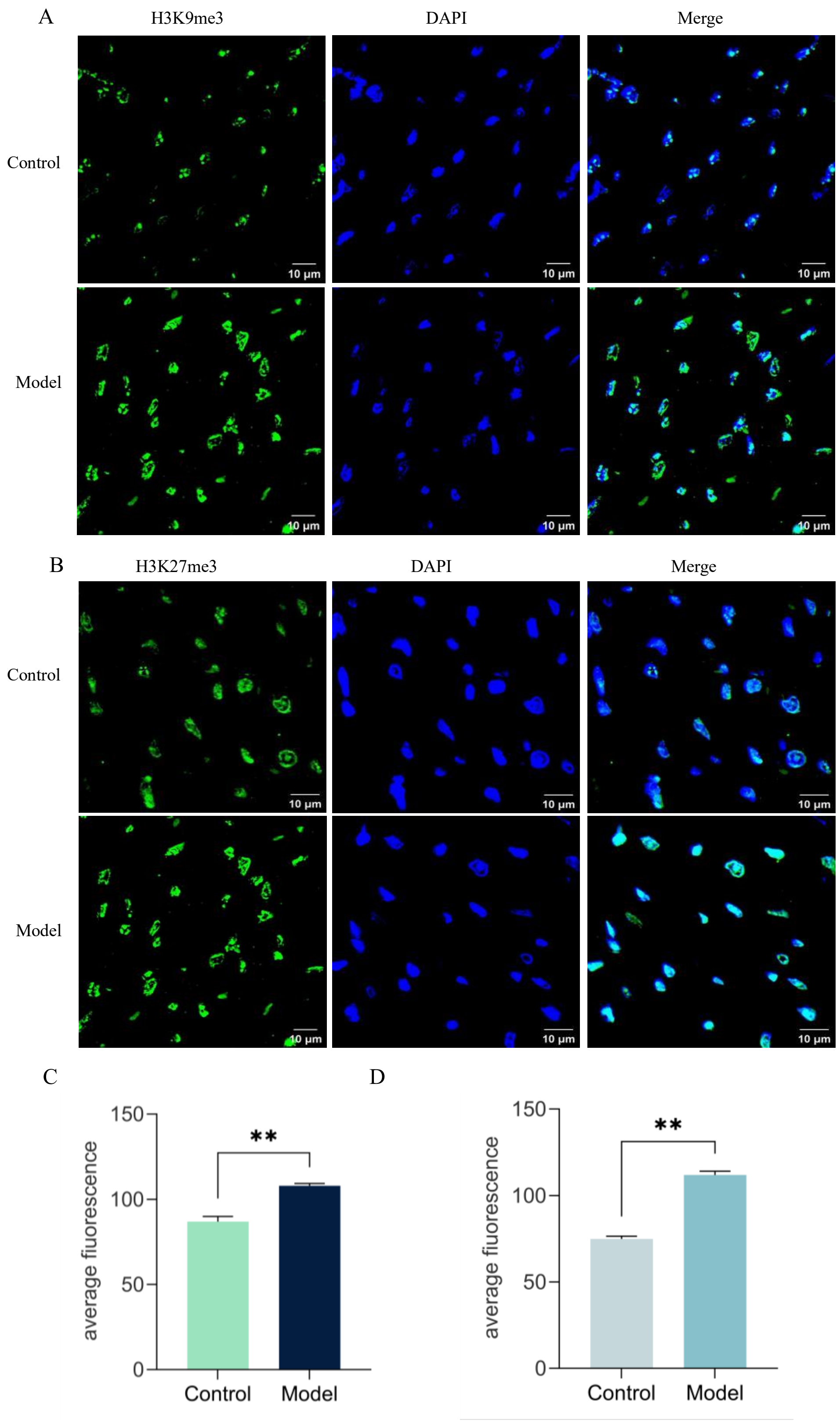

Figure 10: Changes in chromatin recombination in the myocardium of heart failure mice.A-D: Immunofluorescence detection of H3K9me3 and H3K27me3 expression; H3K9me3: trimethylation of histone H3 lysine 9; H3K27me3: trimethylation of histone H3 lysine 27; n=5; compared to the control group,***P*<0.01

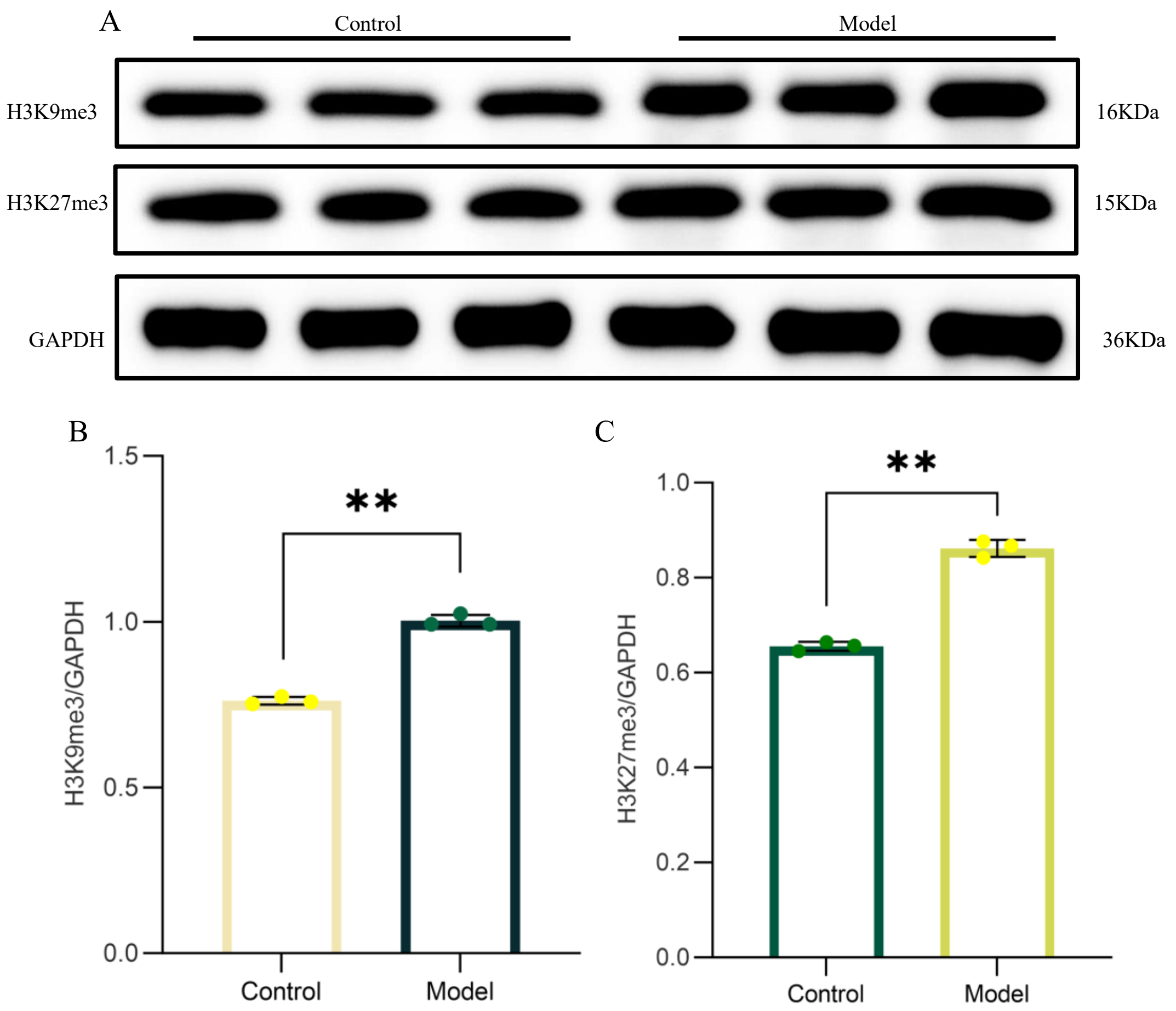

Figure 11: Changes in chromatin recombination in the myocardium of heart failure mice. A-C: Quantitative analysis of H3K9me3 and H3K27me3 proteins; H3K9me3: trimethylation of lysine 9 on histone H3; H3K27me3: trimethylation of lysine 27 on histone H3; n=3; compared with the control group,***P*<0.01

8.ERK pathway is activated in nuclear homeostasis abnormalities mediated by Nesprin-1 expression disorder and heart failure

The ERK pathway is recognized as a pivotal regulator in the development of myocardial hypertrophy. Studies have elucidated the formation of Nesprin-2/ERK complexes at sites of DNA damage in aortic smooth muscle cells, indicating a potential role of Nesprin proteins in modulating ERK signaling[36]. To investigate this phenomenon in the context of heart failure, we employed Western blot analysis to evaluate the expression levels of ERK in cardiac tissue samples from mice in each experimental group. Strikingly, our results revealed a notable increase in ERK expression in the model group compared to the control group, indicative of the activation of the ERK pathway in the cardiac tissue of the isoproterenol-induced heart failure mouse model(Fig12A-C)(P<0.01). Combined with the reduced expression of

Nesprin-1 in iprindel induced heart failure mice, we speculate that the reduced expression of Nesprin-1 may be associated with the activation of ERK pathway.

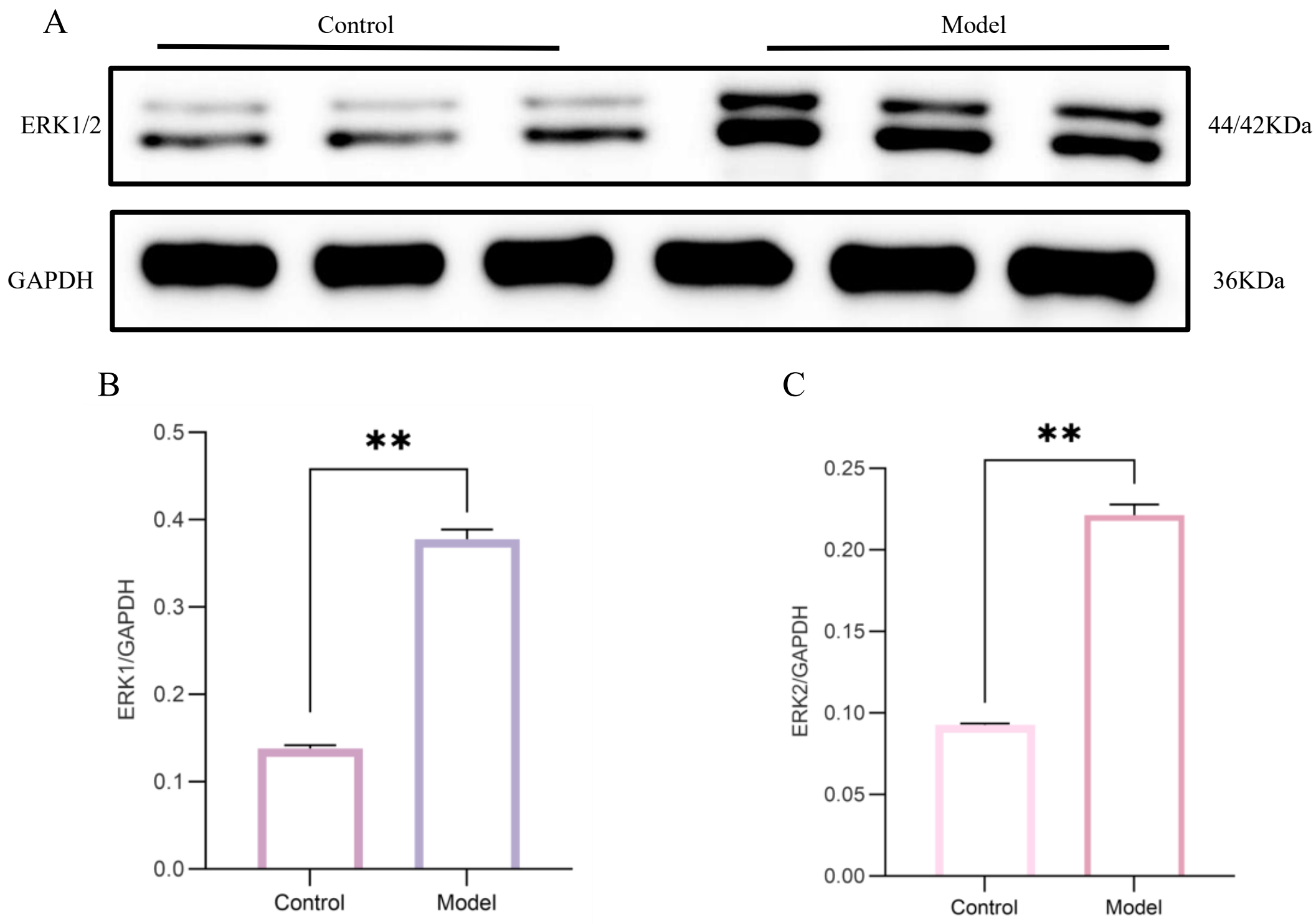

Figure 12: Downregulation of ERK1/2 expression in myocardium of mice with heart failure; A-C: Detection of ERK1/2 expression by protein immunoblotting; ERK: extracellular regulated protein kinase; n=3; compared with the control group,***P*<0.01

Discussion

Heart failure stands as an enduring challenge within global healthcare systems, presenting a formidable burden characterized by its pervasive prevalence, grim prognostic outlook, and profound detriment to patients' quality of life. Despite notable strides in HF management witnessed over recent decades, including the advent of neurohormonal blockade, device-based interventions, and cardiac transplantation, the persistently high rates of mortality and morbidity underscore the insufficiency of current therapeutic strategies[37-39]. Consequently, there exists an imperative mandate for sustained investigative efforts directed towards unraveling the intricate molecular mechanisms governing HF pathogenesis.

In this study, We induced myocardial hypertrophy in mice by intraperitoneal injection of ISO. Our findings indicate that the administration of ISO successfully induced myocardial hypertrophy, characterized by significant changes in heart weight, body weight, and WGA pathological staining results compared to the control group

mice, consistent with previous studies [40, 41]. Long-term stimulation by pathological factors can lead to persistent changes in cardiac structure, resulting in HF [42]. HE staining revealed that Nesprin-1 deficiency exacerbated myocardial structural disorder, vascular proliferation, and myocardial hemorrhage. Additionally, cardiac function tests confirmed the onset of HF in the mice, with significant changes in LVEDd, LVESd, EF, and FS. When myocardial cell volume and pressure overload occur, ventricular muscle cells release BNP, which exerts sodium-losing, diuretic, vasodilatory, and antihypertensive effects [43]. BNP is a crucial biomarker for the clinical diagnosis of HF . Western blotting detected a significant increase in BNP in the myocardial tissue of mice induced by isoproterenol, providing a more reliable basis for confirming the onset of HF in mice.

Nesprin are giant scaffolding proteins located at the NE that play essential roles in maintaining nuclear structure and regulating various cellular processes, including nuclear positioning, cytoskeletal organization, and gene expression[27, 44-48]. While previous research has primarily focused on the role of Nesprin in skeletal and muscular diseases, their involvement in cardiac pathology, particularly HF, remains poorly understood.In this study, we used the ISO-induced mouse heart failure model to initially assess the changes in Nesprin-1 expression and its potential links with nuclear structure, signaling pathways, and gene expression regulation. The ISO-induced HF mouse model established and validated in this study provides new insights into the pathophysiology of heart failure and the role of Nesprin-1 in cardiac function.

Our research has revealed that, compared to the control group, the expression of Nesprin-1 in the myocardium of ISO-induced HF mice is significantly reduced. This finding suggests that changes in Nesprin-1 expression may be linked to pathological remodeling processes associated with HF, such as myocardial hypertrophy and dysfunction. The downregulation of Nesprin-1 may be related to abnormalities in the morphology, nuclear localization, and structural integrity of myocardial cells, which have been preliminarily observed through histopathological examination. However, the specific molecular mechanisms still require further investigation. Additionally, the

dysregulation of Nesprin-1 expression may affect nuclear stability by disrupting the integrity of the nuclear membrane, potentially leading to cellular dysfunction. These findings suggest that, as a key component of the nuclear scaffold and cytoskeletal connection complex, changes in Nesprin-1 expression may be associated with nuclear instability and abnormal cell signaling.

The exact mechanisms underlying Nesprin-1-mediated pathogenesis in HF remain incompletely understood, but our study has shed light on several potential avenues for further investigation. our findings elucidate the multifaceted role of Nesprin-1 in HF pathogenesis, encompassing oxidative stress, electrical conduction abnormalities, and epigenetic dysregulation. First, Nesprin-1 may affect cardiac morphology through interactions with cytoskeletal elements and extracellular matrix proteins, thereby regulating the signaling pathways involved in fibroblast activation. However, the causal relationship between Nesprin-1 and these processes has not been directly demonstrated. For example, how Nesprin-1 affects fibroblast activation by influencing cardiac morphology through interactions with cytoskeletal components and extracellular matrix proteins.

Additionally, Nesprin appears to modulate oxidative stress and DNA damage responses in the heart. Reduced Nesprin expression is associated with heightened oxidative stress, as evidenced by alterations in oxidative stress markers such as malondialdehyde levels and decreased superoxide dismutase content. Increased expression of DNA damage repair proteins, such as γH2AX and 53BP1, further suggests Nesprin's role in preserving genomic integrity and protecting against oxidative stress-induced cardiac injury. Furthermore, the observed reduction in connexin 43 expression in Nesprin-deficient hearts highlights Nesprin's involvement in regulating cardiac conduction and electromechanical coupling. Diminished Cx43 expression may contribute to conduction abnormalities and arrhythmias commonly observed in HF, emphasizing Nesprin's role in regulating both structural and functional aspects of cardiac tissue. Lastly, alterations in histone methylation patterns, specifically changes in H3K9me3 and H3K27me3 levels in Nesprin-deficient hearts, suggest dysregulated epigenetic regulation in HF pathogenesis. These findings

underscore the intricate interplay between Nesprin and epigenetic regulatory mechanisms in maintaining cardiac homeostasis.

Activation of the ERK pathway has been implicated in various hypertrophic stimuli and is known to regulate gene expression, protein synthesis, and cell survival in cardiomyocytes[49-51]. In addition to the identified alterations in Nesprin expression and its implications in cardiac remodeling, our study elucidates the involvement of the ERK signaling pathwaymay be involved in mediating Nesprin-1-induced cardiac hypertrophy and remodeling in response to isoproterenol stimulation. Activation of the ERK pathway has been implicated in various cellular processes, including cell proliferation, differentiation, and survival[52-54]. Here, we demonstrate a significant increase in ERK expression in the cardiac tissue of mice subjected to isoproterenol-induced heart failure, suggesting the activation of the ERK pathway in response to cardiac stress. The observed upregulation of ERK signaling in Nesprin-deficient hearts highlights a potential interaction between Nesprin and the ERK pathway in regulating cardiac function. Previous studies have indicated the formation of Nesprin-2/ERK complexes at sites of DNA damage, implicating Nesprin proteins in the modulation of ERK signaling in other cellular contexts[36]. Our findings extend this understanding to the cardiac setting, where Nesprin deficiency may contribute to ERK pathway activation, thereby exacerbating myocardial injury and dysfunction. The activation of the ERK pathway in response to cardiac stressors, such as isoproterenol administration, suggests a role of ERK signaling in mediating pathological remodeling and maladaptive responses in the heart. Future studies aimed at elucidating the precise mechanisms underlying the crosstalk between Nesprin and the ERK pathway may provide further insights into the molecular mechanisms of heart failure pathogenesis.

The identification of Nesprin-1 as a potential regulator of HF pathogenesis opens up new avenues for therapeutic intervention. Targeting Nesprin-1 and its associated signaling pathways may offer novel strategies for preventing or attenuating adverse cardiac remodeling and dysfunction in HF[55-58]. Small molecule inhibitors, gene therapy approaches, or gene editing techniques could be explored to modulate

Nesprin-1 expression or activity selectively in the heart. Additionally, our study underscores the importance of further research into the molecular mechanisms governing nuclear envelope dynamics and their contribution to cardiovascular disease. In conclusion, our study provides compelling evidence implicating Nesprin-1 in the pathogenesis of HF induced by isoproterenol. By elucidating the role of Nesprin-1 in cardiac remodeling, electrical conduction, we have shed light on novel molecular mechanisms underlying HF pathophysiology. Further investigation into the functional significance of Nesprin-1 and its interactions with key signaling pathways may yield valuable insights into disease progression and facilitate the development of targeted therapies for HF. Ultimately, our findings contribute to a deeper understanding of the complex molecular networks driving HF and offer hope for improved outcomes for patients afflicted with this devastating condition. This comprehensive discussion highlights the significance of our findings and provides a roadmap for future research endeavors aimed at unraveling the intricate molecular mechanisms of HF pathogenesis and identifying innovative therapeutic strategies to combat this disease.

However, this study still has some limitations. Firstly, the lack of specific inhibitors for Nesprin-1, along with the high mortality and lengthy construction period of Nesprin gene knockout mice, prevented us from directly establishing a causal relationship between Nesprin-1, oxidative stress, reduced Cx43 expression, increased DNA damage, and chromatin recombination disorders. Secondly, while we observed that decreased Nesprin-1 expression is associated with various pathological features, such as myocardial structural disorder, changes in oxidative stress markers, and increased expression of DNA damage repair proteins, these findings are only correlational. The specific molecular mechanisms require further functional experiments to confirm. Additionally, this study primarily used ISO-induced HF mouse models. Future studies should validate the role of Nesprin-1 in other HF models, such as those induced by stress overload or myocardial infarction, to enhance the generalizability of the research results. Lastly, regarding the interaction between Nesprin-1 and the ERK signaling pathway and epigenetic regulation (such as histone methylation), this study only provides preliminary clues. The specific mechanisms

need to be elucidated through more in-depth molecular and cellular experiments. In future research, we plan to construct a Nesprin-1-specific knockout model, combined with multi-omics analysis and functional validation, to further uncover its exact role in HF.

Declarations

Ethics approval and consent to participate

Not applicable.

Consent for publication

The manuscript was obtained from all authors for consent for submission and publication

Competing interests

The authors declare no competing interests

Funding

This research was funded by the Hubei Provincial Natural Science Foundation Project (Joint Fund Project, Project No.: 2024AFD143).The funders had no role in study design, data collection and analysis, decision to publish, or preparation of the manuscript.

Authors' contributions

Qin Qin and Dong Cengyuan completed the experiment and wrote the manuscript; Zhou Ziyi participated in some of the experiments and manuscript writing; Liu Yangyuanzhi was involved in the article revision; Professor Cao Chunyu provided guidance on the experimental design; Doctor Teng Lin guided the entire experimental process and manuscript writing.

Availability of Data and Material

All relevant data are within the paper and its Supporting Information files.

Acknowledgements

I would like to thank CJH for their technical assistance in this experiment. And I am very grateful for the funding provided by the Provincial Natural Science Foundation for this experiment.

Reference

[1] Tanai E, Frantz S. Pathophysiology of Heart Failure [J]. Compr Physiol, 2015, 6(1): 187-214.

[2] McDonagh T A, Metra M, Adamo M, et al. 2021 ESC Guidelines for the diagnosis and treatment of acute and chronic heart failure: Developed by the Task Force for the diagnosis and treatment of acute and chronic heart failure of the European Society of Cardiology (ESC) With the special contribution of the Heart Failure Association (HFA) of the ESC [J]. Rev Esp Cardiol (Engl Ed), 2022, 75(6): 523.

[3] Bozkurt B, Coats A J S, Tsutsui H, et al. Universal Definition and Classification of Heart Failure [J]. Journal of Cardiac Failure, 2021, 27(4): 387-413.

[4] Kemp C D, Conte J V. The pathophysiology of heart failure [J]. Cardiovasc Pathol, 2012, 21(5): 365-71.

[5] Ghionzoli N, Gentile F, Del Franco A M, et al. Current and emerging drug targets in heart failure treatment [J]. Heart Failure Reviews, 2021, 27(4): 1119-36.

[6] Savarese G, Becher P M, Lund L H, et al. Global burden of heart failure: a comprehensive and updated review of epidemiology [J]. Cardiovasc Res, 2023, 118(17): 3272-87.

[7] Shahim B, Kapelios C J, Savarese G, et al. Global Public Health Burden of Heart Failure: An Updated Review [J]. Card Fail Rev, 2023, 9: e11.

[8] Borovac J A, D'Amario D, Bozic J, et al. Sympathetic nervous system activation and heart failure: Current state of evidence and the pathophysiology in the light of novel biomarkers [J]. World J Cardiol, 2020, 12(8): 373-408.

[9] Bencivenga L, Palaia M E, Sepe I, et al. Why Do We Not Assess Sympathetic Nervous System Activity in Heart Failure Management: Might GRK2 Serve as a New Biomarker? [J]. Cells, 2021, 10(2).

[10] Wang J, Dai M, Cao Q, et al. Carotid baroreceptor stimulation suppresses ventricular fibrillation in canines with chronic heart failure [J]. Basic Research in Cardiology, 2019, 114(6).

[11] Huang Y, Liu Y-W, Pan H-Z, et al. Transthoracic Pulmonary Artery Denervation for Pulmonary Arterial Hypertension [J]. Arteriosclerosis, Thrombosis, and Vascular Biology, 2019, 39(4): 704-18.

[12] Triposkiadis F, Briasoulis A, Kitai T, et al. The sympathetic nervous system in heart failure revisited [J]. Heart Fail Rev, 2024, 29(2): 355-65.

[13] Grassi G, Mancia G, Esler M. Central and peripheral sympathetic activation in heart failure [J]. Cardiovasc Res, 2022, 118(8): 1857-71.

[14] Gronda E, Dusi V, D'Elia E, et al. Sympathetic activation in heart failure() [J]. Eur Heart J Suppl, 2022, 24(Suppl E): E4-E11.

[15] Munoz-Durango N, Fuentes C A, Castillo A E, et al. Role of the Renin-Angiotensin-Aldosterone System beyond Blood Pressure Regulation: Molecular and Cellular Mechanisms Involved in End-Organ Damage during Arterial Hypertension [J]. Int J Mol Sci, 2016, 17(7).

[16] Pugliese N R, Masi S, Taddei S. The renin-angiotensin-aldosterone system: a crossroad from arterial hypertension to heart failure [J]. Heart Fail Rev, 2020, 25(1): 31-42.

[17] Ferreira N S, Tostes R C, Paradis P, et al. Aldosterone, Inflammation, Immune System, and Hypertension [J]. Am J Hypertens, 2021, 34(1): 15-27.

[18] Weir M R, Dzau V J. The renin-angiotensin-aldosterone system: a specific target for hypertension management [J]. Am J Hypertens, 1999, 12(12 Pt 3): 205S-13S.

[19] Chen Y, He Q, Mo D-C, et al. The angiotensin receptor and neprilysin inhibitor, LCZ696, in heart failure: A meta-analysis of randomized controlled trials [J]. Medicine, 2022, 101(41).

[20] Heidenreich PA B B, Aguilar D,et al. Correction to: 2022 AHA/ACC/HFSA Guideline for the Management of Heart Failure: A Report of the American College of Cardiology/American Heart Association Joint Committee on Clinical Practice Guidelines [J]. Circulation, 2023, 147(14): e263-e421.

[21] Lachowska K, Gruchala M, Narkiewicz K, et al. Sympathetic Activation in Chronic Heart Failure: Potential Benefits of Interventional Therapies [J]. Curr Hypertens Rep, 2016, 18(7): 51.

[22] von Lueder T G, Kotecha D, Atar D, et al. Neurohormonal Blockade in Heart Failure [J]. Card Fail Rev, 2017, 3(1): 19-24.

[23] Ghionzoli N, Gentile F, Del Franco A M, et al. Current and emerging drug targets in heart failure treatment [J]. Heart Fail Rev, 2022, 27(4): 1119-36.

[24] Crisp M, Liu Q, Roux K, et al. Coupling of the nucleus and cytoplasm: Role of the LINC complex [J]. The Journal of Cell Biology, 2006, 172(1): 41-53.

[25] Meinke P, Nguyen T D, Wehnert M S. The LINC complex and human disease [J]. Biochem Soc Trans, 2011, 39(6): 1693-7.

[26] De Silva S, Catibog N, Shanahan C, et al. Investigating novel roles for nesprin-1 and the LINC complex in Dilated Cardiomyopathy [J]. Cardiovascular Research, 2022, 118(Supplement_1).

[27] Zhou C, Li C, Zhou B, et al. Novel nesprin-1 mutations associated with dilated cardiomyopathy cause nuclear envelope disruption and defects in myogenesis [J]. Hum Mol Genet, 2017, 26(12): 2258-76.

[28] Shanahan CM W P, Metcalfe JC. Isolation of gene markers of differentiated and proliferating vascular smooth muscle cells [J]. Circ Res, 1993, 73(1): 193-204.

[29] Chen Z, Ren Z, Mei W, et al. A novel SYNE1 gene mutation in a Chinese family of Emery-Dreifuss muscular dystrophy-like [J]. BMC Medical Genetics, 2017, 18(1).

[30] Stroud M J, Banerjee I, Veevers J, et al. Linker of Nucleoskeleton and Cytoskeleton Complex Proteins in Cardiac Structure, Function, and Disease [J]. Circulation Research, 2014, 114(3): 538-48.

[31] Lindenboim L, Grozki D, Amsalem-Zafran A R, et al. Apoptotic stress induces Bax-dependent, caspase-independent redistribution of LINC complex nesprins [J]. Cell Death Discov, 2020, 6(1): 90.

[32] Boengler K, Rohrbach S, Weissmann N, et al. Importance of Cx43 for Right Ventricular Function [J]. Int J Mol Sci, 2021, 22(3).

[33] Sur-Erdem I, Hussain M S, Asif M, et al. Nesprin-1 impact on tumorigenic cell phenotypes [J]. Mol Biol Rep, 2020, 47(2): 921-34.

[34] Yao L, He F, Zhao Q, et al. Spatial Multiplexed Protein Profiling of Cardiac Ischemia-Reperfusion Injury [J]. Circ Res, 2023, 133(1): 86-103.

[35] Li J, Sha Z, Zhu X, et al. Targeting miR-30d reverses pathological cardiac hypertrophy [J]. EBioMedicine, 2022, 81: 104108.

[36] Warren D T, Tajsic T, Porter L J, et al. Nesprin-2-dependent ERK1/2 compartmentalisation regulates the DNA damage response in vascular smooth muscle cell ageing [J]. Cell Death Differ, 2015, 22(9): 1540-50.

[37] Collaborators G B D R F. Global burden and strength of evidence for 88 risk factors in 204 countries and 811 subnational locations, 1990-2021: a systematic analysis for the Global Burden of Disease Study 2021 [J]. Lancet, 2024, 403(10440): 2162-203.

[38] Savarese G, Lund L H. Global Public Health Burden of Heart Failure [J]. Card Fail Rev, 2017, 3(1): 7-11.

[39] Sapna F, Raveena F, Chandio M, et al. Advancements in Heart Failure Management: A Comprehensive Narrative Review of Emerging Therapies [J]. Cureus, 2023, 15(10): e46486.

[40] Aimo A, Gaggin H K, Barison A, et al. Imaging, Biomarker, and Clinical Predictors of Cardiac Remodeling in Heart Failure With Reduced Ejection Fraction [J]. JACC Heart Fail, 2019, 7(9): 782-94.

[41] Goetze J P, Bruneau B G, Ramos H R, et al. Cardiac natriuretic peptides [J]. Nat Rev Cardiol, 2020, 17(11): 698-717.

[42] Salah K, Stienen S, Pinto Y M, et al. Prognosis and NT-proBNP in heart failure patients with preserved versus reduced ejection fraction [J]. Heart, 2019, 105(15): 1182-9.

[43] Apel E D, Lewis R M, Grady R M, et al. Syne-1, a dystrophin- and Klarsicht-related protein associated with synaptic nuclei at the neuromuscular junction [J]. J Biol Chem, 2000, 275(41): 31986-95.

[44] De Silva S, Fan Z, Kang B, et al. Nesprin-1: novel regulator of striated muscle nuclear positioning and mechanotransduction [J]. Biochem Soc Trans, 2023, 51(3): 1331-45.

[45] Leong E L, Khaing N T, Cadot B, et al. Nesprin-1 LINC complexes recruit microtubule cytoskeleton proteins and drive pathology in Lmna-mutant striated muscle [J]. Hum Mol Genet, 2023, 32(2): 177-91.

[46] Zhang Q, Ragnauth C D, Skepper J N, et al. Nesprin-2 is a multi-isomeric protein that binds lamin and emerin at the nuclear envelope and forms a subcellular network in skeletal muscle [J]. J Cell Sci, 2005, 118(Pt 4): 673-87.

[47] Rajgor D, Shanahan C M. Nesprins: from the nuclear envelope and beyond [J]. Expert Rev Mol Med, 2013, 15: e5.

[48] Zi-Yi Z, Qin Q, Fei Z, et al. Nesprin proteins: bridging nuclear envelope dynamics to muscular dysfunction [J]. Cell Commun Signal, 2024, 22(1): 208.

[49] Gallo S, Vitacolonna A, Bonzano A, et al. ERK: A Key Player in the Pathophysiology of Cardiac Hypertrophy [J]. Int J Mol Sci, 2019, 20(9).

[50] Mutlak M, Kehat I. Extracellular signal-regulated kinases 1/2 as regulators of cardiac hypertrophy [J]. Front Pharmacol, 2015, 6: 149.

[51] Ramos-Kuri M, Meka S H, Salamanca-Buentello F, et al. Molecules linked to Ras signaling as therapeutic targets in cardiac pathologies [J]. Biol Res, 2021, 54(1): 23.

[52] Sugiura R, Satoh R, Takasaki T. ERK: A Double-Edged Sword in Cancer. ERK-Dependent Apoptosis as a Potential Therapeutic Strategy for Cancer [J]. Cells, 2021, 10(10).

[53] Deschênes-Simard X, Malleshaiah M, Ferbeyre G. Extracellular Signal-Regulated Kinases: One Pathway, Multiple Fates [J]. Cancers (Basel), 2023, 16(1).

[54] Wu J, Chen Y, Li R, et al. Synergistic anticancer effect by targeting CDK2 and EGFR-ERK signaling [J]. J Cell Biol, 2024, 223(1).

[55] Li Y L, Cheng X N, Lu T, et al. Syne2b/Nesprin-2 Is Required for Actin Organization and Epithelial Integrity During Epiboly Movement in Zebrafish [J]. Front Cell Dev Biol, 2021, 9: 671887.

[56] Davidson P M, Battistella A, Dejardin T, et al. Nesprin-2 accumulates at the front of the nucleus during confined cell migration [J]. EMBO Rep, 2020, 21(7): e49910.

[57] Chen S N, Sbaizero O, Taylor M R G, et al. Lamin A/C Cardiomyopathy: Implications for Treatment [J]. Curr Cardiol Rep, 2019, 21(12): 160.

[58] Nicolas H A, Hua K, Quigley H, et al. A CRISPR/Cas9 zebrafish lamin A/C mutant model of muscular laminopathy [J]. Dev Dyn, 2022, 251(4): 645-61.

Manuscript

Click here to access/download;Manuscript;renamed_a3a80.docx

Exploring the Role of Nesprin-1 in Isoproterenol-Induced Heart Failure Insights into Pathogenesis and Therapeutic Implications

Qin Qin[1,2#] , Dong Cengyuan[1,2#] , Zhou Zi-yi[1,2] ,Liu Yangyuanzhi[1,2], Cao Chunyu [2,4], TENG Lin[1,3*]

1. Department of Cardiology, Yichang Central People's Hospital/The First Clinical Medical College, Three Gorges University, Yichang 443003, Hubei, People's Republic of China

2. School of Basic Medicine, China Three Gorges University, Yichang 443000, Hubei, People's Republic of China

3. King’s College London British Heart Foundation Centre of Research Excellence, School of Cardiovascular and Metabolic Medicine & Sciences, London SE5 9NU, U.K

4. College of Basic Medical Sciences, Hubei Key Laboratory of Tumor Microencironment and immunotherapy, China Three Gorges University, Yichang 443000, Hubei, People's Republic of China

# Qin Qin and Dong Cengyuan have equally to this work.

* Correspondence to: TENG Lin, MD, Department of Cardiology, Yichang Central People's, NO,183 Yiling Road, Yichang 443003, Hubei, People's Republic of China. E-mail address: tenglin@ctgu.edu.cn

Objective: In this study, we used an isoprenaline-induced heart failure model to investigate the role of Nesprin-1 in the development and progression of heart failure and to clarify the underlying molecular mechanisms.

Methods: A mouse model of heart failure was induced by daily intraperitoneal injection of isoproterenol (5 mg/kg/day) for 24 consecutive days. The body weight of mice was monitored daily. The diastolic left ventricular diameter (LVDd), systolic left ventricular diameter (LVDs), ejection fraction (EF) and short axis shortening rate (FS) of mice were evaluated by echocardiography of small animal hearts. The heart failure model was validated by gross cardiac observation, WGA staining, and Western blot analysis of B-type Natriuretic Peptide (BNP) expression. Hematoxylin-Eosin staining of heart tissue was performed to assess myocardial structure, cell morphology and pathological changes. Quantitative determination of malondialdehyde (MDA) and superoxide dismutase (SOD) expression was performed to evaluate oxidative stress levels. The expression of Nesprin-1 and connexin 43 (Cx43) was detected by immunofluorescence staining. Immunofluorescence staining and Western blot were used to detect the effects of Nesprin-1 expression changes on p53-binding protein 1 (53BP1), phosphorylated histone H2AX (γH2AX), H3 lysine 9 trimethylation (H3K9me3), H3 lysine 27 trimethylation (H3K27me3) expression.

In addition, we used Western blot to analyze the expression of the extracellular regulatory protein kinase (ERK) pathway.

Results: Compared with the control group, mice in the isoproterenol-induced heart failure model group exhibited significant increases in both body weight and heart weight ($P<0.01$), enlargement of cardiomyocyte cross-sectional area ($P<0.01$), markedly elevated BNP expression ($P<0.01$). The echocardiographic results showed that the LVDd and LVDs in diastolic phase were significantly increased, and EF and FS were significantly decreased in mice with heart failure($P<0.01$). Histopathological analysis demonstrated disordered myocardial structure, vascular proliferation, and myocardial hemorrhage in model group mice. The expression of Nesprin-1 was significantly downregulated in the model group ($P<0.01$), accompanied by abnormal nuclear envelope localization that resulted in distorted nuclear morphology of cardiomyocytes, including increased nuclear area and perimeter, elevated nuclear aspect ratio ($P<0.01$) , and decreased nuclear circularity($P<0.01$). Additionally, the model group showed significantly increased levels of the oxidative stress marker MDA coupled with decreased SOD activity ($P<0.05$), reduced Cx43 expression ($P<0.01$), upregulated expression of DNA damage markers (γH2AX and 53BP1)($P<0.01$), significantly elevated levels of H3K9me3 and H3K27me3 ($P<0.01$), and markedly enhanced activation of the ERK signaling pathway ($P< 0.01$).

Conclusion: This study revealed that downregulation of Nesprin-1 may serve as a crucial driver in the progression of heart failure. In the isoproterenol-induced murine heart failure model, reduced Nesprin-1 expression leads to multiple pathological alterations, including nuclear homeostasis imbalance, decreased Cx43 expression, elevated oxidative stress, accumulated DNA damage, and disordered epigenetic regulation. The underlying mechanism may involve activation of the ERK signaling pathway.

Keywords: Heart failure, Nesprin-1, Isoproterenol, Cardiac remodeling, Dysfunction, Epigenetic regulation, DNA damage

Introduction

Heart failure (HF), or cardiac failure, signifies the heart's diminished capacity to efficiently pump blood, resulting in venous congestion and inadequate arterial perfusion due to compromised systolic and/or diastolic functions[1-4]. This condition represents the terminal phase of heart disease and stands as a leading cause of cardiovascular-related mortality worldwide. HF affects approximately 1% to 2% of the global population, with a notably higher prevalence among the elderly[5-7]. Sympathetic nervous system (SNS) overactivity is a hallmark of HF, correlating with disease severity and prognosis. Under normal circumstances, the cardiac sympathetic nervous system enhances cardiac output, gene transcription, and metabolism by releasing neurotransmitters and engaging β-adrenergic receptors (β-AR)[8, 9]. However, excessive SNS activation detrimentally affects the cardiovascular system, potentially precipitating or exacerbating HF[10, 11]. This overdrive leads to heightened heart rate and contractility, inducing a sustained high-load state that depletes cardiac energy reserves, impeding cardiac function. Furthermore, it promotes adverse structural cardiac changes such as ventricular hypertrophy and atrial dilation, diminishing cardiac pump efficiency[12-14]. Increased arterial vasoconstriction further strains the heart by elevating vascular tension. Excessive sympathetic stimulation also incites inflammation and oxidative stress, damaging myocardial cells and hastening disease progression. Disruption of neuroendocrine system balance, including the renin-angiotensin-aldosterone system, can exacerbate fluid retention and increase blood volume, exacerbating cardiac workload[15-18]. Current HF management strategies primarily target the sympathetic nervous system with medications like β-blockers, angiotensin-converting enzyme inhibitors, angiotensin II receptor blockers, and aldosterone receptor antagonists. Despite these interventions, HF incidence, readmission rates, and mortality remain high, underscoring the need for novel therapeutic approaches[19-23].

Traditionally, HF research focused on intracellular factors, neglecting the nuclear envelope's role. The nuclear envelope (NE) is pivotal, spatially segregating nuclear genetic material and regulating macromolecular transport[24]. The Linker of

Nucleoskeleton and Cytoskeleton (LINC) complex, anchored to the NE, includes Nesprin proteins, which bridge the nuclear and cytoskeletal proteins, contributing to cellular structure and function. Nesprin mutations are implicated in various diseases, including Dilated Cardiomyopathy (DCM) and Emery-Dreifuss Muscular Dystrophy (EDMD), highlighting their importance in muscle physiology[25-27]. Nesprin family proteins, integral to the NE, include Nesprin-1 and Nesprin-2, which interact with actin, intermediate filaments, and the nuclear envelope, influencing cell signaling, morphology, and mechanical properties[28]. These proteins regulate gene expression, chromatin structure, and epigenetic modifications, impacting cell function and pathology. Mutations in Nesprin-1 and Nesprin-2 are associated with abnormal nuclear morphology, positioning, and chromatin organization, contributing to DCM and EDMD. Nesprin alterations also affect intercalated disc protein localization, leading to conduction defects in Nesprin-associated cardiomyopathies[27, 29, 30].

Given this background, we hypothesize that the dysregulation of Nesprin-1 expression disrupts the structural connection between the nucleus and the cytoskeleton, thereby impairing nuclear homeostasis, interfering with signal transduction and causing abnormal transcription regulation, which ultimately leads to myocardial remodeling and contractile dysfunction, and then heart failure. Our study aims to elucidate Nesprin-1's role and molecular mechanisms in HF, providing insights for diagnosis and treatment. Understanding Nesprin-1's function may reveal therapeutic targets, guiding future HF therapies.

Materials and methods

Drugs and Chemicals

MedChemExpress provided isoproterenol (HY-B0468) and isoflurane, while Biosharp supplied 4% paraformaldehyde (BP003), hematoxylin-eosin staining (BP019), and other staining-related reagents. Sinopharm Chemical Reagent offered basic reagents such as paraffin (69019361) and xylene (10023418). Antibodies were mainly sourced from Boster, including Cx43 antibody (A00599), BNP antibody (M01186-3), 53BP1 antibody (BM5099), γH2AX antibody (BM4148), H3K9me3 antibody (BM5079), H3K27me3 antibody (BM4341), and ERK antibody (BM4156).

Protein-related reagents were primarily obtained from Beyotime, including RIPA lysis buffer (P0013B) and BCA protein assay kit (P0010). Additionally, Amresco provided electrophoresis reagents such as TEMED (Amresc00761) and acrylamide (Exp2016109), while Jackson supplied HRP-labeled secondary antibodies, specifically HRP-conjugated goat anti-rabbit (BA1054), HRP-conjugated rabbit anti-goat (BA1060), HRP-conjugated goat anti-mouse (BA1051), and HRP-conjugated goat anti-rat (BA1058). Other essential reagents included WGA stock solution (BBL-0351) from Sigma-Aldrich, DAPI stock solution (C1002) from Solarbio, and cytoplasmic/nuclear protein extraction kit (KGP150) from Nanjing KeyGen Biotech.

Establishment of Isoproterenol-Induced Heart Failure Model in Mice

The male C57BL/6 mice weighing 20-22g were purchased from the Animal Experimental Center of Three Gorges University. All mice were housed in specific pathogen-free (SPF) animal facilities at Three Gorges University (Yichang, Hubei, China). The experimental animal quality certification number is 42010200009428. The experimental animal use permit number is 00305510. Twenty-four C57BL/6 mice were randomly divided into two groups: model group and control group. In the model group, mice were administered intraperitoneal injections of isoproterenol (ISO) at a dosage of 5mg/kg. In the control group, mice were administered intraperitoneal injections of an equivalent volume of medical-grade physiological saline. Two groups received intraperitoneal injections continuously for 24 days.

Cardiac Function Assessment

Mice were anesthetized with isoflurane, and after achieving stable anesthesia, the hair in the chest area was removed. The mice were then placed in a supine position and fixed on an ultrasound examination table. Ultrasound coupling agent was applied to the chest area. Using small animal ultrasound, M-mode images of the long axis and short axis of the mouse heart were obtained. Parameters including ejection fraction (EF), fractional shortening (FS), left ventricular end-diastolic diameter (LVEDd), and left ventricular end-systolic diameter (LVESd) were measured and calculated to assess cardiac function in mice. Statistical analysis was performed to analyze the cardiac

function of mice.

Measurement of Body and Heart Weight

Body weight of the mice was measured before each dose.At least 24 hours after the last dose the mice were anesthetized, and their chests were swiftly opened using surgical scissors and forceps to extract the hearts. The hearts were rinsed in pre-cooled physiological saline, and then scissors were used to trim the blood vessels and connective tissues surrounding the heart. The hearts were then rinsed again with fresh physiological saline and dried with filter paper. Gross images of the hearts were captured, and the total heart weight was measured. Some samples were frozen in dry ice and stored in-80℃ refrigerator, while others were fixed in 4% polyformaldehyde fixative for easy observation of histological morphology.

Pathological Section Staining

HE staining: After dehydration, embedding, and sectioning of tissue fixed with 4% polyformaldehyde, the paraffin sections are dewaxed to water. Stain with hematoxylin for 4 minutes, followed by 2-minute rinsing with tap water to remove excess stain. The sections are then differentiated with 0.8% hydrochloric acid alcohol for 2 seconds, rinsed again with tap water, immersed in eosin solution for 20 seconds, adjusted with 95% ethanol for 5 seconds, and dehydrated with anhydrous ethanol for 2 minutes. Finally, the sections are sealed with a transparent environmental agent and observed under a microscope.

WGA staining: The paraffin sections were sequentially dewaxed with eco-friendly degreasing agents (1), (2), and (3) for 10 minutes each, followed by 5-minute immersion in anhydrous ethanol, 95% ethanol, and 75% ethanol respectively. The sections underwent three 3-minute rinses with distilled water. Antigen retrieval was performed by high-pressure treatment of the sections in boiling citric acid (pH6.0) solution for 2 minutes. After cooling, the sections were immersed in distilled water for three 5-minute washes. A circular mark was made on the section using a histological pen, then it was immersed in TBST buffer. A 1:200 diluted WGA working solution (50 uL/section) was added and incubated under light protection for 30 minutes, followed by three 5-minute TBST washes. After removing TBST, a 1:500 diluted

DAPI working solution (50 uL/section) was applied to stain nuclei for 5 minutes. Following TBST rinsing, the sections were mounted with anti-fluorescence quenching medium and stored at 4℃ under light protection. Finally, microscopic examination and image analysis were conducted.

The biochemical kits detects the oxidative stress indicators

Sample Preparation: Myocardial tissue was mixed with PBS buffer at ratios of 1:10 (for MDA detection) and 1:9 (for SOD detection), homogenized, and centrifuged at 10,000 g for 15 min at 4 ℃. The supernatant was collected for subsequent analysis.

Reagent Preparation: MDA detection working solution (including probe working solution and standard) and SOD detection working solution (including enzyme reaction solution) were prepared according to the kit instructions.

MDA Measurement: The sample was mixed with the detection working solution, incubated at 95 ℃ for 40 min, cooled on ice for 5 min, and centrifuged at 10,000 g for 10 min. The supernatant was measured at 532 nm absorbance.

Calculation formula:

MDA(umol/gprot)=[(A_{sample}-A_{blank}) /(A_{standard}-A_{blank})]×C_{standard}×dilution factor / protein concentration

SOD Measurement: 20 uL of sample was added, incubated at 37 ℃ for 20 min, and measured at 450 nm absorbance.

Calculation formulas:

SOD inhibition rate (%) = [(ΔA_{control} - ΔA_{sample}) / ΔA_{control}] × 100%

SOD activity (U/mg prot) = Inhibition rate / (100% - Inhibition rate) × 12 / protein concentration

(Note: In the formulas, C_{standard} is the standard concentration, ΔA is the absorbance difference, and 12 is the conversion factor for reaction volume/sample volume.)

Immunofluorescence Staining Protocol for Mouse Cardiac Tissue

Initially, tissues fixed with 4% paraformaldehyde were processed through dehydration,

embedding, and sectioning, followed by dewaxing the paraffin sections to water. Antigen retrieval was then performed by incubating the sections in preheated 95° C sodium citrate buffer (pH 6.0) under high-pressure heating for 10 minutes. After natural cooling, the sections were washed three times with phosphate-buffered saline (PBS), 3 minutes each. Excess moisture was removed using absorbent paper, and the tissue areas were circled with an immunohistochemical pen before applying diluted normal goat serum for 30-minute blocking at room temperature. After blocking, excess liquid was removed (without washing), and diluted primary antibody was directly applied for overnight incubation at 4 ℃ in a humidified chamber. The following day, sections were thoroughly washed with PBS (⩾3 times, 3 minutes each), dried, and treated with diluted fluorescent secondary antibody for 1-hour incubation at 25 ℃ in a humidified chamber, followed by four additional PBS washes (3 minutes each). For nuclear staining, DAPI was applied under light-protected conditions for 5 minutes, followed by four PBS washes (5 minutes each) to remove excess dye. Finally, sections were dried and mounted with anti-fade mounting medium for observation and image acquisition under a fluorescence microscope.

Western Blot Experimental Procedure

Protein extraction was first performed by mincing tissue samples in a 2mL homogenizer, followed by adding 400 uL lysis buffer containing PMSF for ice-cold homogenization with repeated processing at 5-minute intervals until complete tissue disruption was achieved. After 30 minutes of lysis, the lysate was centrifuged at 12,000 rpm for 5 minutes at 4 ℃, and the supernatant was aliquoted and stored at -20 ℃. Protein concentration was determined using the BCA method: samples were diluted 1:19 with PBS, and a standard curve (0.2-1mg/mL BSA) was prepared and loaded onto a 96-well plate alongside samples (standards in duplicate, samples in triplicate). After adding BCA working solution, the plate was incubated at 37° C for 30 minutes protected from light, and absorbance at 568 nm was measured to calculate protein concentration. Protein samples were mixed with 5×loading buffer and denatured by boiling for 10 minutes before storage.

For electrophoresis, separating and stacking gels were prepared, and 40ug total protein was loaded per well. Initial electrophoresis was performed at 80V until samples entered the separating gel, then switched to 120V for approximately 1.5 hours until the bromophenol blue reached the gel bottom. Prior to transfer, PVDF membranes were cut and activated with methanol, and the transfer sandwich was assembled in the order of "negative plate-fiber pad-filter paper-gel-membrane-filter paper-fiber pad-positive plate" for constant current transfer. After transfer, membranes were blocked with 5% skim milk in TBST (2% BSA for phosphoproteins) at room temperature for 2 hours.

Following overnight primary antibody incubation at 4 ℃, membranes were washed 6 times with TBST, then incubated with HRP-conjugated secondary antibody at 37 ℃ for 2 hours, followed by 5 additional TBST washes. For ECL detection, enhanced solution and peroxidase solution were mixed in equal proportions and applied to membranes. After several minutes of reaction, X-ray films were exposed and processed through development and fixation to obtain results. Throughout the procedure, care was taken to maintain samples at low temperatures, ensure bubble-free transfer, and keep membranes moist during antibody incubations.

Statistical analysis

All quantitative data were expressed as mean ± standard deviation (X±SD). The comparison between the two groups was evaluated using the t-test. *P* value <0.05 indicated statistically significant differences. The data were analyzed using GraphPad Prism 10.0 software.

3.Result

1.Establishment and Validation of Isoproterenol-Induced Heart Failure Mouse Model

Echocardiographic evaluation revealed notable alterations in cardiac parameters across the two experimental groups(Fig1A-B). Mice in the model group displayed significant increases in left ventricular end-diastolic diameter (LVEDd) and left ventricular end-systolic diameter (LVESd) compared to the control groups(Fig1C-D)(*P*<0.01). Conversely, ejection fraction (EF) and fractional

shortening (FS) were markedly reduced in the model group(Fig1E-F)(P<0.01). These findings underscore the effectiveness of the isoproterenol-induced myocardial hypertrophy model in inducing structural and functional changes in cardiac function.

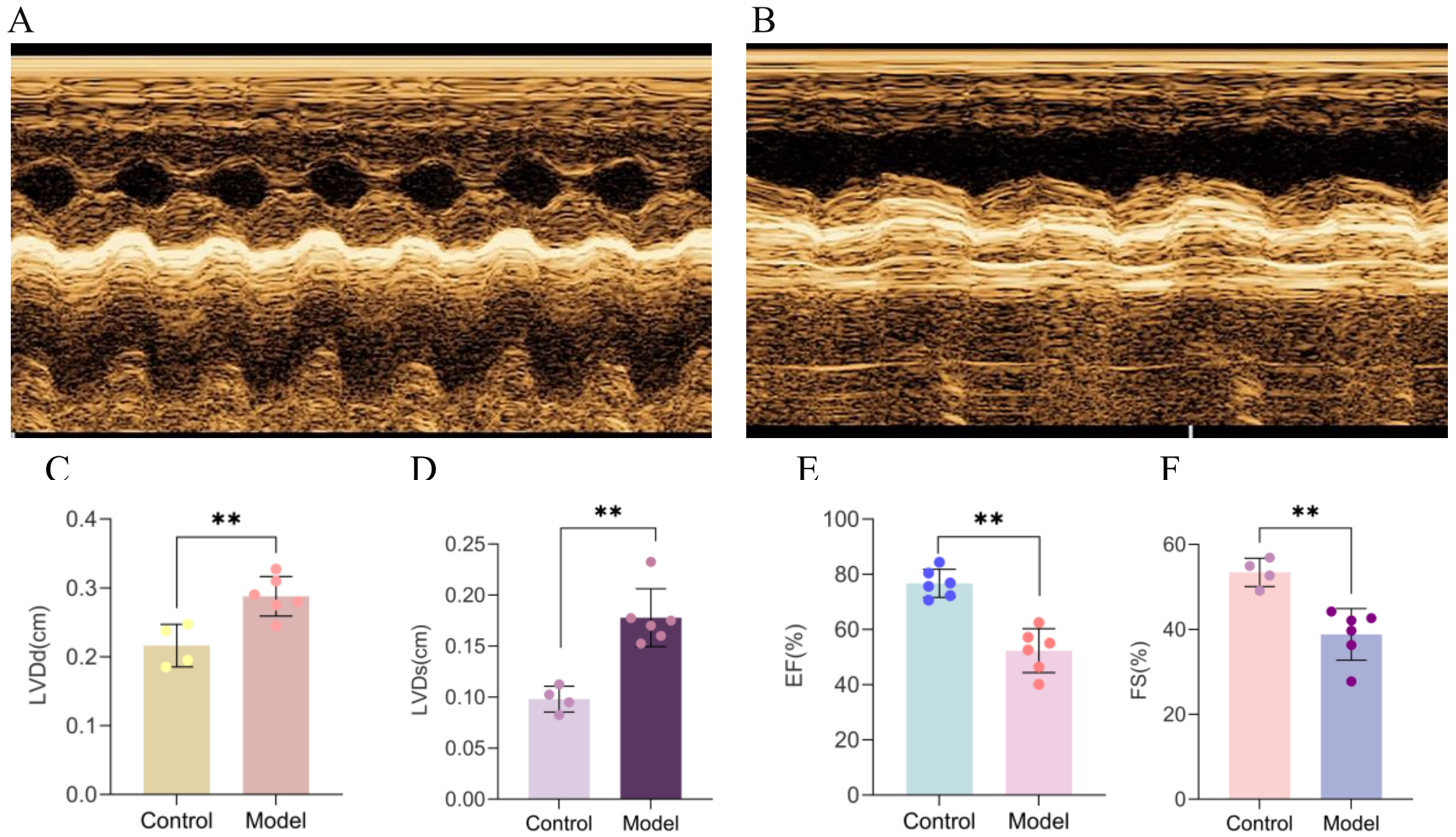

Figure 1: Isoproterenol-induced Decrease in Cardiac Function in Mice. A: Echocardiogram of the control group mice; B: Echocardiogram of the model group mice; C-F: Left ventricular diameter (LVDd), left ventricular diameter at systole (LVDs), ejection fraction (EF), and short-axis shortening rate (FS) of the left ventricle in mice; n≥4; Compared to the control group, **P<0.01

Assessment of cardiac weight provided additional insights into physiological changes among the experimental groups. Mice in the isoproterenol-induced heart failure model group exhibited a significant increase in body weight starting from day 13 of drug administration, compared to controls(Fig2A). Moreover, body weight and cardiac weight significantly increased in the model group after 24 days of isoproterenol administration, indicative of myocardial hypertrophy(Fig2B-C)(P<0.01). WGA staining demonstrated a significant increase in cardiomyocyte cross-sectional area in the model group compared to controls(Fig2D-E)(P<0.01).By examining BNP in both groups using Western Blot, we found that BNP was significantly elevated in the model group compared to the control group(Fig2F-G)(P<0.01). These findings highlight the capacity of isoproterenol to induce cardiac hypertrophy in mice.

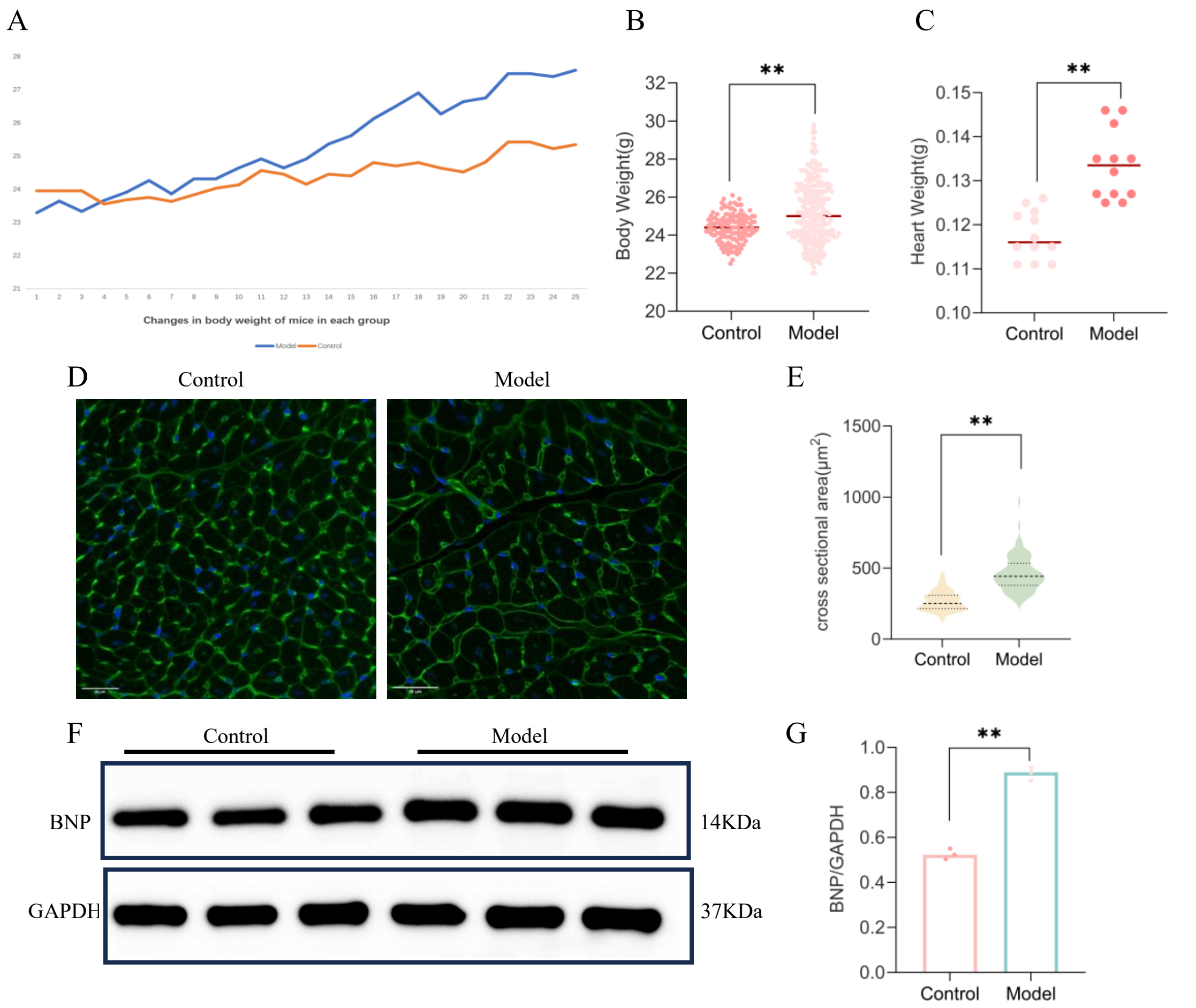

Figure2：Isoproterenol-induced heart failure in mice. A: Graph of changes in mouse body weight; B-C: Body and cardiac weights of the two groups of mice; n=12; D-E: Myocardial WGA staining of mice; n=5; F-G: BNP protein expression levels of the two groups of mice; n=3. Compared with the control group, ***P*<0.01

Morphological and pathological staining of the heart in mice revealed significant differences in myocardial structure between the two groups (Figure 3A). Compared to the control group, the model group mice exhibited a significantly larger heart volume (Figure 3A). A comparison of cross-sectional images from the hearts of the two groups showed that the ventricular cavity in the model group mice was notably enlarged (Figure 3B). HE staining results indicated that the model group mice exhibited disordered myocardial structure, vascular proliferation, and myocardial hemorrhage (Figure 3C-D). These findings collectively highlight the pathological changes in myocardial hypertrophy induced by isoproterenol in mice.

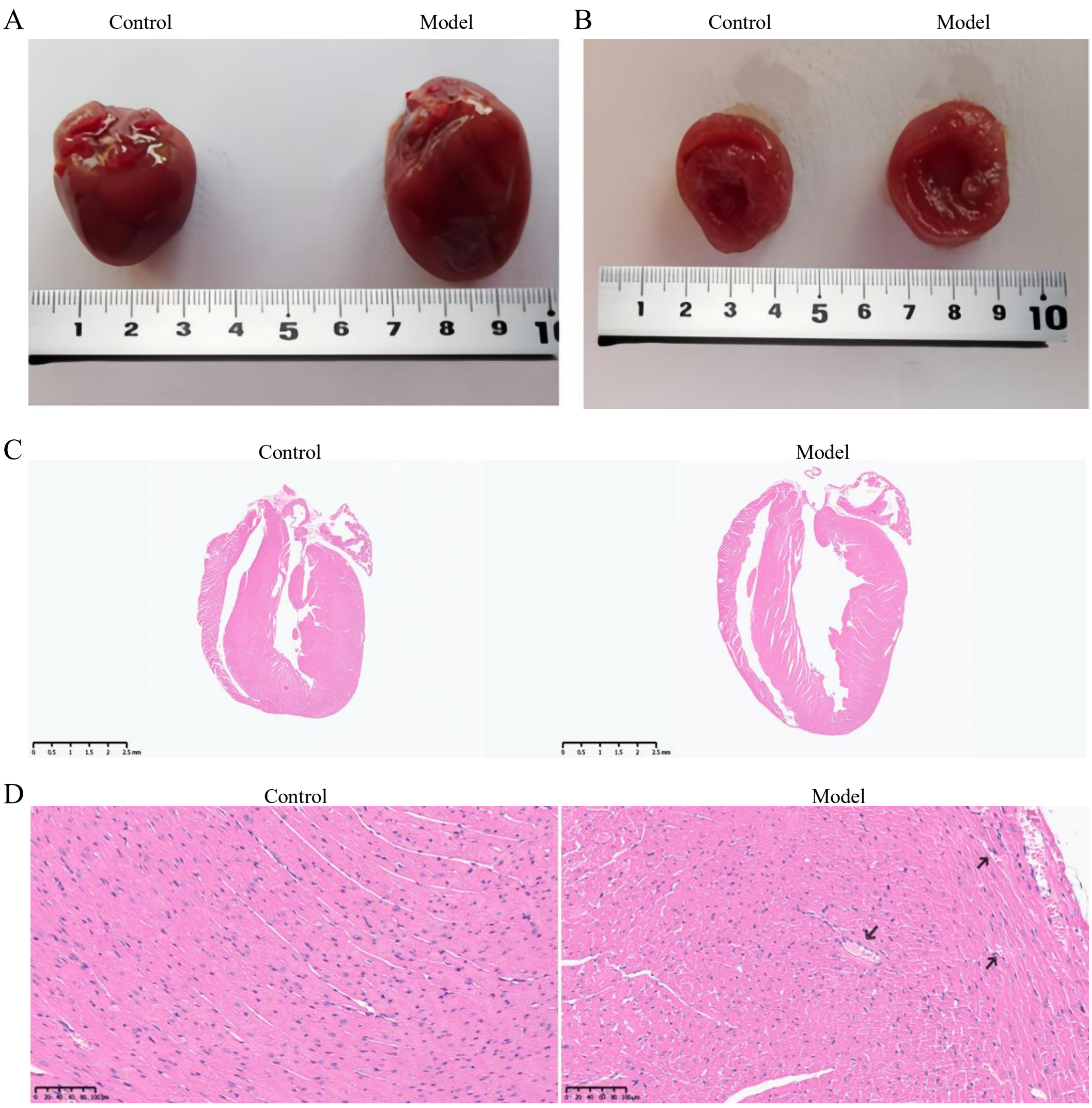

Figure 3: Gross view and pathological staining of mouse heart . A: Gross view of mouse heart; B: Coronary section of mouse heart; C-D: HE staining of mouse myocardial tissue; n=5

2.The expression of Nesprin-1 in the heart of mice with isoproterenol induced heart failure was significantly reduced

The immunofluorescence analysis revealed a significant decrease in Nesprin-1 expression in the hearts of mice induced with isoproterenol-induced heart failure(Fig4A)(P<0.01).By fluorescent staining of Nesprin-1 with specific antibodies, we observed that Nesprin-1 was mainly located at the nuclear membrane of cardiomyocytes (Fig4A). This localization is consistent with its function in maintaining nuclear membrane structure and nuclear-cell skeleton connection.

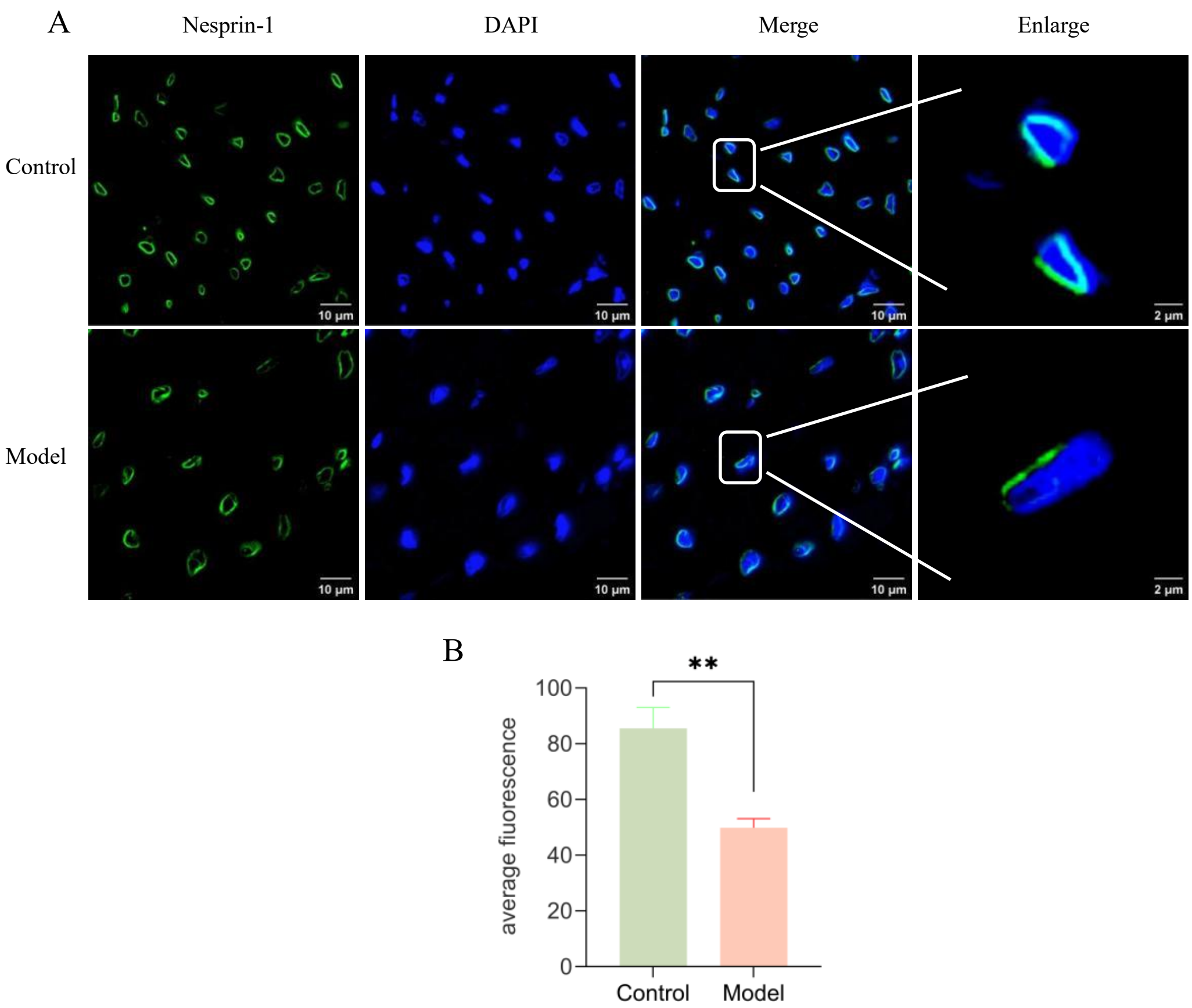

Figure 4: Expression changes of Nesprin-1 in myocardial tissue. A-B: Immunofluorescence staining of Nesprin in mouse myocardial tissue; n=5; Compared with the control group, ***P<0.01

3.Dysregulation of Nesprin-1 expression in cardiac tissue of isoproterenol induced heart failure mouse model affects nuclear homeostasis in heart failure

In our experimental study, we employed immunofluorescence to investigate the effects of Nesprin-1 expression changes on nuclear homeostasis in cardiomyocytes. By randomly selecting 40 fluorescently stained pathological sections from five model groups, we obtained 40 tissue micrographs. Ten nuclei were randomly selected from each image and analyzed using ImageJ software for morphological parameters including nuclear area, perimeter, and aspect ratio. Results demonstrated that compared with the control group, the nuclear morphology of cardiomyocytes in the model group exhibited significant abnormalities, characterized by marked increases in nuclear area, perimeter, and aspect ratio, along with a notable reduction in nuclear roundness (Figure 5A)(P<0.01). Furthermore, immunofluorescence staining revealed partial or complete absence of Nesprin-1 on the nuclear membrane in some cardiomyocytes of the model group (Figure 5B)(P<0.01). These findings indicate that

Nesprin-1 expression dysregulation affects nuclear homeostasis in ISO-induced HF mouse models, though the causal relationship between these two factors requires further investigation.

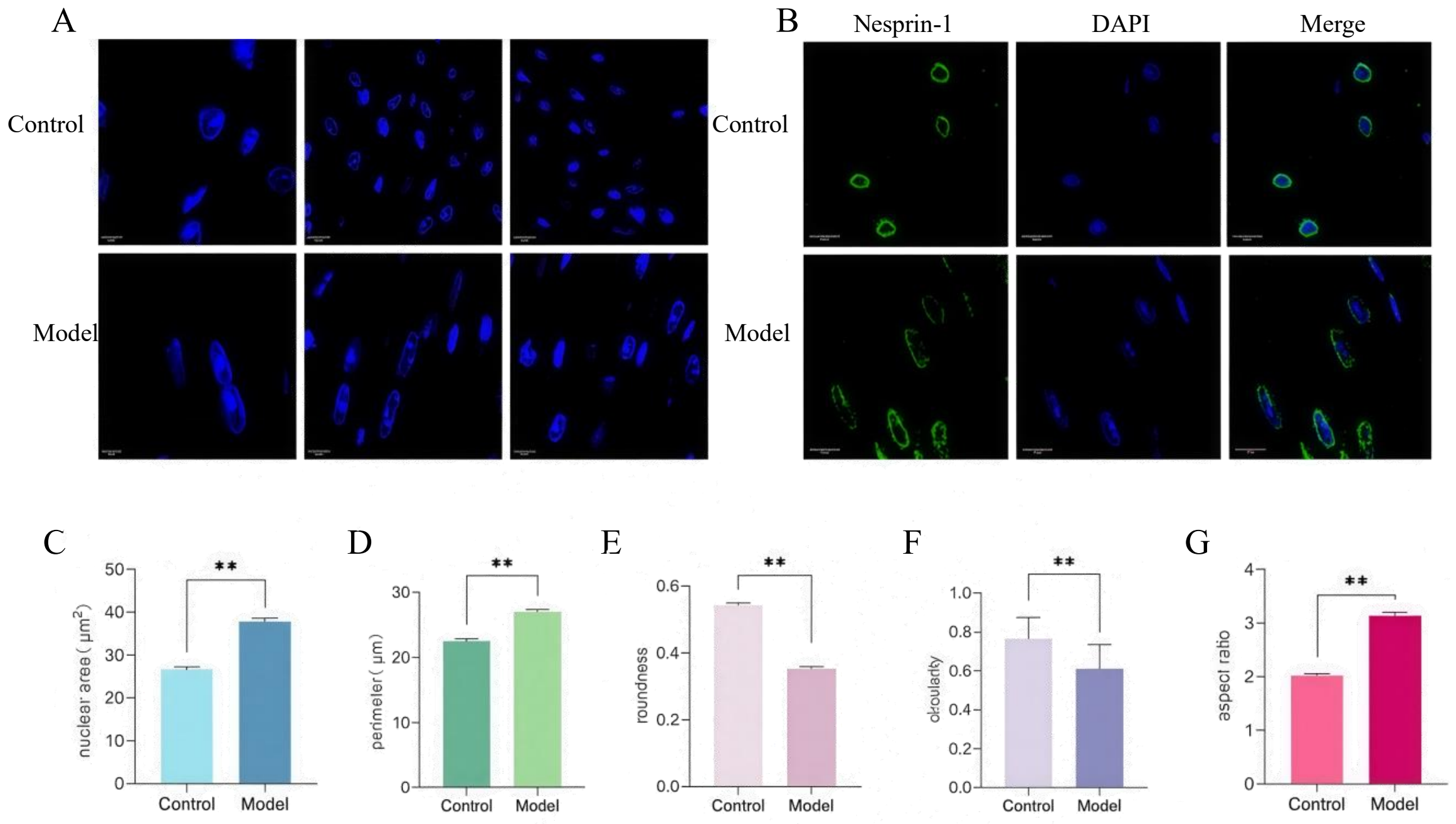

Figure 5: Nuclear Homeostasis Disruption in Heart Failure Mice. Note: A: Changes in nuclear morphology; B: Nesprin-1 immunofluorescence staining; C-G: Nuclear area, nuclear perimeter, nuclear roundness , and aspect ratio of the two groups of mice; n=5; Compared to the control group, ***P<0.01

4.In a mouse model of heart failure with reduced Nesprin-1 expression, oxidative stress responses were increased

Oxidative stress, characterized by an imbalance between oxidizing agents and antioxidant defenses, predisposes tissues to oxidation. This imbalance fuels neutrophilic inflammation, upregulates protease secretion, and fosters the generation of various oxidative intermediates. Acknowledged as a detrimental factor contributing to aging and various diseases, oxidative stress primarily stems from the overproduction of free radicals within the body. In line with this, Nesprin-1 depletion in Drosophila melanogaster has been correlated with mitochondrial dysfunction, thereby inducing oxidative stress[31]. To investigate this phenomenon, we evaluated the expression levels of malondialdehyde (MDA) and superoxide dismutase (SOD) in cardiac tissue. Notably, compared to the control groups, the model group exhibited heightened MDA activity and diminished SOD content(Fig6A-B)(P<0.05). These results indicate that oxidative stress levels were significantly increased and

antioxidant capacity was inhibited in the Nesprin-1 dysregulated HF model.

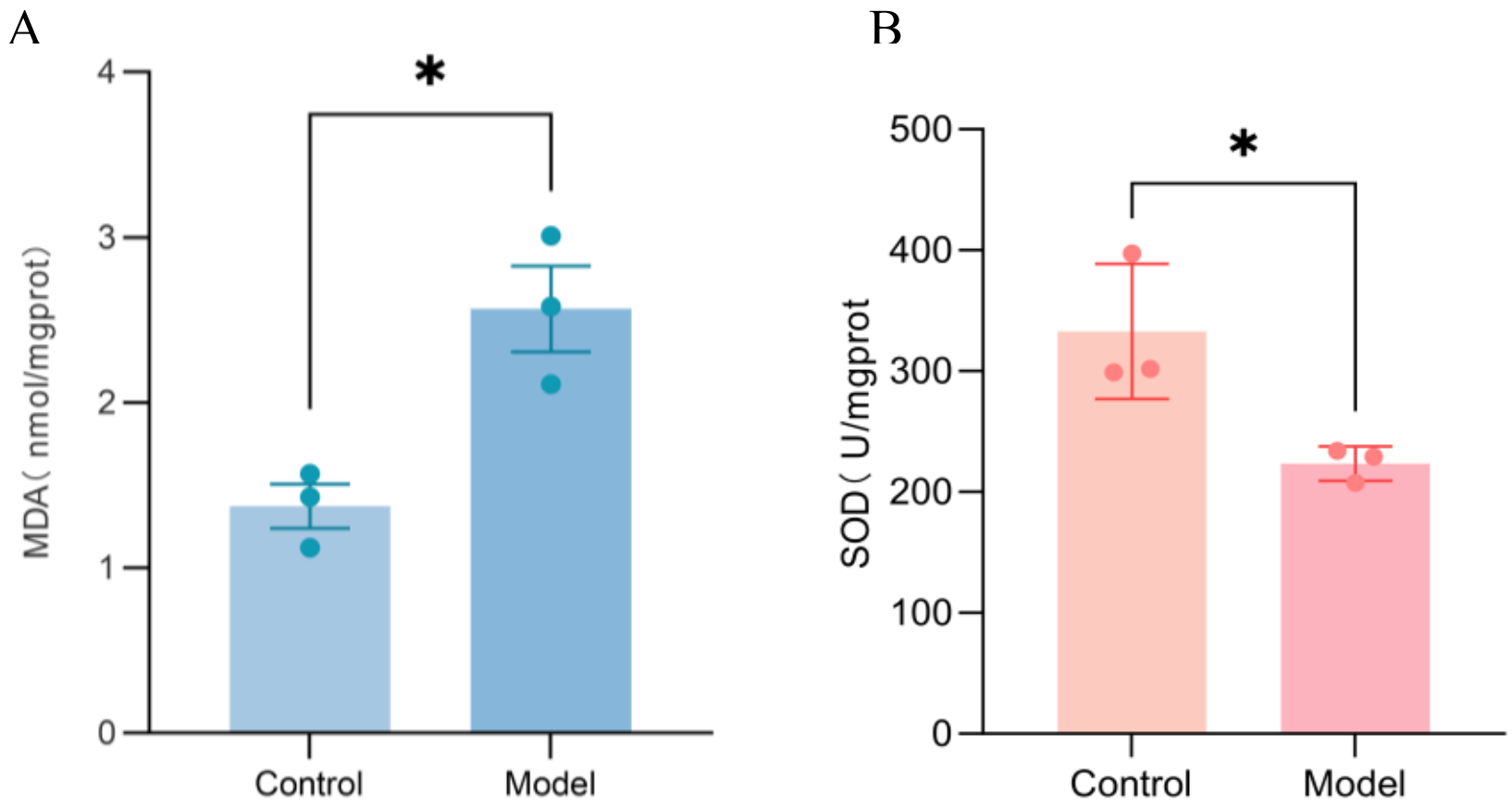

Figure 6: Oxidative stress aggravation in heart failure mice. A-B: Quantitative analysis of MDA and SOD in mouse myocardium; MDA: malondialdehyde; SOD: superoxide dismutase; n=3; compared with the control group, *P<0.05

5.The expression of Cx43 was downregulated in the myocardium of mice with dysregulation of Nesprin-1 expression in heart failure

Conduction dysfunction is a hallmark feature observed in the isoproterenol-induced heart failure mouse model. Cx43, primarily localized at the intercalated discs within mature cardiac myocytes, plays a pivotal role in facilitating electrical conduction among myocytes and enabling chemical signaling and energy substrate exchange, essential for coordinating cellular function[32]. Through immunofluorescence analysis, we investigated the expression levels of Cx43 in mice from each experimental group.Our results demonstrate a significant reduction in Cx43 expression specifically within the model group(Fig7A-B)(P<0.01). In addition, combined with our previous studies, this result will provide a reliable experimental basis for our further study on how the reduction of Nesprin-1 expression affects the localization and function of Cx43 and jointly participates in the occurrence of conduction dysfunction in ISO-induced HF model.

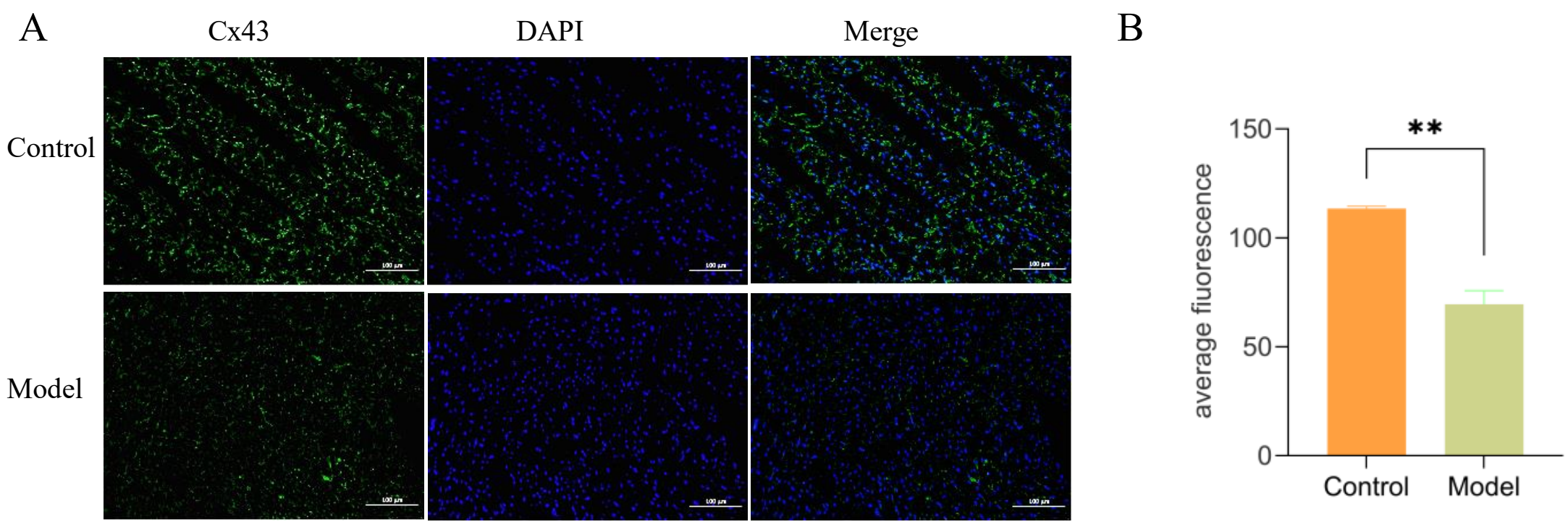

Figure 7: Expression of Cx43 in both groups of mice. A: Cx43 expression was detected by immunofluorescence; B Quantitative analysis of the Cx43; n=5; compared with the control group, ***P*<0.01

6.The myocardial DNA damage was exacerbated in mice exhibiting Nesprin-1 expression dysregulation during heart failure

Decreased Nesprin expression is associated with activated cellular DNA damage in the isoproterenol-induced heart failure mouse model. Nesprin plays a crucial role in maintaining genomic integrity, and its reduction may lead to impaired DNA repair mechanisms[33]. To investigate this phenomenon, immunofluorescence and Western blot analyses were conducted to assess the expression levels of 53BP1 and γH2AX in cardiac tissue. Our results reveal an increased expression of 53BP1 and γH2AX in the model group compared to the control groups(Fig8A-D)($P<0.01$). In agreement with this, Western blot indicated elevated levels of DNA damage in Nesprin-deficient hearts(Fig9A-C)($P<0.01$).These results suggest that the decrease in Nesprin-1 expression may interact with the accumulation of DNA damage in the occurrence of HF.

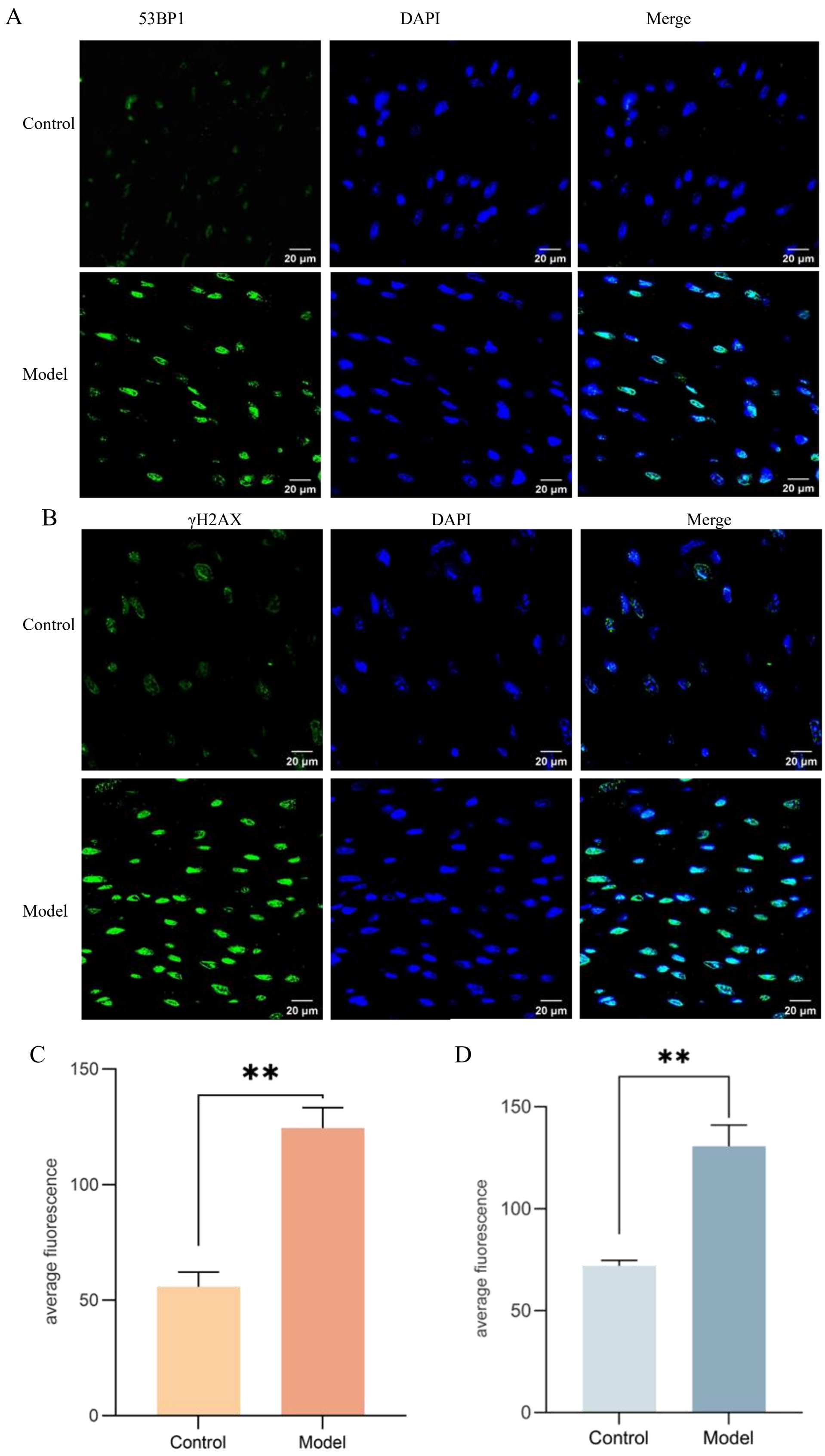

Figure 8: Increased DNA damage in myocardium of mice with heart failure. A-D: Detection of expression of 53BP1 and γH2AX by immunofluorescence; 53BP1: P53 binding protein 1; γH2AX: phosphorylated histone; n=5; compared with the control group,***P*<0.01

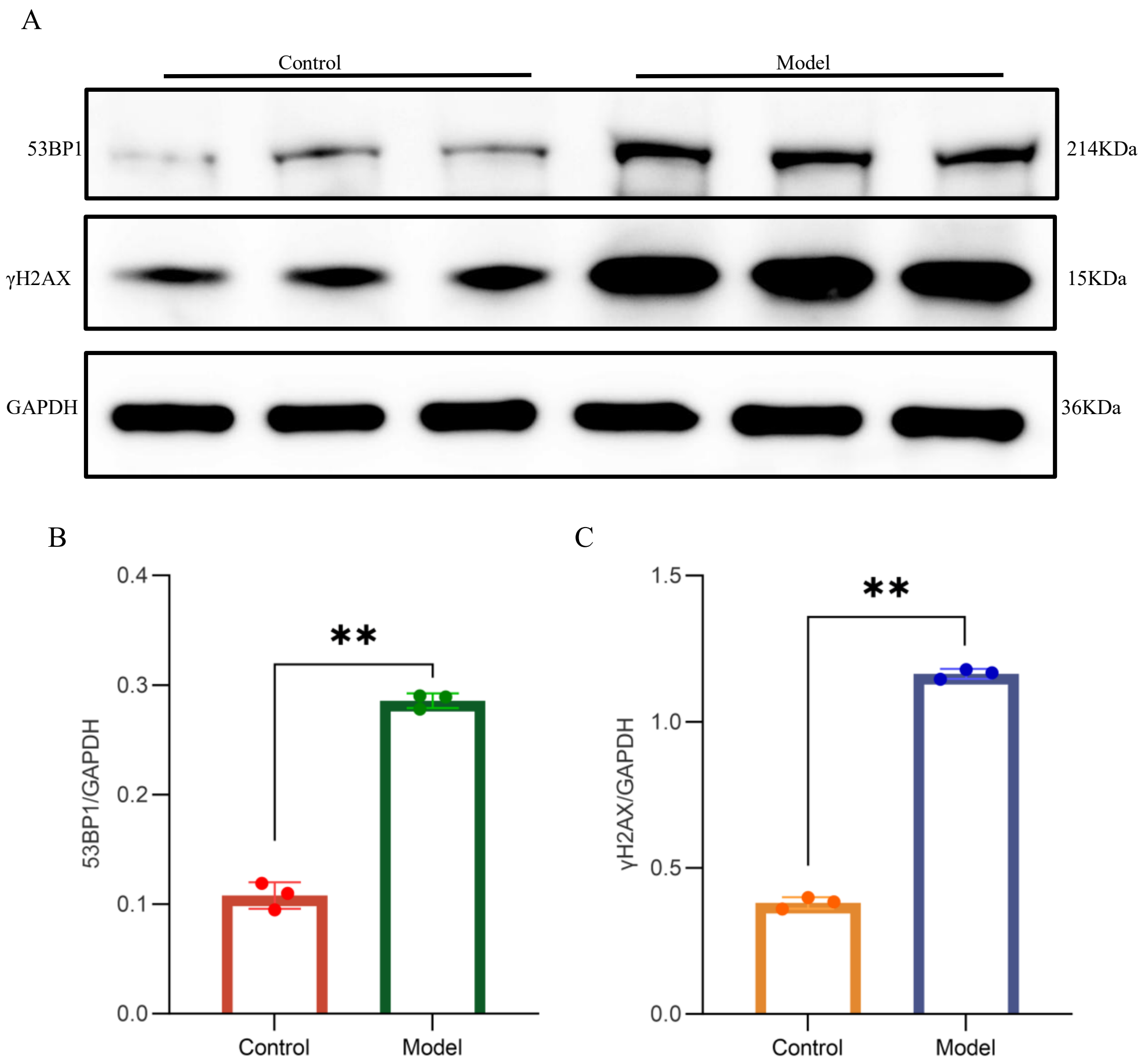

Figure 9: Increased DNA damage in myocardium of mice with heart failure; A-C: Protein quantitative analysis of 53BP1 and γH2AX; 53BP1: P53 binding protein 1; γH2AX: phosphorylated histone; n=3; compared with the control group,***P*<0.01

7.Dysregulation of Nesprin-1 interferes with histone methylation in heart failure

Histone methylation represents a critical modification that alters chromosome structure. Recent studies have highlighted the pivotal role of histone methylation modifications in myocardial hypertrophy, fibrosis, and cardiac function[34, 35]. Through immunofluorescence and Western blot analysis, we assessed the expression levels of H3K9me3 and H3K27me3 in mice from each experimental group. As depicted in the figures, the model group exhibited significantly increase expression levels of H3K9me3 and H3K27me3 compared to the control groups(Fig10A-D)(P<0.01). This result is consistent with the Western blot results(Fig11A-C)(P<0.01). These findings suggest that the histone methylation pattern disorder in the isoproterenol induced heart failure mouse model may be associated with the decreased level of Nesprin-1.

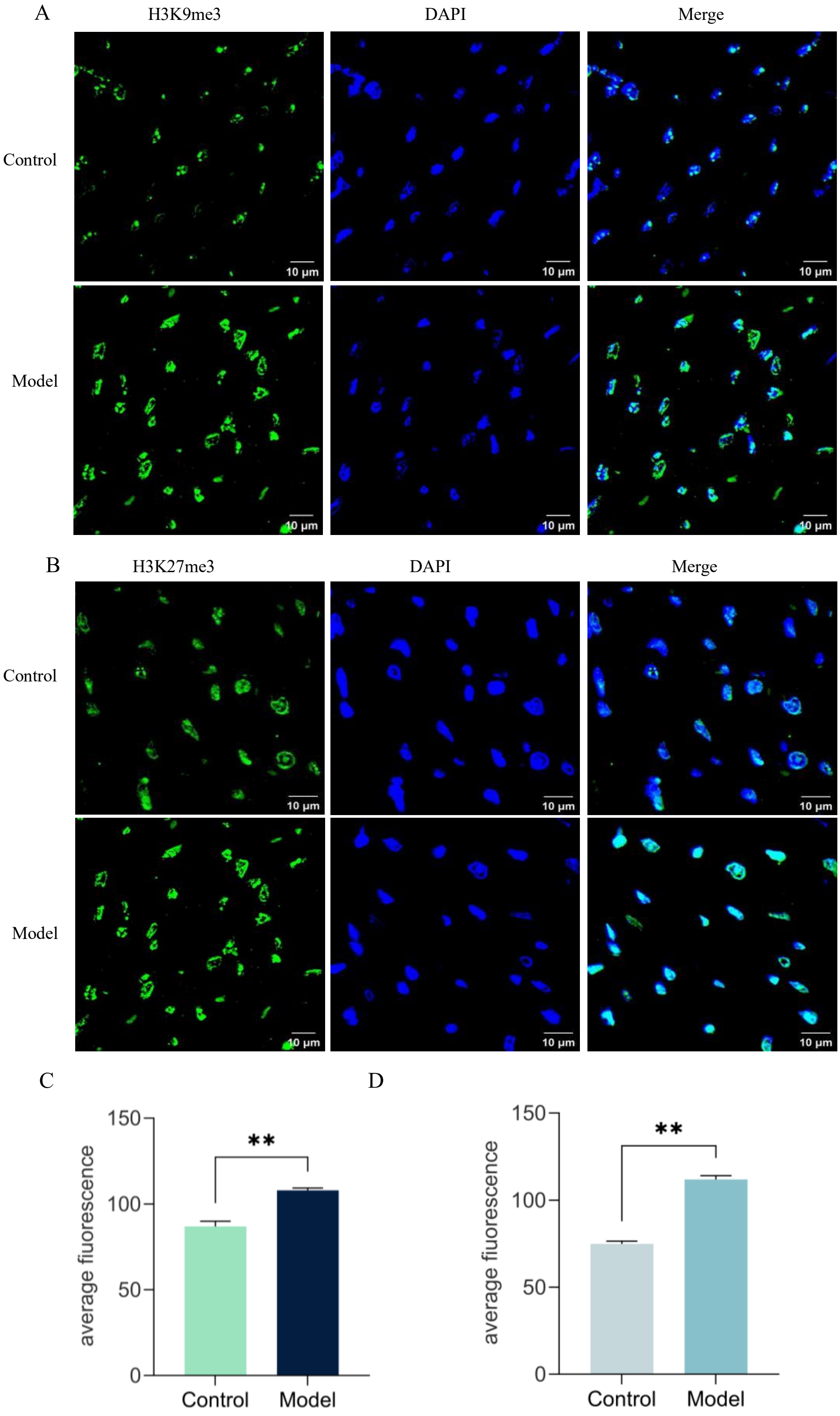

Figure 10: Changes in chromatin recombination in the myocardium of heart failure mice.A-D: Immunofluorescence detection of H3K9me3 and H3K27me3 expression; H3K9me3: trimethylation of histone H3 lysine 9; H3K27me3: trimethylation of histone H3 lysine 27; n=5; compared to the control group,***P*<0.01

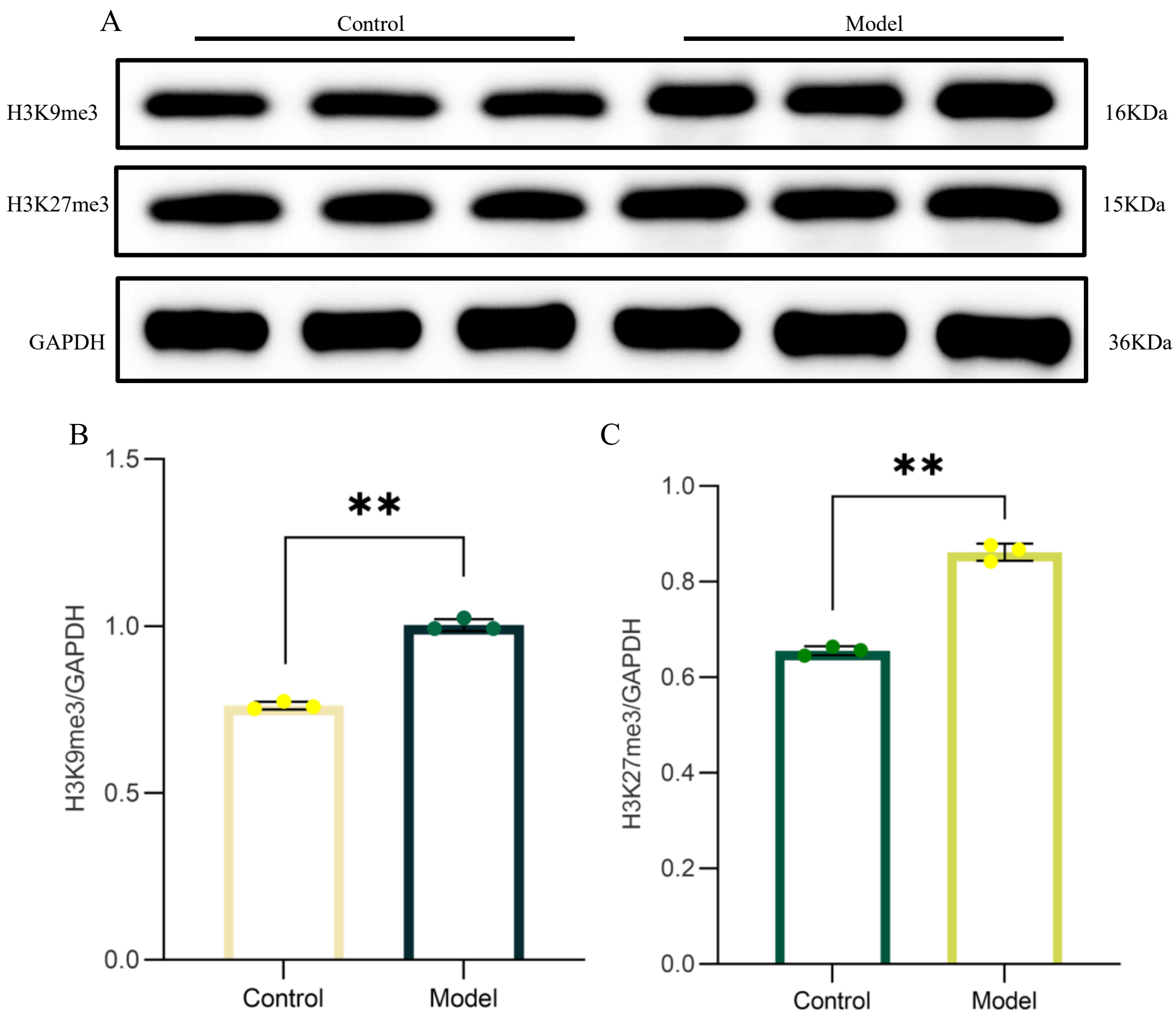

Figure 11: Changes in chromatin recombination in the myocardium of heart failure mice. A-C: Quantitative analysis of H3K9me3 and H3K27me3 proteins; H3K9me3: trimethylation of lysine 9 on histone H3; H3K27me3: trimethylation of lysine 27 on histone H3; n=3; compared with the control group,***P*<0.01

8.ERK pathway is activated in nuclear homeostasis abnormalities mediated by Nesprin-1 expression disorder and heart failure

The ERK pathway is recognized as a pivotal regulator in the development of myocardial hypertrophy. Studies have elucidated the formation of Nesprin-2/ERK complexes at sites of DNA damage in aortic smooth muscle cells, indicating a potential role of Nesprin proteins in modulating ERK signaling[36]. To investigate this phenomenon in the context of heart failure, we employed Western blot analysis to evaluate the expression levels of ERK in cardiac tissue samples from mice in each experimental group. Strikingly, our results revealed a notable increase in ERK expression in the model group compared to the control group, indicative of the activation of the ERK pathway in the cardiac tissue of the isoproterenol-induced heart failure mouse model(Fig12A-C)(*P*<0.01). Combined with the reduced expression of

Nesprin-1 in iprindel induced heart failure mice, we speculate that the reduced expression of Nesprin-1 may be associated with the activation of ERK pathway.

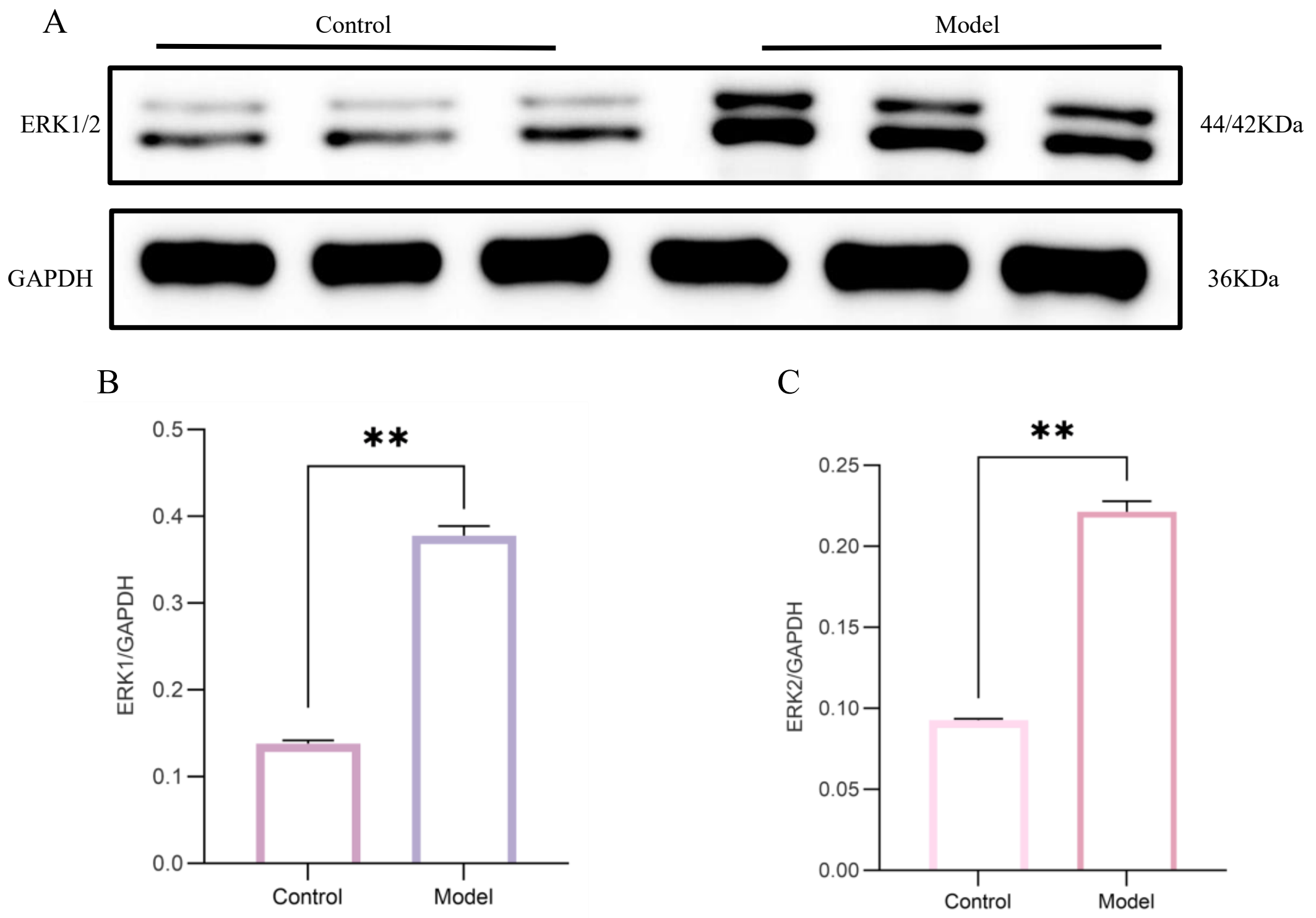

Figure 12: Downregulation of ERK1/2 expression in myocardium of mice with heart failure; A-C: Detection of ERK1/2 expression by protein immunoblotting; ERK: extracellular regulated protein kinase; n=3; compared with the control group,**P<0.01

Discussion

Heart failure stands as an enduring challenge within global healthcare systems, presenting a formidable burden characterized by its pervasive prevalence, grim prognostic outlook, and profound detriment to patients' quality of life. Despite notable strides in HF management witnessed over recent decades, including the advent of neurohormonal blockade, device-based interventions, and cardiac transplantation, the persistently high rates of mortality and morbidity underscore the insufficiency of current therapeutic strategies[37-39]. Consequently, there exists an imperative mandate for sustained investigative efforts directed towards unraveling the intricate molecular mechanisms governing HF pathogenesis.

In this study, We induced myocardial hypertrophy in mice by intraperitoneal injection of ISO. Our findings indicate that the administration of ISO successfully induced myocardial hypertrophy, characterized by significant changes in heart weight, body weight, and WGA pathological staining results compared to the control group

mice, consistent with previous studies [40, 41]. Long-term stimulation by pathological factors can lead to persistent changes in cardiac structure, resulting in HF [42]. HE staining revealed that Nesprin-1 deficiency exacerbated myocardial structural disorder, vascular proliferation, and myocardial hemorrhage. Additionally, cardiac function tests confirmed the onset of HF in the mice, with significant changes in LVEDd, LVESd, EF, and FS. When myocardial cell volume and pressure overload occur, ventricular muscle cells release BNP, which exerts sodium-losing, diuretic, vasodilatory, and antihypertensive effects [43]. BNP is a crucial biomarker for the clinical diagnosis of HF . Western blotting detected a significant increase in BNP in the myocardial tissue of mice induced by isoproterenol, providing a more reliable basis for confirming the onset of HF in mice.

Nesprin are giant scaffolding proteins located at the NE that play essential roles in maintaining nuclear structure and regulating various cellular processes, including nuclear positioning, cytoskeletal organization, and gene expression[27, 44-48]. While previous research has primarily focused on the role of Nesprin in skeletal and muscular diseases, their involvement in cardiac pathology, particularly HF, remains poorly understood.In this study, we used the ISO-induced mouse heart failure model to initially assess the changes in Nesprin-1 expression and its potential links with nuclear structure, signaling pathways, and gene expression regulation. The ISO-induced HF mouse model established and validated in this study provides new insights into the pathophysiology of heart failure and the role of Nesprin-1 in cardiac function.

Our research has revealed that, compared to the control group, the expression of Nesprin-1 in the myocardium of ISO-induced HF mice is significantly reduced. This finding suggests that changes in Nesprin-1 expression may be linked to pathological remodeling processes associated with HF, such as myocardial hypertrophy and dysfunction. The downregulation of Nesprin-1 may be related to abnormalities in the morphology, nuclear localization, and structural integrity of myocardial cells, which have been preliminarily observed through histopathological examination. However, the specific molecular mechanisms still require further investigation. Additionally, the

dysregulation of Nesprin-1 expression may affect nuclear stability by disrupting the integrity of the nuclear membrane, potentially leading to cellular dysfunction. These findings suggest that, as a key component of the nuclear scaffold and cytoskeletal connection complex, changes in Nesprin-1 expression may be associated with nuclear instability and abnormal cell signaling.

The exact mechanisms underlying Nesprin-1-mediated pathogenesis in HF remain incompletely understood, but our study has shed light on several potential avenues for further investigation. our findings elucidate the multifaceted role of Nesprin-1 in HF pathogenesis, encompassing oxidative stress, electrical conduction abnormalities, and epigenetic dysregulation. First, Nesprin-1 may affect cardiac morphology through interactions with cytoskeletal elements and extracellular matrix proteins, thereby regulating the signaling pathways involved in fibroblast activation. However, the causal relationship between Nesprin-1 and these processes has not been directly demonstrated. For example, how Nesprin-1 affects fibroblast activation by influencing cardiac morphology through interactions with cytoskeletal components and extracellular matrix proteins.

Additionally, Nesprin appears to modulate oxidative stress and DNA damage responses in the heart. Reduced Nesprin expression is associated with heightened oxidative stress, as evidenced by alterations in oxidative stress markers such as malondialdehyde levels and decreased superoxide dismutase content. Increased expression of DNA damage repair proteins, such as γH2AX and 53BP1, further suggests Nesprin's role in preserving genomic integrity and protecting against oxidative stress-induced cardiac injury. Furthermore, the observed reduction in connexin 43 expression in Nesprin-deficient hearts highlights Nesprin's involvement in regulating cardiac conduction and electromechanical coupling. Diminished Cx43 expression may contribute to conduction abnormalities and arrhythmias commonly observed in HF, emphasizing Nesprin's role in regulating both structural and functional aspects of cardiac tissue. Lastly, alterations in histone methylation patterns, specifically changes in H3K9me3 and H3K27me3 levels in Nesprin-deficient hearts, suggest dysregulated epigenetic regulation in HF pathogenesis. These findings

underscore the intricate interplay between Nesprin and epigenetic regulatory mechanisms in maintaining cardiac homeostasis.

Activation of the ERK pathway has been implicated in various hypertrophic stimuli and is known to regulate gene expression, protein synthesis, and cell survival in cardiomyocytes[49-51]. In addition to the identified alterations in Nesprin expression and its implications in cardiac remodeling, our study elucidates the involvement of the ERK signaling pathwaymay be involved in mediating Nesprin-1-induced cardiac hypertrophy and remodeling in response to isoproterenol stimulation. Activation of the ERK pathway has been implicated in various cellular processes, including cell proliferation, differentiation, and survival[52-54]. Here, we demonstrate a significant increase in ERK expression in the cardiac tissue of mice subjected to isoproterenol-induced heart failure, suggesting the activation of the ERK pathway in response to cardiac stress. The observed upregulation of ERK signaling in Nesprin-deficient hearts highlights a potential interaction between Nesprin and the ERK pathway in regulating cardiac function. Previous studies have indicated the formation of Nesprin-2/ERK complexes at sites of DNA damage, implicating Nesprin proteins in the modulation of ERK signaling in other cellular contexts[36]. Our findings extend this understanding to the cardiac setting, where Nesprin deficiency may contribute to ERK pathway activation, thereby exacerbating myocardial injury and dysfunction. The activation of the ERK pathway in response to cardiac stressors, such as isoproterenol administration, suggests a role of ERK signaling in mediating pathological remodeling and maladaptive responses in the heart. Future studies aimed at elucidating the precise mechanisms underlying the crosstalk between Nesprin and the ERK pathway may provide further insights into the molecular mechanisms of heart failure pathogenesis.

The identification of Nesprin-1 as a potential regulator of HF pathogenesis opens up new avenues for therapeutic intervention. Targeting Nesprin-1 and its associated signaling pathways may offer novel strategies for preventing or attenuating adverse cardiac remodeling and dysfunction in HF[55-58]. Small molecule inhibitors, gene therapy approaches, or gene editing techniques could be explored to modulate

Nesprin-1 expression or activity selectively in the heart. Additionally, our study underscores the importance of further research into the molecular mechanisms governing nuclear envelope dynamics and their contribution to cardiovascular disease. In conclusion, our study provides compelling evidence implicating Nesprin-1 in the pathogenesis of HF induced by isoproterenol. By elucidating the role of Nesprin-1 in cardiac remodeling, electrical conduction, we have shed light on novel molecular mechanisms underlying HF pathophysiology. Further investigation into the functional significance of Nesprin-1 and its interactions with key signaling pathways may yield valuable insights into disease progression and facilitate the development of targeted therapies for HF. Ultimately, our findings contribute to a deeper understanding of the complex molecular networks driving HF and offer hope for improved outcomes for patients afflicted with this devastating condition. This comprehensive discussion highlights the significance of our findings and provides a roadmap for future research endeavors aimed at unraveling the intricate molecular mechanisms of HF pathogenesis and identifying innovative therapeutic strategies to combat this disease.

However, this study still has some limitations. Firstly, the lack of specific inhibitors for Nesprin-1, along with the high mortality and lengthy construction period of Nesprin gene knockout mice, prevented us from directly establishing a causal relationship between Nesprin-1, oxidative stress, reduced Cx43 expression, increased DNA damage, and chromatin recombination disorders. Secondly, while we observed that decreased Nesprin-1 expression is associated with various pathological features, such as myocardial structural disorder, changes in oxidative stress markers, and increased expression of DNA damage repair proteins, these findings are only correlational. The specific molecular mechanisms require further functional experiments to confirm. Additionally, this study primarily used ISO-induced HF mouse models. Future studies should validate the role of Nesprin-1 in other HF models, such as those induced by stress overload or myocardial infarction, to enhance the generalizability of the research results. Lastly, regarding the interaction between Nesprin-1 and the ERK signaling pathway and epigenetic regulation (such as histone methylation), this study only provides preliminary clues. The specific mechanisms

need to be elucidated through more in-depth molecular and cellular experiments. In future research, we plan to construct a Nesprin-1-specific knockout model, combined with multi-omics analysis and functional validation, to further uncover its exact role in HF.

Declarations

Ethics approval and consent to participate

Not applicable.

Consent for publication

The manuscript was obtained from all authors for consent for submission and publication

Competing interests

The authors declare no competing interests

Funding

This research was funded by the Hubei Provincial Natural Science Foundation Project (Joint Fund Project, Project No.: 2024AFD143).The funders had no role in study design, data collection and analysis, decision to publish, or preparation of the manuscript.

Authors' contributions

Qin Qin and Dong Cengyuan completed the experiment and wrote the manuscript; Zhou Ziyi participated in some of the experiments and manuscript writing; Liu Yangyuanzhi was involved in the article revision; Professor Cao Chunyu provided guidance on the experimental design; Doctor Teng Lin guided the entire experimental process and manuscript writing.

Availability of Data and Material

All relevant data are within the paper and its Supporting Information files.

Acknowledgements

I would like to thank CJH for their technical assistance in this experiment. And I am very grateful for the funding provided by the Provincial Natural Science Foundation for this experiment.

Reference

[1] Tanai E, Frantz S. Pathophysiology of Heart Failure [J]. Compr Physiol, 2015, 6(1): 187-214.
[2] McDonagh T A, Metra M, Adamo M, et al. 2021 ESC Guidelines for the diagnosis and treatment of acute and chronic heart failure: Developed by the Task Force for the diagnosis and treatment of acute and chronic heart failure of the European Society of Cardiology (ESC) With the special contribution of the Heart Failure Association (HFA) of the ESC [J]. Rev Esp Cardiol (Engl Ed), 2022, 75(6): 523.
[3] Bozkurt B, Coats A J S, Tsutsui H, et al. Universal Definition and Classification of Heart Failure [J]. Journal of Cardiac Failure, 2021, 27(4): 387-413.
[4] Kemp C D, Conte J V. The pathophysiology of heart failure [J]. Cardiovasc Pathol, 2012, 21(5): 365-71.
[5] Ghionzoli N, Gentile F, Del Franco A M, et al. Current and emerging drug targets in heart failure treatment [J]. Heart Failure Reviews, 2021, 27(4): 1119-36.
[6] Savarese G, Becher P M, Lund L H, et al. Global burden of heart failure: a comprehensive and updated review of epidemiology [J]. Cardiovasc Res, 2023, 118(17): 3272-87.
[7] Shahim B, Kapelios C J, Savarese G, et al. Global Public Health Burden of Heart Failure: An Updated Review [J]. Card Fail Rev, 2023, 9: e11.
[8] Borovac J A, D'Amario D, Bozic J, et al. Sympathetic nervous system activation and heart failure: Current state of evidence and the pathophysiology in the light of novel biomarkers [J]. World J Cardiol, 2020, 12(8): 373-408.
[9] Bencivenga L, Palaia M E, Sepe I, et al. Why Do We Not Assess Sympathetic Nervous System Activity in Heart Failure Management: Might GRK2 Serve as a New Biomarker? [J]. Cells, 2021, 10(2).
[10] Wang J, Dai M, Cao Q, et al. Carotid baroreceptor stimulation suppresses ventricular fibrillation in canines with chronic heart failure [J]. Basic Research in Cardiology, 2019, 114(6).
[11] Huang Y, Liu Y-W, Pan H-Z, et al. Transthoracic Pulmonary Artery Denervation for Pulmonary Arterial Hypertension [J]. Arteriosclerosis, Thrombosis, and Vascular Biology, 2019, 39(4): 704-18.
[12] Triposkiadis F, Briasoulis A, Kitai T, et al. The sympathetic nervous system in heart failure revisited [J]. Heart Fail Rev, 2024, 29(2): 355-65.
[13] Grassi G, Mancia G, Esler M. Central and peripheral sympathetic activation in heart failure [J]. Cardiovasc Res, 2022, 118(8): 1857-71.
[14] Gronda E, Dusi V, D'Elia E, et al. Sympathetic activation in heart failure() [J]. Eur Heart J Suppl, 2022, 24(Suppl E): E4-E11.
[15] Munoz-Durango N, Fuentes C A, Castillo A E, et al. Role of the Renin-Angiotensin-Aldosterone System beyond Blood Pressure Regulation: Molecular and Cellular Mechanisms Involved in End-Organ Damage during Arterial Hypertension [J]. Int J Mol Sci, 2016, 17(7).
[16] Pugliese N R, Masi S, Taddei S. The renin-angiotensin-aldosterone system: a crossroad from arterial hypertension to heart failure [J]. Heart Fail Rev, 2020, 25(1): 31-42.
[17] Ferreira N S, Tostes R C, Paradis P, et al. Aldosterone, Inflammation, Immune System, and Hypertension [J]. Am J Hypertens, 2021, 34(1): 15-27.
[18] Weir M R, Dzau V J. The renin-angiotensin-aldosterone system: a specific target for hypertension management [J]. Am J Hypertens, 1999, 12(12 Pt 3): 205S-13S.
[19] Chen Y, He Q, Mo D-C, et al. The angiotensin receptor and neprilysin inhibitor, LCZ696, in heart failure: A meta-analysis of randomized controlled trials [J]. Medicine, 2022, 101(41).

[20] Heidenreich PA B B, Aguilar D,et al. Correction to: 2022 AHA/ACC/HFSA Guideline for the Management of Heart Failure: A Report of the American College of Cardiology/American Heart Association Joint Committee on Clinical Practice Guidelines [J]. Circulation, 2023, 147(14): e263-e421.

[21] Lachowska K, Gruchala M, Narkiewicz K, et al. Sympathetic Activation in Chronic Heart Failure: Potential Benefits of Interventional Therapies [J]. Curr Hypertens Rep, 2016, 18(7): 51.

[22] von Lueder T G, Kotecha D, Atar D, et al. Neurohormonal Blockade in Heart Failure [J]. Card Fail Rev, 2017, 3(1): 19-24.

[23] Ghionzoli N, Gentile F, Del Franco A M, et al. Current and emerging drug targets in heart failure treatment [J]. Heart Fail Rev, 2022, 27(4): 1119-36.

[24] Crisp M, Liu Q, Roux K, et al. Coupling of the nucleus and cytoplasm: Role of the LINC complex [J]. The Journal of Cell Biology, 2006, 172(1): 41-53.

[25] Meinke P, Nguyen T D, Wehnert M S. The LINC complex and human disease [J]. Biochem Soc Trans, 2011, 39(6): 1693-7.

[26] De Silva S, Catibog N, Shanahan C, et al. Investigating novel roles for nesprin-1 and the LINC complex in Dilated Cardiomyopathy [J]. Cardiovascular Research, 2022, 118(Supplement_1).

[27] Zhou C, Li C, Zhou B, et al. Novel nesprin-1 mutations associated with dilated cardiomyopathy cause nuclear envelope disruption and defects in myogenesis [J]. Hum Mol Genet, 2017, 26(12): 2258-76.

[28] Shanahan CM W P, Metcalfe JC. Isolation of gene markers of differentiated and proliferating vascular smooth muscle cells [J]. Circ Res, 1993, 73(1): 193-204.

[29] Chen Z, Ren Z, Mei W, et al. A novel SYNE1 gene mutation in a Chinese family of Emery-Dreifuss muscular dystrophy-like [J]. BMC Medical Genetics, 2017, 18(1).

[30] Stroud M J, Banerjee I, Veevers J, et al. Linker of Nucleoskeleton and Cytoskeleton Complex Proteins in Cardiac Structure, Function, and Disease [J]. Circulation Research, 2014, 114(3): 538-48.

[31] Lindenboim L, Grozki D, Amsalem-Zafran A R, et al. Apoptotic stress induces Bax-dependent, caspase-independent redistribution of LINC complex nesprins [J]. Cell Death Discov, 2020, 6(1): 90.

[32] Boengler K, Rohrbach S, Weissmann N, et al. Importance of Cx43 for Right Ventricular Function [J]. Int J Mol Sci, 2021, 22(3).

[33] Sur-Erdem I, Hussain M S, Asif M, et al. Nesprin-1 impact on tumorigenic cell phenotypes [J]. Mol Biol Rep, 2020, 47(2): 921-34.

[34] Yao L, He F, Zhao Q, et al. Spatial Multiplexed Protein Profiling of Cardiac Ischemia-Reperfusion Injury [J]. Circ Res, 2023, 133(1): 86-103.

[35] Li J, Sha Z, Zhu X, et al. Targeting miR-30d reverses pathological cardiac hypertrophy [J]. EBioMedicine, 2022, 81: 104108.

[36] Warren D T, Tajsic T, Porter L J, et al. Nesprin-2-dependent ERK1/2 compartmentalisation regulates the DNA damage response in vascular smooth muscle cell ageing [J]. Cell Death Differ, 2015, 22(9): 1540-50.

[37] Collaborators G B D R F. Global burden and strength of evidence for 88 risk factors in 204 countries and 811 subnational locations, 1990-2021: a systematic analysis for the Global Burden of Disease Study 2021 [J]. Lancet, 2024, 403(10440): 2162-203.

[38] Savarese G, Lund L H. Global Public Health Burden of Heart Failure [J]. Card Fail Rev, 2017, 3(1): 7-11.

[39] Sapna F, Raveena F, Chandio M, et al. Advancements in Heart Failure Management: A Comprehensive Narrative Review of Emerging Therapies [J]. Cureus, 2023, 15(10): e46486.

[40] Aimo A, Gaggin H K, Barison A, et al. Imaging, Biomarker, and Clinical Predictors of Cardiac Remodeling in Heart Failure With Reduced Ejection Fraction [J]. JACC Heart Fail, 2019, 7(9): 782-94.

[41] Goetze J P, Bruneau B G, Ramos H R, et al. Cardiac natriuretic peptides [J]. Nat Rev Cardiol, 2020, 17(11): 698-717.

[42] Salah K, Stienen S, Pinto Y M, et al. Prognosis and NT-proBNP in heart failure patients with preserved versus reduced ejection fraction [J]. Heart, 2019, 105(15): 1182-9.

[43] Apel E D, Lewis R M, Grady R M, et al. Syne-1, a dystrophin- and Klarsicht-related protein associated with synaptic nuclei at the neuromuscular junction [J]. J Biol Chem, 2000, 275(41): 31986-95.

[44] De Silva S, Fan Z, Kang B, et al. Nesprin-1: novel regulator of striated muscle nuclear positioning and mechanotransduction [J]. Biochem Soc Trans, 2023, 51(3): 1331-45.

[45] Leong E L, Khaing N T, Cadot B, et al. Nesprin-1 LINC complexes recruit microtubule cytoskeleton proteins and drive pathology in Lmna-mutant striated muscle [J]. Hum Mol Genet, 2023, 32(2): 177-91.

[46] Zhang Q, Ragnauth C D, Skepper J N, et al. Nesprin-2 is a multi-isomeric protein that binds lamin and emerin at the nuclear envelope and forms a subcellular network in skeletal muscle [J]. J Cell Sci, 2005, 118(Pt 4): 673-87.

[47] Rajgor D, Shanahan C M. Nesprins: from the nuclear envelope and beyond [J]. Expert Rev Mol Med, 2013, 15: e5.

[48] Zi-Yi Z, Qin Q, Fei Z, et al. Nesprin proteins: bridging nuclear envelope dynamics to muscular dysfunction [J]. Cell Commun Signal, 2024, 22(1): 208.

[49] Gallo S, Vitacolonna A, Bonzano A, et al. ERK: A Key Player in the Pathophysiology of Cardiac Hypertrophy [J]. Int J Mol Sci, 2019, 20(9).

[50] Mutlak M, Kehat I. Extracellular signal-regulated kinases 1/2 as regulators of cardiac hypertrophy [J]. Front Pharmacol, 2015, 6: 149.

[51] Ramos-Kuri M, Meka S H, Salamanca-Buentello F, et al. Molecules linked to Ras signaling as therapeutic targets in cardiac pathologies [J]. Biol Res, 2021, 54(1): 23.

[52] Sugiura R, Satoh R, Takasaki T. ERK: A Double-Edged Sword in Cancer. ERK-Dependent Apoptosis as a Potential Therapeutic Strategy for Cancer [J]. Cells, 2021, 10(10).

[53] Deschênes-Simard X, Malleshaiah M, Ferbeyre G. Extracellular Signal-Regulated Kinases: One Pathway, Multiple Fates [J]. Cancers (Basel), 2023, 16(1).

[54] Wu J, Chen Y, Li R, et al. Synergistic anticancer effect by targeting CDK2 and EGFR-ERK signaling [J]. J Cell Biol, 2024, 223(1).

[55] Li Y L, Cheng X N, Lu T, et al. Syne2b/Nesprin-2 Is Required for Actin Organization and Epithelial Integrity During Epiboly Movement in Zebrafish [J]. Front Cell Dev Biol, 2021, 9: 671887.

[56] Davidson P M, Battistella A, Dejardin T, et al. Nesprin-2 accumulates at the front of the nucleus during confined cell migration [J]. EMBO Rep, 2020, 21(7): e49910.

[57] Chen S N, Sbaizero O, Taylor M R G, et al. Lamin A/C Cardiomyopathy: Implications for Treatment [J]. Curr Cardiol Rep, 2019, 21(12): 160.

[58] Nicolas H A, Hua K, Quigley H, et al. A CRISPR/Cas9 zebrafish lamin A/C mutant model of muscular laminopathy [J]. Dev Dyn, 2022, 251(4): 645-61.

Figure

Click here to access/download;Figure;panel B.tif

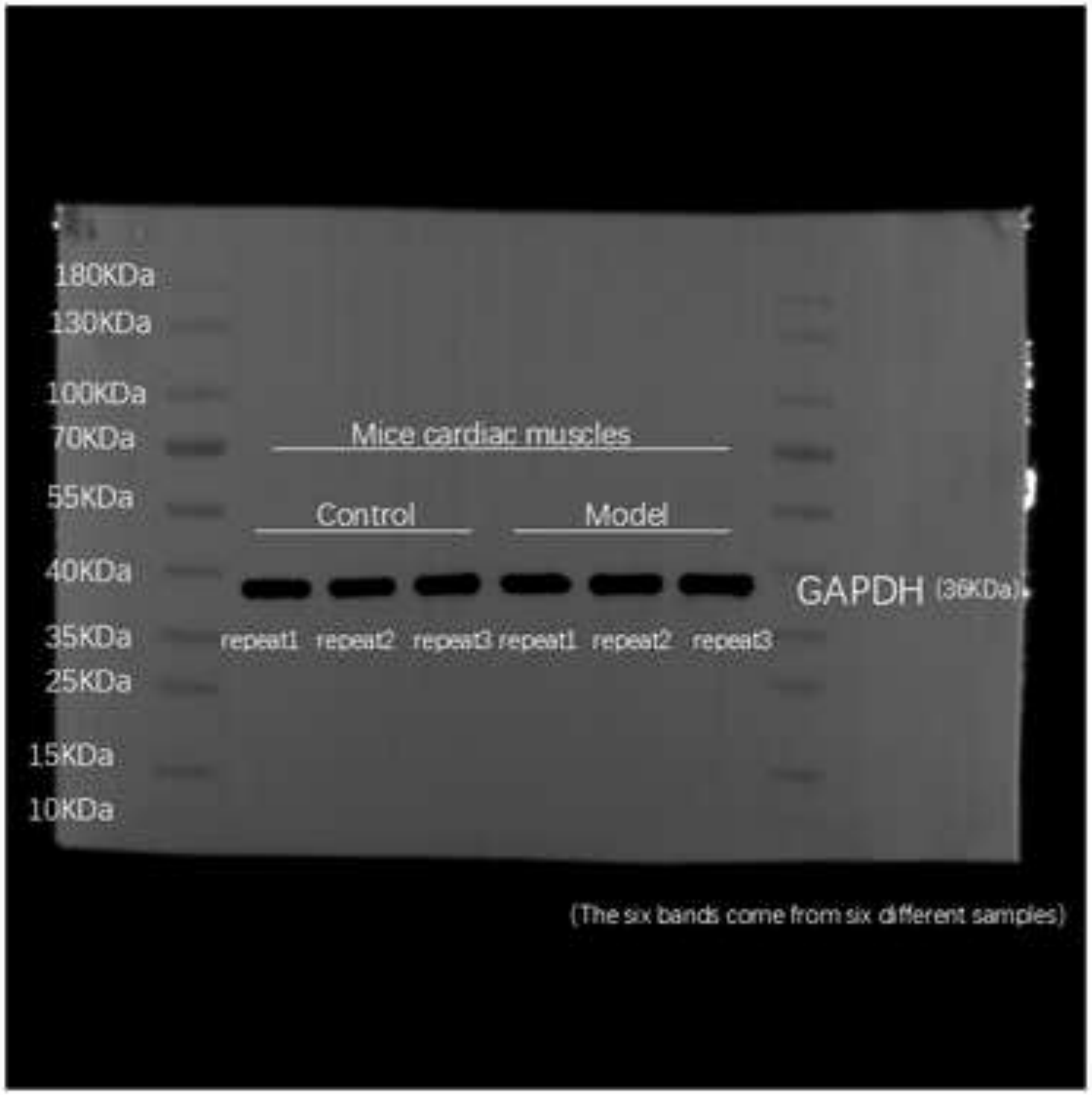

 Click here to access/download;Figure;panel C.tif

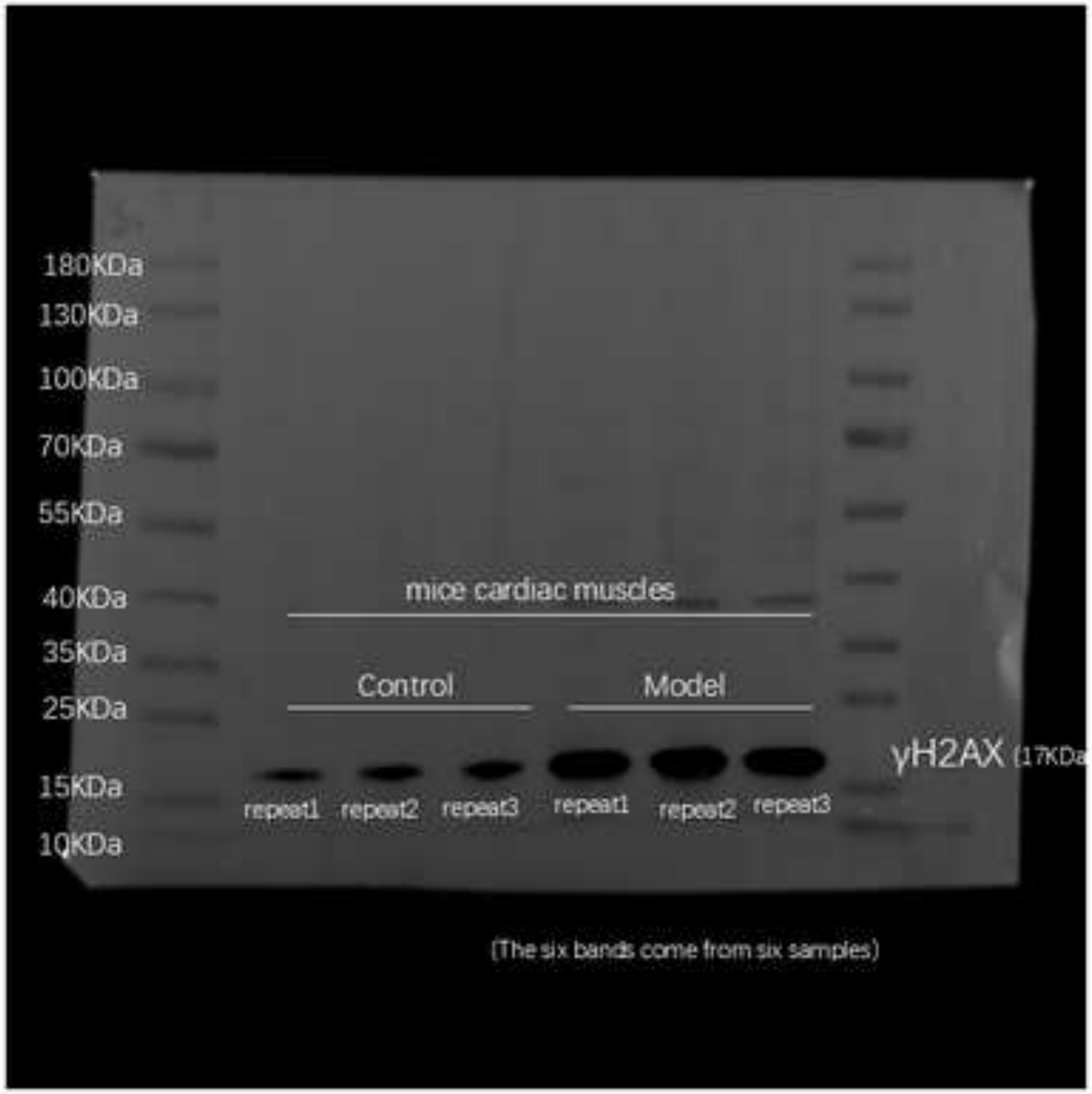

Figure

Click here to access/download;Figure;panel D.tif

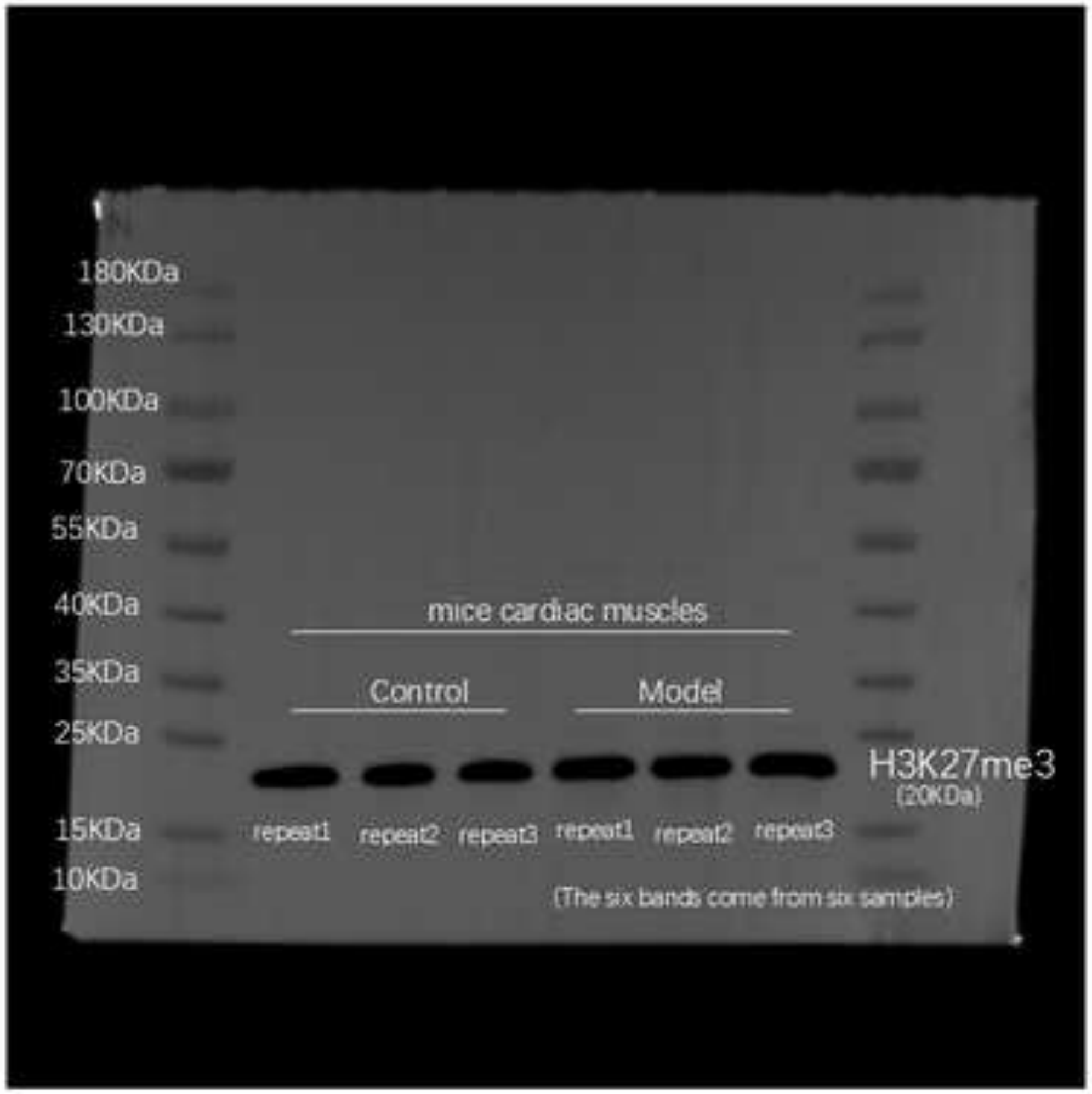

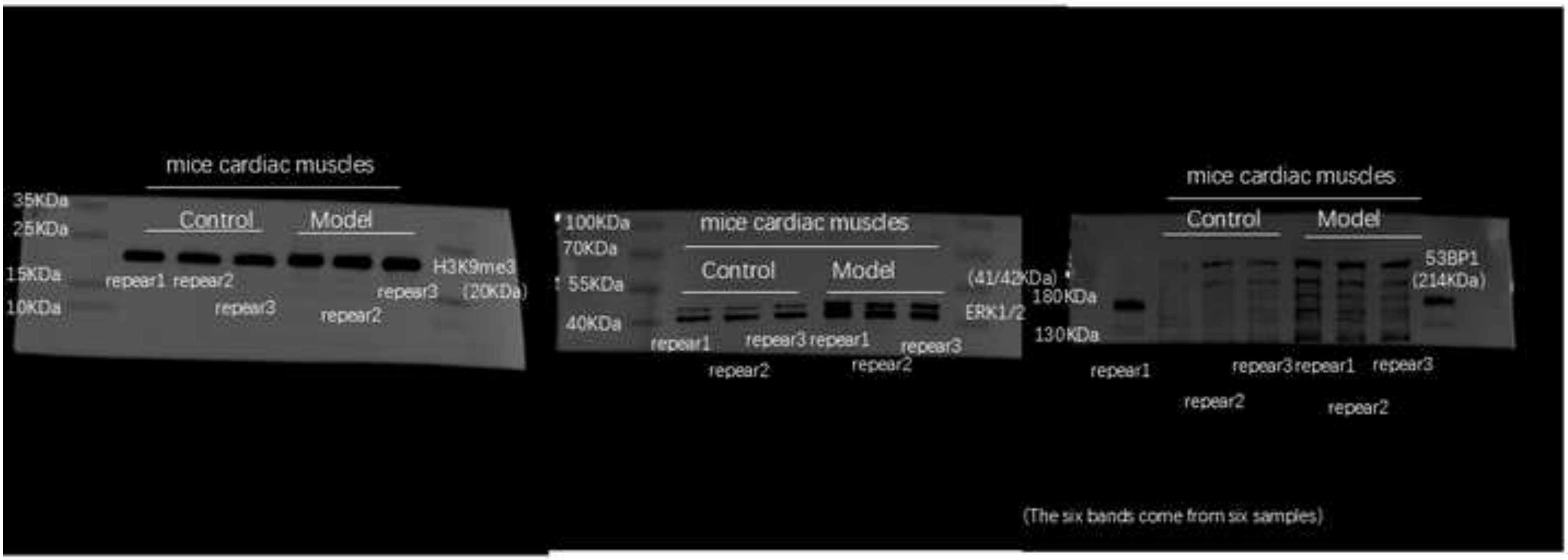
mice cardiac muscles
35KDa
25KDa
Control
Model
15KDa
10KDa
repear1
repear2
repear3
repear2
repear3
H3K9me3
(20KDa)
100KDa
70KDa
mice cardiac muscles
Control
Model
55KDa
40KDa
repear1
repear2
repear3
repear1
repear2
repear3
(41/42KDa)
ERK1/2
mice cardiac muscles
Control
Model
180KDa
130KDa
53BP1
(214KDa)
repear1
repear2
repear3
repear1
repear2
repear3
(The six bands come from six samples)

Figure

Click here to access/download;Figure;panel A.tif

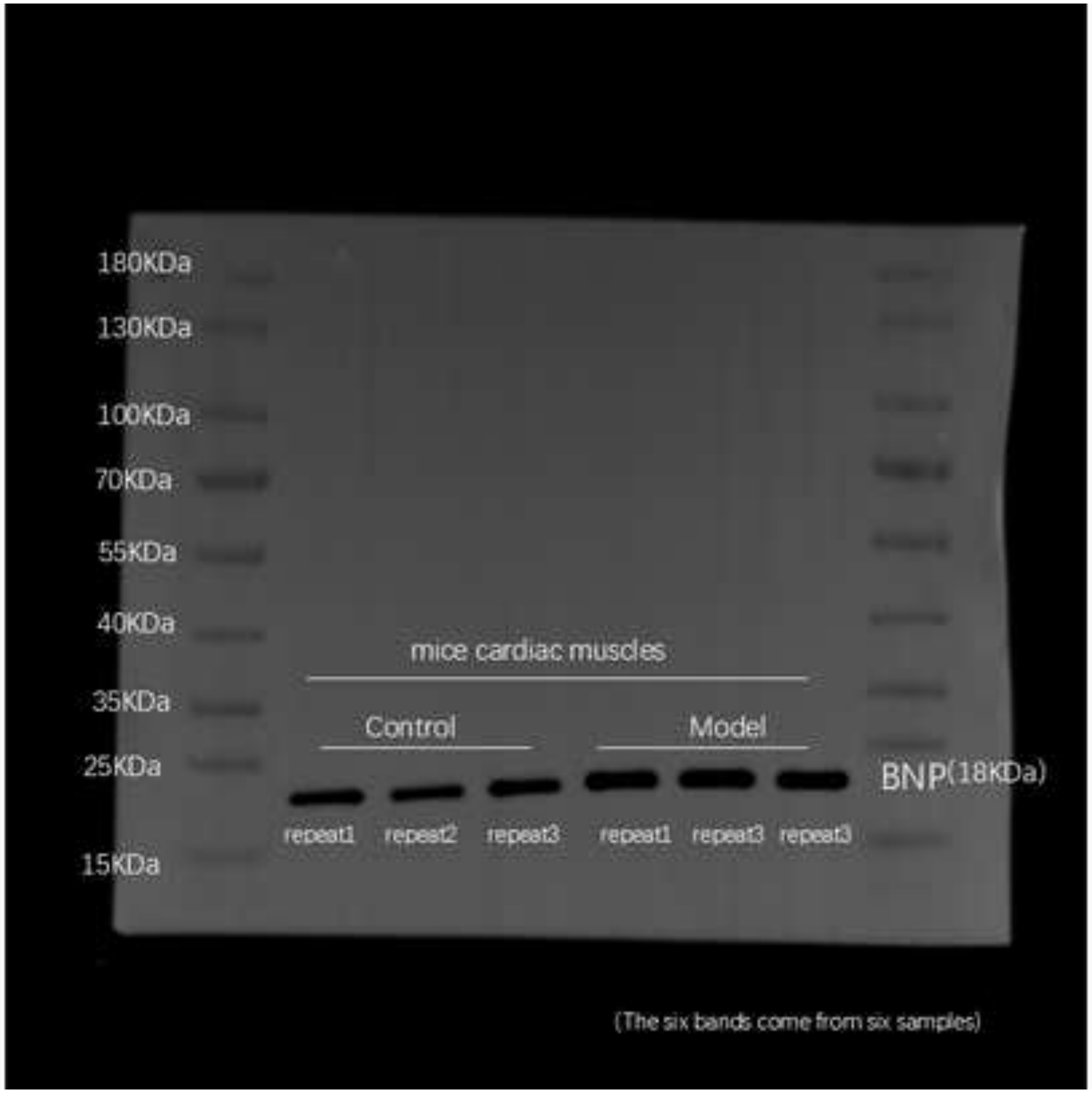

Click here to access/download
Supporting Information
Figure.docx

Click here to access/download
Supporting Information
body weight.xlsx

Click here to access/download
Supporting Information
Protocol for Animal Disposal.docx

Click here to access/download
Supporting Information
renamed_0c1f8.pdf

Click here to access/download
Supporting Information - Compressed/ZIP File Archive
Western Blot.zip

Corrected figure of Figure 2-B

Click here to access/download
Other
renamed_24468.jpg

Click here to access/download

Other

Animal Disposal Response.docx

Exploring the Role of Nesprin-1 in Isoproterenol-Induced Heart Failure Insights into Pathogenesis and Therapeutic Implications

Qin Qin[1,2#] , Dong Cengyuan[1,2#] , Zhou Zi-yi[1,2] ,Liu Yangyuanzhi[1,2], Cao Chunyu [2,4], TENG Lin[1,3*]

1. Department of Cardiology, Yichang Central People's Hospital/The First Clinical Medical College, Three Gorges University, Yichang 443003, Hubei, People's Republic of China

2. School of Basic Medicine, China Three Gorges University, Yichang 443000, Hubei, People's Republic of China

3. King's College London British Heart Foundation Centre of Research Excellence, School of Cardiovascular and Metabolic Medicine & Sciences, London SE5 9NU, U.K

4. College of Basic Medical Sciences, Hubei Key Laboratory of Tumor Microencironment and immunotherapy, China Three Gorges University, Yichang 443000, Hubei, People's Republic of China

# Qin Qin and Dong Cengyuan have equally to this work.

* Correspondence to: TENG Lin, MD, Department of Cardiology, Yichang Central People's, NO,183 Yiling Road, Yichang 443003, Hubei, People's Republic of China. E-mail address: tenglin@ctgu.edu.cn

Objective: In this study, we used an isoprenaline-induced heart failure model to investigate the role of Nesprin-1 in the development and progression of heart failure and to clarify the underlying molecular mechanisms.

Methods: A mouse model of heart failure was induced by daily intraperitoneal injection of isoproterenol (5 mg/kg/day) for 24 consecutive days. The body weight of mice was monitored daily. The diastolic left ventricular diameter (LVDd), systolic left ventricular diameter (LVDs), ejection fraction (EF) and short axis shortening rate (FS) of mice were evaluated by echocardiography of small animal hearts. The heart failure model was validated by gross cardiac observation, WGA staining, and Western blot analysis of B-type Natriuretic Peptide (BNP) expression. Hematoxylin-Eosin staining of heart tissue was performed to assess myocardial structure, cell morphology and pathological changes. Quantitative determination of malondialdehyde (MDA) and superoxide dismutase (SOD) expression was performed to evaluate oxidative stress levels. The expression of Nesprin-1 and connexin 43 (Cx43) was detected by immunofluorescence staining. Immunofluorescence staining and Western blot were used to detect the effects of Nesprin-1 expression changes on p53-binding protein 1 (53BP1), phosphorylated histone H2AX (γH2AX), H3 lysine 9 trimethylation (H3K9me3), H3 lysine 27 trimethylation (H3K27me3) expression.

In addition, we used Western blot to analyze the expression of the extracellular regulatory protein kinase (ERK) pathway.

Results: Compared with the control group, mice in the isoproterenol-induced heart failure model group exhibited significant increases in both body weight and heart weight ($P<0.01$), enlargement of cardiomyocyte cross-sectional area ($P<0.01$), markedly elevated BNP expression ($P<0.01$). The echocardiographic results showed that the LVDd and LVDs in diastolic phase were significantly increased, and EF and FS were significantly decreased in mice with heart failure($P<0.01$). Histopathological analysis demonstrated disordered myocardial structure, vascular proliferation, and myocardial hemorrhage in model group mice. The expression of Nesprin-1 was significantly downregulated in the model group ($P<0.01$), accompanied by abnormal nuclear envelope localization that resulted in distorted nuclear morphology of cardiomyocytes, including increased nuclear area and perimeter, elevated nuclear aspect ratio ($P<0.01$) , and decreased nuclear circularity($P<0.01$). Additionally, the model group showed significantly increased levels of the oxidative stress marker MDA coupled with decreased SOD activity ($P<0.05$), reduced Cx43 expression ($P<0.01$), upregulated expression of DNA damage markers (γH2AX and 53BP1)($P<0.01$), significantly elevated levels of H3K9me3 and H3K27me3 ($P<0.01$), and markedly enhanced activation of the ERK signaling pathway ($P< 0.01$).

Conclusion: This study revealed that downregulation of Nesprin-1 may serve as a crucial driver in the progression of heart failure. In the isoproterenol-induced murine heart failure model, reduced Nesprin-1 expression leads to multiple pathological alterations, including nuclear homeostasis imbalance, decreased Cx43 expression, elevated oxidative stress, accumulated DNA damage, and disordered epigenetic regulation. The underlying mechanism may involve activation of the ERK signaling pathway.

Keywords: Heart failure, Nesprin-1, Isoproterenol, Cardiac remodeling, Dysfunction, Epigenetic regulation, DNA damage

Introduction

Heart failure (HF), or cardiac failure, signifies the heart's diminished capacity to efficiently pump blood, resulting in venous congestion and inadequate arterial perfusion due to compromised systolic and/or diastolic functions[1-4]. This condition represents the terminal phase of heart disease and stands as a leading cause of cardiovascular-related mortality worldwide. HF affects approximately 1% to 2% of the global population, with a notably higher prevalence among the elderly[5-7]. Sympathetic nervous system (SNS) overactivity is a hallmark of HF, correlating with disease severity and prognosis. Under normal circumstances, the cardiac sympathetic nervous system enhances cardiac output, gene transcription, and metabolism by releasing neurotransmitters and engaging β-adrenergic receptors (β-AR)[8, 9]. However, excessive SNS activation detrimentally affects the cardiovascular system, potentially precipitating or exacerbating HF[10, 11]. This overdrive leads to heightened heart rate and contractility, inducing a sustained high-load state that depletes cardiac energy reserves, impeding cardiac function. Furthermore, it promotes adverse structural cardiac changes such as ventricular hypertrophy and atrial dilation, diminishing cardiac pump efficiency[12-14]. Increased arterial vasoconstriction further strains the heart by elevating vascular tension. Excessive sympathetic stimulation also incites inflammation and oxidative stress, damaging myocardial cells and hastening disease progression. Disruption of neuroendocrine system balance, including the renin-angiotensin-aldosterone system, can exacerbate fluid retention and increase blood volume, exacerbating cardiac workload[15-18]. Current HF management strategies primarily target the sympathetic nervous system with medications like β-blockers, angiotensin-converting enzyme inhibitors, angiotensin II receptor blockers, and aldosterone receptor antagonists. Despite these interventions, HF incidence, readmission rates, and mortality remain high, underscoring the need for novel therapeutic approaches[19-23].

Traditionally, HF research focused on intracellular factors, neglecting the nuclear envelope's role. The nuclear envelope (NE) is pivotal, spatially segregating nuclear genetic material and regulating macromolecular transport[24]. The Linker of

Nucleoskeleton and Cytoskeleton (LINC) complex, anchored to the NE, includes Nesprin proteins, which bridge the nuclear and cytoskeletal proteins, contributing to cellular structure and function. Nesprin mutations are implicated in various diseases, including Dilated Cardiomyopathy (DCM) and Emery-Dreifuss Muscular Dystrophy (EDMD), highlighting their importance in muscle physiology[25-27]. Nesprin family proteins, integral to the NE, include Nesprin-1 and Nesprin-2, which interact with actin, intermediate filaments, and the nuclear envelope, influencing cell signaling, morphology, and mechanical properties[28]. These proteins regulate gene expression, chromatin structure, and epigenetic modifications, impacting cell function and pathology. Mutations in Nesprin-1 and Nesprin-2 are associated with abnormal nuclear morphology, positioning, and chromatin organization, contributing to DCM and EDMD. Nesprin alterations also affect intercalated disc protein localization, leading to conduction defects in Nesprin-associated cardiomyopathies[27, 29, 30].

Given this background, we hypothesize that the dysregulation of Nesprin-1 expression disrupts the structural connection between the nucleus and the cytoskeleton, thereby impairing nuclear homeostasis, interfering with signal transduction and causing abnormal transcription regulation, which ultimately leads to myocardial remodeling and contractile dysfunction, and then heart failure. Our study aims to elucidate Nesprin-1's role and molecular mechanisms in HF, providing insights for diagnosis and treatment. Understanding Nesprin-1's function may reveal therapeutic targets, guiding future HF therapies.

Materials and methods

Drugs and Chemicals

MedChemExpress provided isoproterenol (HY-B0468) and isoflurane, while Biosharp supplied 4% paraformaldehyde (BP003), hematoxylin-eosin staining (BP019), and other staining-related reagents. Sinopharm Chemical Reagent offered basic reagents such as paraffin (69019361) and xylene (10023418). Antibodies were mainly sourced from Boster, including Cx43 antibody (A00599), BNP antibody (M01186-3), 53BP1 antibody (BM5099), γH2AX antibody (BM4148), H3K9me3 antibody (BM5079), H3K27me3 antibody (BM4341), and ERK antibody (BM4156).

Protein-related reagents were primarily obtained from Beyotime, including RIPA lysis buffer (P0013B) and BCA protein assay kit (P0010). Additionally, Amresco provided electrophoresis reagents such as TEMED (Amresc00761) and acrylamide (Exp2016109), while Jackson supplied HRP-labeled secondary antibodies, specifically HRP-conjugated goat anti-rabbit (BA1054), HRP-conjugated rabbit anti-goat (BA1060), HRP-conjugated goat anti-mouse (BA1051), and HRP-conjugated goat anti-rat (BA1058). Other essential reagents included WGA stock solution (BBL-0351) from Sigma-Aldrich, DAPI stock solution (C1002) from Solarbio, and cytoplasmic/nuclear protein extraction kit (KGP150) from Nanjing KeyGen Biotech.

Establishment of Isoproterenol-Induced Heart Failure Model in Mice

The male C57BL/6 mice weighing 20-22g were purchased from the Animal Experimental Center of Three Gorges University. All mice were housed in specific pathogen-free (SPF) animal facilities at Three Gorges University (Yichang, Hubei, China). The experimental animal quality certification number is 42010200009428. The experimental animal use permit number is 00305510. Twenty-four C57BL/6 mice were randomly divided into two groups: model group and control group. In the model group, mice were administered intraperitoneal injections of isoproterenol (ISO) at a dosage of 5mg/kg. In the control group, mice were administered intraperitoneal injections of an equivalent volume of medical-grade physiological saline. Two groups received intraperitoneal injections continuously for 24 days.

Cardiac Function Assessment

Mice were anesthetized with isoflurane, and after achieving stable anesthesia, the hair in the chest area was removed. The mice were then placed in a supine position and fixed on an ultrasound examination table. Ultrasound coupling agent was applied to the chest area. Using small animal ultrasound, M-mode images of the long axis and short axis of the mouse heart were obtained. Parameters including ejection fraction (EF), fractional shortening (FS), left ventricular end-diastolic diameter (LVEDd), and left ventricular end-systolic diameter (LVESd) were measured and calculated to assess cardiac function in mice. Statistical analysis was performed to analyze the cardiac

function of mice.

Measurement of Body and Heart Weight

Body weight of the mice was measured before each dose.At least 24 hours after the last dose the mice were anesthetized, and their chests were swiftly opened using surgical scissors and forceps to extract the hearts. The hearts were rinsed in pre-cooled physiological saline, and then scissors were used to trim the blood vessels and connective tissues surrounding the heart. The hearts were then rinsed again with fresh physiological saline and dried with filter paper. Gross images of the hearts were captured, and the total heart weight was measured. Some samples were frozen in dry ice and stored in-80℃ refrigerator, while others were fixed in 4% polyformaldehyde fixative for easy observation of histological morphology.

Pathological Section Staining

HE staining: After dehydration, embedding, and sectioning of tissue fixed with 4% polyformaldehyde, the paraffin sections are dewaxed to water. Stain with hematoxylin for 4 minutes, followed by 2-minute rinsing with tap water to remove excess stain. The sections are then differentiated with 0.8% hydrochloric acid alcohol for 2 seconds, rinsed again with tap water, immersed in eosin solution for 20 seconds, adjusted with 95% ethanol for 5 seconds, and dehydrated with anhydrous ethanol for 2 minutes. Finally, the sections are sealed with a transparent environmental agent and observed under a microscope.

WGA staining: The paraffin sections were sequentially dewaxed with eco-friendly degreasing agents (1), (2), and (3) for 10 minutes each, followed by 5-minute immersion in anhydrous ethanol, 95% ethanol, and 75% ethanol respectively. The sections underwent three 3-minute rinses with distilled water. Antigen retrieval was performed by high-pressure treatment of the sections in boiling citric acid (pH6.0) solution for 2 minutes. After cooling, the sections were immersed in distilled water for three 5-minute washes. A circular mark was made on the section using a histological pen, then it was immersed in TBST buffer. A 1:200 diluted WGA working solution (50 uL/section) was added and incubated under light protection for 30 minutes, followed by three 5-minute TBST washes. After removing TBST, a 1:500 diluted

DAPI working solution (50 uL/section) was applied to stain nuclei for 5 minutes. Following TBST rinsing, the sections were mounted with anti-fluorescence quenching medium and stored at 4℃ under light protection. Finally, microscopic examination and image analysis were conducted.

The biochemical kits detects the oxidative stress indicators

Sample Preparation: Myocardial tissue was mixed with PBS buffer at ratios of 1:10 (for MDA detection) and 1:9 (for SOD detection), homogenized, and centrifuged at 10,000 g for 15 min at 4 ℃. The supernatant was collected for subsequent analysis.

Reagent Preparation: MDA detection working solution (including probe working solution and standard) and SOD detection working solution (including enzyme reaction solution) were prepared according to the kit instructions.

MDA Measurement: The sample was mixed with the detection working solution, incubated at 95 ℃ for 40 min, cooled on ice for 5 min, and centrifuged at 10,000 g for 10 min. The supernatant was measured at 532 nm absorbance.

Calculation formula:

MDA(umol/gprot)=[(A_{sample}-A_{blank}) /(A_{standard}-A_{blank})]×C_{standard}×dilution factor / protein concentration

SOD Measurement: 20 uL of sample was added, incubated at 37 ℃ for 20 min, and measured at 450 nm absorbance.

Calculation formulas:

SOD inhibition rate (%) = [(ΔA_{control} - ΔA_{sample}) / ΔA_{control}] × 100%

SOD activity (U/mg prot) = Inhibition rate / (100% - Inhibition rate) × 12 / protein concentration

(Note: In the formulas, C_{standard} is the standard concentration, ΔA is the absorbance difference, and 12 is the conversion factor for reaction volume/sample volume.)

Immunofluorescence Staining Protocol for Mouse Cardiac Tissue

Initially, tissues fixed with 4% paraformaldehyde were processed through dehydration,

embedding, and sectioning, followed by dewaxing the paraffin sections to water. Antigen retrieval was then performed by incubating the sections in preheated 95° C sodium citrate buffer (pH 6.0) under high-pressure heating for 10 minutes. After natural cooling, the sections were washed three times with phosphate-buffered saline (PBS), 3 minutes each. Excess moisture was removed using absorbent paper, and the tissue areas were circled with an immunohistochemical pen before applying diluted normal goat serum for 30-minute blocking at room temperature. After blocking, excess liquid was removed (without washing), and diluted primary antibody was directly applied for overnight incubation at 4 ℃ in a humidified chamber. The following day, sections were thoroughly washed with PBS (⩾3 times, 3 minutes each), dried, and treated with diluted fluorescent secondary antibody for 1-hour incubation at 25 ℃ in a humidified chamber, followed by four additional PBS washes (3 minutes each). For nuclear staining, DAPI was applied under light-protected conditions for 5 minutes, followed by four PBS washes (5 minutes each) to remove excess dye. Finally, sections were dried and mounted with anti-fade mounting medium for observation and image acquisition under a fluorescence microscope.

Western Blot Experimental Procedure

Protein extraction was first performed by mincing tissue samples in a 2mL homogenizer, followed by adding 400 uL lysis buffer containing PMSF for ice-cold homogenization with repeated processing at 5-minute intervals until complete tissue disruption was achieved. After 30 minutes of lysis, the lysate was centrifuged at 12,000 rpm for 5 minutes at 4 ℃, and the supernatant was aliquoted and stored at -20 ℃. Protein concentration was determined using the BCA method: samples were diluted 1:19 with PBS, and a standard curve (0.2-1mg/mL BSA) was prepared and loaded onto a 96-well plate alongside samples (standards in duplicate, samples in triplicate). After adding BCA working solution, the plate was incubated at 37° C for 30 minutes protected from light, and absorbance at 568 nm was measured to calculate protein concentration. Protein samples were mixed with 5×loading buffer and denatured by boiling for 10 minutes before storage.

For electrophoresis, separating and stacking gels were prepared, and 40ug total protein was loaded per well. Initial electrophoresis was performed at 80V until samples entered the separating gel, then switched to 120V for approximately 1.5 hours until the bromophenol blue reached the gel bottom. Prior to transfer, PVDF membranes were cut and activated with methanol, and the transfer sandwich was assembled in the order of "negative plate-fiber pad-filter paper-gel-membrane-filter paper-fiber pad-positive plate" for constant current transfer. After transfer, membranes were blocked with 5% skim milk in TBST (2% BSA for phosphoproteins) at room temperature for 2 hours.

Following overnight primary antibody incubation at 4 ℃, membranes were washed 6 times with TBST, then incubated with HRP-conjugated secondary antibody at 37 ℃ for 2 hours, followed by 5 additional TBST washes. For ECL detection, enhanced solution and peroxidase solution were mixed in equal proportions and applied to membranes. After several minutes of reaction, X-ray films were exposed and processed through development and fixation to obtain results. Throughout the procedure, care was taken to maintain samples at low temperatures, ensure bubble-free transfer, and keep membranes moist during antibody incubations.

Statistical analysis

All quantitative data were expressed as mean ± standard deviation (X±SD). The comparison between the two groups was evaluated using the t-test. *P* value <0.05 indicated statistically significant differences. The data were analyzed using GraphPad Prism 10.0 software.

3.Result

1.Establishment and Validation of Isoproterenol-Induced Heart Failure Mouse Model

Echocardiographic evaluation revealed notable alterations in cardiac parameters across the two experimental groups(Fig1A-B). Mice in the model group displayed significant increases in left ventricular end-diastolic diameter (LVEDd) and left ventricular end-systolic diameter (LVESd) compared to the control groups(Fig1C-D)($P<0.01$). Conversely, ejection fraction (EF) and fractional

shortening (FS) were markedly reduced in the model group(Fig1E-F)(P<0.01). These findings underscore the effectiveness of the isoproterenol-induced myocardial hypertrophy model in inducing structural and functional changes in cardiac function.

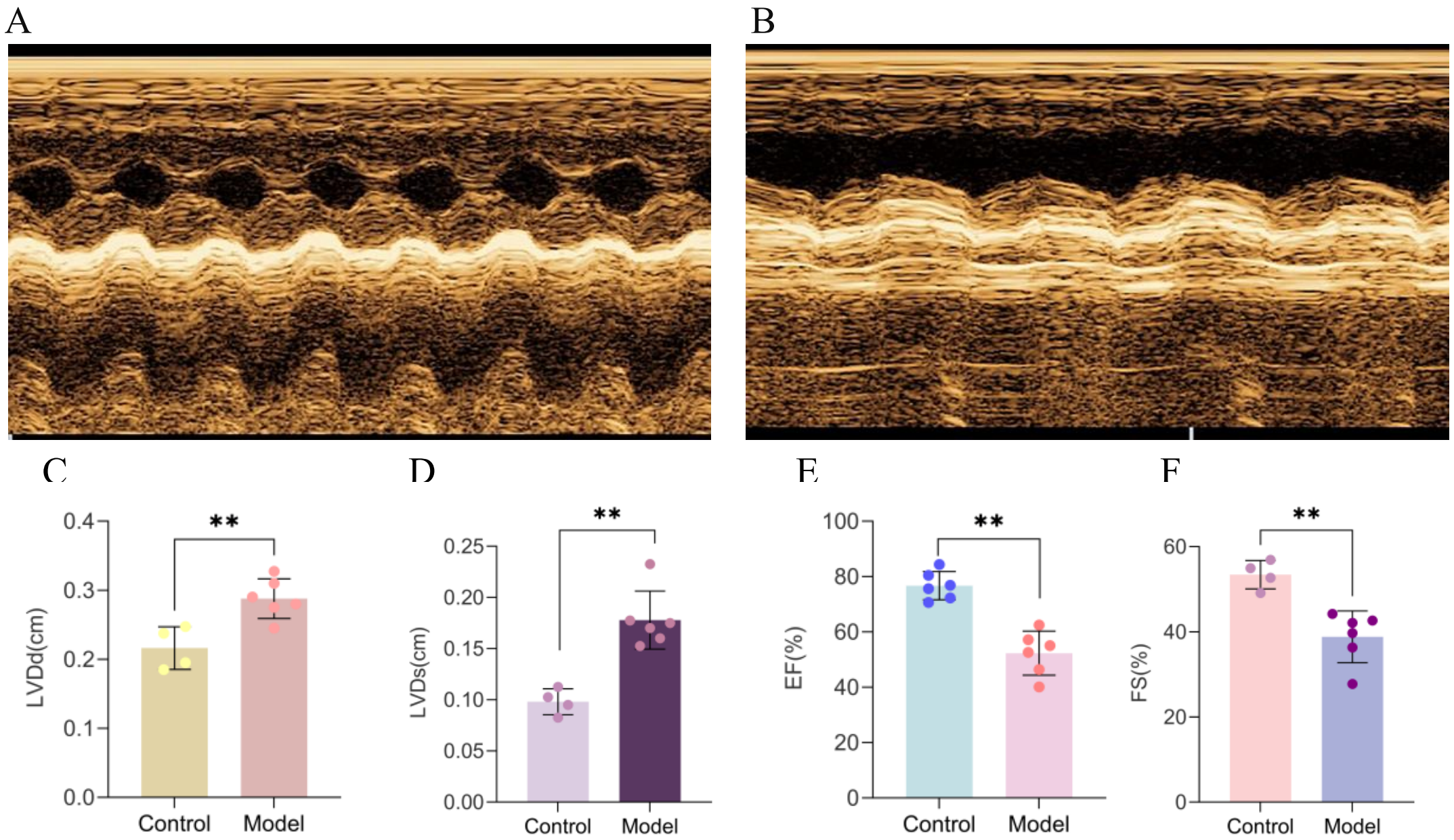

Figure 1: Isoproterenol-induced Decrease in Cardiac Function in Mice. A: Echocardiogram of the control group mice; B: Echocardiogram of the model group mice; C-F: Left ventricular diameter (LVDd), left ventricular diameter at systole (LVDs), ejection fraction (EF), and short-axis shortening rate (FS) of the left ventricle in mice; n≥4; Compared to the control group, ***P*<0.01

Assessment of cardiac weight provided additional insights into physiological changes among the experimental groups. Mice in the isoproterenol-induced heart failure model group exhibited a significant increase in body weight starting from day 13 of drug administration, compared to controls(Fig2A). Moreover, body weight and cardiac weight significantly increased in the model group after 24 days of isoproterenol administration, indicative of myocardial hypertrophy(Fig2B-C)(P<0.01). WGA staining demonstrated a significant increase in cardiomyocyte cross-sectional area in the model group compared to controls(Fig2D-E)(P<0.01).By examining BNP in both groups using Western Blot, we found that BNP was significantly elevated in the model group compared to the control group(Fig2F-G)(P<0.01). These findings highlight the capacity of isoproterenol to induce cardiac hypertrophy in mice.

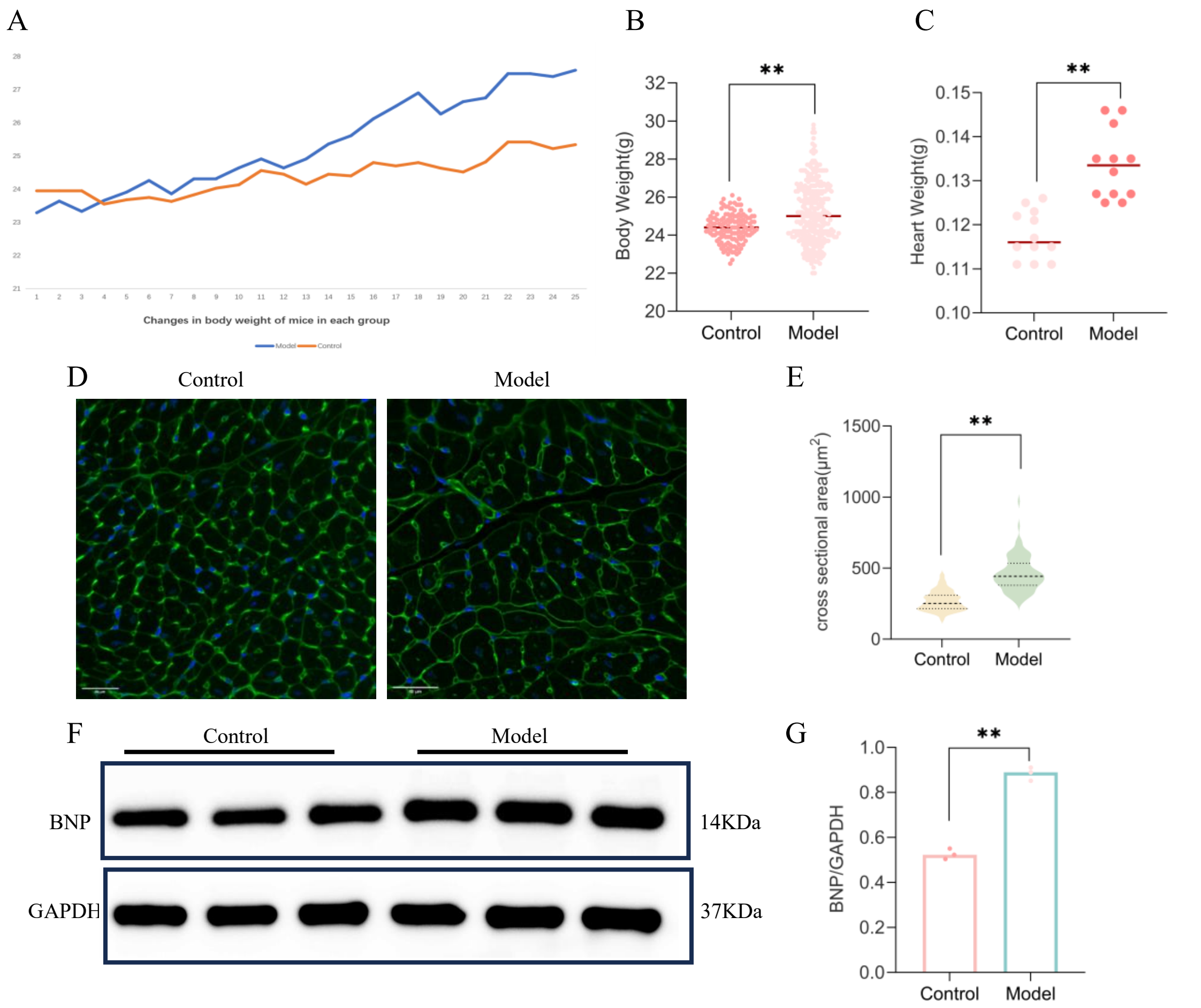

Figure2：Isoproterenol-induced heart failure in mice. A: Graph of changes in mouse body weight; B-C: Body and cardiac weights of the two groups of mice; n=12; D-E: Myocardial WGA staining of mice; n=5; F-G: BNP protein expression levels of the two groups of mice; n=3. Compared with the control group, ** P<0.01

Morphological and pathological staining of the heart in mice revealed significant differences in myocardial structure between the two groups (Figure 3A). Compared to the control group, the model group mice exhibited a significantly larger heart volume (Figure 3A). A comparison of cross-sectional images from the hearts of the two groups showed that the ventricular cavity in the model group mice was notably enlarged (Figure 3B). HE staining results indicated that the model group mice exhibited disordered myocardial structure, vascular proliferation, and myocardial hemorrhage (Figure 3C-D). These findings collectively highlight the pathological changes in myocardial hypertrophy induced by isoproterenol in mice.

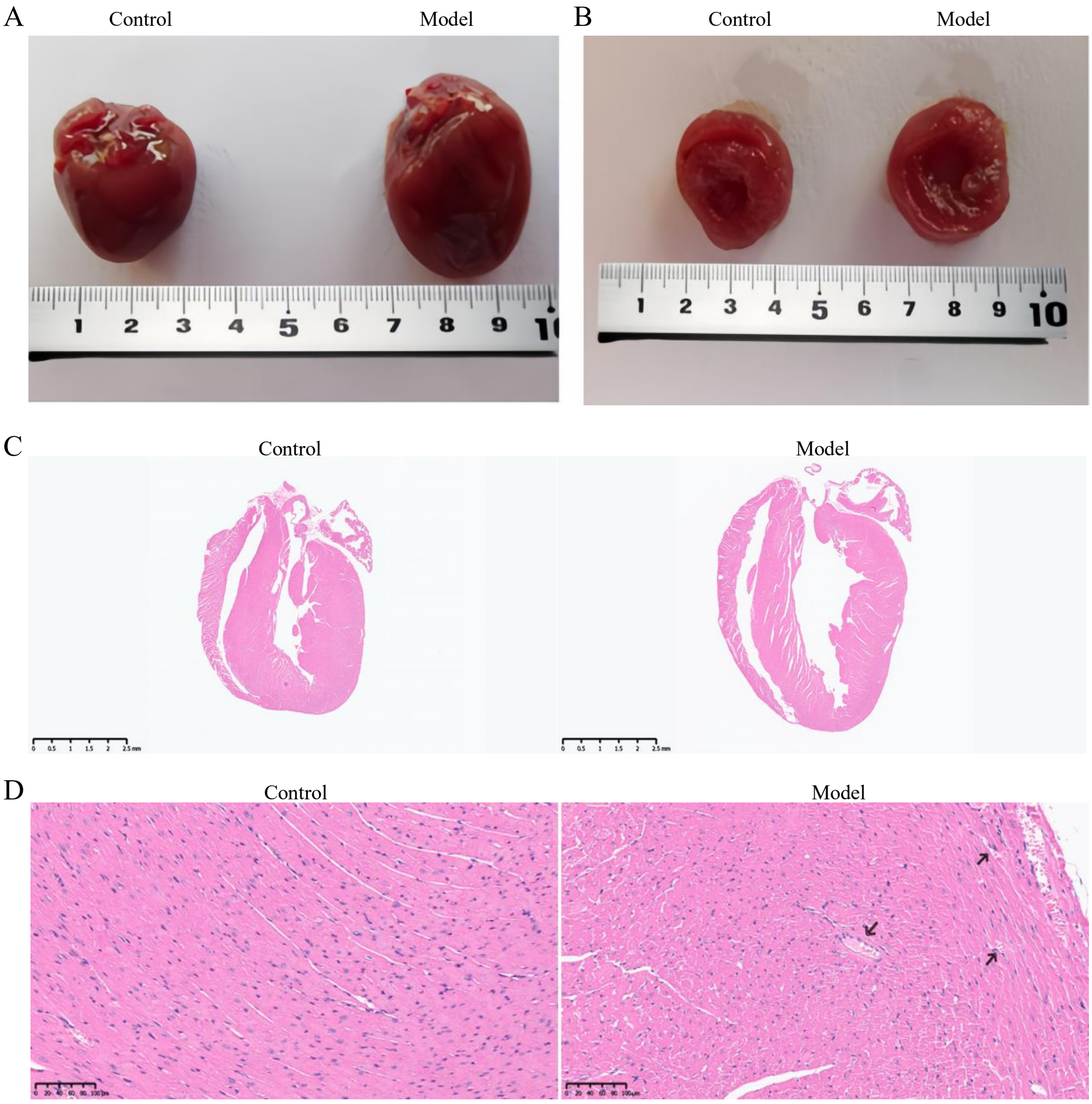

Figure 3: Gross view and pathological staining of mouse heart . A: Gross view of mouse heart; B: Coronary section of mouse heart; C-D: HE staining of mouse myocardial tissue; n=5

2.The expression of Nesprin-1 in the heart of mice with isoproterenol induced heart failure was significantly reduced

The immunofluorescence analysis revealed a significant decrease in Nesprin-1 expression in the hearts of mice induced with isoproterenol-induced heart failure(Fig4A)(P<0.01).By fluorescent staining of Nesprin-1 with specific antibodies, we observed that Nesprin-1 was mainly located at the nuclear membrane of cardiomyocytes (Fig4A). This localization is consistent with its function in maintaining nuclear membrane structure and nuclear-cell skeleton connection.

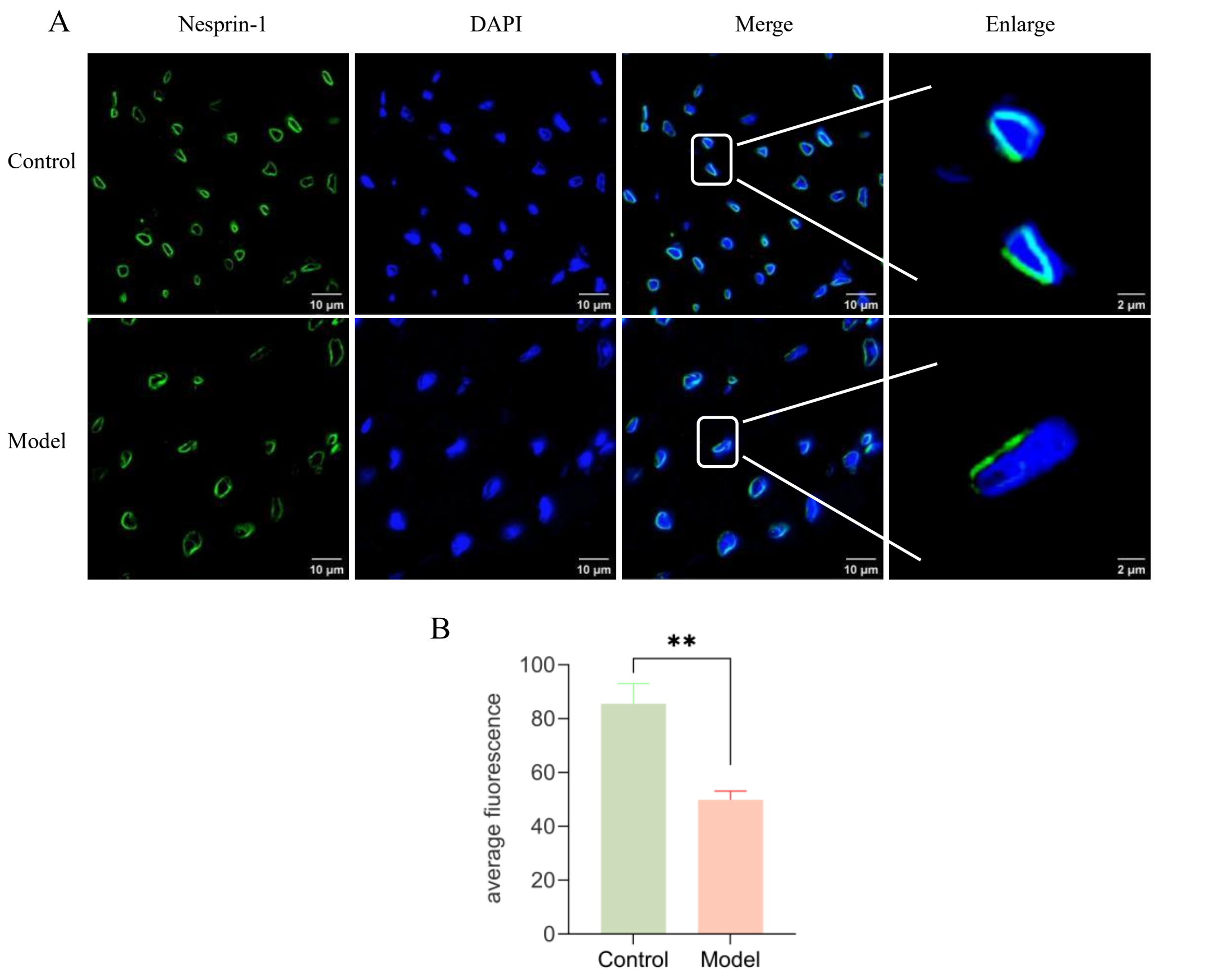

Figure 4: Expression changes of Nesprin-1 in myocardial tissue. A-B: Immunofluorescence staining of Nesprin in mouse myocardial tissue; n=5; Compared with the control group, ***P*<0.01

3.Dysregulation of Nesprin-1 expression in cardiac tissue of isoproterenol induced heart failure mouse model affects nuclear homeostasis in heart failure

In our experimental study, we employed immunofluorescence to investigate the effects of Nesprin-1 expression changes on nuclear homeostasis in cardiomyocytes. By randomly selecting 40 fluorescently stained pathological sections from five model groups, we obtained 40 tissue micrographs. Ten nuclei were randomly selected from each image and analyzed using ImageJ software for morphological parameters including nuclear area, perimeter, and aspect ratio. Results demonstrated that compared with the control group, the nuclear morphology of cardiomyocytes in the model group exhibited significant abnormalities, characterized by marked increases in nuclear area, perimeter, and aspect ratio, along with a notable reduction in nuclear roundness (Figure 5A)(*P*<0.01). Furthermore, immunofluorescence staining revealed partial or complete absence of Nesprin-1 on the nuclear membrane in some cardiomyocytes of the model group (Figure 5B)(*P*<0.01). These findings indicate that

Nesprin-1 expression dysregulation affects nuclear homeostasis in ISO-induced HF mouse models, though the causal relationship between these two factors requires further investigation.

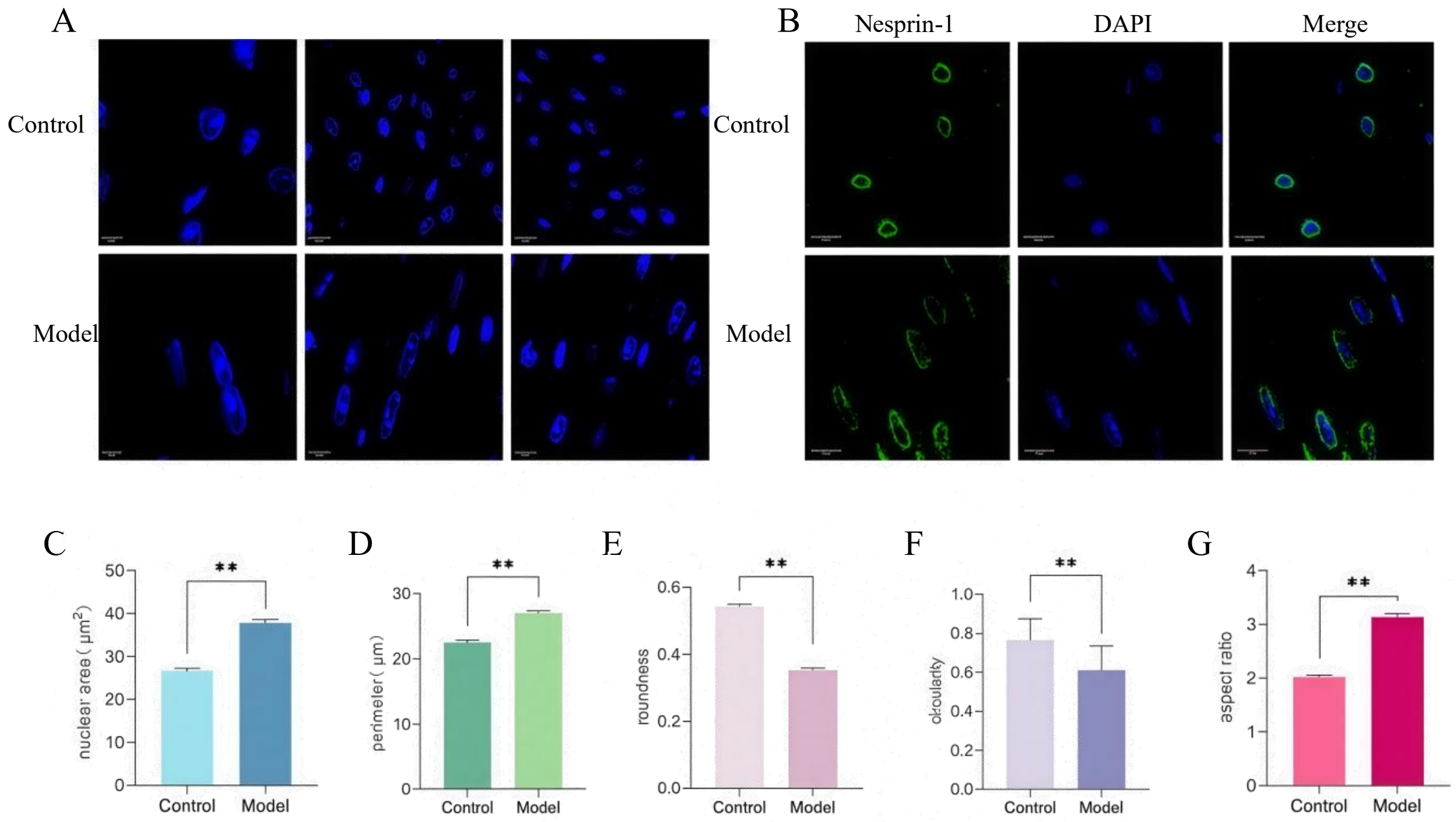

Figure 5: Nuclear Homeostasis Disruption in Heart Failure Mice. Note: A: Changes in nuclear morphology; B: Nesprin-1 immunofluorescence staining; C-G: Nuclear area, nuclear perimeter, nuclear roundness , and aspect ratio of the two groups of mice; n=5; Compared to the control group, ***P*<0.01

4.In a mouse model of heart failure with reduced Nesprin-1 expression, oxidative stress responses were increased

Oxidative stress, characterized by an imbalance between oxidizing agents and antioxidant defenses, predisposes tissues to oxidation. This imbalance fuels neutrophilic inflammation, upregulates protease secretion, and fosters the generation of various oxidative intermediates. Acknowledged as a detrimental factor contributing to aging and various diseases, oxidative stress primarily stems from the overproduction of free radicals within the body. In line with this, Nesprin-1 depletion in Drosophila melanogaster has been correlated with mitochondrial dysfunction, thereby inducing oxidative stress[31]. To investigate this phenomenon, we evaluated the expression levels of malondialdehyde (MDA) and superoxide dismutase (SOD) in cardiac tissue. Notably, compared to the control groups, the model group exhibited heightened MDA activity and diminished SOD content(Fig6A-B)(*P*<0.05). These results indicate that oxidative stress levels were significantly increased and

antioxidant capacity was inhibited in the Nesprin-1 dysregulated HF model.

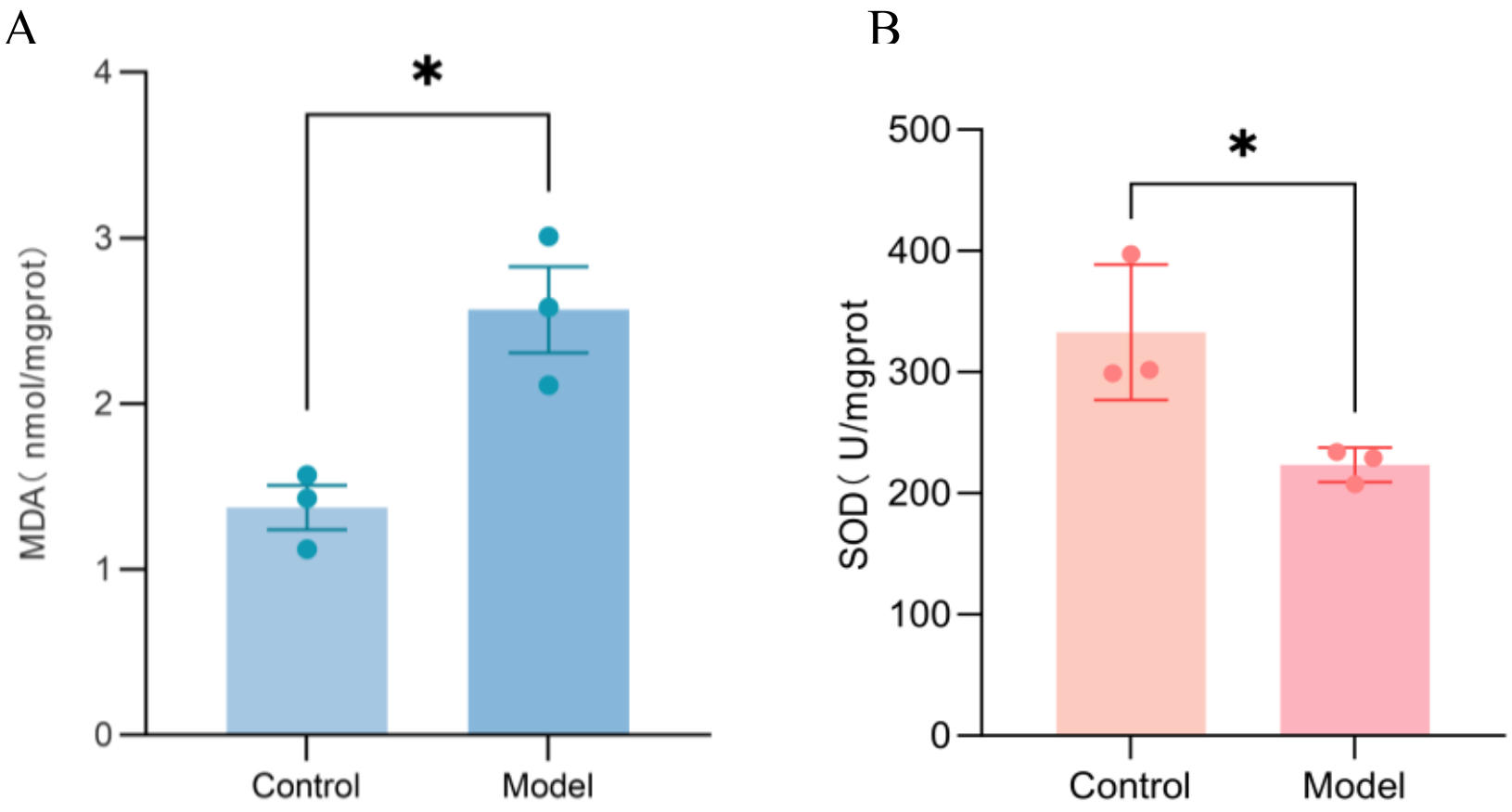

Figure 6: Oxidative stress aggravation in heart failure mice. A-B: Quantitative analysis of MDA and SOD in mouse myocardium; MDA: malondialdehyde; SOD: superoxide dismutase; n=3; compared with the control group, *$P<0.05$

5.The expression of Cx43 was downregulated in the myocardium of mice with dysregulation of Nesprin-1 expression in heart failure

Conduction dysfunction is a hallmark feature observed in the isoproterenol-induced heart failure mouse model. Cx43, primarily localized at the intercalated discs within mature cardiac myocytes, plays a pivotal role in facilitating electrical conduction among myocytes and enabling chemical signaling and energy substrate exchange, essential for coordinating cellular function[32]. Through immunofluorescence analysis, we investigated the expression levels of Cx43 in mice from each experimental group.Our results demonstrate a significant reduction in Cx43 expression specifically within the model group(Fig7A-B)($P<0.01$). In addition, combined with our previous studies, this result will provide a reliable experimental basis for our further study on how the reduction of Nesprin-1 expression affects the localization and function of Cx43 and jointly participates in the occurrence of conduction dysfunction in ISO-induced HF model.

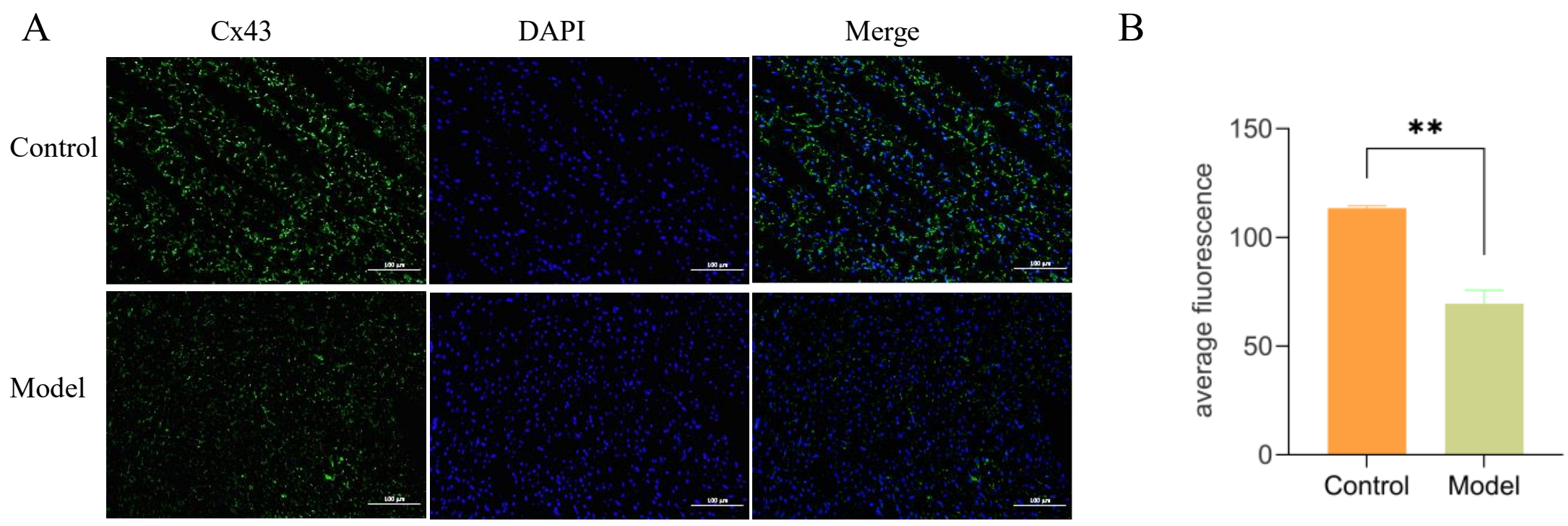

Figure 7: Expression of Cx43 in both groups of mice. A: Cx43 expression was detected by immunofluorescence; B Quantitative analysis of the Cx43; n=5; compared with the control group, ***P*<0.01

6.The myocardial DNA damage was exacerbated in mice exhibiting Nesprin-1 expression dysregulation during heart failure

Decreased Nesprin expression is associated with activated cellular DNA damage in the isoproterenol-induced heart failure mouse model. Nesprin plays a crucial role in maintaining genomic integrity, and its reduction may lead to impaired DNA repair mechanisms[33]. To investigate this phenomenon, immunofluorescence and Western blot analyses were conducted to assess the expression levels of 53BP1 and γH2AX in cardiac tissue. Our results reveal an increased expression of 53BP1 and γH2AX in the model group compared to the control groups(Fig8A-D)(*P*<0.01). In agreement with this, Western blot indicated elevated levels of DNA damage in Nesprin-deficient hearts(Fig9A-C)(*P*<0.01).These results suggest that the decrease in Nesprin-1 expression may interact with the accumulation of DNA damage in the occurrence of HF.

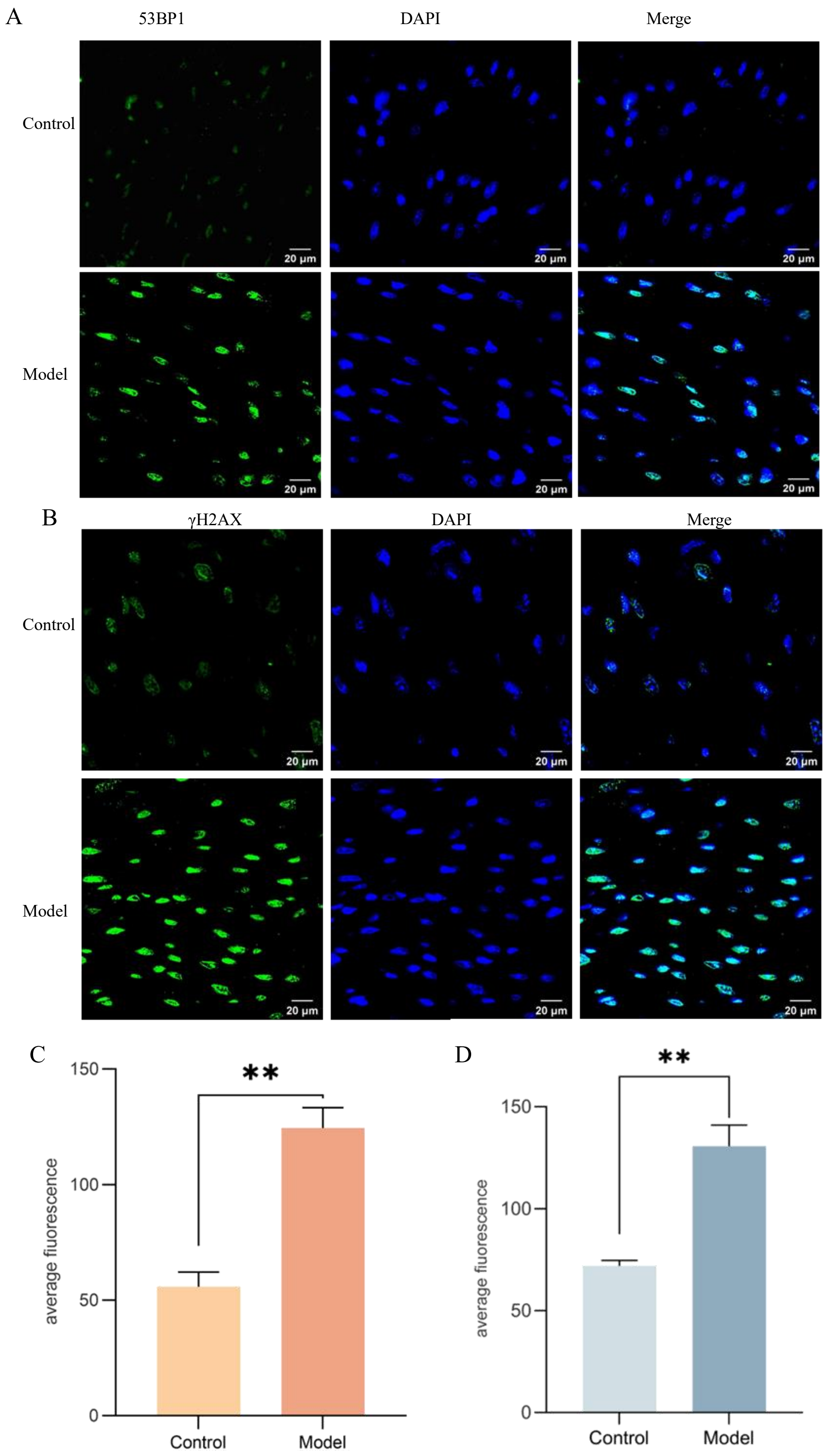

Figure 8: Increased DNA damage in myocardium of mice with heart failure. A-D: Detection of expression of 53BP1 and γH2AX by immunofluorescence; 53BP1: P53 binding protein 1; γH2AX: phosphorylated histone; n=5; compared with the control group,***P*<0.01

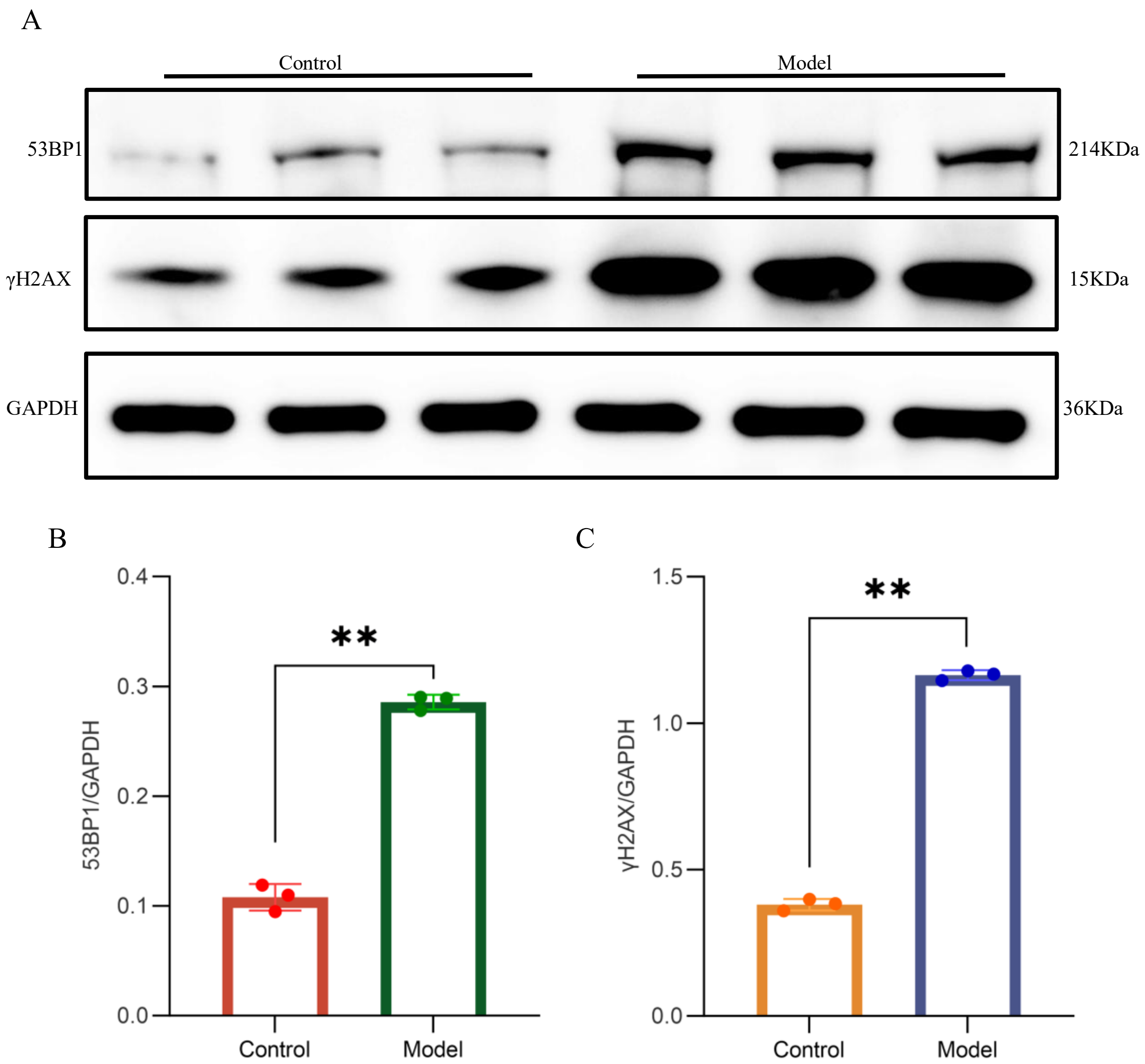

Figure 9: Increased DNA damage in myocardium of mice with heart failure; A-C: Protein quantitative analysis of 53BP1 and γH2AX; 53BP1: P53 binding protein 1; γH2AX: phosphorylated histone; n=3; compared with the control group,***P*<0.01

7.Dysregulation of Nesprin-1 interferes with histone methylation in heart failure

Histone methylation represents a critical modification that alters chromosome structure. Recent studies have highlighted the pivotal role of histone methylation modifications in myocardial hypertrophy, fibrosis, and cardiac function[34, 35]. Through immunofluorescence and Western blot analysis, we assessed the expression levels of H3K9me3 and H3K27me3 in mice from each experimental group. As depicted in the figures, the model group exhibited significantly increase expression levels of H3K9me3 and H3K27me3 compared to the control groups(Fig10A-D)(*P*<0.01). This result is consistent with the Western blot results(Fig11A-C)(*P*<0.01). These findings suggest that the histone methylation pattern disorder in the isoproterenol induced heart failure mouse model may be associated with the decreased level of Nesprin-1.

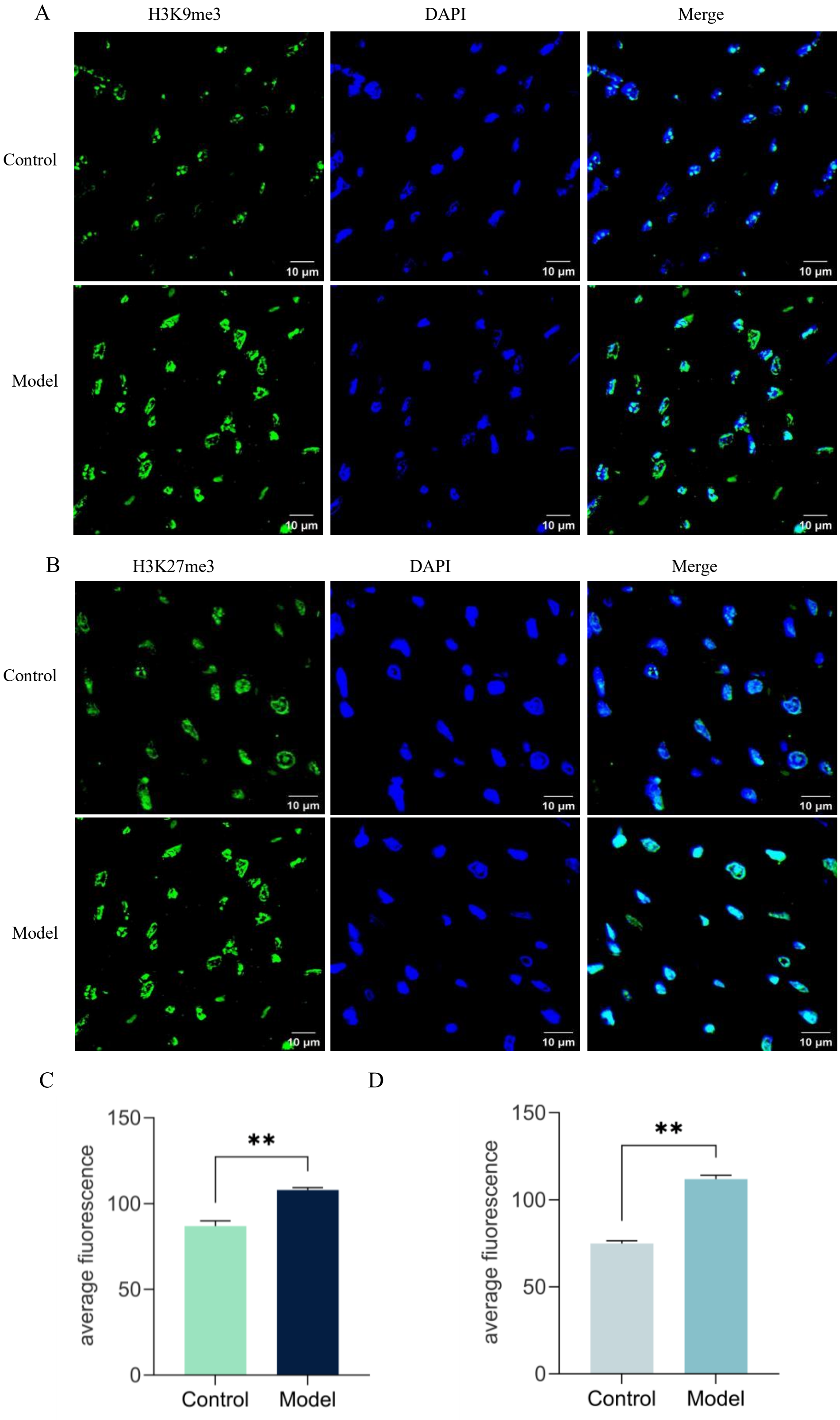

Figure 10: Changes in chromatin recombination in the myocardium of heart failure mice.A-D: Immunofluorescence detection of H3K9me3 and H3K27me3 expression; H3K9me3: trimethylation of histone H3 lysine 9; H3K27me3: trimethylation of histone H3 lysine 27; n=5; compared to the control group,***P*<0.01

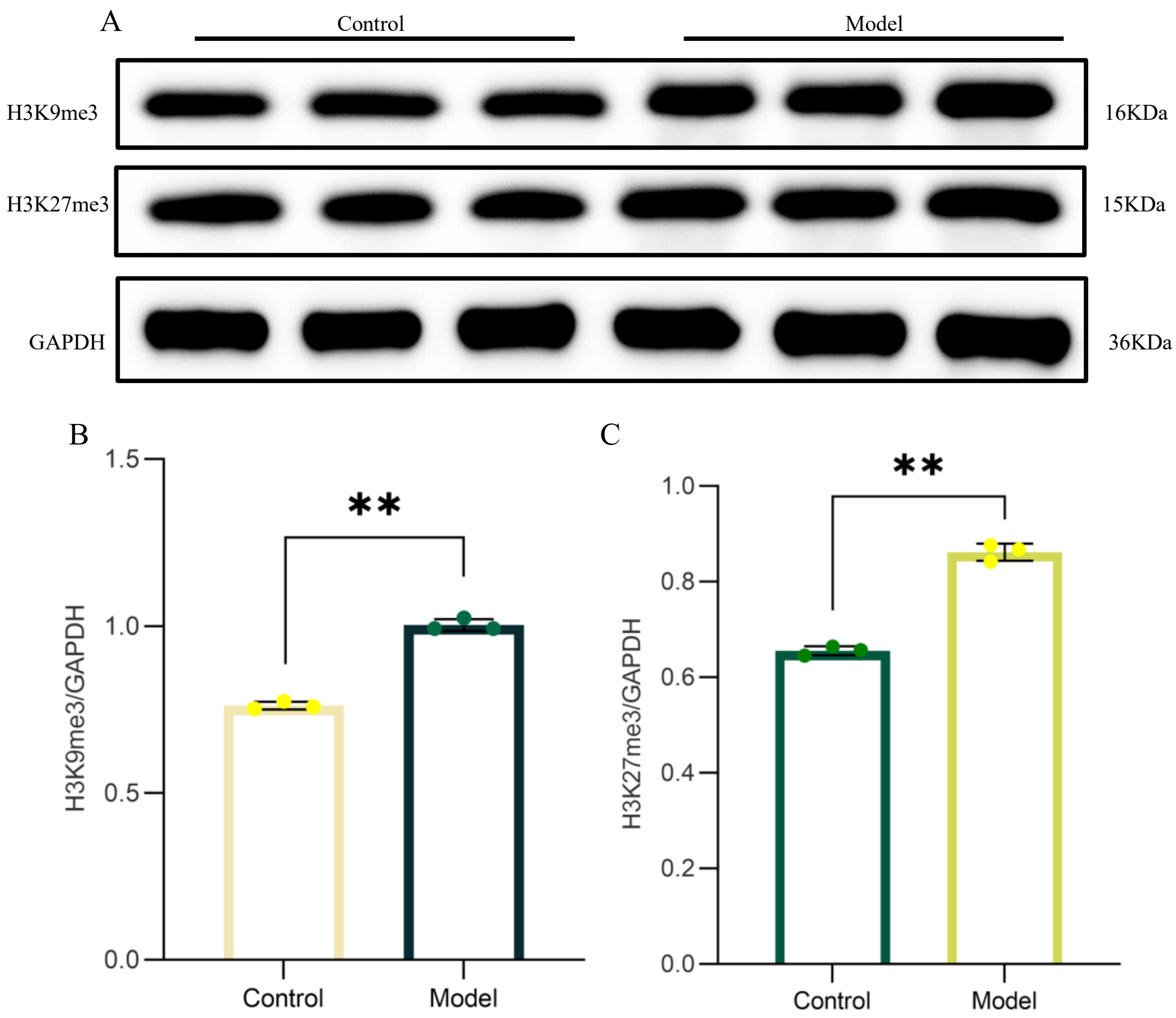

Figure 11: Changes in chromatin recombination in the myocardium of heart failure mice. A-C: Quantitative analysis of H3K9me3 and H3K27me3 proteins; H3K9me3: trimethylation of lysine 9 on histone H3; H3K27me3: trimethylation of lysine 27 on histone H3; n=3; compared with the control group,***P*<0.01

8.ERK pathway is activated in nuclear homeostasis abnormalities mediated by Nesprin-1 expression disorder and heart failure

The ERK pathway is recognized as a pivotal regulator in the development of myocardial hypertrophy. Studies have elucidated the formation of Nesprin-2/ERK complexes at sites of DNA damage in aortic smooth muscle cells, indicating a potential role of Nesprin proteins in modulating ERK signaling[36]. To investigate this phenomenon in the context of heart failure, we employed Western blot analysis to evaluate the expression levels of ERK in cardiac tissue samples from mice in each experimental group. Strikingly, our results revealed a notable increase in ERK expression in the model group compared to the control group, indicative of the activation of the ERK pathway in the cardiac tissue of the isoproterenol-induced heart failure mouse model(Fig12A-C)(*P*<0.01). Combined with the reduced expression of

Nesprin-1 in iprindel induced heart failure mice, we speculate that the reduced expression of Nesprin-1 may be associated with the activation of ERK pathway.

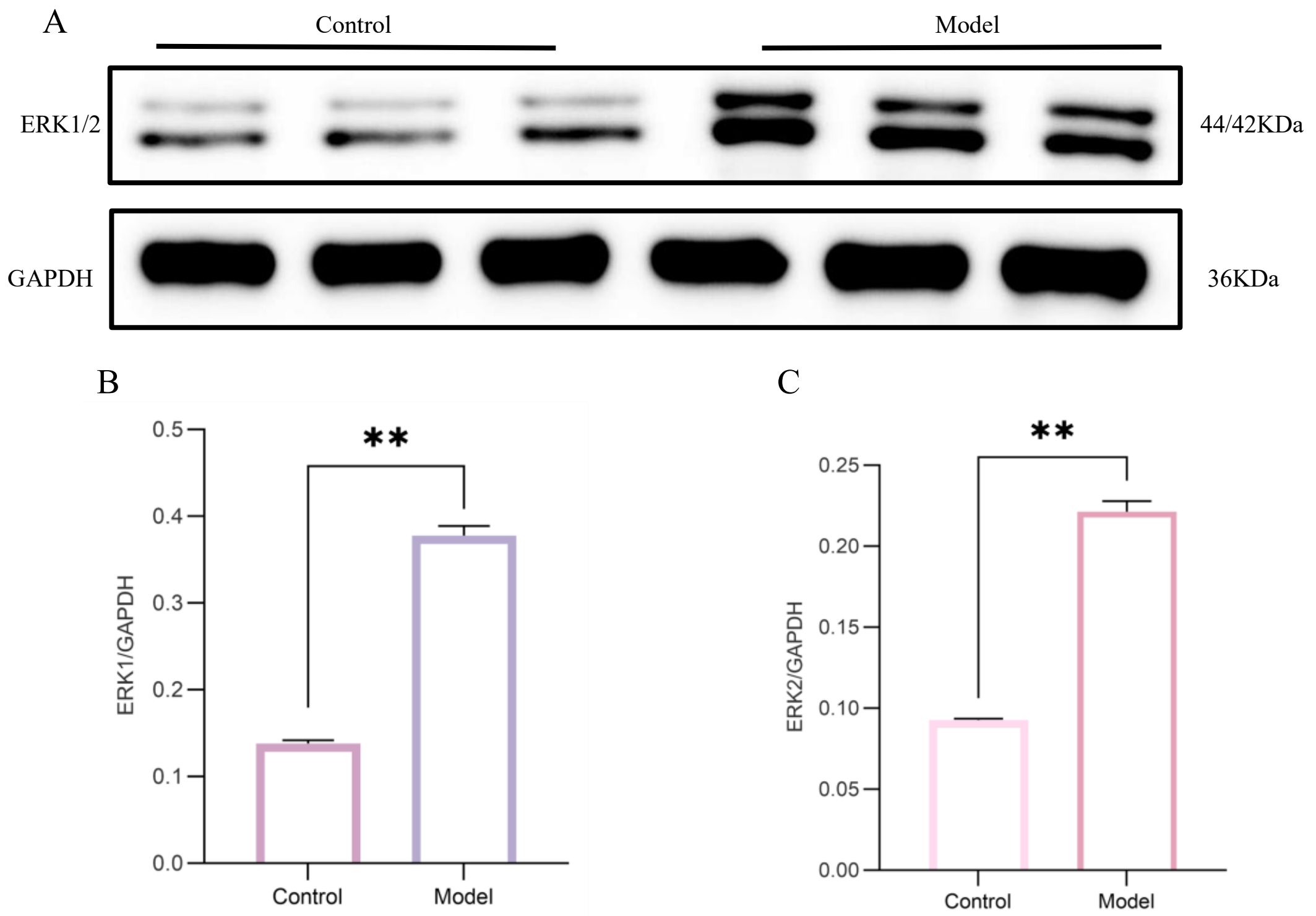

Figure 12: Downregulation of ERK1/2 expression in myocardium of mice with heart failure; A-C: Detection of ERK1/2 expression by protein immunoblotting; ERK: extracellular regulated protein kinase; n=3; compared with the control group,***P*<0.01

Discussion

Heart failure stands as an enduring challenge within global healthcare systems, presenting a formidable burden characterized by its pervasive prevalence, grim prognostic outlook, and profound detriment to patients' quality of life. Despite notable strides in HF management witnessed over recent decades, including the advent of neurohormonal blockade, device-based interventions, and cardiac transplantation, the persistently high rates of mortality and morbidity underscore the insufficiency of current therapeutic strategies[37-39]. Consequently, there exists an imperative mandate for sustained investigative efforts directed towards unraveling the intricate molecular mechanisms governing HF pathogenesis.

In this study, We induced myocardial hypertrophy in mice by intraperitoneal injection of ISO. Our findings indicate that the administration of ISO successfully induced myocardial hypertrophy, characterized by significant changes in heart weight, body weight, and WGA pathological staining results compared to the control group

mice, consistent with previous studies [40, 41]. Long-term stimulation by pathological factors can lead to persistent changes in cardiac structure, resulting in HF [42]. HE staining revealed that Nesprin-1 deficiency exacerbated myocardial structural disorder, vascular proliferation, and myocardial hemorrhage. Additionally, cardiac function tests confirmed the onset of HF in the mice, with significant changes in LVEDd, LVESd, EF, and FS. When myocardial cell volume and pressure overload occur, ventricular muscle cells release BNP, which exerts sodium-losing, diuretic, vasodilatory, and antihypertensive effects [43]. BNP is a crucial biomarker for the clinical diagnosis of HF . Western blotting detected a significant increase in BNP in the myocardial tissue of mice induced by isoproterenol, providing a more reliable basis for confirming the onset of HF in mice.

Nesprin are giant scaffolding proteins located at the NE that play essential roles in maintaining nuclear structure and regulating various cellular processes, including nuclear positioning, cytoskeletal organization, and gene expression[27, 44-48]. While previous research has primarily focused on the role of Nesprin in skeletal and muscular diseases, their involvement in cardiac pathology, particularly HF, remains poorly understood.In this study, we used the ISO-induced mouse heart failure model to initially assess the changes in Nesprin-1 expression and its potential links with nuclear structure, signaling pathways, and gene expression regulation. The ISO-induced HF mouse model established and validated in this study provides new insights into the pathophysiology of heart failure and the role of Nesprin-1 in cardiac function.

Our research has revealed that, compared to the control group, the expression of Nesprin-1 in the myocardium of ISO-induced HF mice is significantly reduced. This finding suggests that changes in Nesprin-1 expression may be linked to pathological remodeling processes associated with HF, such as myocardial hypertrophy and dysfunction. The downregulation of Nesprin-1 may be related to abnormalities in the morphology, nuclear localization, and structural integrity of myocardial cells, which have been preliminarily observed through histopathological examination. However, the specific molecular mechanisms still require further investigation. Additionally, the

dysregulation of Nesprin-1 expression may affect nuclear stability by disrupting the integrity of the nuclear membrane, potentially leading to cellular dysfunction. These findings suggest that, as a key component of the nuclear scaffold and cytoskeletal connection complex, changes in Nesprin-1 expression may be associated with nuclear instability and abnormal cell signaling.

The exact mechanisms underlying Nesprin-1-mediated pathogenesis in HF remain incompletely understood, but our study has shed light on several potential avenues for further investigation. our findings elucidate the multifaceted role of Nesprin-1 in HF pathogenesis, encompassing oxidative stress, electrical conduction abnormalities, and epigenetic dysregulation. First, Nesprin-1 may affect cardiac morphology through interactions with cytoskeletal elements and extracellular matrix proteins, thereby regulating the signaling pathways involved in fibroblast activation. However, the causal relationship between Nesprin-1 and these processes has not been directly demonstrated. For example, how Nesprin-1 affects fibroblast activation by influencing cardiac morphology through interactions with cytoskeletal components and extracellular matrix proteins.

Additionally, Nesprin appears to modulate oxidative stress and DNA damage responses in the heart. Reduced Nesprin expression is associated with heightened oxidative stress, as evidenced by alterations in oxidative stress markers such as malondialdehyde levels and decreased superoxide dismutase content. Increased expression of DNA damage repair proteins, such as γH2AX and 53BP1, further suggests Nesprin's role in preserving genomic integrity and protecting against oxidative stress-induced cardiac injury. Furthermore, the observed reduction in connexin 43 expression in Nesprin-deficient hearts highlights Nesprin's involvement in regulating cardiac conduction and electromechanical coupling. Diminished Cx43 expression may contribute to conduction abnormalities and arrhythmias commonly observed in HF, emphasizing Nesprin's role in regulating both structural and functional aspects of cardiac tissue. Lastly, alterations in histone methylation patterns, specifically changes in H3K9me3 and H3K27me3 levels in Nesprin-deficient hearts, suggest dysregulated epigenetic regulation in HF pathogenesis. These findings

underscore the intricate interplay between Nesprin and epigenetic regulatory mechanisms in maintaining cardiac homeostasis.

Activation of the ERK pathway has been implicated in various hypertrophic stimuli and is known to regulate gene expression, protein synthesis, and cell survival in cardiomyocytes[49-51]. In addition to the identified alterations in Nesprin expression and its implications in cardiac remodeling, our study elucidates the involvement of the ERK signaling pathwaymay be involved in mediating Nesprin-1-induced cardiac hypertrophy and remodeling in response to isoproterenol stimulation. Activation of the ERK pathway has been implicated in various cellular processes, including cell proliferation, differentiation, and survival[52-54]. Here, we demonstrate a significant increase in ERK expression in the cardiac tissue of mice subjected to isoproterenol-induced heart failure, suggesting the activation of the ERK pathway in response to cardiac stress. The observed upregulation of ERK signaling in Nesprin-deficient hearts highlights a potential interaction between Nesprin and the ERK pathway in regulating cardiac function. Previous studies have indicated the formation of Nesprin-2/ERK complexes at sites of DNA damage, implicating Nesprin proteins in the modulation of ERK signaling in other cellular contexts[36]. Our findings extend this understanding to the cardiac setting, where Nesprin deficiency may contribute to ERK pathway activation, thereby exacerbating myocardial injury and dysfunction. The activation of the ERK pathway in response to cardiac stressors, such as isoproterenol administration, suggests a role of ERK signaling in mediating pathological remodeling and maladaptive responses in the heart. Future studies aimed at elucidating the precise mechanisms underlying the crosstalk between Nesprin and the ERK pathway may provide further insights into the molecular mechanisms of heart failure pathogenesis.

The identification of Nesprin-1 as a potential regulator of HF pathogenesis opens up new avenues for therapeutic intervention. Targeting Nesprin-1 and its associated signaling pathways may offer novel strategies for preventing or attenuating adverse cardiac remodeling and dysfunction in HF[55-58]. Small molecule inhibitors, gene therapy approaches, or gene editing techniques could be explored to modulate

Nesprin-1 expression or activity selectively in the heart. Additionally, our study underscores the importance of further research into the molecular mechanisms governing nuclear envelope dynamics and their contribution to cardiovascular disease. In conclusion, our study provides compelling evidence implicating Nesprin-1 in the pathogenesis of HF induced by isoproterenol. By elucidating the role of Nesprin-1 in cardiac remodeling, electrical conduction, we have shed light on novel molecular mechanisms underlying HF pathophysiology. Further investigation into the functional significance of Nesprin-1 and its interactions with key signaling pathways may yield valuable insights into disease progression and facilitate the development of targeted therapies for HF. Ultimately, our findings contribute to a deeper understanding of the complex molecular networks driving HF and offer hope for improved outcomes for patients afflicted with this devastating condition. This comprehensive discussion highlights the significance of our findings and provides a roadmap for future research endeavors aimed at unraveling the intricate molecular mechanisms of HF pathogenesis and identifying innovative therapeutic strategies to combat this disease.

However, this study still has some limitations. Firstly, the lack of specific inhibitors for Nesprin-1, along with the high mortality and lengthy construction period of Nesprin gene knockout mice, prevented us from directly establishing a causal relationship between Nesprin-1, oxidative stress, reduced Cx43 expression, increased DNA damage, and chromatin recombination disorders. Secondly, while we observed that decreased Nesprin-1 expression is associated with various pathological features, such as myocardial structural disorder, changes in oxidative stress markers, and increased expression of DNA damage repair proteins, these findings are only correlational. The specific molecular mechanisms require further functional experiments to confirm. Additionally, this study primarily used ISO-induced HF mouse models. Future studies should validate the role of Nesprin-1 in other HF models, such as those induced by stress overload or myocardial infarction, to enhance the generalizability of the research results. Lastly, regarding the interaction between Nesprin-1 and the ERK signaling pathway and epigenetic regulation (such as histone methylation), this study only provides preliminary clues. The specific mechanisms

need to be elucidated through more in-depth molecular and cellular experiments. In future research, we plan to construct a Nesprin-1-specific knockout model, combined with multi-omics analysis and functional validation, to further uncover its exact role in HF.

Declarations

Ethics approval and consent to participate

Not applicable.

Consent for publication

The manuscript was obtained from all authors for consent for submission and publication

Competing interests

The authors declare no competing interests

Funding

This research was funded by the Hubei Provincial Natural Science Foundation Project (Joint Fund Project, Project No.: 2024AFD143).The funders had no role in study design, data collection and analysis, decision to publish, or preparation of the manuscript.

Authors' contributions

Qin Qin and Dong Cengyuan completed the experiment and wrote the manuscript; Zhou Ziyi participated in some of the experiments and manuscript writing; Liu Yangyuanzhi was involved in the article revision; Professor Cao Chunyu provided guidance on the experimental design; Doctor Teng Lin guided the entire experimental process and manuscript writing.

Availability of Data and Material

The data that support the findings of this study are available from the corresponding author upon reasonable request.

Acknowledgements

I would like to thank CJH for their technical assistance in this experiment. And I am very grateful for the funding provided by the Provincial Natural Science Foundation for this experiment.

Reference

[1] Tanai E, Frantz S. Pathophysiology of Heart Failure [J]. Compr Physiol, 2015, 6(1): 187-214.

[2] McDonagh T A, Metra M, Adamo M, et al. 2021 ESC Guidelines for the diagnosis and treatment of acute and chronic heart failure: Developed by the Task Force for the diagnosis and treatment of acute and chronic heart failure of the European Society of Cardiology (ESC) With the special contribution of the Heart Failure Association (HFA) of the ESC [J]. Rev Esp Cardiol (Engl Ed), 2022, 75(6): 523.

[3] Bozkurt B, Coats A J S, Tsutsui H, et al. Universal Definition and Classification of Heart Failure [J]. Journal of Cardiac Failure, 2021, 27(4): 387-413.

[4] Kemp C D, Conte J V. The pathophysiology of heart failure [J]. Cardiovasc Pathol, 2012, 21(5): 365-71.

[5] Ghionzoli N, Gentile F, Del Franco A M, et al. Current and emerging drug targets in heart failure treatment [J]. Heart Failure Reviews, 2021, 27(4): 1119-36.

[6] Savarese G, Becher P M, Lund L H, et al. Global burden of heart failure: a comprehensive and updated review of epidemiology [J]. Cardiovasc Res, 2023, 118(17): 3272-87.

[7] Shahim B, Kapelios C J, Savarese G, et al. Global Public Health Burden of Heart Failure: An Updated Review [J]. Card Fail Rev, 2023, 9: e11.

[8] Borovac J A, D'Amario D, Bozic J, et al. Sympathetic nervous system activation and heart failure: Current state of evidence and the pathophysiology in the light of novel biomarkers [J]. World J Cardiol, 2020, 12(8): 373-408.

[9] Bencivenga L, Palaia M E, Sepe I, et al. Why Do We Not Assess Sympathetic Nervous System Activity in Heart Failure Management: Might GRK2 Serve as a New Biomarker? [J]. Cells, 2021, 10(2).

[10] Wang J, Dai M, Cao Q, et al. Carotid baroreceptor stimulation suppresses ventricular fibrillation in canines with chronic heart failure [J]. Basic Research in Cardiology, 2019, 114(6).

[11] Huang Y, Liu Y-W, Pan H-Z, et al. Transthoracic Pulmonary Artery Denervation for Pulmonary Arterial Hypertension [J]. Arteriosclerosis, Thrombosis, and Vascular Biology, 2019, 39(4): 704-18.

[12] Triposkiadis F, Briasoulis A, Kitai T, et al. The sympathetic nervous system in heart failure revisited [J]. Heart Fail Rev, 2024, 29(2): 355-65.

[13] Grassi G, Mancia G, Esler M. Central and peripheral sympathetic activation in heart failure [J]. Cardiovasc Res, 2022, 118(8): 1857-71.

[14] Gronda E, Dusi V, D'Elia E, et al. Sympathetic activation in heart failure() [J]. Eur Heart J Suppl, 2022, 24(Suppl E): E4-E11.

[15] Munoz-Durango N, Fuentes C A, Castillo A E, et al. Role of the Renin-Angiotensin-Aldosterone System beyond Blood Pressure Regulation: Molecular and Cellular Mechanisms Involved in End-Organ Damage during Arterial Hypertension [J]. Int J Mol Sci, 2016, 17(7).

[16] Pugliese N R, Masi S, Taddei S. The renin-angiotensin-aldosterone system: a crossroad from arterial hypertension to heart failure [J]. Heart Fail Rev, 2020, 25(1): 31-42.

[17] Ferreira N S, Tostes R C, Paradis P, et al. Aldosterone, Inflammation, Immune System, and Hypertension [J]. Am J Hypertens, 2021, 34(1): 15-27.

[18] Weir M R, Dzau V J. The renin-angiotensin-aldosterone system: a specific target for hypertension management [J]. Am J Hypertens, 1999, 12(12 Pt 3): 205S-13S.

[19] Chen Y, He Q, Mo D-C, et al. The angiotensin receptor and neprilysin inhibitor, LCZ696, in heart failure: A meta-analysis of randomized controlled trials [J]. Medicine, 2022, 101(41).

[20] Heidenreich PA B B, Aguilar D,et al. Correction to: 2022 AHA/ACC/HFSA Guideline for the Management of Heart Failure: A Report of the American College of Cardiology/American Heart Association Joint Committee on Clinical Practice Guidelines [J]. Circulation, 2023, 147(14): e263-e421.

[21] Lachowska K, Gruchala M, Narkiewicz K, et al. Sympathetic Activation in Chronic Heart Failure: Potential Benefits of Interventional Therapies [J]. Curr Hypertens Rep, 2016, 18(7): 51.

[22] von Lueder T G, Kotecha D, Atar D, et al. Neurohormonal Blockade in Heart Failure [J]. Card Fail Rev, 2017, 3(1): 19-24.

[23] Ghionzoli N, Gentile F, Del Franco A M, et al. Current and emerging drug targets in heart failure treatment [J]. Heart Fail Rev, 2022, 27(4): 1119-36.

[24] Crisp M, Liu Q, Roux K, et al. Coupling of the nucleus and cytoplasm: Role of the LINC complex [J]. The Journal of Cell Biology, 2006, 172(1): 41-53.

[25] Meinke P, Nguyen T D, Wehnert M S. The LINC complex and human disease [J]. Biochem Soc Trans, 2011, 39(6): 1693-7.

[26] De Silva S, Catibog N, Shanahan C, et al. Investigating novel roles for nesprin-1 and the LINC complex in Dilated Cardiomyopathy [J]. Cardiovascular Research, 2022, 118(Supplement_1).

[27] Zhou C, Li C, Zhou B, et al. Novel nesprin-1 mutations associated with dilated cardiomyopathy cause nuclear envelope disruption and defects in myogenesis [J]. Hum Mol Genet, 2017, 26(12): 2258-76.

[28] Shanahan CM W P, Metcalfe JC. Isolation of gene markers of differentiated and proliferating vascular smooth muscle cells [J]. Circ Res, 1993, 73(1): 193-204.

[29] Chen Z, Ren Z, Mei W, et al. A novel SYNE1 gene mutation in a Chinese family of Emery-Dreifuss muscular dystrophy-like [J]. BMC Medical Genetics, 2017, 18(1).

[30] Stroud M J, Banerjee I, Veevers J, et al. Linker of Nucleoskeleton and Cytoskeleton Complex Proteins in Cardiac Structure, Function, and Disease [J]. Circulation Research, 2014, 114(3): 538-48.

[31] Lindenboim L, Grozki D, Amsalem-Zafran A R, et al. Apoptotic stress induces Bax-dependent, caspase-independent redistribution of LINC complex nesprins [J]. Cell Death Discov, 2020, 6(1): 90.

[32] Boengler K, Rohrbach S, Weissmann N, et al. Importance of Cx43 for Right Ventricular Function [J]. Int J Mol Sci, 2021, 22(3).

[33] Sur-Erdem I, Hussain M S, Asif M, et al. Nesprin-1 impact on tumorigenic cell phenotypes [J]. Mol Biol Rep, 2020, 47(2): 921-34.

[34] Yao L, He F, Zhao Q, et al. Spatial Multiplexed Protein Profiling of Cardiac Ischemia-Reperfusion Injury [J]. Circ Res, 2023, 133(1): 86-103.

[35] Li J, Sha Z, Zhu X, et al. Targeting miR-30d reverses pathological cardiac hypertrophy [J]. EBioMedicine, 2022, 81: 104108.

[36] Warren D T, Tajsic T, Porter L J, et al. Nesprin-2-dependent ERK1/2 compartmentalisation regulates the DNA damage response in vascular smooth muscle cell ageing [J]. Cell Death Differ, 2015, 22(9): 1540-50.

[37] Collaborators G B D R F. Global burden and strength of evidence for 88 risk factors in 204 countries and 811 subnational locations, 1990-2021: a systematic analysis for the Global Burden of Disease Study 2021 [J]. Lancet, 2024, 403(10440): 2162-203.
[38] Savarese G, Lund L H. Global Public Health Burden of Heart Failure [J]. Card Fail Rev, 2017, 3(1): 7-11.
[39] Sapna F, Raveena F, Chandio M, et al. Advancements in Heart Failure Management: A Comprehensive Narrative Review of Emerging Therapies [J]. Cureus, 2023, 15(10): e46486.
[40] Aimo A, Gaggin H K, Barison A, et al. Imaging, Biomarker, and Clinical Predictors of Cardiac Remodeling in Heart Failure With Reduced Ejection Fraction [J]. JACC Heart Fail, 2019, 7(9): 782-94.
[41] Goetze J P, Bruneau B G, Ramos H R, et al. Cardiac natriuretic peptides [J]. Nat Rev Cardiol, 2020, 17(11): 698-717.
[42] Salah K, Stienen S, Pinto Y M, et al. Prognosis and NT-proBNP in heart failure patients with preserved versus reduced ejection fraction [J]. Heart, 2019, 105(15): 1182-9.
[43] Apel E D, Lewis R M, Grady R M, et al. Syne-1, a dystrophin- and Klarsicht-related protein associated with synaptic nuclei at the neuromuscular junction [J]. J Biol Chem, 2000, 275(41): 31986-95.
[44] De Silva S, Fan Z, Kang B, et al. Nesprin-1: novel regulator of striated muscle nuclear positioning and mechanotransduction [J]. Biochem Soc Trans, 2023, 51(3): 1331-45.
[45] Leong E L, Khaing N T, Cadot B, et al. Nesprin-1 LINC complexes recruit microtubule cytoskeleton proteins and drive pathology in Lmna-mutant striated muscle [J]. Hum Mol Genet, 2023, 32(2): 177-91.
[46] Zhang Q, Ragnauth C D, Skepper J N, et al. Nesprin-2 is a multi-isomeric protein that binds lamin and emerin at the nuclear envelope and forms a subcellular network in skeletal muscle [J]. J Cell Sci, 2005, 118(Pt 4): 673-87.
[47] Rajgor D, Shanahan C M. Nesprins: from the nuclear envelope and beyond [J]. Expert Rev Mol Med, 2013, 15: e5.
[48] Zi-Yi Z, Qin Q, Fei Z, et al. Nesprin proteins: bridging nuclear envelope dynamics to muscular dysfunction [J]. Cell Commun Signal, 2024, 22(1): 208.
[49] Gallo S, Vitacolonna A, Bonzano A, et al. ERK: A Key Player in the Pathophysiology of Cardiac Hypertrophy [J]. Int J Mol Sci, 2019, 20(9).
[50] Mutlak M, Kehat I. Extracellular signal-regulated kinases 1/2 as regulators of cardiac hypertrophy [J]. Front Pharmacol, 2015, 6: 149.
[51] Ramos-Kuri M, Meka S H, Salamanca-Buentello F, et al. Molecules linked to Ras signaling as therapeutic targets in cardiac pathologies [J]. Biol Res, 2021, 54(1): 23.
[52] Sugiura R, Satoh R, Takasaki T. ERK: A Double-Edged Sword in Cancer. ERK-Dependent Apoptosis as a Potential Therapeutic Strategy for Cancer [J]. Cells, 2021, 10(10).
[53] Deschênes-Simard X, Malleshaiah M, Ferbeyre G. Extracellular Signal-Regulated Kinases: One Pathway, Multiple Fates [J]. Cancers (Basel), 2023, 16(1).
[54] Wu J, Chen Y, Li R, et al. Synergistic anticancer effect by targeting CDK2 and EGFR-ERK signaling [J]. J Cell Biol, 2024, 223(1).
[55] Li Y L, Cheng X N, Lu T, et al. Syne2b/Nesprin-2 Is Required for Actin Organization and Epithelial Integrity During Epiboly Movement in Zebrafish [J]. Front Cell Dev Biol, 2021, 9: 671887.

[56] Davidson P M, Battistella A, Dejardin T, et al. Nesprin-2 accumulates at the front of the nucleus during confined cell migration [J]. EMBO Rep, 2020, 21(7): e49910.

[57] Chen S N, Sbaizero O, Taylor M R G, et al. Lamin A/C Cardiomyopathy: Implications for Treatment [J]. Curr Cardiol Rep, 2019, 21(12): 160.

[58] Nicolas H A, Hua K, Quigley H, et al. A CRISPR/Cas9 zebrafish lamin A/C mutant model of muscular laminopathy [J]. Dev Dyn, 2022, 251(4): 645-61.
