# Supplementary material for: Exploring the Role of Nesprin1 in isoproterenol-induced heart failure: Insights into pathogenesis and therapeutic implications
Source: PLoS One. 2026 Aug 11;21(8):e0354482. doi: 10.1371/journal.pone.0354482 (PMC13460607; doi:10.1371/journal.pone.0354482)
Supplement: Supplementary file 3 [file pone.0354482.s005.docx]

**Response to Editorial Office Requests**

Manuscript ID: PONE-D-25-08527R2

Title: Exploring the Role of Nesprin-1 in Isoproterenol-Induced Heart Failure: Insights into Pathogenesis and Therapeutic Implications

Dear Editor,

Thank you for the opportunity to revise our manuscript to meet PLOS ONE’s submission requirements. We have carefully addressed all the issues raised in your email. Please find our point-by-point responses below.

***Point 1:*** *Please upload a Response to Reviewers letter which should include a point by point response to each of the points made by the Editor and / or Reviewers.*

**Response:** We have prepared and uploaded this point-by-point response letter as requested.

**Reviewer #1
Minor comment:**

**Comment 1:** Throughout the manuscript:The English is understandable, but not highly polished. Many sentences are wordy and awkwardly structured, and some terms are misused. A professional language review is needed to improve fluency and scientific clarity.Exemple L221. The authors wrote: "The ross and pathological examination". did they mean "The gross pathological examination"?Many other places.Exemple L364 “significantly reduction” should be corrected to “significant reduction.” A careful proofreading of the section for grammatical accuracy is recommended.

**Response 1:**Thanks for your constructive feedback on our manuscript. We appreciate your careful assessment of the grammar and language issues. Each sentence has been carefully checked and revised to improve readability.The red indicates the modified text.

**Comment 2:** In the result presentation, the authors mention "significant" differences. These sentences should explicitly state the precise p-values and statistical tests.

**Response 2:**Thank you very much for your valuable suggestions. We have made changes to the original text according to your suggestions.

**Comment 3:** Overall, and specifically in the discussion section:Some sentences are complex and could benefit from restructuring to enhance readability. For example, the transition between the introduction of Nesprin1 and its connection to HF pathogenesis could be smoother.

**Response 3:** We feel great thanks for your professional review work on our article.We have made corresponding modifications to the discussion section according to your suggestions. You can see the specific modifications in the original text.

**Comment 4:** Ensure that the argument follows a logical progression, from the established burden of HF to the novel contributions of this study, in the discussion section.

**Response 4:**We sincerely thank you for your careful reading.In the discussion section, we first outlined the significant risks of heart failure and the necessity of conducting research on it. We then confirmed the successful construction of the heart failure mouse model by analyzing the basic physiological parameters, pathological staining, and cardiac color Doppler ultrasound results in mice. Next, by analyzing various pathological manifestations in heart failure mice, we explored potential directions for the role of Nesprin-1 in the disease process. Finally, we summarized the innovations and limitations of this study.

**Major comments:**

**Comment 1:** The authors suggest that Nesprin1 downregulation is causative, but this is not experimentally proven, unless I did not read correctly (authors did not use genetic knockdown or RNAi), wherease, the findings are correlational (i.e., Nesprin1 is reduced in heart failure). Would suggest the authors to clarify this limitation.

**Response 1:**Thank you for your valuable and practical suggestions. As you understand, this study has confirmed a series of pathological phenomena in heart failure mice with reduced Nesprin1 levels, which may be associated with the downregulation of Nesprin-1. However, due to certain objective reasons, this conclusion has not been experimentally verified. This limitation is addressed in the revised discussion. We will use additional techniques in future studies to further investigate this area.

**Comment 2:** The Authors suggest that reduced Nesprin1 leads to ERK activation. Would you please elaborate with more mechanistic evidence? I understand the discussion highlights the significance of Nesprin1, however, it would be beneficial to include a comparison with other known mechanisms of HF progression. More explicit connections between previous research and the current findings could strengthen the justification for Nesprin1’s role.I would suggest the authors to add more reference to prior studies when applicable) regarding the discussion on Nesprin1’s involvement in oxidative stress, nuclear homeostasis, and the ERK pathway.

**Response 2:** Sincerely thank you for your insights into the paper. I understand the rationality of your suggestion to add a comparison of Nesprin1 with other mechanisms of heart failure and a more detailed explanation of the mechanism. After careful discussion by the team, we believe that this study is the first exploratory work to reveal the expression changes of Nesprin1 in ISO-induced heart failure models and its association with nuclear homeostasis imbalance and oxidative stress. Maintaining the existing discussion framework will better highlight the original value of this new finding.Regarding the molecular mechanism of Nesprin1 down-regulation and ERK activation, this is indeed a question of great scientific value, but considering that this study mainly focuses on establishing phenotypic associations, detailed mechanism analysis will be the focus of our follow-up research using the gene knockout animal model we are building. Your valuable suggestions have pointed to important follow-up research directions for us, including systematic comparison of the relationship between Nesprin1 and other heart failure pathways, and in-depth analysis of the molecular mechanisms of its regulation of ERK pathways.

We earnestly hope that you will understand the limitations of the current research phase, and we commit to further exploring these key scientific questions in future work.

**Comment 3:** While increased MDA and decreased SOD suggest oxidative stress, the study does not establish whether oxidative stress is the cause or consequence of Nesprin1 downregulation.

**Response 3:**I sincerely appreciate this insightful comment. My data demonstrate that ISO-induced heart failure mice with Nesprin-1 downregulation exhibit increased MDA and decreased SOD activity, indicating enhanced oxidative stress. However, as the reviewer astutely noted, the current study cannot definitively determine whether oxidative stress drives or results from Nesprin-1 suppression.

I recognize that Nesprin-1’s role in cardiac pathology remains underexplored, with limited existing literature. Nevertheless, given heart failure’s clinical significance and prior evidence of Nesprin-1’s functional importance in other disease contexts, along with its myocardial-specific isoform expression patterns, this study was designed to investigate three key aspects: Nesprin-1’s expression profile in ISO-induced HF, its potential functional contributions to disease progression, and possible mechanistic pathways.

These findings establish an initial association between Nesprin-1 dysregulation and oxidative stress in HF, creating a foundation for future mechanistic studies. I am currently generating Nesprin-1 knockout mice to specifically examine the causal relationship between Nesprin-1 deficiency and oxidative stress, elucidate the involved molecular pathways, and characterize its precise regulatory role in HF pathogenesis. I look forward to sharing these more definitive results in future work.

**Comment 4:**Overall, the study is scientifically sound but needs improved language, better statistical transparency, and stronger mechanistic evidence to fully support its conclusions.

**Response 4**:Thank you for your valuable comments. I have made the corresponding changes.

**Comment 5:**Major revisions are recommended to improve the manuscript’s clarity, presentation, and interpretation of findings.

**Response 5:**Thank you very much for your valuable suggestions. In the original text, I have selected a clearer picture and modified the language expression and result explanation of the whole text to improve the readability of the article.

**Reviewer #2:**

**Comment 1:**Abstract:

1. BNP should be spelled out as the term was stated the first time in the article

2. CX43 and Nesprin 1 expression were both determined using immunofluorescence analysis, hence can be stated in one sentence.

3. BNP, CX43 and Nesprin 1 should be briefly introduced in the abstract.

4. Please state the cardiac parameters examined.

5. Any gene manipulation done before the conclusion made by stating that “This study elucidates the critical role of Nesprin1 in isoproterenol-induced……” ?

**Response 1:**Thank you very much for your suggestions and questions. Firstly, the issues with the wording in the abstract have been corrected in the original text. Secondly, regarding whether gene manipulation was performed, my response is that although gene mice were constructed, this experiment did not include this part. The primary goal of this study is to provide a research foundation for further studies on the specific mechanisms of Nesprin-1 in the development of heart failure. Therefore, no gene manipulation was conducted in this study.

**Comment 2:**Introduction

The background and rationale were sufficiently addressed

**Response 2:**I sincerely thank you for your full affirmation of the background and argumentation basis in the introduction of this study. I am very glad that the scientific basis and clinical relevance of this study have been clearly and effectively expounded.

**Comment 3:**Materials and Methods:

General comments: Technical details are not sufficient

**Response 3:**Thank you for your valuable suggestions. Under your guidance, I have modified the details of the materials and methods section in the article. See the red font for the specific content.

**Comment 4:**Drugs and Chemicals

Catalog no. of the chemicals/products used were not stated

**Response 4:**Thank you for pointing this out. I have added the catalogue number of the important chemicals/products in the relevant section.

**Comment 5:**Establishment of Isoproterenol-Induced Heart Failure Model in Mice

Sex and Age of the animals were not stated

**Response 5:**Thank you for your suggestion. In line 140 of the article, it has been stated that the animals used in this study are male C57BL/6 mice with a weight of 20-22g.

**Comment 6:**Cardiac Function Assessment

Specific name of the statistical test was not stated

**Response 6:**Thank you for your suggestion. The relevant testing methods have been explained in the last part of the methodology.

**Comment 7:**Measurement of Body and Heart Weight

Clarify the timing of which the mice were anesthetized

**Response 7:**Thank you for raising this concern. In this study, mouse body weight was measured using an electronic scale while the animals were conscious, thereby avoiding the effects of repeated anesthesia. All mice were euthanized 24 hours after the final administration, followed by cardiac tissue collection and weighing. No anesthetic agents were used during any body weight measurements throughout the study.

**Comment 8:**The Biochemical Kits Detects The Oxidative Stress Indicators

Please simplify the demonstration of protein concentration calculation

**Response 8:**Thank you! I have simplified the MDA/SOD calculation formula in the original text.

**Comment 9:**Immunofluorescence Staining Protocol for Mouse Cardiac Tissue

The model of the microscope and the software were not stated

**Response 9:**Thank you for your insightful comments on the manuscript.I have updated the microscope model and software used for immunofluorescence in this article.

**Comment 10:**Western Blot Experimental Procedure:1. The catalog no and the company which RIPA buffer, phosphate and protease inhibitors were purchased from should be stated. 2. Details of gel documentation system and densitometry analysis software should be stated

**Response 10:**Thank you for your very meaningful suggestion. According to your instructions, the relevant parts have been described in the section of Materials and Methods respectively.

**Comment 11:**Results:1. Sample size determination (animal numbers) was not demonstrated/explained2. Western blot in Figure 2 was not labelled.3. Specific statistical test for each assay was not stated clearly4. Importantly knockdown of Nesprin 1 was not demonstrated and hence the data doesn’t support the stated results.Discussion:The data provided doesn’t support the discussion as knockdown model of Nesprin 1 was not used

**Response 11:**I sincerely appreciate your valuable comments on our manuscript. We have carefully examined all the issues you raised and made corresponding revisions. Regarding the sample size, we have supplemented the figure legends in the Results section to clarify that the sample size meets statistical requirements. For the oversight in labeling Figure 2 Western Blot, we have re-examined the original data and improved the labeling of experimental groups, molecular weight markers, and internal reference protein bands.

In terms of statistical methods, we have added clarification that two-sample t-tests were used for normally distributed data (confirmed by Shapiro-Wilk test) and Mann-Whitney U tests for non-normally distributed data. We fully understand your concern regarding the validation of Nesprin 1 knockdown efficiency. This study primarily focuses on preliminary exploration of Nesprin-1 expression changes and their pathological significance in the ISO-induced heart failure model. We are currently developing Nesprin-1 knockout mouse models to enable more systematic mechanistic studies in the future.

The key findings of this study include: significant downregulation of Nesprin-1 expression in the ISO-induced heart failure model, accompanied by pathological phenomena such as nuclear homeostasis imbalance and aggravated oxidative stress, which may be related to ERK signaling pathway activation. We sincerely accept your suggestions, which are invaluable for improving the completeness of this research. While this study has certain limitations, it establishes an important foundation for subsequent mechanistic investigations. We are fully prepared to provide any additional materials as needed.

**Comment 12:**Declarations:Ethics approval and consent to participate;Animal ethics approval no. was not stated in the main text;Availability of Data and Material:While original western blots were provided, other minimal data sets were not included in the supplement information sheet

**Response 12:** I sincerely appreciate your valuable feedback regarding ethical approval and data accessibility in this paper. Your suggestions to enhance research transparency and reproducibility are of great importance to me. Concerning the animal ethics review, I have supplemented complete information in the "Materials and Methods" section: "All mice were housed in a Specific Pathogen-Free (SPF) facility at China's Three Gorges University (Yichang, Hubei Province). The experimental animal quality certification number is 42010200009428, and the experimental animal use permit number is 00305510. All procedures complied with international standards for laboratory animal care and use." Regarding data availability, although I have provided all Western blot raw images in the supplementary materials, I recognize the importance of additional data. The foundational datasets supporting key research findings have been systematically organized and are available to corresponding authors upon request. This clarification has been incorporated into the "Data Availability Statement" section of the paper. Thank you for your attention to these critical aspects of my work, and I hope this explanation addresses your concerns. If any other supplementary materials are required, I will provide them at any time.

***Point 2:*** *Your abstract cannot contain citations. Please remove any existing citations from the abstract section. You may only include citations in the body text of the manuscript, and please ensure that they remain in ascending numerical order on first mention.*

**Response:** We have thoroughly checked the manuscript and completely removed all citations from the Abstract section. Furthermore, we have updated the reference list to ensure that all citations in the body text remain in ascending numerical order upon their first mention.

***Point 3:*** *To comply with PLOS ONE submissions requirements, in your Methods section, please provide additional information regarding the experiments involving animals and ensure you have included details on (1) methods of sacrifice, and (2) efforts to alleviate suffering.*

**Response:** Thank you for pointing out this important compliance requirement. We have strictly updated the Methods section to align with PLOS ONE’s animal research guidelines.

Specifically, we have added detailed descriptions regarding:

(1) Methods of sacrifice: Mice were euthanized via rapid exsanguination following swift surgical excision of the heart under deep anesthesia in 3% isoflurane. The adequacy of anesthesia was strictly verified by the loss of the pedal withdrawal reflex prior to any surgical intervention to ensure a painless procedure.

(2) Efforts to alleviate suffering: We added details regarding our daily health and behavioral monitoring protocols, efforts to minimize handling stress during intraperitoneal injections, and the establishment of humane endpoints.

These detailed modifications have been incorporated into the "Measurement of Body and Heart Weight" subsection in the revised manuscript ( Page 6, Lines 156 – 172 ). We have also ensured that our Ethical Approval information is clearly stated at the part of “Establishment of Isoproterenol-Induced Heart Failure Model in Mice”.

We hope these revisions meet the journal's requirements and look forward to the scientific review process.

Sincerely,

Teng Lin, MD

Department of Cardiology/Yichang centre people's hospital

The First College of China Three Gorges University

No.183, Yiling Road, Yichang, 443003, China

Email:tenglin@ctgu.edu.cn
