# Supplementary material for: Exploring the Role of Nesprin1 in isoproterenol-induced heart failure: Insights into pathogenesis and therapeutic implications
Source: PLoS One. 2026 Aug 11;21(8):e0354482. doi: 10.1371/journal.pone.0354482 (PMC13460607; doi:10.1371/journal.pone.0354482)
Supplement: Supplementary file 4 [file pone.0354482.s006.docx]

Dear Editor,

Thank you for providing us with the opportunity to resubmit our manuscript titled "Exploring the Role of Nesprin-1 in Isoproterenol-Induced Heart Failure: Insights into Pathogenesis and Therapeutic Implications" (Manuscript ID: PONE-D-25-08527R2).

We have carefully addressed all the technical and language concerns raised in the previous editorial correspondence. In addition to the requested revisions, we would like to clarify three specific administrative changes:

**1. Reinstatement of the Manuscript**

First, we sincerely apologize for a technical error during the submission process. The "Decline to Resubmit" option was accidentally selected. We have since reinstated the submission and wish to confirm our continued commitment to the publication process in PLOS ONE. We apologize for any confusion this may have caused the editorial office.

**2. Update to Institutional Affiliation**

The nomenclature of the primary institution has been updated to " Department of Cardiology, The First College of Clinical Medical Science, China Three Gorges University & Yichang Central People's Hospital, Yichang, China". This change was made to strictly adhere to the latest official standardized naming conventions issued by our university for all newly published research.

**3. Response to Editorial and Language Comments**

3.1. "The manuscript still requires professional language editing..."

Response: We entirely agree. Prior to this resubmission, the entire manuscript underwent comprehensive professional English language editing. We have corrected grammatical errors, resolved tense inconsistencies particularly in the Methods section , fixed plural/singular misuses, and eliminated awkward phrasing to ensure scientific concepts are communicated clearly and accurately.

3.2. "...where there are confusion in the subtitles of the results section..."

Response: Thank you for pointing this out. We reviewed the subtitles in the Results section and found that their formatting was indeed inconsistent. We have now standardized all subtitles in the Results section into concise, unified noun phrases to improve readability and structural flow.

3.3. "...as well as the revised sections in the discussion (duplications and repetition in the limitations of current study). This has resulted in difficulty in following through the manuscript flow and scientific concepts."

Response: We apologize for the redundancy in our previous draft. We have carefully reviewed and rewritten the Discussion section, with a specific focus on the "Limitations" paragraph. We removed the repetitive statements regarding the lack of direct causality and the observational nature of the study. The revised Discussion is now more concise, and the logical flow connecting Nesprin-1 downregulation to specific pathological features has been significantly improved. Sentence fragments and structural redundancies have been fully resolved.

**4. Response to Journal Requirements:**

4.1. Reference List Review:

Response: As requested, we have thoroughly reviewed our reference list. We confirm that the reference list is complete, properly formatted, and correct. We have verified that none of the cited papers have been retracted.

4.2. Figure Guidelines Check:

Response: We have reviewed all figures against the journal's technical requirements using the provided guidelines and the PLOS figure tool (NAAS). All figures have been checked to ensure they meet the publication quality standards regarding resolution, format, and legibility.

We hope that these comprehensive revisions have satisfactorily addressed your concerns and that the manuscript is now suitable for publication in your journal. Thank you again for your time and continuous support.

**5. Update to Authorship**

We have added one co-author, mengpan liu, to the manuscript. This individual provided significant intellectual contributions during this revision stage, particularly in the extensive professional language editing, structural reorganization of the Results and Discussion sections, and critical review of the final version. Crucially, we wish to state that all original authors have reached a unanimous consensus regarding this addition. There are no disputes over authorship. If required by the Editorial Office, we are prepared to provide a formal document signed by all authors to verify this agreement. Additionally, we have also corrected some typographical errors and standardized the capitalization of author names in the submission system to ensure accurate indexing.

Best regards,

Dr. Lin Teng

Department of Cardiology, Yichang Central People's Hospital

College of Basic Medical Sciences, China Three Gorges University

E-mail: tenglin@ctgu.edu.cn
