# Supplementary material for: Exploring the Role of Nesprin1 in isoproterenol-induced heart failure: Insights into pathogenesis and therapeutic implications
Source: PLoS One. 2026 Aug 11;21(8):e0354482. doi: 10.1371/journal.pone.0354482 (PMC13460607; doi:10.1371/journal.pone.0354482)
Supplement: Supplementary file 5 [file pone.0354482.s001.zip › Photo description of the supplementary material.docx]

Photo description of the supplementary material

Panel A represents the protein blot analysis shown in Figure 2 D. Panel B represents protein blot analysis of the internal reference GAPDH. Panel C represents the protein blot analysis shown in Figure 8 E. Panel D represents the protein blot analysis shown in Figure 9 E.Panel E shows from left to right the protein blot analysis shown in Figure 9 E, 10 A and 8 E, respectively.
