# Supplementary material for: Exploring the Role of Nesprin1 in isoproterenol-induced heart failure: Insights into pathogenesis and therapeutic implications
Source: PLoS One. 2026 Aug 11;21(8):e0354482. doi: 10.1371/journal.pone.0354482 (PMC13460607; doi:10.1371/journal.pone.0354482)
Supplement: Peer review history

# Peer review: Decision Letter 0

**DOI:** 10.1371/journal.pone.0354482.r001

**Authors:** Wei Hsum Yap

4 Jun 2025

Dear Dr. Teng,

Thank you for submitting your manuscript to PLOS ONE. After careful consideration, we feel that it has merit but does not fully meet PLOS ONE’s publication criteria as it currently stands. Therefore, we invite you to submit a revised version of the manuscript that addresses the points raised during the review process.

==============================

The submission presented by Lin Teng et al. evaluated the role of Nesprin1 in isoproterenol-induced heart failure and its implications for cardiac pathogenesis mechanism. The results provide potential implications for understanding the molecular mechanisms of cardiac dysfunction. Overall, the study have used multiple experimental approaches, including echocardiography, immunofluorescence, Western blotting, and histological analysis, which strengthens the study. Nevertheless, there are areas that could be improved to enhance clarity, readability, and scientific rigor. Specifically, the claim on the causative role of Nespirin1 in HF is unsupported with specific experimental validation, where the findings suggested correlational implications. Suggest revising the manuscript according to reviewers comments and improve the overall paper interpretations.

==============================

Please submit your revised manuscript by Jul 19 2025 11:59PM. If you will need more time than this to complete your revisions, please reply to this message or contact the journal office at plosone@plos.org. When you're ready to submit your revision, log on to https://www.editorialmanager.com/pone/ and select the 'Submissions Needing Revision' folder to locate your manuscript file.

- A rebuttal letter that responds to each point raised by the academic editor and reviewer(s). You should upload this letter as a separate file labeled 'Response to Reviewers'.
- A marked-up copy of your manuscript that highlights changes made to the original version. You should upload this as a separate file labeled 'Revised Manuscript with Track Changes'.
- An unmarked version of your revised paper without tracked changes. You should upload this as a separate file labeled 'Manuscript'.

If you would like to make changes to your financial disclosure, please include your updated statement in your cover letter. Guidelines for resubmitting your figure files are available below the reviewer comments at the end of this letter.

If applicable, we recommend that you deposit your laboratory protocols in protocols.io to enhance the reproducibility of your results. Protocols.io assigns your protocol its own identifier (DOI) so that it can be cited independently in the future. For instructions see: https://journals.plos.org/plosone/s/submission-guidelines#loc-laboratory-protocols. Additionally, PLOS ONE offers an option for publishing peer-reviewed Lab Protocol articles, which describe protocols hosted on protocols.io. Read more information on sharing protocols at https://plos.org/protocols?utm_medium=editorial-email&utm_source=authorletters&utm_campaign=protocols.

We look forward to receiving your revised manuscript.

Kind regards,

Wei Hsum Yap

Academic Editor

PLOS ONE

Journal Requirements:

When submitting your revision, we need you to address these additional requirements.

1.Please ensure that your manuscript meets PLOS ONE's style requirements, including those for file naming. The PLOS ONE style templates can be found at

https://journals.plos.org/plosone/s/file?id=wjVg/PLOSOne_formatting_sample_main_body.pdf and

https://journals.plos.org/plosone/s/file?id=ba62/PLOSOne_formatting_sample_title_authors_affiliations.pdf

2. To comply with PLOS ONE submissions requirements, in your Methods section, please provide additional information regarding the experiments involving animals and ensure you have included details on (1) methods of sacrifice, and (2) efforts to alleviate suffering.

3. Please note that funding information should not appear in any section or other areas of your manuscript. We will only publish funding information present in the Funding Statement section of the online submission form. Please remove any funding-related text from the manuscript.

4. Thank you for stating the following financial disclosure:

“This research was funded by the Hubei Provincial Natural Science Foundation Project (Joint Fund Project, Project No.: 2024AFD143).”

Please state what role the funders took in the study.  If the funders had no role, please state: "The funders had no role in study design, data collection and analysis, decision to publish, or preparation of the manuscript."

If this statement is not correct you must amend it as needed.

Please include this amended Role of Funder statement in your cover letter; we will change the online submission form on your behalf.

5. We note that your Data Availability Statement is currently as follows: All relevant data are within the manuscript and in Supporting Information files.

Please confirm at this time whether or not your submission contains all raw data required to replicate the results of your study. Authors must share the “minimal data set” for their submission. PLOS defines the minimal data set to consist of the data required to replicate all study findings reported in the article, as well as related metadata and methods (https://journals.plos.org/plosone/s/data-availability#loc-minimal-data-set-definition).

For example, authors should submit the following data:

- The values behind the means, standard deviations and other measures reported;

- The values used to build graphs;

- The points extracted from images for analysis.

Authors do not need to submit their entire data set if only a portion of the data was used in the reported study.

If your submission does not contain these data, please either upload them as Supporting Information files or deposit them to a stable, public repository and provide us with the relevant URLs, DOIs, or accession numbers. For a list of recommended repositories, please see https://journals.plos.org/plosone/s/recommended-repositories.

If there are ethical or legal restrictions on sharing a de-identified data set, please explain them in detail (e.g., data contain potentially sensitive information, data are owned by a third-party organization, etc.) and who has imposed them (e.g., an ethics committee). Please also provide contact information for a data access committee, ethics committee, or other institutional body to which data requests may be sent. If data are owned by a third party, please indicate how others may request data access.

6. When completing the data availability statement of the submission form, you indicated that you will make your data available on acceptance. We strongly recommend all authors decide on a data sharing plan before acceptance, as the process can be lengthy and hold up publication timelines. Please note that, though access restrictions are acceptable now, your entire data will need to be made freely accessible if your manuscript is accepted for publication. This policy applies to all data except where public deposition would breach compliance with the protocol approved by your research ethics board. If you are unable to adhere to our open data policy, please kindly revise your statement to explain your reasoning and we will seek the editor's input on an exemption. Please be assured that, once you have provided your new statement, the assessment of your exemption will not hold up the peer review process.

7. PLOS requires an ORCID iD for the corresponding author in Editorial Manager on papers submitted after December 6th, 2016. Please ensure that you have an ORCID iD and that it is validated in Editorial Manager. To do this, go to ‘Update my Information’ (in the upper left-hand corner of the main menu), and click on the Fetch/Validate link next to the ORCID field. This will take you to the ORCID site and allow you to create a new iD or authenticate a pre-existing iD in Editorial Manager.

8. Please include your full ethics statement in the ‘Methods’ section of your manuscript file. In your statement, please include the full name of the IRB or ethics committee who approved or waived your study, as well as whether or not you obtained informed written or verbal consent. If consent was waived for your study, please include this information in your statement as well.

9. Please include captions for your Supporting Information files at the end of your manuscript, and update any in-text citations to match accordingly. Please see our Supporting Information guidelines for more information: http://journals.plos.org/plosone/s/supporting-information.

10. PLOS ONE now requires that authors provide the original uncropped and unadjusted images underlying all blot or gel results reported in a submission’s figures or Supporting Information files. This policy and the journal’s other requirements for blot/gel reporting and figure preparation are described in detail at https://journals.plos.org/plosone/s/figures#loc-blot-and-gel-reporting-requirements and https://journals.plos.org/plosone/s/figures#loc-preparing-figures-from-image-files. When you submit your revised manuscript, please ensure that your figures adhere fully to these guidelines and provide the original underlying images for all blot or gel data reported in your submission. See the following link for instructions on providing the original image data: https://journals.plos.org/plosone/s/figures#loc-original-images-for-blots-and-gels.

In your cover letter, please note whether your blot/gel image data are in Supporting Information or posted at a public data repository, provide the repository URL if relevant, and provide specific details as to which raw blot/gel images, if any, are not available. Email us at plosone@plos.org if you have any questions.

Additional Editor Comments:

The submission presented by Lin Teng et al. evaluated the role of Nesprin1 in isoproterenol-induced heart failure and its implications for cardiac pathogenesis mechanism. The results provide potential implications for understanding the molecular mechanisms of cardiac dysfunction. Overall, the study have used multiple experimental approaches, including echocardiography, immunofluorescence, Western blotting, and histological analysis, which strengthens the study. Nevertheless, there are areas that could be improved to enhance clarity, readability, and scientific rigor. Specifically, the claim on the causative role of Nespirin1 in HF is unsupported with specific experimental validation, where the findings suggested correlational implications. Suggest revising the manuscript according to reviewers comments and improve the overall paper interpretations.

[Note: HTML markup is below. Please do not edit.]

Reviewers' comments:

Reviewer's Responses to Questions

**Comments to the Author**

1. Is the manuscript technically sound, and do the data support the conclusions?

Reviewer #1: Yes

Reviewer #2: No

**********

2. Has the statistical analysis been performed appropriately and rigorously?

Reviewer #1: Yes

Reviewer #2: I Don't Know

**********

3. Have the authors made all data underlying the findings in their manuscript fully available?

The PLOS Data policy

Reviewer #1: Yes

Reviewer #2: No

**********

4. Is the manuscript presented in an intelligible fashion and written in standard English?

Reviewer #1: Yes

Reviewer #2: Yes

**********

Reviewer #1: In their study, Lin Teng et al. assessed the role of Nesprin1 in isoproterenol-induced heart failure and its implications for cardiac pathogenesis mechanism, which results provide potential implications for understanding the molecular mechanisms of cardiac dysfunction.

Overall, the study appears to be well-structured and scientifically sound. Authors used multiple experimental approaches, including echocardiography, immunofluorescence, Western blotting, and histological analysis, which strengthens the study, but there are areas that could be improved to enhance clarity, readability, and scientific rigor, and improve the overall paper.

Minors comments

Throughout the manuscript:

The English is understandable, but not highly polished. Many sentences are wordy and awkwardly structured, and some terms are misused. A professional language review is needed to improve fluency and scientific clarity.

Exemple L221. The authors wrote: "The ross and pathological examination". did they mean "The gross pathological examination"?

Many other places.

Exemple L364 “significantly reduction” should be corrected to “significant reduction.” A careful proofreading of the section for grammatical accuracy is recommended.

In the result presentation, the authors mention "significant" differences. These sentences should explicitly state the precise p-values and statistical tests.

Overall, and specifically in the discussion section:

Some sentences are complex and could benefit from restructuring to enhance readability. For example, the transition between the introduction of Nesprin1 and its connection to HF pathogenesis could be smoother.

Ensure that the argument follows a logical progression, from the established burden of HF to the novel contributions of this study, in the discussion section.

Major comments:

The authors suggest that Nesprin1 downregulation is causative, but this is not experimentally proven, unless I did not read correctly (authors did not use genetic knockdown or RNAi), wherease, the findings are correlational (i.e., Nesprin1 is reduced in heart failure). Would suggest the authors to clarify this limitation.

The Authors suggest that reduced Nesprin1 leads to ERK activation. Would you please elaborate with more mechanistic evidence?

While increased MDA and decreased SOD suggest oxidative stress, the study does not establish whether oxidative stress is the cause or consequence of Nesprin1 downregulation.

I understand the discussion highlights the significance of Nesprin1, however, it would be beneficial to include a comparison with other known mechanisms of HF progression. More explicit connections between previous research and the current findings could strengthen the justification for Nesprin1’s role.

I would suggest the authors to add more reference to prior studies when applicable) regarding the discussion on Nesprin1’s involvement in oxidative stress, nuclear homeostasis, and the ERK pathway.

Overall, the study is scientifically sound but needs improved language, better statistical transparency, and stronger mechanistic evidence to fully support its conclusions.

Major revisions are recommended to improve the manuscript’s clarity, presentation, and interpretation of findings.

Reviewer #2: Abstract:

1. BNP should be spelled out as the term was stated the first time in the article

2. CX43 and Nesprin 1 expression were both determined using immunofluorescence analysis, hence can be stated in one sentence.

3. BNP, CX43 and Nesprin 1 should be briefly introduced in the abstract.

4. Please state the cardiac parameters examined.

5. Any gene manipulation done before the conclusion made by stating that “This study elucidates the critical role of Nesprin1 in isoproterenol-induced……” ?

Introduction

The background and rationale were sufficiently addressed

Materials and Methods:

General comments: Technical details are not sufficient

Drugs and Chemicals

Catalog no. of the chemicals/products used were not stated

Establishment of Isoproterenol-Induced Heart Failure Model in Mice

Sex and Age of the animals were not stated

Cardiac Function Assessment

Specific name of the statistical test was not stated

Measurement of Body and Heart Weight

Clarify the timing of which the mice were anesthetized

The Biochemical Kits Detects The Oxidative Stress Indicators

Please simplify the demonstration of protein concentration calculation

Immunofluorescence Staining Protocol for Mouse Cardiac Tissue

The model of the microscope and the software were not stated

Western Blot Experimental Procedure

1. The catalog no and the company which RIPA buffer, phosphate and protease inhibitors were purchased from should be stated

2. Details of gel documentation system and densitometry analysis software should be stated

Results:

1. Sample size determination (animal numbers) was not demonstrated/explained

2. Western blot in Figure 2 was not labelled.

3. Specific statistical test for each assay was not stated clearly

4. Importantly knockdown of Nesprin 1 was not demonstrated and hence the data doesn’t support the stated results

Discussion:

The data provided doesn’t support the discussion as knockdown model of Nesprin 1 was not used

Declarations:

Ethics approval and consent to participate

Animal ethics approval no. was not stated in the main text

Availability of Data and Material:

While original western blots were provided, other minimal data sets were not included in the supplement information sheet

**********

what does this mean?). If published, this will include your full peer review and any attached files.

If you choose “no”, your identity will remain anonymous but your review may still be made public.

**Do you want your identity to be public for this peer review?**  For information about this choice, including consent withdrawal, please see our Privacy Policy

Reviewer #1: No

Reviewer #2: No

**********

[NOTE: If reviewer comments were submitted as an attachment file, they will be attached to this email and accessible via the submission site. Please log into your account, locate the manuscript record, and check for the action link "View Attachments". If this link does not appear, there are no attachment files.]

While revising your submission, please upload your figure files to the Preflight Analysis and Conversion Engine (PACE) digital diagnostic tool, https://pacev2.apexcovantage.com/. PACE helps ensure that figures meet PLOS requirements. To use PACE, you must first register as a user. Registration is free. Then, login and navigate to the UPLOAD tab, where you will find detailed instructions on how to use the tool. If you encounter any issues or have any questions when using PACE, please email PLOS at figures@plos.org. Please note that Supporting Information files do not need this step.

Attachment Submitted filename: Reviewer Comments PLOS ONE.pdf

# Author comment: Author response to Decision Letter 1

**DOI:** 10.1371/journal.pone.0354482.r002

19 Nov 2025

Dear Editor,

We sincerely thank you and the reviewers for valuable comments and the support extended to our manuscript. We deeply appreciate your dedication and professionalism throughout this process. In accordance with the guidelines, we have provided detailed responses to the reviewers' comments point by point below(Changes are marked in red in the manuscript).

Reviewer #1

Minor comment:

Comment 1: Throughout the manuscript:The English is understandable, but not highly polished. Many sentences are wordy and awkwardly structured, and some terms are misused. A professional language review is needed to improve fluency and scientific clarity.Exemple L221. The authors wrote: "The ross and pathological examination". did they mean "The gross pathological examination"?Many other places.Exemple L364 “significantly reduction” should be corrected to “significant reduction.” A careful proofreading of the section for grammatical accuracy is recommended.

Response 1:Thanks for your constructive feedback on our manuscript. We appreciate your careful assessment of the grammar and language issues. Each sentence has been carefully checked and revised to improve readability.The red indicates the modified text.

Comment 2: In the result presentation, the authors mention "significant" differences. These sentences should explicitly state the precise p-values and statistical tests.

Response 2:Thank you very much for your valuable suggestions. We have made changes to the original text according to your suggestions.

Comment 3: Overall, and specifically in the discussion section:Some sentences are complex and could benefit from restructuring to enhance readability. For example, the transition between the introduction of Nesprin1 and its connection to HF pathogenesis could be smoother.

Response 3: We feel great thanks for your professional review work on our article.We have made corresponding modifications to the discussion section according to your suggestions. You can see the specific modifications in the original text.

Comment 4: Ensure that the argument follows a logical progression, from the established burden of HF to the novel contributions of this study, in the discussion section.

Response 4:We sincerely thank you for your careful reading.In the discussion section, we first outlined the significant risks of heart failure and the necessity of conducting research on it. We then confirmed the successful construction of the heart failure mouse model by analyzing the basic physiological parameters, pathological staining, and cardiac color Doppler ultrasound results in mice. Next, by analyzing various pathological manifestations in heart failure mice, we explored potential directions for the role of Nesprin-1 in the disease process. Finally, we summarized the innovations and limitations of this study.

Major comments:

Comment 1: The authors suggest that Nesprin1 downregulation is causative, but this is not experimentally proven, unless I did not read correctly (authors did not use genetic knockdown or RNAi), wherease, the findings are correlational (i.e., Nesprin1 is reduced in heart failure). Would suggest the authors to clarify this limitation.

Response 1:Thank you for your valuable and practical suggestions. As you understand, this study has confirmed a series of pathological phenomena in heart failure mice with reduced Nesprin1 levels, which may be associated with the downregulation of Nesprin-1. However, due to certain objective reasons, this conclusion has not been experimentally verified. This limitation is addressed in the revised discussion. We will use additional techniques in future studies to further investigate this area.

Comment 2: The Authors suggest that reduced Nesprin1 leads to ERK activation. Would you please elaborate with more mechanistic evidence? I understand the discussion highlights the significance of Nesprin1, however, it would be beneficial to include a comparison with other known mechanisms of HF progression. More explicit connections between previous research and the current findings could strengthen the justification for Nesprin1’s role.I would suggest the authors to add more reference to prior studies when applicable) regarding the discussion on Nesprin1’s involvement in oxidative stress, nuclear homeostasis, and the ERK pathway.

Response 2: Sincerely thank you for your insights into the paper. I understand the rationality of your suggestion to add a comparison of Nesprin1 with other mechanisms of heart failure and a more detailed explanation of the mechanism. After careful discussion by the team, we believe that this study is the first exploratory work to reveal the expression changes of Nesprin1 in ISO-induced heart failure models and its association with nuclear homeostasis imbalance and oxidative stress. Maintaining the existing discussion framework will better highlight the original value of this new finding.Regarding the molecular mechanism of Nesprin1 down-regulation and ERK activation, this is indeed a question of great scientific value, but considering that this study mainly focuses on establishing phenotypic associations, detailed mechanism analysis will be the focus of our follow-up research using the gene knockout animal model we are building. Your valuable suggestions have pointed to important follow-up research directions for us, including systematic comparison of the relationship between Nesprin1 and other heart failure pathways, and in-depth analysis of the molecular mechanisms of its regulation of ERK pathways.

We earnestly hope that you will understand the limitations of the current research phase, and we commit to further exploring these key scientific questions in future work.

Comment 3: While increased MDA and decreased SOD suggest oxidative stress, the study does not establish whether oxidative stress is the cause or consequence of Nesprin1 downregulation.

Response 3:I sincerely appreciate this insightful comment. My data demonstrate that ISO-induced heart failure mice with Nesprin-1 downregulation exhibit increased MDA and decreased SOD activity, indicating enhanced oxidative stress. However, as the reviewer astutely noted, the current study cannot definitively determine whether oxidative stress drives or results from Nesprin-1 suppression.

I recognize that Nesprin-1’s role in cardiac pathology remains underexplored, with limited existing literature. Nevertheless, given heart failure’s clinical significance and prior evidence of Nesprin-1’s functional importance in other disease contexts, along with its myocardial-specific isoform expression patterns, this study was designed to investigate three key aspects: Nesprin-1’s expression profile in ISO-induced HF, its potential functional contributions to disease progression, and possible mechanistic pathways.

These findings establish an initial association between Nesprin-1 dysregulation and oxidative stress in HF, creating a foundation for future mechanistic studies. I am currently generating Nesprin-1 knockout mice to specifically examine the causal relationship between Nesprin-1 deficiency and oxidative stress, elucidate the involved molecular pathways, and characterize its precise regulatory role in HF pathogenesis. I look forward to sharing these more definitive results in future work.

Comment 4:Overall, the study is scientifically sound but needs improved language, better statistical transparency, and stronger mechanistic evidence to fully support its conclusions.

Response 4:Thank you for your valuable comments. I have made the corresponding changes.

Comment 5:Major revisions are recommended to improve the manuscript’s clarity, presentation, and interpretation of findings.

Response 5:Thank you very much for your valuable suggestions. In the original text, I have selected a clearer picture and modified the language expression and result explanation of the whole text to improve the readability of the article.

Reviewer #2:

Comment 1:Abstract:

1. BNP should be spelled out as the term was stated the first time in the article

2. CX43 and Nesprin 1 expression were both determined using immunofluorescence analysis, hence can be stated in one sentence.

3. BNP, CX43 and Nesprin 1 should be briefly introduced in the abstract.

4. Please state the cardiac parameters examined.

5. Any gene manipulation done before the conclusion made by stating that “This study elucidates the critical role of Nesprin1 in isoproterenol-induced……” ?

Response 1:Thank you very much for your suggestions and questions. Firstly, the issues with the wording in the abstract have been corrected in the original text. Secondly, regarding whether gene manipulation was performed, my response is that although gene mice were constructed, this experiment did not include this part. The primary goal of this study is to provide a research foundation for further studies on the specific mechanisms of Nesprin-1 in the development of heart failure. Therefore, no gene manipulation was conducted in this study.

Comment 2:Introduction

The background and rationale were sufficiently addressed

Response 2:I sincerely thank you for your full affirmation of the background and argumentation basis in the introduction of this study. I am very glad that the scientific basis and clinical relevance of this study have been clearly and effectively expounded.

Comment 3:Materials and Methods:

General comments: Technical details are not sufficient

Response 3:Thank you for your valuable suggestions. Under your guidance, I have modified the details of the materials and methods section in the article. See the red font for the specific content.

Comment 4:Drugs and Chemicals

Catalog no. of the chemicals/products used were not stated

Response 4:Thank you for pointing this out. I have added the catalogue number of the important chemicals/products in the relevant section.

Comment 5:Establishment of Isoproterenol-Induced Heart Failure Model in Mice

Sex and Age of the animals were not stated

Response 5:Thank you for your suggestion. In line 140 of the article, it has been stated that the animals used in this study are male C57BL/6 mice with a weight of 20-22g.

Comment 6:Cardiac Function Assessment

Specific name of the statistical test was not stated

Response 6:Thank you for your suggestion. The relevant testing methods have been explained in the last part of the methodology.

Comment 7:Measurement of Body and Heart Weight

Clarify the timing of which the mice were anesthetized

Response 7:Thank you for raising this concern. In this study, mouse body weight was measured using an electronic scale while the animals were conscious, thereby avoiding the effects of repeated anesthesia. All mice were euthanized 24 hours after the final administration, followed by cardiac tissue collection and weighing. No anesthetic agents were used during any body weight measurements throughout the study.

Comment 8:The Biochemical Kits Detects The Oxidative Stress Indicators

Please simplify the demonstration of protein concentration calculation

Response 8:Thank you! I have simplified the MDA/SOD calculation formula in the original text.

Comment 9:Immunofluorescence Staining Protocol for Mouse Cardiac Tissue

The model of the microscope and the software were not stated

Response 9:Thank you for your insightful comments on the manuscript.I have updated the microscope model and software used for immunofluorescence in this article.

Comment 10:Western Blot Experimental Procedure:1. The catalog no and the company which RIPA buffer, phosphate and protease inhibitors were purchased from should be stated. 2. Details of gel documentation system and densitometry analysis software should be stated

Response 10:Thank you for your very meaningful suggestion. According to your instructions, the relevant parts have been described in the section of Materials and Methods respectively.

Comment 11:Results:1. Sample size determination (animal numbers) was not demonstrated/explained2. Western blot in Figure 2 was not labelled.3. Specific statistical test for each assay was not stated clearly4. Importantly knockdown of Nesprin 1 was not demonstrated and hence the data doesn’t support the stated results.Discussion:The data provided doesn’t support the discussion as knockdown model of Nesprin 1 was not used

Response 11:I sincerely appreciate your valuable comments on our manuscript. We have carefully examined all the issues you raised and made corresponding revisions. Regarding the sample size, we have supplemented the figure legends in the Results section to clarify that the sample size meets statistical requirements. For the oversight in labeling Figure 2 Western Blot, we have re-examined the original data and improved the labeling of experimental groups, molecular weight markers, and internal reference protein bands.

In terms of statistical methods, we have added clarification that two-sample t-tests were used for normally distributed data (confirmed by Shapiro-Wilk test) and Mann-Whitney U tests for non-normally distributed data. We fully understand your concern regarding the validation of Nesprin 1 knockdown efficiency. This study primarily focuses on preliminary exploration of Nesprin-1 expression changes and their pathological significance in the ISO-induced heart failure model. We are currently developing Nesprin-1 knockout mouse models to enable more systematic mechanistic studies in the future.

The key findings of this study include: significant downregulation of Nesprin-1 expression in the ISO-induced heart failure model, accompanied by pathological phenomena such as nuclear homeostasis imbalance and aggravated oxidative stress, which may be related to ERK signaling pathway activation. We sincerely accept your suggestions, which are invaluable for improving the completeness of this research. While this study has certain limitations, it establishes an important foundation for subsequent mechanistic investigations. We are fully prepared to provide any additional materials as needed.

Comment 12:Declarations:Ethics approval and consent to participate;Animal ethics approval no. was not stated in the main text;Availability of Data and Material:While original western blots were provided, other minimal data sets were not included in the supplement information sheet

Response 12: I sincerely appreciate your valuable feedback regarding ethical approval and data accessibility in this paper. Your suggestions to enhance research transparency and reproducibility are of great importance to me. Concerning the animal ethics review, I have supplemented complete information in the "Materials and Methods" section: "All mice were housed in a Specific Pathogen-Free (SPF) facility at China's Three Gorges University (Yichang, Hubei Province). The experimental animal quality certification number is 42010200009428, and the experimental animal use permit number is 00305510. All procedures complied with international standards for laboratory animal care and use." Regarding data availability, although I have provided all Western blot raw images in the supplementary materials, I recognize the importance of additional data. The foundational datasets supporting key research findings have been systematically organized and are available to corresponding authors upon request. This clarification has been incorporated into the "Data Availability Statement" section of the paper. Thank you for your attention to these critical aspects of my work, and I hope this explanation addresses your concerns. If any other supplementary materials are required, I will provide them at any time.

We have carefully considered all the comments and suggestions and have made the necessary revisions to improve the quality and clarity of our manuscript. We hope that the changes we've made address the concerns raised by the reviewers to your satisfaction. Simultaneously, we are also eagerly looking forward to your reply.

Thank you once again for your time and consideration.

Sincerely,

Teng Lin

Department of Cardiology/Yichang centre people's hospital

The First College of China Three Gorges University

No.183, Yiling Road, Yichang, 443003, China

Email:tenglin@ctgu.edu.cn

# Editor report: Decision Letter 1

**DOI:** 10.1371/journal.pone.0354482.r003

**Authors:** Wei Hsum Yap

19 Dec 2025

Dear Dr. Teng,

Thank you for submitting your manuscript to PLOS ONE. After careful consideration, we feel that it has merit but does not fully meet PLOS ONE’s publication criteria as it currently stands. Therefore, we invite you to submit a revised version of the manuscript that addresses the points raised during the review process.

==============================

While the authors have made some amendments in the revision, there are still several major flaws in the manuscript which require attention:

1. The ethics application and review board for the project was not included, specifically the institutional ethics review board (IACUC) was not mentioned. In addition, there were discrepancy and confusion in the information provided: the manuscript indicated that The experimental animal quality certification number is 42010200009428 and The experimental animal use permit number is 00305510, but the information provided to the journal submission was as below: The animal review number is 2023660H. All experimental animals were euthanized after being anesthetized with anesthetic drugs. Please review and revise accordingly.

2. The manuscript have not addressed to the comments from both reviewers to indicate that reduced expression of Nesprin-1 is an observation, not a causative outcome from the ISO-induced HF model. Though the authors have included a paragraph indicating the limitations, there were multiple occurrences where the sentences constructed led to misinterpretation and overclaim. Please review and revise.

3. There were numerous grammar errors noted in the manuscript, suggest proofreading the article for enhanced clarity.

==============================

Please submit your revised manuscript by Feb 02 2026 11:59PM. If you will need more time than this to complete your revisions, please reply to this message or contact the journal office at plosone@plos.org. When you're ready to submit your revision, log on to https://www.editorialmanager.com/pone/ and select the 'Submissions Needing Revision' folder to locate your manuscript file.

- A letter that responds to each point raised by the academic editor and reviewer(s). You should upload this letter as a separate file labeled 'Response to Reviewers'.
- A marked-up copy of your manuscript that highlights changes made to the original version. You should upload this as a separate file labeled 'Revised Manuscript with Track Changes'.
- An unmarked version of your revised paper without tracked changes. You should upload this as a separate file labeled 'Manuscript'.

If you would like to make changes to your financial disclosure, please include your updated statement in your cover letter. Guidelines for resubmitting your figure files are available below the reviewer comments at the end of this letter.

If applicable, we recommend that you deposit your laboratory protocols in protocols.io to enhance the reproducibility of your results. Protocols.io assigns your protocol its own identifier (DOI) so that it can be cited independently in the future. For instructions see: https://journals.plos.org/plosone/s/submission-guidelines#loc-laboratory-protocols. Additionally, PLOS ONE offers an option for publishing peer-reviewed Lab Protocol articles, which describe protocols hosted on protocols.io. Read more information on sharing protocols at https://plos.org/protocols?utm_medium=editorial-email&utm_source=authorletters&utm_campaign=protocols.

We look forward to receiving your revised manuscript.

Kind regards,

Wei Hsum Yap

Academic Editor

PLOS One

Journal Requirements:

If the reviewer comments include a recommendation to cite specific previously published works, please review and evaluate these publications to determine whether they are relevant and should be cited. There is no requirement to cite these works unless the editor has indicated otherwise.

Additional Editor Comments:

While the authors have made some amendments in the revision, there are still several major flaws in the manuscript which require attention:

1. The ethics application and review board for the project was not included, specifically the institutional ethics review board (IACUC) was not mentioned. In addition, there were discrepancy and confusion in the information provided: the manuscript indicated that The experimental animal quality certification number is 42010200009428 and The experimental animal use permit number is 00305510, but the information provided to the journal submission was as below: The animal review number is 2023660H. All experimental animals were euthanized after being anesthetized with anesthetic drugs. Please review and revise accordingly.

2. The manuscript have not addressed to the comments from both reviewers to indicate that reduced expression of Nesprin-1 is an observation, not a causative outcome from the ISO-induced HF model. Though the authors have included a paragraph indicating the limitations, there were multiple occurrences where the sentences constructed led to misinterpretation and overclaim. Please review and revise.

3. There were numerous grammar errors noted in the manuscript, suggest proofreading the article for enhanced clarity.

[Note: HTML markup is below. Please do not edit.]

To ensure your figures meet our technical requirements, please review our figure guidelines: https://journals.plos.org/plosone/s/figures

You may also use PLOS’s free figure tool, NAAS, to help you prepare publication quality figures: https://journals.plos.org/plosone/s/figures#loc-tools-for-figure-preparation.

NAAS will assess whether your figures meet our technical requirements by comparing each figure against our figure specifications.

Attachment Submitted filename: PONE-D-25-08527_R1.pdf

# Author comment: Author response to Decision Letter 2

**DOI:** 10.1371/journal.pone.0354482.r004

20 Apr 2026

Response to Editorial Office Requests

Manuscript ID: PONE-D-25-08527R2

Title: Exploring the Role of Nesprin-1 in Isoproterenol-Induced Heart Failure: Insights into Pathogenesis and Therapeutic Implications

Dear Editor,

Thank you for the opportunity to revise our manuscript to meet PLOS ONE’s submission requirements. We have carefully addressed all the issues raised in your email. Please find our point-by-point responses below.

Point 1: Please upload a Response to Reviewers letter which should include a point by point response to each of the points made by the Editor and / or Reviewers.

Response: We have prepared and uploaded this point-by-point response letter as requested.

Reviewer #1

Minor comment:

Comment 1: Throughout the manuscript:The English is understandable, but not highly polished. Many sentences are wordy and awkwardly structured, and some terms are misused. A professional language review is needed to improve fluency and scientific clarity.Exemple L221. The authors wrote: "The ross and pathological examination". did they mean "The gross pathological examination"?Many other places.Exemple L364 “significantly reduction” should be corrected to “significant reduction.” A careful proofreading of the section for grammatical accuracy is recommended.

Response 1:Thanks for your constructive feedback on our manuscript. We appreciate your careful assessment of the grammar and language issues. Each sentence has been carefully checked and revised to improve readability.The red indicates the modified text.

Comment 2: In the result presentation, the authors mention "significant" differences. These sentences should explicitly state the precise p-values and statistical tests.

Response 2:Thank you very much for your valuable suggestions. We have made changes to the original text according to your suggestions.

Comment 3: Overall, and specifically in the discussion section:Some sentences are complex and could benefit from restructuring to enhance readability. For example, the transition between the introduction of Nesprin1 and its connection to HF pathogenesis could be smoother.

Response 3: We feel great thanks for your professional review work on our article.We have made corresponding modifications to the discussion section according to your suggestions. You can see the specific modifications in the original text.

Comment 4: Ensure that the argument follows a logical progression, from the established burden of HF to the novel contributions of this study, in the discussion section.

Response 4:We sincerely thank you for your careful reading.In the discussion section, we first outlined the significant risks of heart failure and the necessity of conducting research on it. We then confirmed the successful construction of the heart failure mouse model by analyzing the basic physiological parameters, pathological staining, and cardiac color Doppler ultrasound results in mice. Next, by analyzing various pathological manifestations in heart failure mice, we explored potential directions for the role of Nesprin-1 in the disease process. Finally, we summarized the innovations and limitations of this study.

Major comments:

Comment 1: The authors suggest that Nesprin1 downregulation is causative, but this is not experimentally proven, unless I did not read correctly (authors did not use genetic knockdown or RNAi), wherease, the findings are correlational (i.e., Nesprin1 is reduced in heart failure). Would suggest the authors to clarify this limitation.

Response 1:Thank you for your valuable and practical suggestions. As you understand, this study has confirmed a series of pathological phenomena in heart failure mice with reduced Nesprin1 levels, which may be associated with the downregulation of Nesprin-1. However, due to certain objective reasons, this conclusion has not been experimentally verified. This limitation is addressed in the revised discussion. We will use additional techniques in future studies to further investigate this area.

Comment 2: The Authors suggest that reduced Nesprin1 leads to ERK activation. Would you please elaborate with more mechanistic evidence? I understand the discussion highlights the significance of Nesprin1, however, it would be beneficial to include a comparison with other known mechanisms of HF progression. More explicit connections between previous research and the current findings could strengthen the justification for Nesprin1’s role.I would suggest the authors to add more reference to prior studies when applicable) regarding the discussion on Nesprin1’s involvement in oxidative stress, nuclear homeostasis, and the ERK pathway.

Response 2: Sincerely thank you for your insights into the paper. I understand the rationality of your suggestion to add a comparison of Nesprin1 with other mechanisms of heart failure and a more detailed explanation of the mechanism. After careful discussion by the team, we believe that this study is the first exploratory work to reveal the expression changes of Nesprin1 in ISO-induced heart failure models and its association with nuclear homeostasis imbalance and oxidative stress. Maintaining the existing discussion framework will better highlight the original value of this new finding.Regarding the molecular mechanism of Nesprin1 down-regulation and ERK activation, this is indeed a question of great scientific value, but considering that this study mainly focuses on establishing phenotypic associations, detailed mechanism analysis will be the focus of our follow-up research using the gene knockout animal model we are building. Your valuable suggestions have pointed to important follow-up research directions for us, including systematic comparison of the relationship between Nesprin1 and other heart failure pathways, and in-depth analysis of the molecular mechanisms of its regulation of ERK pathways.

We earnestly hope that you will understand the limitations of the current research phase, and we commit to further exploring these key scientific questions in future work.

Comment 3: While increased MDA and decreased SOD suggest oxidative stress, the study does not establish whether oxidative stress is the cause or consequence of Nesprin1 downregulation.

Response 3:I sincerely appreciate this insightful comment. My data demonstrate that ISO-induced heart failure mice with Nesprin-1 downregulation exhibit increased MDA and decreased SOD activity, indicating enhanced oxidative stress. However, as the reviewer astutely noted, the current study cannot definitively determine whether oxidative stress drives or results from Nesprin-1 suppression.

I recognize that Nesprin-1’s role in cardiac pathology remains underexplored, with limited existing literature. Nevertheless, given heart failure’s clinical significance and prior evidence of Nesprin-1’s functional importance in other disease contexts, along with its myocardial-specific isoform expression patterns, this study was designed to investigate three key aspects: Nesprin-1’s expression profile in ISO-induced HF, its potential functional contributions to disease progression, and possible mechanistic pathways.

These findings establish an initial association between Nesprin-1 dysregulation and oxidative stress in HF, creating a foundation for future mechanistic studies. I am currently generating Nesprin-1 knockout mice to specifically examine the causal relationship between Nesprin-1 deficiency and oxidative stress, elucidate the involved molecular pathways, and characterize its precise regulatory role in HF pathogenesis. I look forward to sharing these more definitive results in future work.

Comment 4:Overall, the study is scientifically sound but needs improved language, better statistical transparency, and stronger mechanistic evidence to fully support its conclusions.

Response 4:Thank you for your valuable comments. I have made the corresponding changes.

Comment 5:Major revisions are recommended to improve the manuscript’s clarity, presentation, and interpretation of findings.

Response 5:Thank you very much for your valuable suggestions. In the original text, I have selected a clearer picture and modified the language expression and result explanation of the whole text to improve the readability of the article.

Reviewer #2:

Comment 1:Abstract:

1. BNP should be spelled out as the term was stated the first time in the article

2. CX43 and Nesprin 1 expression were both determined using immunofluorescence analysis, hence can be stated in one sentence.

3. BNP, CX43 and Nesprin 1 should be briefly introduced in the abstract.

4. Please state the cardiac parameters examined.

5. Any gene manipulation done before the conclusion made by stating that “This study elucidates the critical role of Nesprin1 in isoproterenol-induced……” ?

Response 1:Thank you very much for your suggestions and questions. Firstly, the issues with the wording in the abstract have been corrected in the original text. Secondly, regarding whether gene manipulation was performed, my response is that although gene mice were constructed, this experiment did not include this part. The primary goal of this study is to provide a research foundation for further studies on the specific mechanisms of Nesprin-1 in the development of heart failure. Therefore, no gene manipulation was conducted in this study.

Comment 2:Introduction

The background and rationale were sufficiently addressed

Response 2:I sincerely thank you for your full affirmation of the background and argumentation basis in the introduction of this study. I am very glad that the scientific basis and clinical relevance of this study have been clearly and effectively expounded.

Comment 3:Materials and Methods:

General comments: Technical details are not sufficient

Response 3:Thank you for your valuable suggestions. Under your guidance, I have modified the details of the materials and methods section in the article. See the red font for the specific content.

Comment 4:Drugs and Chemicals

Catalog no. of the chemicals/products used were not stated

Response 4:Thank you for pointing this out. I have added the catalogue number of the important chemicals/products in the relevant section.

Comment 5:Establishment of Isoproterenol-Induced Heart Failure Model in Mice

Sex and Age of the animals were not stated

Response 5:Thank you for your suggestion. In line 140 of the article, it has been stated that the animals used in this study are male C57BL/6 mice with a weight of 20-22g.

Comment 6:Cardiac Function Assessment

Specific name of the statistical test was not stated

Response 6:Thank you for your suggestion. The relevant testing methods have been explained in the last part of the methodology.

Comment 7:Measurement of Body and Heart Weight

Clarify the timing of which the mice were anesthetized

Response 7:Thank you for raising this concern. In this study, mouse body weight was measured using an electronic scale while the animals were conscious, thereby avoiding the effects of repeated anesthesia. All mice were euthanized 24 hours after the final administration, followed by cardiac tissue collection and weighing. No anesthetic agents were used during any body weight measurements throughout the study.

Comment 8:The Biochemical Kits Detects The Oxidative Stress Indicators

Please simplify the demonstration of protein concentration calculation

Response 8:Thank you! I have simplified the MDA/SOD calculation formula in the original text.

Comment 9:Immunofluorescence Staining Protocol for Mouse Cardiac Tissue

The model of the microscope and the software were not stated

Response 9:Thank you for your insightful comments on the manuscript.I have updated the microscope model and software used for immunofluorescence in this article.

Comment 10:Western Blot Experimental Procedure:1. The catalog no and the company which RIPA buffer, phosphate and protease inhibitors were purchased from should be stated. 2. Details of gel documentation system and densitometry analysis software should be stated

Response 10:Thank you for your very meaningful suggestion. According to your instructions, the relevant parts have been described in the section of Materials and Methods respectively.

Comment 11:Results:1. Sample size determination (animal numbers) was not demonstrated/explained2. Western blot in Figure 2 was not labelled.3. Specific statistical test for each assay was not stated clearly4. Importantly knockdown of Nesprin 1 was not demonstrated and hence the data doesn’t support the stated results.Discussion:The data provided doesn’t support the discussion as knockdown model of Nesprin 1 was not used

Response 11:I sincerely appreciate your valuable comments on our manuscript. We have carefully examined all the issues you raised and made corresponding revisions. Regarding the sample size, we have supplemented the figure legends in the Results section to clarify that the sample size meets statistical requirements. For the oversight in labeling Figure 2 Western Blot, we have re-examined the original data and improved the labeling of experimental groups, molecular weight markers, and internal reference protein bands.

In terms of statistical methods, we have added clarification that two-sample t-tests were used for normally distributed data (confirmed by Shapiro-Wilk test) and Mann-Whitney U tests for non-normally distributed data. We fully understand your concern regarding the validation of Nesprin 1 knockdown efficiency. This study primarily focuses on preliminary exploration of Nesprin-1 expression changes and their pathological significance in the ISO-induced heart failure model. We are currently developing Nesprin-1 knockout mouse models to enable more systematic mechanistic studies in the future.

The key findings of this study include: significant downregulation of Nesprin-1 expression in the ISO-induced heart failure model, accompanied by pathological phenomena such as nuclear homeostasis imbalance and aggravated oxidative stress, which may be related to ERK signaling pathway activation. We sincerely accept your suggestions, which are invaluable for improving the completeness of this research. While this study has certain limitations, it establishes an important foundation for subsequent mechanistic investigations. We are fully prepared to provide any additional materials as needed.

Comment 12:Declarations:Ethics approval and consent to participate;Animal ethics approval no. was not stated in the main text;Availability of Data and Material:While original western blots were provided, other minimal data sets were not included in the supplement information sheet

Response 12: I sincerely appreciate your valuable feedback regarding ethical approval and data accessibility in this paper. Your suggestions to enhance research transparency and reproducibility are of great importance to me. Concerning the animal ethics review, I have supplemented complete information in the "Materials and Methods" section: "All mice were housed in a Specific Pathogen-Free (SPF) facility at China's Three Gorges University (Yichang, Hubei Province). The experimental animal quality certification number is 42010200009428, and the experimental animal use permit number is 00305510. All procedures complied with international standards for laboratory animal care and use." Regarding data availability, although I have provided all Western blot raw images in the supplementary materials, I recognize the importance of additional data. The foundational datasets supporting key research findings have been systematically organized and are available to corresponding authors upon request. This clarification has been incorporated into the "Data Availability Statement" section of the paper. Thank you for your attention to these critical aspects of my work, and I hope this explanation addresses your concerns. If any other supplementary materials are required, I will provide them at any time.

Point 2: Your abstract cannot contain citations. Please remove any existing citations from the abstract section. You may only include citations in the body text of the manuscript, and please ensure that they remain in ascending numerical order on first mention.

Response: We have thoroughly checked the manuscript and completely removed all citations from th

Attachment Submitted filename: Response to Reviewers.docx

# Editor report: Decision Letter 2

**DOI:** 10.1371/journal.pone.0354482.r005

**Authors:** Wei Hsum Yap

5 May 2026

Dear Dr. Teng,

Thank you for submitting your manuscript to PLOS ONE. After careful consideration, we feel that it has merit but does not fully meet PLOS ONE’s publication criteria as it currently stands. Therefore, we invite you to submit a revised version of the manuscript that addresses the points raised during the review process.

Please submit your revised manuscript by Jun 19 2026 11:59PM. If you will need more time than this to complete your revisions, please reply to this message or contact the journal office at plosone@plos.org. When you're ready to submit your revision, log on to https://www.editorialmanager.com/pone/ and select the 'Submissions Needing Revision' folder to locate your manuscript file.

- A letter that responds to each point raised by the academic editor and reviewer(s). You should upload this letter as a separate file labeled 'Response to Reviewers'.
- A marked-up copy of your manuscript that highlights changes made to the original version. You should upload this as a separate file labeled 'Revised Manuscript with Track Changes'.
- An unmarked version of your revised paper without tracked changes. You should upload this as a separate file labeled 'Manuscript'.

If you would like to make changes to your financial disclosure, please include your updated statement in your cover letter. Guidelines for resubmitting your figure files are available below the reviewer comments at the end of this letter.

If applicable, we recommend that you deposit your laboratory protocols in protocols.io to enhance the reproducibility of your results. Protocols.io assigns your protocol its own identifier (DOI) so that it can be cited independently in the future. For instructions see: https://journals.plos.org/plosone/s/submission-guidelines#loc-laboratory-protocols. Additionally, PLOS ONE offers an option for publishing peer-reviewed Lab Protocol articles, which describe protocols hosted on protocols.io. Read more information on sharing protocols at https://plos.org/protocols?utm_medium=editorial-email&utm_source=authorletters&utm_campaign=protocols.

As the corresponding author, your ORCID iD is verified in the submission system and will appear in the published article. PLOS supports the use of ORCID, and we encourage all coauthors to register for an ORCID iD and use it as well. Please encourage your coauthors to verify their ORCID iD within the submission system before final acceptance, as unverified ORCID iDs will not appear in the published article. *Only*  the individual author can complete the verification step; PLOS staff *cannot*  verify ORCID iDs on behalf of authors.

We look forward to receiving your revised manuscript.

Kind regards,

Wei Hsum Yap

Academic Editor

PLOS One

Journal Requirements:

If the reviewer comments include a recommendation to cite specific previously published works, please review and evaluate these publications to determine whether they are relevant and should be cited. There is no requirement to cite these works unless the editor has indicated otherwise.

Please review your reference list to ensure that it is complete and correct. If you have cited papers that have been retracted, please include the rationale for doing so in the manuscript text, or remove these references and replace them with relevant current references. Any changes to the reference list should be mentioned in the rebuttal letter that accompanies your revised manuscript. If you need to cite a retracted article, indicate the article’s retracted status in the References list and also include a citation and full reference for the retraction notice.

Additional Editor Comments:

The authors have addressed the mechanistic weakness or lack of causality limitations in their results analysis and discussion. Nevertheless, the manuscript still requires professional language editing where there are confusion in the subtitles of the results section, as well as the revised sections in the discussion (duplications and repetition in the limitations of current study). This has resulted in difficulty in following through the manuscript flow and scientific concepts. Please have the manuscript edited by a professional English language editing service prior to resubmission.

[Note: HTML markup is below. Please do not edit.]

[NOTE: If reviewer comments were submitted as an attachment file, they will be attached to this email and accessible via the submission site. Please log into your account, locate the manuscript record, and check for the action link "View Attachments". If this link does not appear, there are no attachment files.]

To ensure your figures meet our technical requirements, please review our figure guidelines: https://journals.plos.org/plosone/s/figures

You may also use PLOS’s free figure tool, NAAS, to help you prepare publication quality figures: https://journals.plos.org/plosone/s/figures#loc-tools-for-figure-preparation.

NAAS will assess whether your figures meet our technical requirements by comparing each figure against our figure specifications.

# Author comment: Author response to Decision Letter 3

**DOI:** 10.1371/journal.pone.0354482.r006

19 Jun 2026

Dear Editor,

Thank you for providing us with the opportunity to resubmit our manuscript titled "Exploring the Role of Nesprin-1 in Isoproterenol-Induced Heart Failure: Insights into Pathogenesis and Therapeutic Implications" (Manuscript ID: PONE-D-25-08527R2).

We have carefully addressed all the technical and language concerns raised in the previous editorial correspondence. In addition to the requested revisions, we would like to clarify three specific administrative changes:

1. Reinstatement of the Manuscript

First, we sincerely apologize for a technical error during the submission process. The "Decline to Resubmit" option was accidentally selected. We have since reinstated the submission and wish to confirm our continued commitment to the publication process in PLOS ONE. We apologize for any confusion this may have caused the editorial office.

2. Update to Institutional Affiliation

The nomenclature of the primary institution has been updated to " Department of Cardiology, The First College of Clinical Medical Science, China Three Gorges University & Yichang Central People's Hospital, Yichang, China". This change was made to strictly adhere to the latest official standardized naming conventions issued by our university for all newly published research.

3. Response to Editorial and Language Comments

3.1. "The manuscript still requires professional language editing..."

Response: We entirely agree. Prior to this resubmission, the entire manuscript underwent comprehensive professional English language editing. We have corrected grammatical errors, resolved tense inconsistencies particularly in the Methods section , fixed plural/singular misuses, and eliminated awkward phrasing to ensure scientific concepts are communicated clearly and accurately.

3.2. "...where there are confusion in the subtitles of the results section..."

Response: Thank you for pointing this out. We reviewed the subtitles in the Results section and found that their formatting was indeed inconsistent. We have now standardized all subtitles in the Results section into concise, unified noun phrases to improve readability and structural flow.

3.3. "...as well as the revised sections in the discussion (duplications and repetition in the limitations of current study). This has resulted in difficulty in following through the manuscript flow and scientific concepts."

Response: We apologize for the redundancy in our previous draft. We have carefully reviewed and rewritten the Discussion section, with a specific focus on the "Limitations" paragraph. We removed the repetitive statements regarding the lack of direct causality and the observational nature of the study. The revised Discussion is now more concise, and the logical flow connecting Nesprin-1 downregulation to specific pathological features has been significantly improved. Sentence fragments and structural redundancies have been fully resolved.

4. Response to Journal Requirements:

4.1. Reference List Review:

Response: As requested, we have thoroughly reviewed our reference list. We confirm that the reference list is complete, properly formatted, and correct. We have verified that none of the cited papers have been retracted.

4.2. Figure Guidelines Check:

Response: We have reviewed all figures against the journal's technical requirements using the provided guidelines and the PLOS figure tool (NAAS). All figures have been checked to ensure they meet the publication quality standards regarding resolution, format, and legibility.

We hope that these comprehensive revisions have satisfactorily addressed your concerns and that the manuscript is now suitable for publication in your journal. Thank you again for your time and continuous support.

5. Update to Authorship

We have added one co-author, mengpan liu, to the manuscript. This individual provided significant intellectual contributions during this revision stage, particularly in the extensive professional language editing, structural reorganization of the Results and Discussion sections, and critical review of the final version. Crucially, we wish to state that all original authors have reached a unanimous consensus regarding this addition. There are no disputes over authorship. If required by the Editorial Office, we are prepared to provide a formal document signed by all authors to verify this agreement.

Best regards,

Dr. Teng Lin

Department of Cardiology, Yichang Central People's Hospital

College of Basic Medical Sciences, China Three Gorges University

E-mail: tenglin@ctgu.edu.cn

Attachment Submitted filename: response.docx

# Editor report: Decision Letter 3

**DOI:** 10.1371/journal.pone.0354482.r007

**Authors:** Wei Hsum Yap

8 Jul 2026

Exploring the Role of Nesprin1 in Isoproterenol-Induced Heart Failure�Insights into Pathogenesis and Therapeutic Implications

PONE-D-25-08527R3

Dear Dr. Teng,

We’re pleased to inform you that your manuscript has been judged scientifically suitable for publication and will be formally accepted for publication once it meets all outstanding technical requirements.

Within one week, you’ll receive an e-mail detailing the required amendments. When these have been addressed, you’ll receive a formal acceptance letter and your manuscript will be scheduled for publication.

An invoice will be generated when your article is formally accepted. Please note, if your institution has a publishing partnership with PLOS and your article meets the relevant criteria, all or part of your publication costs will be covered. Please make sure your user information is up-to-date by logging into Editorial Manager at Editorial Manager® and clicking the ‘Update My Information' link at the top of the page. For questions related to billing, please contact billing support.

If your institution or institutions have a press office, please notify them about your upcoming paper to help maximize its impact. If they’ll be preparing press materials, please inform our press team as soon as possible -- no later than 48 hours after receiving the formal acceptance. Your manuscript will remain under strict press embargo until 2 pm Eastern Time on the date of publication. For more information, please contact onepress@plos.org.

Kind regards,

Wei Hsum Yap

Academic Editor

PLOS One

Additional Editor Comments (optional):

Reviewers' comments:

# Editor report: Acceptance letter

**DOI:** 10.1371/journal.pone.0354482.r008

**Authors:** Wei Hsum Yap

PONE-D-25-08527R3

PLOS One

Dear Dr. Teng,

I'm pleased to inform you that your manuscript has been deemed suitable for publication in PLOS One. Congratulations! Your manuscript is now being handed over to our production team.

At this stage, our production department will prepare your paper for publication. This includes ensuring the following:

* All references, tables, and figures are properly cited

* All relevant supporting information is included in the manuscript submission,

* There are no issues that prevent the paper from being properly typeset

You will receive further instructions from the production team, including instructions on how to review your proof when it is ready. Please keep in mind that we are working through a large volume of accepted articles, so please give us a few days to review your paper and let you know the next and final steps.

Lastly, if your institution or institutions have a press office, please let them know about your upcoming paper now to help maximize its impact. If they'll be preparing press materials, please inform our press team within the next 48 hours. Your manuscript will remain under strict press embargo until 2 pm Eastern Time on the date of publication. For more information, please contact onepress@plos.org.

You will receive an invoice from PLOS for your publication fee after your manuscript has reached the completed accept phase. If you receive an email requesting payment before acceptance or for any other service, this may be a phishing scheme. Learn how to identify phishing emails and protect your accounts at https://explore.plos.org/phishing.

If we can help with anything else, please email us at customercare@plos.org.

Thank you for submitting your work to PLOS ONE and supporting open access.

Kind regards,

PLOS ONE Editorial Office Staff

on behalf of

Dr. Wei Hsum Yap

Academic Editor

PLOS One
